# Women experiencing homelessness and mental illness in a Housing First multi-site trial: Looking beyond housing to social outcomes and well-being

**DOI:** 10.1371/journal.pone.0277074

**Published:** 2023-02-10

**Authors:** Patricia O’Campo, Rosane Nisenbaum, Anne G. Crocker, Tonia Nicholls, Faith Eiboff, Carol E. Adair

**Affiliations:** 1 MAP Centre for Urban Health Solutions, St Michael’s Hospital, Toronto, Canada; 2 Dalla Lana School of Public Health, University of Toronto, Toronto, Canada; 3 Institut National de Psychiatrie Légale Philippe-Pinel, Montreal, Canada; 4 Department of Psychiatry & Addictions, Université de Montréal, Montreal, Canada; 5 School of Criminology, Université de Montréal, Montreal, Canada; 6 British Columbia Mental Health & Substance Use Services, Provincial Health Services Authority, Vancouver, Canada; 7 Department of Psychiatry, University of British Columbia, Vancouver, Canada; 8 Interdisciplinary Studies, School of Population and Public Health, University of British Columbia, Vancouver, Canada; 9 Department of Psychiatry, Cumming School of Medicine, University of Calgary, Calgary, Canada; 10 Department of Community Health Sciences, Cumming School of Medicine, University of Calgary, Calgary, Canada; SUNY Downstate Health Sciences University, UNITED STATES

## Abstract

**Objective:**

There is scant research on the effectiveness of permanent supportive housing for homeless women with mental illness. This study examines the effectiveness of Housing First with an unprecedentedly large sample of homeless women from five Canadian cities, and explore baseline risk factors that predict social, health and well-being outcomes over a 24 month-period.

**Methods:**

The At Home/Chez Soi multi-site randomized controlled Housing First trial recruited over 600 women between October 2009 and July 2011. This is a post-hoc subgroup exploratory analysis of self-identified women with at least one follow-up interview who were randomized to Housing First (HF) (*n* = 374) or treatment-as-usual (TAU) (*n* = 279) and had at least one follow-up interview. Linear mixed models and generalized estimating equations were used after multiple imputation was applied to address missing data.

**Results:**

At the end of follow-up, the mean percentage of days spent stably housed was higher for women in the intervention 74.8% (95%CI = 71.7%–77.8%) compared with women in the treatment-as-usual group, 37.9% (95%CI = 34.4%–41.3%), *p*<0.001. With few exceptions, social and mental health outcomes were similar for both groups at 6-, 12-, 18- and 24-months post-enrollment. Suicidality was a consistent predictor of increased mental health symptoms (beta = 2.85, 95% CI 1.59–4.11, p<0.001), decreased quality of life (beta = -3.99, 95% CI -6.49 to -1.49, p<0.001), decreased community functioning (beta = -1.16, 95% CI -2.10 to -0.22, p = 0.015) and more emergency department visits (rate ratio = 1.44, 95% CI 1.10–1.87, p<0.001) over the study period. Lower education was a predictor of lower community functioning (beta = -1.32, 95% CI -2.27 to -0.37, p = 0.006) and higher substance use problems (rate ratio = 1.27, 95% CI 1.06–1.52, p = 0.009) during the study.

**Conclusions:**

Housing First interventions ensured that women experiencing homelessness are quickly and consistently stably housed. However, they did not differentially impact health and social measures compared to treatment as usual. Ensuring positive health and social outcomes may require greater supports at enrolment for subgroups such as those with low educational attainment, and additional attention to severity of baseline mental health challenges, such as suicidality.

**Trial registration:**

International Standard Randomized Control Trial Number Register Identifier: ISRCTN42520374.

## Introduction

Homelessness in Canada is primarily experienced by men, yet data show that over one-third of the homeless people in this country are women and close to 90% of the single parents who are homeless are women [1–3]. Mental health and substance use disproportionately affect women experiencing homelessness. Between 20–50% experience major depressive disorder and major depressive episodes as demonstrated in systematic reviews [4], between 30–40% experience post-traumatic stress disorder [5, 6], as many as one in five report moderate-to-high levels of suicidality [6], and a large proportion experience exceptional vulnerability to an array of health and social risks [6–11], including premature mortality [12]. A recent meta-analysis of observational studies in high-income countries reports that three-quarter of homeless individuals have a mental illness diagnosis with no differences by gender [13].

Women who are homeless have different health and social vulnerabilities compared to homeless men. They have higher rates of distress including suicidal ideation [14], greater experiences of trauma prior to and during episodes of homelessness including more adverse childhood events (ACEs) such as physical and sexual abuse and trauma-related disorders [6, 15–22], few options for effective woman-centred services and treatments for these traumatic events and other ailments, all of which predispose them to homelessness [14, 23–27]. They are also substantially more likely than homeless men to engage in survival sex work and are thus more vulnerable to exploitation, violence, infectious disease, gynecological problems and unwanted pregnancies [6, 14]. Homeless women with children express considerable traumatic distress because of frequent child apprehensions [28]. Because women who are homeless often seek shelter in informal settings such as couch surfing or sleeping rough, they are less visible and have less access to services [14, 29, 30] and have received less attention in research and policy and practice.

Program planners and policy makers operate in a context of too little gender-specific data on programs and interventions and weak studies addressing the experiences of women [3, 4, 31]. As such, there have been calls for more research that moves away from gender-neutral approaches (i.e., examining men’s and women’s experiences with the same lens, as if there are no gender differences) [14, 31–33] towards more gender-sensitive frameworks, including women-only analyses that capture the variability around the unique challenges women who are homeless face and accelerating evidence-informed practice with this population [3, 29, 34, 35]. For interventions, there is a need for studies with large enough samples to examine whether and how programs work for women [4, 36–41].

Currently, with some exceptions [42], what we do know about housing interventions for women experiencing homelessness comes from small subsamples in longitudinal observational studies [34, 43], randomized trial pilot [44], or qualitative evaluations [32, 45]. A large multi-site supportive housing demonstration project for chronically homeless adults in 11 US communities, with 173 women as part of the sample, reported substantial improvements in housing stability but no gender differences in this and other service utilization or clinical outcomes over two years of follow-up [46]. In an observational study of an integrated service system initiative in 18 sites across the US, women (N = 2727) demonstrated greater improvements in family relationships and social support compared to men (N = 4502) over 18-months of follow-up [42].

Housing First, a supportive housing approach with demonstrated success in transitioning individuals experiencing chronic homelessness and mental illness into permanent housing, has negligible evidence on outcomes for women [47–50]. The Housing First model is based on harm reduction principles of choice and self-determination, and provides immediate access to scattered-site housing through the provision of rent supplements and flexible recovery-oriented supports [49, 51]. Participation in Housing First is not contingent on participation in services or requirements of ‘housing readiness’ (e.g., sobriety or medication compliance) [49].

The At Home/Chez Soi randomized trial conducted in five cities across Canada provides an unprecedented opportunity to examine the impact of a housing first initiative among women for several reasons. First, it is the largest randomized trial of a Housing First initiative and includes an unprecedented sample of several hundred women. This large sample size facilitates a woman only analysis to focus on variability within this subsample and factors that are relevant for women living with homelessness such as sources of trauma or parental roles. A woman only analysis, too rarely seen in studies of homelessness, ensures that the focus remains on women within the sample as gender analyses largely focus on comparing experiences with those of men. With 24 months of follow-up and novel risk variables not previously examined in a Housing First analysis we can provide unique point-in-time and longitudinal evidence not previously considered because of prior sample size and variable availability limitations.

The present study employs the largest longitudinal sample of women experiencing homelessness and mental illness in a Housing First randomized controlled trial (RCT) and extends the follow up duration typically seen to 24 months. Our objectives were: (i) To compare change at the end of the study in health and social outcomes between women who received and did not receive the Housing First intervention; ii) To examine whether the Housing First intervention leads to better housing stability over 24 months, and health and social and outcomes between 6- to 24-months post enrolment; and, (iii) To explore baseline demographic, social, health measures, and ACEs as predictors of key study outcomes during the study period.

## Methods

Methods and measures for this analysis have been extensively described elsewhere [48, 52–54]. Briefly, the Canadian At Home/Chez Soi five city RCT randomized Housing First among adults experiencing homelessness and serious mental illness [53]. Participants randomized to the Housing First (HF) intervention received scattered-site housing and either moderate or high needs-based services to support their recovery and integration into the community (i.e., Intensive Case Management or Assertive Community Treatment). Participants randomized to treatment-as-usual (TAU) had access to housing and services through other community programs. Each site had additional focus on a unique population (e.g., ethno-racial populations in Toronto and Indigenous populations in Winnipeg) and had specialized services to address their needs. Fidelity assessments ensured strong adherence to program theory of change [55]. Information about the study, such as the randomization process and a description of the intervention, was provided to all study participants prior to enrolment. An informed consent process was created to ensure that participants had the time and information to understand the complexities of the trial and follow-up activities. All participants provided written informed consent prior to enrolment. All enrolment and consent documents were harmonized across sites, however, each site had separate approval from relevant university/site Research Ethics Boards.

Over 24 months, participants were interviewed every 3 months to assess housing stability and every 6 months to evaluate health and social outcomes. Previous results from 2,148 participants (including 683 women) demonstrated that those in the HF arm showed positive improvements in housing stability, quality of life, and community functioning compared to participants in usual care [46, 48, 52]. This paper reports on an exploratory analysis of 653 participants who self-identified as women and had at least one follow-up after their baseline assessment (96%), including 588 followed to 24 months. Across all sites, 374 participants were from the HF group and 279 from the TAU group (S1 Fig in S1 File).

### Outcomes and other study variables

We describe in this section the outcome variables and any other scales used in this analysis. Stable housing was defined as “living in one’s own room, apartment, or house, or with family, with an expected duration of residence of 6 months or more or tenancy rights” while enrolled in the study, taking into account participants’ move in/out dates for each type of residence (e.g., street place, unstable residence, stable residence, institution) [56]. The *percentage of days stably housed during the 24-month period* was calculated as the total number of days stably housed divided by the total number of days for which any type of residence data were provided by the participant over 24 months and varied between 0% and 100% [48, 53]. Unlike the other outcomes, housing stability was measured at the end of the follow-up period for several reasons. First, the treatment group establishes stable housing within the first 1–3 months of enrolment and housing status rarely changes after that point in time. A small proportion of the treatment group was not consistently stably housed throughout the duration of the program; thus, this single cumulative measure captures housing stability better than if we had measured it at each follow-up time point. In sum, this cumulative housing stability is the best measure for the outcome of housing as it does not demonstrate incremental changes over the 24-month follow-up period.

The other outcomes were assessed via in-person interviews at baseline, 6-, 12-, 18, and 24 months post-enrollment.

The Lehman Quality of Life Interview 20 (QoLI-20) measured participants’ condition-specific quality of life. Twenty items scored on a 7-point Likert scale define a total quality of life and domains of family, finance, leisure, living, safety, social, and overall quality of life. In this work, we used the total score calculated as the sum of all 20 items, which ranges from 20 to 140, with higher scores indicating better quality of life [57].

Community functioning was measured using the Multnomah Community Ability Scale (MCAS), a 17-item scale capturing total community functioning and degree of clinician/observer-rated functional ability in domains of health, adaptation, social skills, and behavior. In this work, we used the total score calculated as the sum of all 17 times, ranging from 17–85, with higher scores indicating less impairment [48].

The Colorado Symptom Index (CSI) assessed how often specific psychiatric symptoms were experienced, from ‘at least every day’ to ‘not at all’. Fourteen self-report items were summed (range 14 to 40), with lower scores reflecting fewer symptoms [58, 59].

Emergency department (ED) visits were measured using self-reported counts in the past 6 months. The number of past-month substance-related problems were measured via the 5-item Global Assessment of Individual Needs Short Screener (GAIN-SS); higher counts correspond to greater problem severity [53].

The Community Integration Scale (CIS) measured self-reported psychological integration with the immediate community on a scale from 4–20; physical integration used counts of previous month social activities (e.g., attending a concert, meeting people for coffee) on a scale of 0–7. Higher scores on both reflect greater integration [60].

Baseline and predictor variables included data collected on traumatic events that occurred before the age of 18 years using the Adverse Childhood Experiences module assessing ten early childhood adversities (yes/no items): emotional, physical or sexual abuse; emotional or physical neglect; household substance use, household mental illness and household criminal justice involvement; mother treated violently, and parental separation or divorce [61]. Scores ranged from 0 to 10 with higher scores indicating greater childhood adversity. We created two categories representing high levels of reported ACEs, ≥5 adversities, and four or fewer including none [62, 63]. Mental health diagnoses and levels of suicidality were assessed via the MINI Neuropsychiatric Interview 6.0 administered as part of the baseline enrolment interview [64]. No, low, moderate and high levels of suicidality were dichotomized into ‘no/low’ and ‘moderate/high’ suicidality. Information about all other variables (e.g., demographics, financial support of children, sources of income) was obtained via self-report during the in-person baseline interviews.

### Statistical analyses

Descriptive statistics for a wide range of descriptors were selected to characterize the sample by treatment group. Longitudinal outcomes were analyzed using linear mixed analysis of repeated measures models for continuous outcomes and generalized estimating equations for count outcomes (Poisson distribution for CIS-Physical Integration and negative binomial distribution for GAIN-SS and ED Visits), assuming an unstructured correlation matrix for the repeated measures. Percentage of days stably housed over the 24-months follow-up (range 0–100%) was modeled as a continuous outcome in a linear model. We also performed a sensitivity analysis by considering the proportion (0–1) of days stably housed and fitting a fractional logistic model. The fractional logistic model, which uses a quasi-likelihood function for estimation, was introduced by Papke and Woolridge (1996) [65] and is appropriate for proportions when the values of 0 and 1 are admissible and observable. The fractional logistic model predicts the mean of the proportion conditional on covariates. To ensure that the predicted mean is also between 0 and 1, the logit model for the mean is considered. The parameters estimated from this model can be exponentiated to yield odds ratios and 95% confidence intervals for the association between stable housing during the 24 months follow-up and covariates.

For the first objective, for each group, the adjusted change from baseline to 24-months follow-up was estimated by mean differences and 95% confidence intervals (continuous outcomes), and rate ratios and 95% confidence intervals (count outcomes). We fit models that included treatment group (fixed effect, HF vs TAU), categorical time (24-months vs baseline), interaction of treatment x time, and study city and need level at baseline (high vs. moderate) as covariates.

For the second objective, in a more detailed examination of possible differential effect over time, we tested for differences between HF and TAU at each follow-up time point from 6- to 24-months, adjusting for baseline outcomes. Except for percentage (or proportion) of days stably housed over 24 months, models included treatment group, categorical time (6-, 12-, 18-, 24-months), interaction of treatment x time, and city, need level and baseline outcome values as covariates. At each time point, we compared HF vs TAU by estimating mean differences and 95% confidence intervals or rate ratios and 95% confidence intervals. For percentage (or proportion) of days stably housed over 24-months we included treatment group, city, need level and percentage of days stably housed in the three months prior to baseline as covariates.

For the third objective, the differences between HF and TAU were no longer the focus. Rather we took advantage of the large sample of women in a study on homelessness to identify predictors of our outcomes. To accomplish this we expanded the models from the second objective and added the following baseline predictors: age at enrolment (continuous), education (1 = less than high school, 0 = high school or higher), duration of homelessness (1 = ≥3, 0 = <3 years), whether minor children were being supported by the participant (1 = yes, 0 = no), level of suicidality (1 = moderate or high, 0 = mild or none), and high level of ACEs at baseline (1 = ≥5, 0 = <5 ACEs). These predictors were chosen because they represent a knowledge gap or have yielded inconsistent findings on longitudinal studies of women experiencing homelessness. We were unable to include ethnicity or Indigenous identity due to multicollinearity with study city.

Multiple imputation with chained equations (MICE) was used to handle missing data from loss to follow-up, withdrawal, skipped interviews, nonresponse on specific items, and systematically measured lack of interviewer confidence in participant responses. We imputed all outcomes simultaneously and MICE imputation models included all variables needed to address three objectives [66]. Although outcome missing rates varied between 0.53% to 31.9% (S1 Table in S1 File), approximately 70% of the participants had at least one missing value in at least one outcome in at least one point in time. Therefore, we chose 70 imputations following guidelines given by White et al. (2010) that the percentage of cases with incomplete data should be similar to the number of imputations generated [66]. Comparisons between observed and imputed data via diagnostic plots were used to assess the quality and plausibility of the imputations [67].

Data were imputed using Stata software (mi impute chained, Stata Statistical Software: Release 13. College Station, TX: StataCorp LP) and analyzed using SAS (PROC MIXED, GENMOD, GLIMMIX, PROC MIANALYZE, SAS 9.4, SAS Institute Inc, Cary, NC). SAS Syntax is provided in S2 Table in S1 File. Figures were created using the ggplot2 package in R software version 3.4.0 (R Core Team (2017) [68]. All statistical tests were two-tailed and significance was defined as *p* < .05. No adjustments for multiple testing were applied [69].

## Results

We took advantage of the rich data collected in this study and present a broad array of participant demographic, social, and health characteristics at enrolment (Table 1). Fifteen percent of the women were born outside of Canada (median time in Canada: TAU = 25yrs; HF = 21yrs). Three percent of women were employed at baseline and 90% received social assistance of some type as a source of income. Around 40% (*n* = 274) reported having minor children, and of these, 22% (*n* = 60) were providing full or partial financial support to the child(ren). The most prevalent mental illnesses were depressive episodes, PTSD, and alcohol or substance dependence. Approximately 40% had moderate-to-high suicidality, around 40% had post-traumatic stress disorder, about 60% reported ≥5 ACEs, and the majority had three or more co-morbid medical conditions.

**Table 1:** Baseline characteristics of women participants in the Canadian At Home/Chez Soi study by randomization group (*N* = 653).

Characteristics	Treatment as Usual (N = 279)		Housing First (N = 374)	
	N	%	N	%
Age (M ± SD), y	40.2 ± 11.7		39.7 ± 11.2	
Ethnic or cultural identity				
Aboriginal	76	27.2	95	25.4
Ethno-racial	67	24.0	81	21.7
White	136	48.8	198	52.9
Education				
Less than high school	130	46.9	202	54.3
Completed high school	57	20.6	61	16.4
Some post-secondary school	90	32.5	109	29.3
Marital status				
Married/Partnered	22	7.9	16	4.3
Divorced/Separated/Widowed	74	26.5	106	28.4
Single/Never married	183	65.6	251	67.3
Country of birth, & length of time in Canada for immigrants				
Born in Canada	235	84.8	315	84.2
Immigrant, 10 years or less	8	2.8	12	3.2
Immigrant, more than 10 years	34	12.3	47	12.6
Employment status				
Unemployed (unemployed, volunteer work, retired, student, housewife/husband, other)	270	96.8	362	97.3
Employed (including self-employed and special work program)	9	3.2	10	2.7
Sources of income				
Earnings from regular/casual work or busking	16	5.7	18	4.8
Unemployment insurance	22	7.9	26	7.0
Social assistance (disability, welfare, pension, personal needs allowance)	245	87.8	338	90.4
Selling papers, crafts, pan-handling, squeegee, or $ from recycling (bottles, scrap metal)	16	5.7	22	5.9
Other (loans, theft, sex work, help from family or friends)	12	4.3	10	2.7
Housing status				
Absolutely Homeless	215	77.1	296	79.1
Precariously housed	64	22.9	78	20.9
Lifetime duration of homelessness (M ± SD)^a^, y	3.8 ± 4.6		3.8 ± 4.8	
Less than 3 years duration	155	57.8	211	58.0
3 years or more duration	113	42.2	153	42.0
Has minor children (≤18 yrs of age)				
0	154	56.0	221	59.1
1–2	81	29.4	110	29.4
3 or more	40	14.6	43	11.5
Has minor children (≤18 yrs of age) & provides full/partial support				
0	88	72.7	125	82.2
1	20	16.5	16	10.5
2 or more	13	10.7	11	7.2
Relationship to minor children (≤ 18 yrs of age)				
Biological parent	121	100.0	151	98.7
Partner’s, adoptive or other adult relative’s child	0	0	5	3.4
MCAS^b^ score (M ± SD)	60.7 ± 8.0		61.8 ± 7.8	
MINI^c^ diagnostic categories				
Depressive episode	170	60.9	218	58.3
Manic or hypomanic episode	50	17.9	55	14.7
Post-traumatic stress disorder	126	45.3	145	38.8
Panic disorder	94	33.7	102	27.3
Mood disorder with psychotic features	54	19.4	54	14.5
Psychotic disorder	81	29.0	95	25.4
Alcohol dependence	97	34.8	108	28.9
Substance dependence	134	48.0	167	44.7
Suicidality				
Not suicidal	46	16.5	62	16.6
Low	119	42.7	170	45.5
Moderate	50	17.9	70	18.7
High	64	22.9	72	19.3
Adverse Childhood Experiences Scale, mean (SD)	5.1	3.0	5.2	3.2
Less than 5	95	39.9	137	40.5
5 or more	143	60.1	201	59.5
Comorbid medical conditions				
Less than 3	51	18.6	100	27.0
3 or more	223	81.4	270	73.0
Study Component				
Moderate Needs	144	51.6	232	62.0
High Needs	135	48.4	142	38.0

^a^ Median (IQR), y 2.0 (0.8–5.0) for Treatment as Usual and 2.0 (0.7–5.0) for Housing First;

^b^ MCAS: Multnomah Community Ability Scale. Possible scores range from 17 to 85, with lower scores indicating more disability.;

^c^ MINI: The Mini-International Neuropsychiatric Interview

Table 2 displays results from models examining changes from baseline to 24 months for those in HF and TAU arms for each of our social and well-being outcomes, and comparing groups with respect to housing stability over the 24-month period. Over the 24-month follow-up period, substantial improvements were observed for all the outcomes for both HF and TAU participants. Of note, mean total QoLI-20 scores improved 16.0 points (95%CI = 13.6–18.4) and 13.4 points (95%CI = 10.6–16.2) in the HF and TAU groups, respectively, and number of ED visits decreased by approximately 60% in both groups.

**Table 2:** Post-imputation pooled change in health and social measures from baseline to 24 months among women living with mental illness and homelessness, adjusted for study city, and need level (ACT or ICM).

	Housing First (HF) (N = 374)		Treatment as Usual (TAU) (N = 279)		p-value for differences between HF and TAU
**Continuous Outcomes (95% CI)**	Mean Change from Baseline to 24 Months	95% CI	Mean Change from Baseline to 24 Months	95% CI	p
Mental illness symptom severity (CSI^a^)	-6.3	-7.5 to -5.0	-6.9	-8.4 to -5.4	0.534
Community functioning (MCAS^b^)	3.8	2.8–4.9	4.8	3.6–6.0	0.236
Condition-specific QoL (QoLI-20^c^)	16.0	13.6–18.4	13.4	10.6–16.2	0.178
Psychological community integration (CIS^d^)	2.0	1.5–2.4	2.0	1.4–2.6	0.941
**Count Outcomes**	Rate Ratio Comparing 24 Months with Baseline	95% CI	Rate Ratio Comparing 24 Months with Baseline	95% CI	p
Physical community integration in past month (CIS^d^)	0.97	0.86–1.08	1.03	0.92–1.14	0.439
Severity of substance problems in past month (GAIN-SS^e^)	0.79	0.67–0.93	0.83	0.70–0.98	0.688
Number of emergency department visits in past 6 months	0.38	0.29–0.50	0.37	0.27–0.51	0.926

*Note*. Housing outcome is mean proportion of days spent stably housed.

^a^ CSI: Colorado Symptom Index. Possible scores range from 14 to 70, with higher values indicating greater symptom severity.

^b^ MCAS: Multnomah Community Ability Scale. Possible scores range from 17 to 85, with lower values indicating more disability.

^c^ QoLI-20: Lehman Quality of Life Interview 20. Possible scores range from 20 to 140, with higher values indicating greater quality of life.

^d^ CIS: Community Integration Scale. Possible scores range from 4 to 20 for the Psychological component and 0–7 for the Physical component, with higher values indicating greater integration.

^e^ GAIN-SS: Global Assessment of Individual Needs Short Screener. Possible scores range from 0 to 5 with higher values indicating greater problem severity.

**Fig 1:**
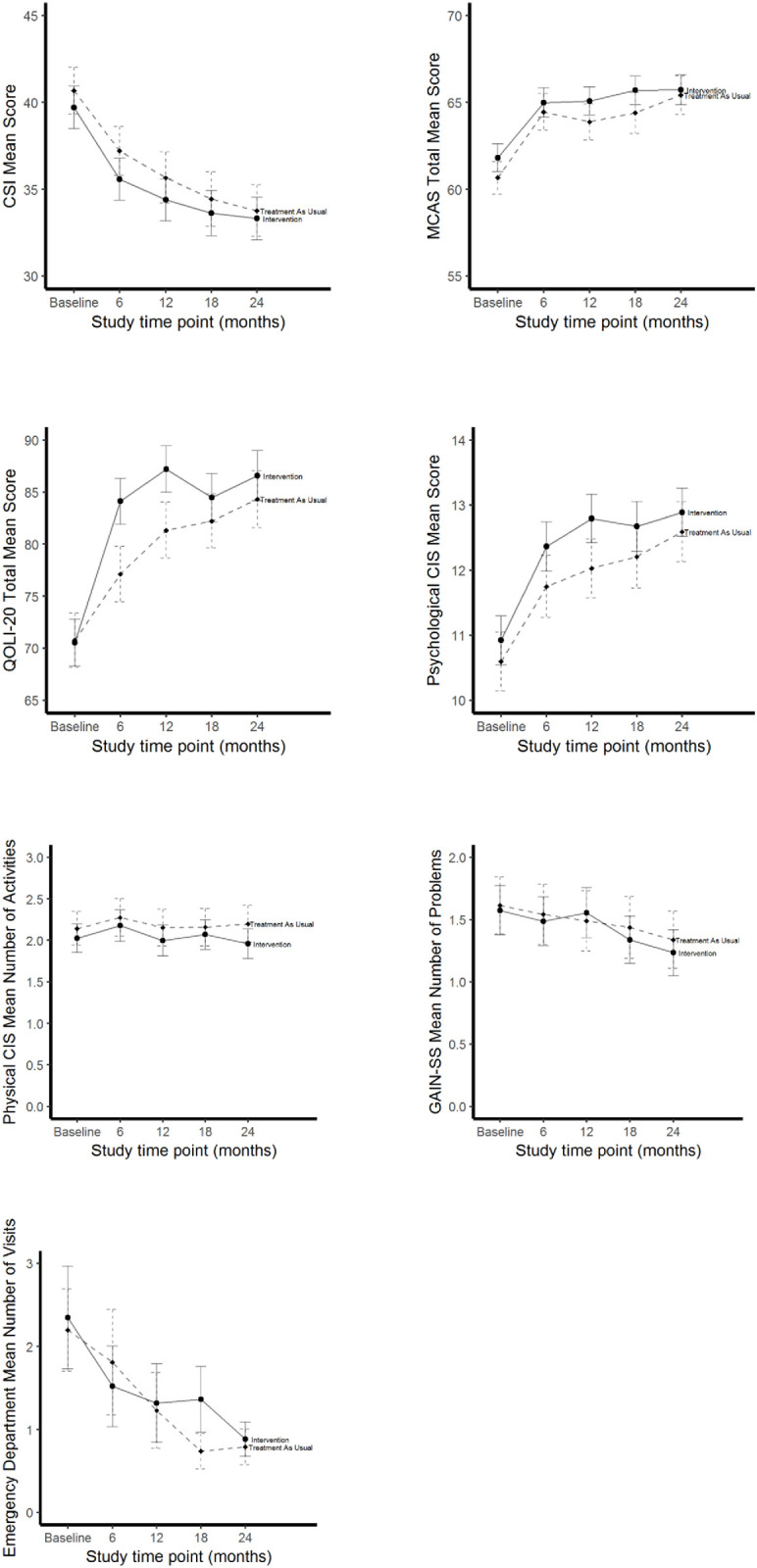
Health and social outcomes among women who are homeless and mentally ill, unadjusted means and 95% confidence intervals over 24 months of follow-up by study time point and treatment group. *Note*. *N* = 653. CSI = Colorado Symptom Index; MCAS = Multnomah Community Ability Scale; Lehman Quality of Life Interview 20 = QoLI-20; CIS = Community Integration Scale; GAIN-SS = Global Assessment of Individual Needs Short Screener.

These improvements over the 24-month period are more clearly illustrated in Fig 1 for the HF intervention and TAU groups for all social and health unadjusted outcomes at *each observation point* from baseline to 24-months. For all outcomes, except CIS-Physical where scores remained flat throughout the follow-up, both groups experienced similar significant change from baseline to 24-months (Fig 1).

The mean percentage of days spent stably housed during follow-up for women receiving HF was 74.8% (95%CI = 71.7%–77.8%) compared with 37.9% (95%CI = 34.4%–41.3%) TAU, a difference of 36.9% (95%CI = 32.4%–41.4%, *p*<0.001). In addition, the odds of stable housing during follow-up was over 5 times higher among those in the HF group than in the TAU group (OR = 5.09, 95% CI = 4.08–6.35, p<0.001). Table 3 displays changes over time in greater detail through results of the models to assessing whether social and health outcomes were better in HF vs. TAU groups *at each follow-up point*. For condition-specific quality of life, significant improvements of 7.1 and 6.1 points were observed in the HF group at the 6- and 12-month follow-ups, respectively. The HF group also showed significant improvements in psychological community integration by 0.7 points at the 6-month follow-up. However, in the last year of the trial, the ED visits fell faster for the TAU group (0.6 visits) compared to the HF group (1.1 visits) at 18 months, with a rate ratio of 1.81 (95%CI = 1.26–2.60).

**Table 3:** Post-imputation pooled comparisons between housing first and treatment as usual at 6, 12, 18 and 24 months for women in the At Home/Chez Soi trial adjusted for study city, need level (ICM or ACT) and baseline outcome values^f^.

	Month	Housing First		Treatment as Usual		Comparison		
Continuous Outcome		Mean	95% CI	Mean	95% CI	Mean Difference	95% CI	p
Mental illness symptom severity (CSI^a^)	6	35.8	34.8–36.8	36.8	35.6–38.0	-1.0	-2.5 to 0.5	0.183
	12	34.6	33.6–35.6	35.3	34.1–36.6	-0.7	-2.3 to 0.8	0.364
	18	33.8	32.7–35.0	34.1	32.7–35.4	-0.2	-2.0 to 1.5	0.785
	24	33.7	32.6–34.8	33.4	32.1–34.7	0.3	-1.4 to 2.0	0.737
Community functioning (MCAS^b^)	6	64.9	64.1–65.7	64.9	64.0–65.9	-0.02	-1.2 to 1.2	0.975
	12	65.0	64.2–65.8	64.3	63.4–65.3	0.7	-0.6 to 1.9	0.299
	18	65.6	64.8–66.5	64.9	63.8–65.9	0.8	-0.6 to 2.1	0.267
	24	65.5	64.7–66.4	65.9	64.9–66.9	-0.4	-1.7 to 0.9	0.593
Condition-specific QoL (QoLI-20^c^)	6	84.4	82.4–86.4	77.3	75.0–79.6	7.1	4.1–10.1	< .001
	12	87.4	85.4–89.4	81.3	79.0–83.7	6.1	3.1–9.2	< .001
	18	84.8	82.8–86.9	82.2	79.9–84.6	2.6	-0.4 to 5.6	0.091
	24	86.8	84.6–89.0	84.3	81.8–86.7	2.5	-0.7 to 5.8	0.124
Psychological community integration (CIS^d^)	6	12.4	12.0–12.7	11.8	11.4–12.3	0.5	-0.1 to 1.1	0.077
	12	12.8	12.4–13.2	12.1	11.6–12.5	0.7	0.1 to 1.3	0.017
	18	12.7	12.3–13.1	12.3	11.8–12.7	0.4	-0.2 to 1.0	0.194
	24	12.9	12.5–13.2	12.7	12.2–13.1	0.2	-0.4 to 0.8	0.458
**Count Outcome**		**Rate**	**95% CI**	**Rate**	**95% CI**	**Rate Ratio**	**95% CI**	**p**
Physical community integration in past month (CIS^d^)	6	2.1	1.9–2.3	2.1	1.9–2.3	0.98	0.87–1.12	0.789
	12	1.9	1.7–2.1	2.0	1.8–2.2	0.95	0.84–1.09	0.492
	18	2.0	1.8–2.1	2.0	1.8–2.2	0.99	0.87–1.12	0.858
	24	1.9	1.7–2.0	2.0	1.8–2.2	0.91	0.80–1.03	0.148
Severity of substance problems in past month (GAIN-SS^e^)	6	1.2	1.0–1.4	1.2	1.0–1.5	0.97	0.78–1.20	0.767
	12	1.3	1.1–1.5	1.2	1.0–1.4	1.09	0.87–1.37	0.452
	18	1.1	0.9–1.3	1.2	0.9–1.4	0.74	1.20–0.63	0.628
	24	1.1	0.9–1.2	1.0	0.9–1.2	1.00	0.79–1.27	0.977
Number of emergency department visits in past 6 months	6	1.2	0.9–1.4	1.4	1.0–1.9	0.80	0.55–1.16	0.241
	12	1.1	0.7–1.4	1.1	0.7–1.6	0.96	0.58–1.57	0.858
	18	1.1	0.8–1.4	0.6	0.5–0.8	1.81	1.26–2.60	0.001
	24	0.8	0.6–0.9	0.7	0.5–0.9	1.13	0.83–1.55	0.426

^a^ CSI: Colorado Symptom Index. Possible scores range from 14 to 70, with higher values indicating greater symptom severity.

^b^ MCAS: Multnomah Community Ability Scale. Possible scores range from 17 to 85, with lower values indicating more disability.

^c^ QoLI-20: Lehman Quality of Life Interview 20. Possible scores range from 20 to 140, with higher values indicating greater quality of life.

^d^ CIS: Community Integration Scale. Possible scores range from 4 to 20 for the Psychological component and 0–7 for the Physical component, with higher values indicating greater integration.

^e^ GAIN-SS: Global Assessment of Individual Needs Short Screener. Possible scores range from 0 to 5 with higher values indicating greater problem severity.

^f^ Housing stability is not included in this table as we did not examine it at each follow-up point.

Table 4 no longer focuses on differences between HF and TAU as results presented here identify predictors of our outcomes. Moderate to high suicidality had the most consistent association to poorer health and service utilization outcomes at 24 months follow-up. Compared to those with no or low suicidality at baseline, moderate-to-high suicidality at baseline was associated with nearly three points on the CSI indicating greater mental health symptom severity (beta = 2.85, 95%CI = 1.59–4.11, *p<*0.001), QoLI-20 mean scores were almost four points lower indicating reduced quality of life (beta = -3.99, 95%CI = -6.49 to -1.49, *p =* 0.002), and almost 1.5 more ED visits (rate ratio = 1.44, 95%CI = 1.10–1.87, *p =* 0.007). Low education was also associated with several outcomes. Compared to those with higher education, women with less than a high school level of education at baseline predicted lower community functioning (MCAS) scores (beta = -1.32, 95%CI = -2.27 to -0.37, *p =* 0.006), 15% fewer social activities (CIS) in the previous month (rate ratio = 0.85, 95%CI = 0.77–0.93, *p<*0.001), and more severe substance-related problems at 24 months follow-up (rate ratio = 1.27, 95%CI = 1.06–1.52, *p =* 0.009). Having been homeless for ≥3 years at baseline was associated with lower percentage days stably housed at 24-months (beta = -6.1%, 95%CI = -11.0% to -1.3%, *p =* 0.013; OR = 0.74, 95% CI 0.58–0.94, p = 0.0132). An ACE score of 5 or more was associated with a 1.5 point increase in mental illness symptom severity (95%CI = 0.25–2.76, *p =* 0.019). Finally, we explored and found no evidence that baseline levels of ACEs and suicidality, separately, were effect modifiers for any of the above analyses.

**Table 4:** Post-imputation pooled coefficient estimates (95% confidence interval) for predictors in linear mixed models for repeated measures for continuous outcomes and rate ratios (95% confidence interval) for generalized estimating equations models for count outcomes for the sample of women in the At Home/Chez Soi study (N = 653).

	Mental illness symptom severity (CSI)			Community functioning (MCAS)			Condition-specific QoL (QoLI-20)			Psychological community integration (CIS)		
	Est	95% CI	p-val	Est	95% CI	p-val	Est	95% CI	p-val	Est	95% CI	p-val
Age (years)	-0.01	-0.06–0.04	0.680	-0.02	-0.060-.02	0.310	-0.06	-0.17–0.04	0.250	-0.01	-0.03–0.00	0.140
Less than high school	0.21	-0.98–1.40	0.731	**-1.32**	**-2.27 to -0.37**	**0.006**	0.87	-1.56–3.30	0.480	0.15	-0.26–0.55	0.476
3 or more years of homelessness	0.91	-0.27–2.09	0.132	**-1.75**	**-2.72 to -0.78**	**< .001**	-0.75	-3.14–1.64	0540	-0.07	-0.48–0.33	0.728
Supports ≥1 child under 18 years	0.11	-1.89–2.10	0.917	0.62	-0.96–2.20	0.442	0.47	-3.52–4.47	0.820	0.48	-0.20–1.17	0.168
Moderate or high suicidality at baseline	**2.85**	**1.59–4.11**	**< .001**	**-1.16**	**-2.10 to -0.22**	**0.015**	**-3.99**	**-6.49 to -1.49**	**< .001**	-0.30	-0.71–0.11	0.150
5 or more ACE	**1.50**	**0.25–2.76**	**0.019**	0.21	-0.75–1.17	0.666	-0.56	-3.14–2.02	0.670	0.31	-0.12–0.73	0.157
	Physical community integration in past month (CIS) (N = 653)			Severity of substance problems in past month (GAIN-SS) (N = 653)			Number of emergency department visits in past 6 months (N = 653)			Stable housing (N = 653)		
		95% CI	p-val	RR	95% CI	p-val	RR	95% CI	p-val	beta* or Odds ratio**	95% CI	p-val
Age (years)	1.0	1.0–1.0	0.834	**0.99**	**0.98–1.00**	**0.003**	1.00	0.99–1.01	0.920	0.16	-0.04–0.36	0.122
										1.01	1.00–1,02	0.1021
Less than high school	**0.85**	**0.77–0.93**	**< .001**	**1.27**	**1.06–1.52**	**0.009**	1.11	0.83–1.49	0.474	-2.78	-7.43–1.88	0.242
										0.87	0.69–1.09	0.222
3 or more years of homelessness	0.98	0.93–1.11	0.720	**1.21**	**1.03–1.43**	**0.021**	0.99	0.76–1.29	0.951	**-6.14**	**-10.9–1.32**	**0.013**
										**0.74**	**0.58–0.94**	**0.013**
Supports ≥1 child under 18 years	1.10	0.96–1.27	0.176	1.05	0.80–1.39	0.715	0.92	0.67–1.26	0.598	5.57	-2.17–13.3	0.158
										1.31	0.89–1.92	0.171
Moderate or high suicidality at baseline	1.02	0.93–1.12	0.634	1.06	0.90–1.27	0.465	**1.44**	**1.10–1.87**	**<0.001**	-1.82	-6.49–2.80	0.440
										0.91	0.73–1.15	0.432
5 or more ACE	1.04	0.95–1.15	0.356	1.16	0.96–1.39	0.114	1.05	0.77–1.42	0.772	2.931.16	2.05–7.92 0.90–1.48	0.249 0.245

Each model includes treatment group, categorical time, interaction of treatment x time, city, need level, baseline outcome values and all baseline predictors. For percentage or proportion of days stably housed over 24 months, time and interaction treatment by time are not included in linear* and fractional logistic models**

## Discussion

This is the first analysis of women’s outcomes in an RCT of Housing First. Prior studies and trials on housing interventions for people who experience homelessness often included very small numbers of women or few variables reflecting women’s unique needs and experiences that have precluded documentation of the heterogeneity of experiences and in-depth understanding of this population.

In this Canadian multi-site study, the largest longitudinal sample of women experiencing homelessness to date, the intervention group experienced housing stability for 75% of the 24-month follow-up, as compared to 38% for women in the TAU group, a finding similar to previously published reports of At Home/Chez Soi participants, the majority of whom were men [48, 52]. Even if the large proportion of time spent stably housed for women who received Housing First compared to the TAU group did not translate into greater improvements in health and social outcomes by the end of the study, there were some early differences favoring the treatment group in the first year for quality of life and psychological community integration. Immediate placement in housing and early housing stability for HF compared to TAU [70] participants likely contributed to faster improvements in these outcomes.

While ED visits declined in a similar fashion for HF and TAU over the 24-month follow-up, an unanticipated finding was a significant reduction in ED visits of 0.5 visits at 18 months for the TAU group over the HF group. For this rare count outcome, the dip in ED visits for TAU at 18 months could be a chance improvement as both groups had similar levels of ED visits by 24 months. Analyses of the full At Home/Chez Soi trial data did not show differences for this outcome [48]. One possibility is that uncertainty around continued housing as the trial was ending may have created high levels of stress in the Housing First group, but if this were the case, we would have likely seen similar changes in other study outcomes or among men in the analyses of the full sample.

Since improvements in outcomes over the 24-month period were similar for both the HF and TAU groups, we moved beyond the intention-to-treat analysis and examined baseline predictors for our outcomes across the full sample. We focused on baseline suicidality, ACEs, and parental responsibilities as predictors- variables rarely examined in past studies of supported housing or Housing First [34, 42–44, 71, 72]. As might be expected, women with ≥5 ACEs or moderate-to-high levels of suicidality at baseline showed poorer improvement by 24 months compared to those with low ACE or suicidality scores. Moderate-to-high suicidality was a strong predictor of poorer outcomes at 24 months, including mental health, quality of life, community integration, and ED visits. Past cross-sectional studies of combined samples of homelessness men and women have demonstrated similar links between suicidality, childhood and adult trauma, substance abuse, and mental health [20, 21, 73]. However, while cross-sectional associations between suicidality and substance among women who are homeless have been reported in the literature [19, 74] our study is the first to examine these associations longitudinally.

Contrary to what we might have anticipated on the importance of ACEs in predicting multiple study outcomes given past research [75], we found that high levels of ACEs only predicted significantly worse scores on mental illness severity (CSI). This is consistent with a recent study of the full sample of At Home/Chez Soi where ACEs total scores predicted several mental health conditions at 24-months [50]. Our choice of a cutoff representing high numbers of ACEs, while informed by existing literature, may have limited our ability to detect an association. Alternatively, ACEs may be limited in capturing relevant adverse conditions. A recent study has suggested that an expanded ACEs tool might be used to capture important domains currently missing such as severe poverty or family loss and separation [76], which might be particularly relevant in capturing sources of trauma and adversity for women experiencing homelessness. Our findings also suggested that low educational attainment at baseline was a significant predictor of worse mental health, community integration, and substance use outcomes independently of the other variables in the model. Low educational attainment may be a proxy for other factors such as low health literacy which might impact management of health conditions and requires further investigation [77]. Providing economic support for dependent children was not related to any of our study outcomes.

Although the At Home/Chez Soi support services were important for intervention participants to remain stably housed and make gains in health and social standing, these supports and services were not designed in this Housing First program to be tailored to women’s unique needs. Psychological housing stability which includes considerations of safety, security and feelings of home, is particularly important for women while the At Home/Chez Soi focused predominantly on material housing stability [78]. Women who are unstably housed or homeless need services that address trauma and promote informal peer support networks [32, 79]. Although staff were trained to be responsive to individual needs, the At Home/Chez Soi intervention was not specifically designed to provide the latter two resources. In addition, more than 40% of our participants were homeless for more than three years at enrolment. Two years of stable housing with mental health supports may not have been sufficient to resolve chronic distress from being homeless with co-morbid health, trauma and substance use problems, particularly given the high rates of reported PTSD and ACEs [14].

The lack of greater improvements in the HF arm in this analysis might have been due to the intensive follow-up schedule that could have led to a Hawthorne effect among the TAU group, and higher loss to follow-up in the TAU group might have contributed to smaller differences between groups at 24 months. Despite our sample of homeless women being relatively large, it may not have been sufficient to detect small effects. The At Home/Chez Soi study did not capture gender-sensitive outcomes specific to women experiencing homelessness and therefore we may have missed important changes, such as those associated with positive social support, increased safety from crime and victimization, or strengthening of relationships with children, family or friends [3, 80].

Our study adds to the growing yet sparse literature on the characteristics and needs of women experiencing homelessness and the effectiveness of interventions to eliminate homelessness. Given that women make up more than one third of the homeless population, and their needs differ from that of men [3, 14, 27, 31], it is important that their experiences with homelessness and recovery receive ample focus to better inform the design and implementation of interventions that meet their unique needs. Moreover, the homelessness epidemic has only worsened in the context of the COVID pandemic so the issues remain relevant and these new analyses can provide insights [81].

## Supporting information

S1 File (DOCX)
