# Supplementary material for: Women experiencing homelessness and mental illness in a Housing First multi-site trial: Looking beyond housing to social outcomes and well-being
Source: PLoS One. 2023 Feb 10;18(2):e0277074. doi: 10.1371/journal.pone.0277074 (PMC9916643; doi:10.1371/journal.pone.0277074)
Supplement: S1 File — (DOCX) [file pone.0277074.s001.docx]

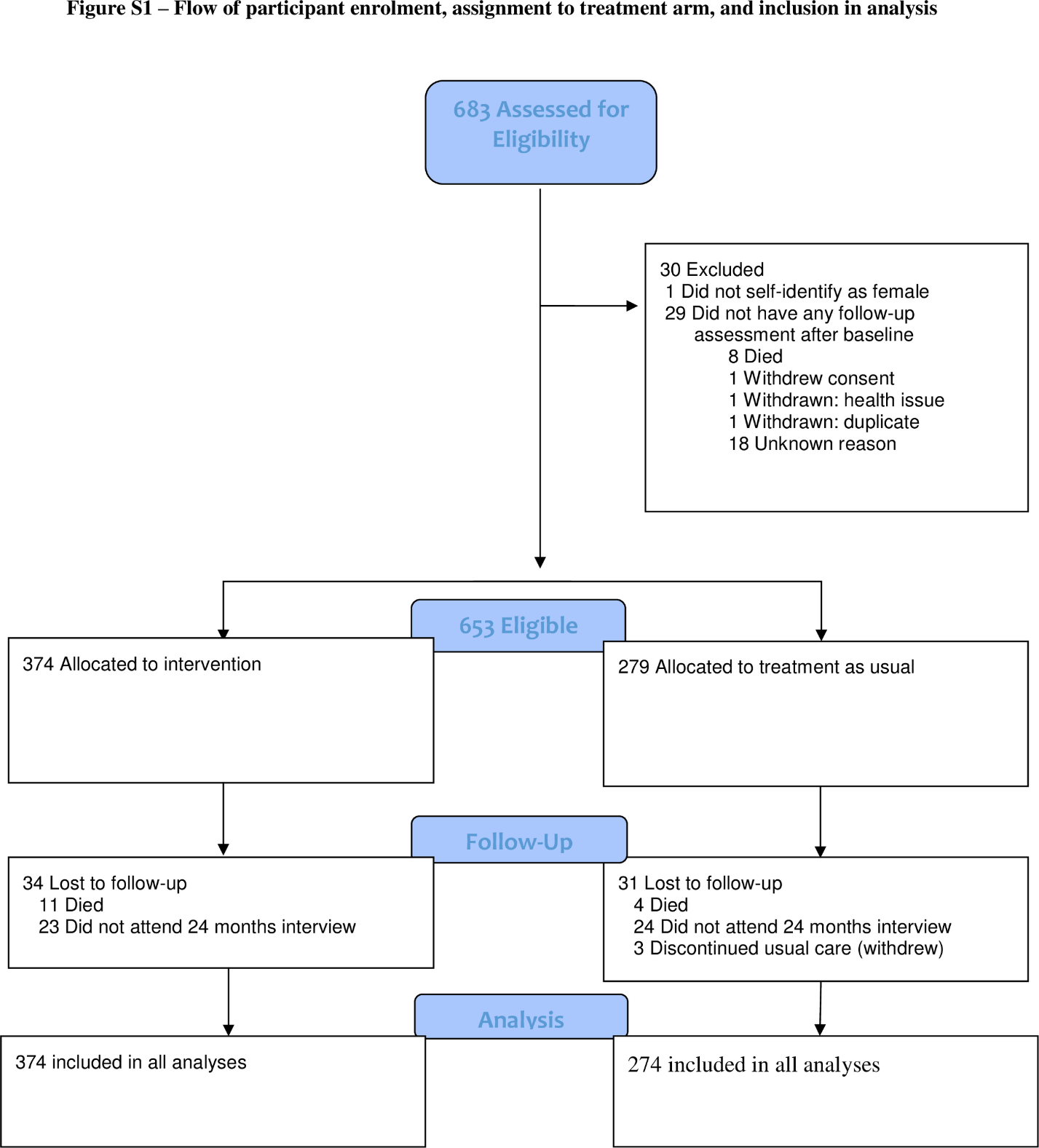


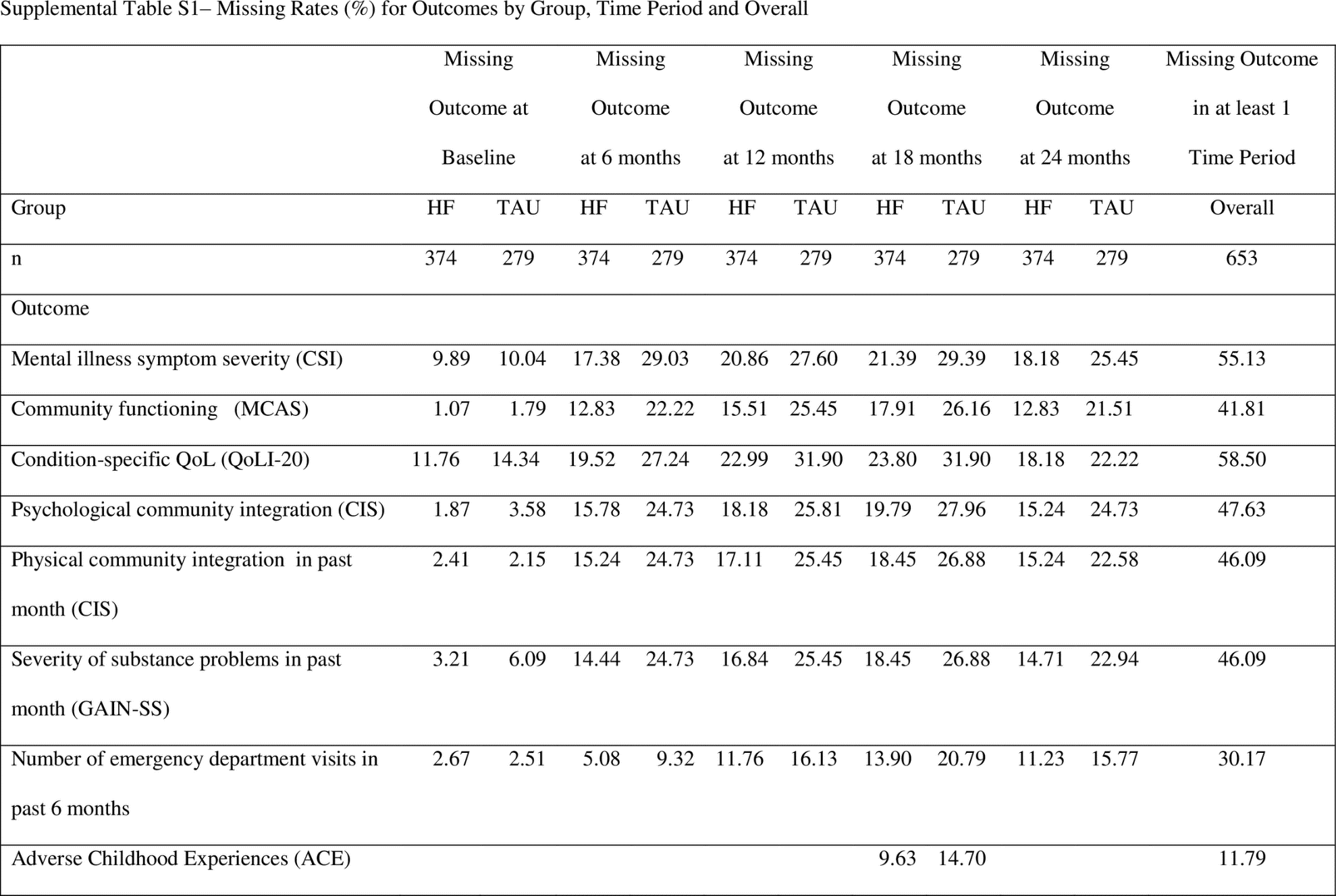


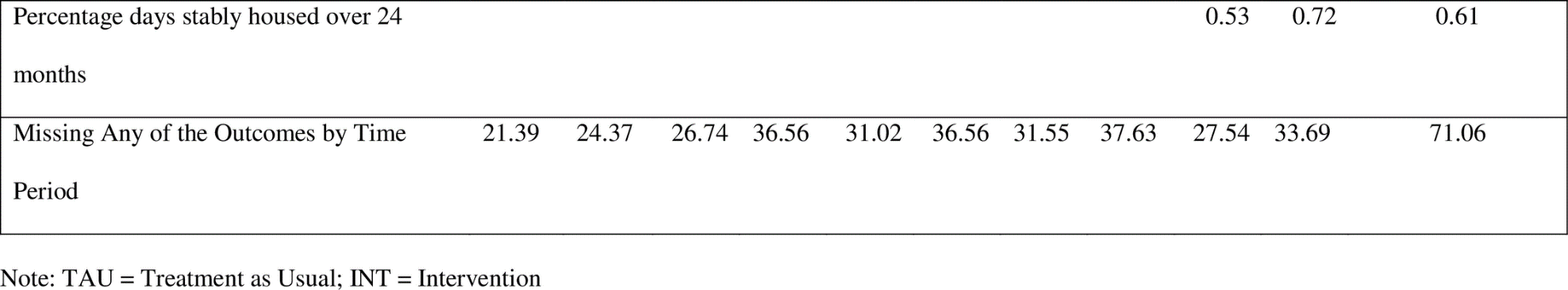


Supplemental Table S2- SAS Syntax for Regression Models

| **Variable Definitions** | |
| --- | --- |
| PCTDAYS_STABLE24M | Percentage of days stably housed over the 24 months follow-up (0-100) |
| PCTDAYS_STABLE_BASE | Percentage of days stably housed 3 months prior to baseline (0-100) |
| PSTABLE24M | Proportion of days stably housed over the 24 months follow-up (0-1) |
| PSTABLEBASE | Proportion of days stably housed 3 months prior to baseline (0-1) |
| CSI_SCORE  MCAS_TOTAL_SCORE  QOLI20_TOTAL_SCORE  CIS_PSYCH_SCORE | Continuous variables at 6, 12, 18, 24 months follow-up and baseline (add suffix 0)  Colorado Symptom Index  Multnomah Community Ability Scale  Quality of Life  Community Integration Scale Psychological |
| CIS_PHYS_SCORE  GAIN_PASTMONTH_SCORE  COUNT_ERVISIT | Count variables 6, 12, 18, 24 months at follow-up and baseline (add suffix 0)  Community Integration Scale Psychological  Global Assessment of Individual Needs Short Screener  Number ED Visits in the past 6 months |
| TIME | Follow-up Time: 6, 12, 18, 24 months |
| grp_int | Treament indicator 1=Intervention, 0=TAU |
| ARM, STUDY_ARM | Character variable INT, TAU |
| SITE1  SITE2  SITE3  SITE4 | Indicator variables for each city (reference city is SITE5) |
| CENTRE | Character variable for study city |
| act_analysis | Need level indicator 1=ACT/high; 0=ICM/moderate |
| COMP, COMPONENT | Character variable ACT, ICM |
| AGE | Continuous age at baseline 1 |
| LESSTHAN_HS | Indicator of less than high school education at baseline 1=Yes, 0=No |
| TYRSHLESS_3ORMORE | Indicator of 3 or more years of homelessness at baseline 1=Yes, 0=No |
| CHILD_SUP_1ORMORE | Indicator of supporting 1 or more children at baseline 1=Yes, 0=No |
| HIGHMOD_SUICIDALITY | Indicator of high/moderate suicidality at baseline 1=Yes, 0=No |
| ACE | Indicator of 5 or more ACEs 1=Yes, 0=No |
| **Syntax by Objective and Table** | |
| **Objective 1, Table 2**  **Continuous Outcomes: Example for CSI_SCORE**  **proc** **mixed** data=post_mi noclprint noitprint covtest method=ml;  by _imputation_;  class subjectnumber study_arm month centre component;  model CSI_SCORE= study_arm month centre study_arm*month component/solution;  repeated month/subject=subjectnumber type=un;  estimate "Month=24 vs Month=0 for INT" month **1** -**1** study_arm*month **1** -**1** **0** **0** ;  estimate "Month=24 vs Month=0 for TAU" month **1** -**1** study_arm*month **0** **0** **1** -**1**;  lsmeans study_arm*month/slice=month ;  ods output SolutionF=SOLcsi1 CovParms=COVcsi1 lsmeans=lsmeanscsi1  Estimates=ESTcsi1 Tests3=type3csi1;  **run**;  **proc** **mianalyze** parms(classvar=full)=solcsi1;  class study_arm month centre component;  modeleffects Intercept STUDY_ARM month CENTRE STUDY_ARM*month COMPONENT;  **run**;  **proc** **sort** data = lsmeansCSI1;  by study_arm month _imputation_;  **run**;  ods output parameterestimates = outlsmeans1;  **proc** **mianalyze** data=lsmeansCSI1;  by study_arm month;  modeleffects ESTIMATE;  stderr stderr;  **run**;  **proc** **sort** data = Estcsi1;  by label _imputation_;  **run**;  ods select parameterestimates;  **proc** **mianalyze** data = estcsi1 ;  by label;  modeleffects estimate;  stderr stderr;  **run**;  **Objective 1 Table 2**  **Count Outcomes: Example for GAIN (negative binomial).**  **COUNT_ERVISIT (negative binomial), CIS_PHYS_SCORE (Poisson)**  **proc** **genmod** data =post_mi;  by _imputation_;  class subjectnumber study_arm (ref='TAU') month (ref='base') centre (ref='SITE5') component (ref='ICM') /param=GLM;  model GAIN_PASTMONTH_SCORE =study_arm month centre study_arm*month component /dist=negbin type3 COVB;  repeated subject=subjectnumber/ type=un ECOVB;  estimate "RR Month=24 vs Month=0 for INT" MONTH **1** -**1** study_arm*month **1** -**1** **0** **0** /exp;  estimate "RR Month=24 vs Month=0 for TAU" MONTH **1** -**1** study_arm*month **0** **0** **1** -**1**/exp;  lsmeans study_arm*month/ilink cl;  ods output  GEEEmpPEst =gmparms  GEERCov =rcov  ParmInfo=gmpinfo  CovB=gmcovb  Estimates=EST  LSMeans=gmlsmeans  Type3 =type3gain;  **run**;  **quit**;  **proc** **mianalyze** parms(classvar=LEVEL)=gmparms ;  class study_arm month centre component;  modeleffects Intercept study_arm month centre study_arm*month component;  **run**;  **data** est3;  set est;  if MEANESTIMATE>**.**;  **run**;  **proc** **sort** data = Est3;  by label _imputation_;  **run**;  ods output parameterestimates = outrates;  **proc** **mianalyze** data = est3;  by label;  modeleffects LBETAESTIMATE;  stderr stderr;  **run**;  **data** outrates2;  set outrates;  rate_ratio=exp(estimate);  lcl_rate_ratio=exp(LCLMEAN);  ucl_rate_ratio=exp(UCLMEAN);  **run**;  **proc** **print** noobs;  var label rate_ratio lcl_rate_ratio ucl_rate_ratio probt;  **Objective 2**  **Outcome: Percentage of days stably housed over 24 months**  **proc** **mixed** data=stablehousing noitprint method=ml;  by _imputation_;  class study_arm centre component;  model PCTDAYS_STABLE24M= study_arm centre component PCTDAYS_STABLE_BASE /solution;  estimate "INT vs TAU" study_arm **1** -**1** ;  lsmeans study_arm ;  ods output SolutionF=SOLhouse lsmeans=lsmeanshouse Estimates=ESThouse ;  **run**;  **proc** **mianalyze** parms(classvar=full)=solhouse;  class study_arm centre component;  modeleffects Intercept STUDY_ARM CENTRE COMPONENT PCTDAYS_STABLE_BA;  **run**;  **proc** **sort** data = lsmeanshouse;  by study_arm _imputation_;  **run**;  ods output parameterestimates = outlsmeans1;  **proc** **mianalyze** data=lsmeanshouse;  by study_arm;  modeleffects ESTIMATE;  stderr stderr;  **run**;  ods select parameterestimates;  **proc** **mianalyze** data = esthouse ;  by label;  modeleffects estimate;  stderr stderr;  **run**;  **Outcome: Proportion of days stably housed over 24 months**  **proc** **glimmix** data= stablehousing method=quad;  by _imputation_;  model PSTABLE24M= grp_int MONCTON MONTREAL TORONTO VANCOUVER act_analysis PSTABLEBASE  / dist=binomial link=logit solution ddfm=none chisq; /* The DDFM=NONE and CHISQ options are used to produce large-sample Wald tests for the parameters and fixed effects*/  random residual_;  ods output parameterestimates=parms1;  run;  ods output parameterestimates = outparms1;  **proc** **mianalyze** parms=parms1;  modeleffects Intercept grp_int MONCTON MONTREAL TORONTO VANCOUVER act_analysis PSTABLEBASE;  **run**;  /* Exponentiating the pooled estimates of log (OR) after imputation */  **data** oddsratios1;  set outparms1;  where PARM ne 'Intercept';  odds_ratio=exp(estimate);  lcl_odds_ratio=exp(lclmean);  ucl_odds_ratio=exp(uclmean);  **run**;  **proc** **print** noobs;  var PARM estimate stderr odds_ratio lcl_odds_ratio ucl_odds_ratio probt;  format odds_ratio lcl_odds_ratio ucl_odds_ratio **5.2**;  **run**;  **Objective 2 Table 3**  **Continuous Outcomes (Example CSI_SCORE)**  **proc** **mixed** data=post_mi noclprint noitprint covtest method=ml;  by _imputation_;  class subjectnumber study_arm time centre component;  model CSI_SCORE= study_arm time centre study_arm*time component CSI0 /solution;  repeated time/subject=subjectnumber type=un;  estimate "INT vs TAU for time = 6" study_arm **1** -**1** study_arm*time **1** **0** **0** **0** -**1** **0** **0** **0** ;  estimate "INT vs TAU for time =12" study_arm **1** -**1** study_arm*time **0** **1** **0** **0** **0** -**1** **0** **0** ;  estimate "INT vs TAU for time =18" study_arm **1** -**1** study_arm*time **0** **0** **1** **0** **0** **0** -**1** **0** ;  estimate "INT vs TAU for time =24" study_arm **1** -**1** study_arm*time **0** **0** **0** **1** **0** **0** **0** -**1** ;  lsmeans study_arm*time/slice=time ;  ods output SolutionF=SOLcsi1 CovParms=COVcsi1 lsmeans=lsmeanscsi1 Estimates=ESTcsi1 Tests3=type3csi1;  **run**;  **proc** **mianalyze** parms(classvar=full)=solcsi1;  class study_arm time centre component;  modeleffects Intercept STUDY_ARM time CENTRE STUDY_ARM*time COMPONENT CSI0;  **run**;  **proc** **sort** data = lsmeansCSI1;  by study_arm time _imputation_;  **run**;  ods output parameterestimates = outlsmeans1;  **proc** **mianalyze** data=lsmeansCSI1;  by study_arm time;  modeleffects ESTIMATE;  stderr stderr;  **run**;  **proc** **sort** data = Estcsi1;  by label _imputation_;  **run**;  ods select parameterestimates;  **proc** **mianalyze** data = estcsi1 ;  by label;  modeleffects estimate;  stderr stderr;  **run**;  **Objective 2 Table 3**  **Count Outcomes (Example GAIN)**  **proc** **genmod** data =post_mi3;  by _imputation_;  class subjectnumber study_arm (ref='TAU') time (ref='24') centre (ref='SITE5') component (ref='ICM') /param=GLM;  model GAIN_PASTMONTH_SCORE =study_arm time centre study_arm*time component GAIN0/dist=negbin type3 COVB;  repeated subject=subjectnumber/ type=un ECOVB;  estimate "RR INT vs TAU for time = 6" study_arm **1** -**1** study_arm*time **1** **0** **0** **0** -**1** **0** **0** **0** /EXP;  estimate "RR INT vs TAU for time =12" study_arm **1** -**1** study_arm*time **0** **1** **0** **0** **0** -**1** **0** **0**/EXP;  estimate "RR INT vs TAU for time =18" study_arm **1** -**1** study_arm*time **0** **0** **1** **0** **0** **0** -**1** **0** /EXP;  estimate "RR INT vs TAU for time =24" study_arm **1** -**1** study_arm*time **0** **0** **0** **1** **0** **0** **0** -**1**/EXP;  lsmeans study_arm*time/ilink cl;  ods output  GEEEmpPEst =gmparms  GEERCov =rcov  ParmInfo=gmpinfo  CovB=gmcovb  Estimates=EST  LSMeans=gmlsmeans  Type3 =type3gain;  **run**;  **quit**;  **proc** **mianalyze** parms(classvar=LEVEL)=gmparms;  class study_arm time centre component;  modeleffects Intercept study_arm time centre study_arm*time component GAIN0;  **run**;  **data** est3;  set est;  if MEANESTIMATE>**.**;  **run**;  **proc** **sort** data = Est3;  by label _imputation_;  **run**;  ods output parameterestimates = outrates;  **proc** **mianalyze** data = est3;  by label;  modeleffects LBETAESTIMATE;  stderr stderr;  **data** outrates2;  set outrates;  rate_ratio=exp(estimate);  lcl_rate_ratio=exp(LCLMEAN);  ucl_rate_ratio=exp(UCLMEAN);  **run**;  **proc** **print** noobs;  var label rate_ratio lcl_rate_ratio ucl_rate_ratio probt;  **run**;  **Objective 3**  **Outcome: Percentage of days stably housed**  **proc** **mixed** data=stablehousing noitprint method=ml;  by _imputation_;  class ARM centre COMP ACE;  model PCTDAYS_STABLE24M= ARM centre COMP PCTDAYS_STABLE_BASE  AGE LESSTHAN_HS TYRSHLESS_3ORMORE CHILD_SUP_1ORMORE HIGHMOD_SUICIDALITY ACE  /solution;  ods output SolutionF=SOLhouse;  **run**;  ods output parameterestimates = outparms;  **proc** **mianalyze** parms(classvar=full)=solhouse;  class ARM centre COMP ACE;  modeleffects Intercept ARM CENTRE COMP PCTDAYS_STABLE_BA  AGE LESSTHAN_HS TYRSHLESS_3ORMORE CHILD_SUP_1ORMORE HIGHMOD_SUICIDALITY ACE;  **run**;  **proc** **print** noobs data=outparms;where estimate ne **0** and PARM ne 'Intercept';  var PARM CENTRE COMP ESTIMATE /*STDERR*/ LCLMEAN UCLMEAN PROBT;  format ESTIMATE LCLMEAN UCLMEAN **6.2**;  **run**;    **Outcome: Proportion of days stably housed**  **proc** **glimmix** data= stablehousing method=quad;  by _imputation_;  model PSTABLE24M= grp_int MONCTON MONTREAL TORONTO VANCOUVER act_analysis PSTABLEBASE AGE LESSTHAN_HS TYRSHLESS_3ORMORE CHILD_SUP_1ORMORE HIGHMOD_SUICIDALITY ACE  / dist=binomial link=logit solution ddfm=none chisq;  random _residual_;  ods output parameterestimates=parms2 ;  run;  ods output parameterestimates = outparms2;  **proc** **mianalyze** parms=parms2;  modeleffects Intercept grp_int MONCTON MONTREAL TORONTO VANCOUVER act_analysis PSTABLEBASE AGE LESSTHAN_HS TYRSHLESS_3ORMORE CHILD_SUP_1ORMORE HIGHMOD_SUICIDALITY ACE;  **run**;  **data** oddsratios2;  set outparms2;  where PARM ne 'Intercept';  odds_ratio=exp(estimate);  lcl_odds_ratio=exp(lclmean);  ucl_odds_ratio=exp(uclmean);  **run**;  **proc** **print** noobs;  var PARM estimate stderr odds_ratio lcl_odds_ratio ucl_odds_ratio probt;  format odds_ratio lcl_odds_ratio ucl_odds_ratio **5.2**;  **run**;    **Objective 3 Table 4**  **Continuous Outcomes (Example CSI_SCORE)**  **proc** **mixed** data=post_mi noclprint noitprint covtest method=ml;  by _imputation_;  class subjectnumber arm time centre comp ;  model CSI_SCORE= arm time centre arm*time comp CSI0  AGE LESSTHAN_HS TYRSHLESS_3ORMORE CHILD_SUP_1ORMORE HIGHMOD_SUICIDALITY ACE/solution;  repeated time/subject=subjectnumber type=un;  ods output SolutionF=SOLcsi1 CovParms=COVcsi1 Tests3=type3csi1 ;  **run**;  ods output parameterestimates = outparms1;  **proc** **mianalyze** parms(classvar=full)=solcsi1;  class arm time centre comp ;  modeleffects Intercept arm time CENTRE arm*time comp CSI0  AGE LESSTHAN_HS TYRSHLESS_3ORMORE CHILD_SUP_1ORMORE HIGHMOD_SUICIDALITY ACE;  **run**;  **proc** **print** noobs data=outparms1; where estimate ne **0** and PARM ne 'Intercept';  var PARM ARM TIME CENTRE COMP ESTIMATE LCLMEAN UCLMEAN PROBT;  format ESTIMATE LCLMEAN UCLMEAN **5.2**;  ods rtf close;  **Objective 3 Table 4**  **Count Outcomes (Example GAIN)**  **proc** **genmod** data =post_mi;  by _imputation_;  class subjectnumber ARM (ref='TAU') time (ref='24') centre (ref='Winnipeg') COMP (ref='ICM') /param=GLM;  model GAIN_PASTMONTH_SCORE =ARM time centre ARM*time COMP GAIN0  AGE LESSTHAN_HS TYRSHLESS_3ORMORE CHILD_SUP_1ORMORE HIGHMOD_SUICIDALITY ACE  /dist=negbin type3 COVB;  repeated subject=subjectnumber/ type=un ECOVB;  ods output  GEEEmpPEst =gmparms  GEERCov =rcov  ParmInfo=gmpinfo  CovB=gmcovb  Type3 =type3gain;  **run**;  ods output parameterestimates = outparms6;  **proc** **mianalyze** parms(classvar=LEVEL)=gmparms;  class ARM time centre COMP ;  modeleffects Intercept ARM time centre ARM*time COMP GAIN0  AGE LESSTHAN_HS TYRSHLESS_3ORMORE CHILD_SUP_1ORMORE HIGHMOD_SUICIDALITY ACE  **run**;  **proc** **print** noobs data=outparms6; where estimate ne **0** and PARM ne 'Intercept';  var PARM ARM TIME CENTRE COMP ESTIMATE LCLMEAN UCLMEAN PROBT;  format ESTIMATE LCLMEAN UCLMEAN **5.2**;  **run**;  **data** rateratios;  set outparms6;  where PARM ne 'Intercept';  rate_ratio=exp(estimate);  lcl_rate_ratio=exp(LCLMEAN);  ucl_rate_ratio=exp(UCLMEAN);  **run**;  **proc** **print** noobs;  var PARM ARM TIME CENTRE COMP rate_ratio lcl_rate_ratio ucl_rate_ratio PROBT;  format rate_ratio lcl_rate_ratio ucl_rate_ratio **5.2**;  **run**; | |
