# Supplementary material for: Women experiencing homelessness and mental illness in a Housing First multi-site trial: Looking beyond housing to social outcomes and well-being
Source: PLoS One. 2023 Feb 10;18(2):e0277074. doi: 10.1371/journal.pone.0277074 (PMC9916643; doi:10.1371/journal.pone.0277074)
Supplement: Attachment — Submitted filename: PONE-D-21-13748_R1_reviewer.pdf [file pone.0277074.s003.pdf]

PLOS ONE

Women experiencing homelessness and mental illness in a Housing First multi-site trial: Looking beyond housing to social outcomes and well-being.

--Manuscript Draft--

**Manuscript Number:**	PONE-D-21-13748R1
**Article Type:**	Research Article
**Full Title:**	Women experiencing homelessness and mental illness in a Housing First multi-site trial: Looking beyond housing to social outcomes and well-being.
**Short Title:**	Women's experiences with Housing First
**Corresponding Author:**	Patricia O'Campo<br>St Michael's Hospital<br>Toronto, Ontario CANADA
**Keywords:**	women, homelessness, Housing First, randomized trial, mental health, suicidality, quality of life
**Abstract:**	Objective: There is scant research on the effectiveness of permanent supportive housing for homeless women with mental illness. This study examines the effectiveness of Housing First with an unprecedentedly large sample of homeless women from five Canadian cities, and explore baseline risk factors that predict social, health and well-being outcomes over a 24 month-period.<br>Methods: The At Home/Chez Soi multi-site randomized controlled Housing First trial recruited over 600 women between October 2009 and July 2011. This is a post-hoc subgroup analysis of self-identified women with at least one follow-up interview who were randomized to Housing First (HF) (n =374) or treatment-as-usual (TAU) (n =279) and had at least one follow-up interview. Linear mixed models and generalized estimating equations were used after multiple imputation was applied to address missing data.<br>Results: At the end of follow-up, the mean percentage of days spent stably housed was higher for women in the intervention 74.8% (95%CI=71.7%–77.8%) compared with women in the treatment-as-usual group, 37.9% (95%CI=34.4%–41.3%), p <0.001. With few exceptions, social and mental health outcomes were similar for both groups at 6-, 12-, 18- and 24-months post-enrollment. Suicidality was a consistent predictor of increased mental health symptoms (beta=2.85, 95% CI 1.59-4.11, p<0.001), decreased quality of life (beta=-3.99, 95% CI -6.49 to -1.49, p<0.001), decreased community functioning (beta= -1.16, 95% CI -2.10 to -0.22, p=0.015) and more emergency department visits (rate ratio = 1.44, 95% CI 1.10-1.87, p<0.001) over the study period. Lower education was a predictor of lower community functioning (beta=-1.32, 95% CI -2.27 to -0.37, p=0.006) and higher substance use problems (rate ratio=1.27, 95% CI 1.06-1.52, p=0.009) during the study.<br>Conclusions: Housing First interventions ensured that women experiencing homelessness are quickly and consistently stably housed. However, they did not differentially impact health and social measures compared to treatment as usual. Ensuring positive health and social outcomes may require greater supports at enrolment for subgroups such as those with low educational attainment, and additional attention to severity of baseline mental health challenges, such as suicidality.
**Order of Authors:**	Patricia O'Campo
	Rosane Nisenbaum
	Anne G. Crocker
	Tonia L. Nicholls
	Faith Eiboff
	Carol Adair
**Response to Reviewers:**	Here we respond to the specific comments raised by the Editor. These are also included in our “Response to Reviewer” document along with supplemental figures/tables.

Powered by Editorial Manager® and ProduXion Manager® from Aries Systems Corporation

Comment: Regarding missingness, it's important to address the missing data mechanisms in more depth than currently, and also whether these mechanisms result in data that are missing at random, missing completely at random, or even not missing at random. Given the diverse reasons for missingness, it is possible that more than one type of missingness is present in the data.
Answer: Missing data mechanisms have been widely presented and discussed in the literature. Although the Missing Completely at Random mechanism simplifies analyses, it is quite difficult to find real life applications that satisfy its assumption [VanBuuren2012], and therefore we did not consider it in our data.
We did however consider the Missing at Random (MAR) Mechanism, where the probability of missingness depends only on observed data but not on the data that are missing. That is, after controlling for or stratifying by observed variables, missingness is random. We followed recommendation from Van Buuren 2012 and Barnard 1999 to include in our imputation models a high the number of predictors of the incomplete variables in order to make the MAR assumptions more plausible.
Van Buuren, S. (2012), Flexible Imputation of Missing Data. Chapman & Hall/CRC, Boca Raton, FL.

Barnard J, Meng XL Applications of multiple imputation in medical studies: from AIDS to NHANES. Statistical Methods in Medical Research. 8:17-36, 1999

Comment: 70% of data had at least one missing value. With such a large percent like 70%, a reader might think that is in the entire dataset. It seems from context and supplementary table 1 that this was all variables that are used in this paper's analysis, so please say that more clearly. Also given that 70% of data used in the study had at least one missing value, please cite research about the implications of MI in data with such a large percent missing.
Answer: We followed recommendations from White et al, 2010 and Horton et al. 2007 who suggest that depending on the number and type of variables, and amount of missing data, 50 to 100 imputations yield better estimates. However, a rule of thumb was provided by White et al. 2010 that the number of imputations should be at least 100 x (percent of incomplete cases) and we applied this recommendation to our data analysis approach.
Horton, N. & Kleinman, K.P. (2007). Much ado about nothing: A comparison of missing data methods and software to fit incomplete data regression models. The American Statistician 61(1): 79-90. Software appendix: http://www.math.smith.edu/muchado-appendix.pdf

White, I.R., Royston, P., and Wood, A.M. (2010). Multiple imputation using chained equations: Issues and guidance for practice. Statistics in Medicine 30:377-399. (Includes Stata codes)

Comment: One reviewer asked for sensitivity analysis for imputed versus non-imputed. Diagnostics of MI method may be appropriate.
Answer: As requested, we provide a sample of multiple imputation diagnostic plots as suggested by Van Buuren, 2012 at the end of this document.
We also added the following sentence to the methods: Comparisons between observed and imputed data via diagnostic plots were used to assess the quality and plausibility of the imputations.
Van Buuren, S. (2012), Flexible Imputation of Missing Data. Chapman & Hall/CRC, Boca Raton, FL.

Comment: With regard to sample size calculation, an alternative to a standard power calculation would be Gelman and Carlin type-M and type-S errors, which is less centered on p-values.
Answer:Because ours was a post-hoc subgroup exploratory analysis of the main study, the sample size calculation which was predetermined for the main hypotheses for the main study involving the full sample, does not apply here. As such, for the analyses we present here, we would like to emphasize that our study was hypothesis generating and therefore power calculations do not apply. The original sample sizes were calculated for the original study, please refer to protocol in Goering et al.
Goering PN, Streiner DL, Adair C, Aubry T, Barker J, Distasio J, Hwang SW, Komaroff J, Latimer E, Somers J, Zabkiewicz DM. The At Home/Chez Soi trial protocol: a

Powered by Editorial Manager® and ProduXion Manager® from Aries Systems Corporation

pragmatic, multi-site, randomised controlled trial of a Housing First intervention for homeless individuals with mental illness in five Canadian cities. BMJ Open. 2011 Nov 14;1(2):e000323

Comment: The effects of the experiment have been displayed as p-values. An effect size measure such as Hedge's g or Cohen's d in addition to the differences that are in the tables 2-4. These effect size measures may give a clearer picture of the true results of the experiment.
Answer: We are familiar with Cohen's d and the Hedge's g correction as alternate ways of illustrating differences between treatment and control groups. We did not use Cohen's d in any of our dozens of publications that emerged from this randomized trial as in our experience it is not as common in health or medical studies as it would be in psychology. In fact, a quick review of evaluations or randomized trials published in Plos One over a one-year period (back to Dec 2020, see citations below) demonstrated that these studies report findings in a similar way to which we presented our findings. Evaluations presented mean values of outcomes for treatment and controls groups along with 95% confidence intervals and p-values. Some studies add in the t-statistics. As such, we feel that adding Cohen's d values were not necessary.
Hajek P, Przulj D, Pesola F, McRobbie H, Peerbux S, Phillips-Waller A, et al. (2021) A randomised controlled trial of the 5:2 diet. PLoS ONE 16(11): e0258853.
Ngugi P, Babic A, Were MC (2021) A multivariate statistical evaluation of actual use of electronic health record systems implementations in Kenya. PLoS ONE 16(9): e0256799.
Müller SA, Diallo AOK, Rocha C, Wood R, Landsmann L, Camara BS, et al. (2021) Mixed methods study evaluating the implementation of the WHO hand hygiene strategy focusing on alcohol based handrub and training among health care workers in Faranah, Guinea. PLoS ONE 16(8): e0256760.
Andersen TO, Dissing AS, Varga TV, Rod NH (2021) The SmartSleep Experiment: Evaluation of changes in night-time smartphone behavior following a mass media citizen science campaign. PLoS ONE 16(7): e0253783.
Greene MC, Likindikoki S, Rees S, Bonz A, Kaysen D, Misinzo L, et al. (2021) Evaluation of an integrated intervention to reduce psychological distress and intimate partner violence in refugees: Results from the Nguvu cluster randomized feasibility trial. PLoS ONE 16(6): e0252982.
Heimgartner N, Meier S, Grolimund S, Ponti S, Arpagaus S, Kappeler F, et al. (2021) Randomized controlled evaluation of the psychophysiological effects of social support stress management in healthy women. PLoS ONE 16(6): e0252568.
Dardas A, Williams A, Wang L (2021) Evaluating changes in workplace culture: Effectiveness of a caregiver-friendly workplace program in a public post-secondary educational institution. PLoS ONE 16(5): e0250978.
O'Donnell A, Wilson L, Bosch JA, Borrows R (2020) Life satisfaction and happiness in patients shielding from the COVID-19 global pandemic: A randomised controlled study of the 'mood as information' theory. PLoS ONE 15(12): e0243278.

Comment: P-values have been displayed to 3 digits in all tables. That's too many digits and gives false precision. Just one significant digit is fine. For instance, in table 2 the p-values would be 0.001 (as is currently there), 0.5, 0.2, 0.2, 0.9, 0.4, 0.7, 0.9.
Answer:We respectfully disagree with this suggestion to report p-values using 1 significant digit. We not only feel this is imprecise but this practice is not aligned with what we discovered in our review of studies published in Plos One. Studies reviewed (see item above) reported p-values using 3 digits or sometimes more significant digits. Therefore, we have not reduced the number of significant digits we report for our p-values in our tables and text.

Comment: Figure 1 is really nice display of results, but the treated and control groups should be shifted slightly so the confidence intervals are clearer and don't overlap—e.g., intervention at 0.5 months below each time point and control at 0.5 months above each time point. Many of these continuous variable outcomes in figure 1 probably have complex distributions, and it may be helpful to show the distributions of these variables for treatment versus control, such as using a violin plot or similar data display, rather than the point and CI shown in figure 1.
Answer:Thank you for your appreciation of our figures. The figures are intended to augment the study findings in the tables as Table 3 presents results of multivariate analyses. The Figures with the unadjusted results are intended to be a visual

Powered by Editorial Manager® and ProduXion Manager® from Aries Systems Corporation

supplement to Table 3 as the differences or lack thereof are easier to grasp quickly in the figures. While CIs are included in the Figures to help identify whether the values at any one time point differ between treatment and TAU, the data in the Tables reporting multivariate regression findings should be the primary source of information about differences and significance of those differences. To change the scales on the figures to further separate the trend lines for the treatment and TAU would create larger figures that would take up too much space and is not necessary given that the multivariate findings are presented in Table 3.
Comment: The tables are somewhat hard to read with many lines, and they may benefit from using data displays such as dumbbell plots for comparisons of the treatment vs control.
Answer:We have added formats to the table to increase readability. In particular, we have shaded every other row in Tables 1, 3 and 4. We have added lines between each outcome to more clearly delineate the findings for each outcome and co-variate. We believe the format changes have considerably increased the readability of the Tables. Moreover the figures we provided as supplementary information about Table 3 is a visual aid to rapidly understanding the findings from the analysis of these trial data.

Comment: The mixed models should report evaluation of assumptions of the models, such as linear regression, Poisson regression.
Answer:To respond to this suggestion we have added the following text in the Statistical analysis section: Normality of residuals was graphically checked for linear mixed models. The decision to fit a negative binomial rather than a Poisson model was based on whether over-dispersion (, ie, if the variance was much larger than the mean) was present in the count outcome data.

Comment: Table 2 shows percent of the time housed over all visits. However, the other measures in table 2 are a comparison of baseline to 24 months adjusted for city and need level, which leaves out the middle 3 follow-up visits. It seems like these observations in table 2 could be more completely displayed as an adjusted version of Figure 1 that includes all time points adjusted for city and need level.
Answer:There were several reasons why our study measured housing success as cumulative stability at 24 months and we now state this in the methods. First, given the Housing First Theory of Change and Program Manual, stable housing is established within the first 1-3 months of enrolment for those in the treatment arm and housing status rarely changes after that point in time. Thus, we wanted to align with the program's theory of change. Second, to measure housing status at different time points over the 24 months means that we would have seen very little change for those in the treatment arm and a single cumulative measure made more sense for this outcome. This measure captures overall stability and instability in that a small proportion of the treatment group was not stably housed throughout and this single measure captures that information. Third, this single measure captures the overall housing stability or instability across the two groups over a long period of time and for this outcome, knowing the housing status at different time points in the study is less relevant to the overall goals of the program. In sum, we chose the best measure for the outcome of housing even if it did not match how we approached other outcomes such as quality of life which is expected to demonstrate incremental changes over the 24-month follow-up period.

Comment: Table 4 shows mixed model effects that leaves out the treatment effect and treatment x time, among other variables. It seems that this table should at least include treatment effect coefficients. Omitting the main effect of treatment doesn't avoid table 2 fallacy. The authors should address whether table 2 fallacy may apply here.
Answer:Table 4 presents the findings for the third objective of the paper (see bottom of page 7 of the manuscript for the objective) intended to "explore baseline demographic, social, health measures, and ACEs as predictors of key study outcomes during the study period." This objective is not concerned with differences between treatment and TAU. In fact, we include that objective because few differences between treatment and TAU were reported and we can, therefore, take advantage of this large sample of homeless women to learn more about predictors of our key outcomes. Consequently, our analysis for this objective does not account for treatment group membership by design. We do not address the issue of type 2 fallacy for this objective as it does not apply. However, we have made our intent clearer in the statistical analyses, see top of page 11 and in the results on page 13

	Comment: 3. Please provide additional details regarding participant consent. In the ethics statement in the Methods and online submission information, please ensure that you have specified what type you obtained (for instance, written or verbal, and if verbal, how it was documented and witnessed). If your study included minors, state whether you obtained consent from parents or guardians. If the need for consent was waived by the ethics committee, please include this information.<br>Answer:We have expanded the description of the consent process and Ethics Board approval process on page 8 of the revised manuscript.<br><br>Comment: 4. We note that the grant information you provided in the 'Funding Information' and 'Financial Disclosure' sections do not match.<br>Answer:We tried upon our initial submission to create a new entry online for our funding agency, the Mental Health Commission of Canada. However, when we try to add this new funding agency to your database the system freezes and cannot seem to complete the task. Therefore we did not do this task but are willing if we get assistance to do so. We did also try to email the generic help email but received an unhelpful response that we should enter the information into the field with no guidance on how to overcome our problem.<br><br>Comment: When you resubmit, please ensure that you provide the correct grant numbers for the awards you received for your study in the 'Funding Information' section.<br>Answer:There was no grant number for this award.
**Additional Information:**	
**Question**	**Response**
**Financial Disclosure**<br><br>Enter a financial disclosure statement that describes the sources of funding for the work included in this submission. Review the submission guidelines for detailed requirements. View published research articles from *PLOS ONE* for specific examples.<br><br>This statement is required for submission and **will appear in the published article** if the submission is accepted. Please make sure it is accurate.	This research has been made possible through a financial contribution from Health Canada (https://www.canada.ca/en/health-canada.html). The views expressed herein solely represent the authors. The Mental Health Commission of Canada (https://www.mentalhealthcommission.ca/English) oversaw the conduct of the study and provided training and technical support to the service teams and research staff throughout the project. However, the funder had no role in the analysis and interpretation of the data, preparation, review or approval of the manuscript.

**Unfunded studies**<br>Enter: *The author(s) received no specific funding for this work.*<br><br>**Funded studies**<br>Enter a statement with the following details:<br>• Initials of the authors who received each award<br>• Grant numbers awarded to each author<br>• The full name of each funder<br>• URL of each funder website<br>• Did the sponsors or funders play any role in the study design, data collection and analysis, decision to publish, or preparation of the manuscript?<br>• **NO** - Include this sentence at the end of your statement: *The funders had no role in study design, data collection and analysis, decision to publish, or preparation of the manuscript.*<br>• **YES** - Specify the role(s) played.<br><br>* typeset	
**Competing Interests**<br><br>Use the instructions below to enter a competing interest statement for this submission. On behalf of all authors, disclose any competing interests that could be perceived to bias this work—acknowledging all financial support and any other relevant financial or non-financial competing interests.<br><br>This statement is required for submission and **will appear in the published article** if the submission is accepted. Please make sure it is accurate and that any funding sources listed in your Funding Information later in the submission form are also declared in your Financial Disclosure statement.<br><br>View published research articles from *PLOS ONE* for specific examples.	The authors have declared that no competing interests exist.

**NO authors have competing interests**<br><br>Enter: *The authors have declared that no competing interests exist.*<br><br>**Authors with competing interests**<br><br>Enter competing interest details beginning with this statement:<br><br>*I have read the journal's policy and the authors of this manuscript have the following competing interests: [insert competing interests here]*<br><br>* typeset	
**Ethics Statement**<br><br>Enter an ethics statement for this submission. This statement is required if the study involved:<br><br>• Human participants<br>• Human specimens or tissue<br>• Vertebrate animals or cephalopods<br>• Vertebrate embryos or tissues<br>• Field research<br><br>Write "N/A" if the submission does not require an ethics statement.<br><br>General guidance is provided below. Consult the submission guidelines for detailed instructions. **Make sure that all information entered here is included in the Methods section of the manuscript.**	Information about the study, such as the randomization process and a description of the intervention, was provided to all participants study prior to enrolment. A process was created to ensure that participants understood the complexities of the trial and follow-up activities. All participants provided informed consent prior to enrolment. All enrolment and consent documents were harmonized across sites, however, each site had a separate approvals from relevant university/site Research Ethics Boards.

Powered by Editorial Manager® and ProduXion Manager® from Aries Systems Corporation

**Format for specific study types**<br><br>**Human Subject Research (involving human participants and/or tissue)**<br>• Give the name of the institutional review board or ethics committee that approved the study<br>• Include the approval number and/or a statement indicating approval of this research<br>• Indicate the form of consent obtained (written/oral) or the reason that consent was not obtained (e.g. the data were analyzed anonymously)<br><br>**Animal Research (involving vertebrate animals, embryos or tissues)**<br>• Provide the name of the Institutional Animal Care and Use Committee (IACUC) or other relevant ethics board that reviewed the study protocol, and indicate whether they approved this research or granted a formal waiver of ethical approval<br>• Include an approval number if one was obtained<br>• If the study involved *non-human primates*, add *additional details* about animal welfare and steps taken to ameliorate suffering<br>• If anesthesia, euthanasia, or any kind of animal sacrifice is part of the study, include briefly which substances and/or methods were applied<br><br>**Field Research**<br><br>Include the following details if this study involves the collection of plant, animal, or other materials from a natural setting:<br>• Field permit number<br>• Name of the institution or relevant body that granted permission	
**Data Availability**<br><br>Authors are required to make all data underlying the findings described fully available, without restriction, and from the time of publication. PLOS allows rare exceptions to address legal and ethical concerns. See the PLOS Data Policy and FAQ for detailed information.	Yes - all data are fully available without restriction

A Data Availability Statement describing where the data can be found is required at submission. Your answers to this question constitute the Data Availability Statement and **will be published in the article**, if accepted.<br><br>**Important:** Stating 'data available on request from the author' is not sufficient. If your data are only available upon request, select 'No' for the first question and explain your exceptional situation in the text box.<br><br>Do the authors confirm that all data underlying the findings described in their manuscript are fully available without restriction?	
**Describe where the data may be found in full sentences. If you are copying our sample text, replace any instances of XXX with the appropriate details.**<br><br>• If the data are **held or will be held in a public repository**, include URLs, accession numbers or DOIs. If this information will only be available after acceptance, indicate this by ticking the box below. For example: *All XXX files are available from the XXX database (accession number(s) XXX, XXX.).*<br>• If the data are all contained **within the manuscript and/or Supporting Information files**, enter the following: *All relevant data are within the manuscript and its Supporting Information files.*<br>• If neither of these applies but you are able to provide **details of access elsewhere**, with or without limitations, please do so. For example:<br><br>*Data cannot be shared publicly because of [XXX]. Data are available from the XXX Institutional Data Access / Ethics Committee (contact via XXX) for researchers who meet the criteria for access to confidential data.*<br><br>*The data underlying the results presented in the study are available from (include the name of the third party*	All relevant data are within the manuscript.

and contact information or URL).<br>• This text is appropriate if the data are owned by a third party and authors do not have permission to share the data.<br><br>* typeset	
Additional data availability information:	

Women experiencing homelessness and mental illness in a Housing First multi-site trial. Looking beyond housing to social outcomes and well-being.

Patricia O'Campo[1,*], Rosane Nisenbaum[1,2,¶], Anne G. Crocker[3,¶], Tonia L. Nicholls[4,¶], Faith Eiboff[5,¶], Carol Adair[6,¶]

[1]Centre for Urban Health Solutions, St Michael's Hospital, Dalla Lana School of Public Health, University of Toronto, Toronto, Ontario, Canada

[2]Applied Health Research Centre, Li Ka Shing Knowledge Institute, Toronto, Ontario, Canada

[3]Research & Academics, Institut national de psychiatrie légale Philippe-Pinel, Department of Psychiatry & Addictions, and School of Criminology, Université de Montréal, Montreal, Quebec, Canada

[4]Department of Psychiatry, University of British Columbia and BC Mental Health and Substance Use Services, Vancouver, British Columbia, Canada

[5]Interdisciplinary Studies, School of Population and Public Health, University of British Columbia, Vancouver, British Columbia, Canada

[6]Department of Psychiatry and Department of Community Health Sciences, Cumming School of Medicine, University of Calgary, Calgary, Alberta, Canada

[*]Corresponding author

E-mail: patricia.ocampo@unityhealth.to (PO)

[¶]These authors contributed equally to this work.

Manuscript Click here to access/download;Manuscript;Manuscript.docx

Women experiencing homelessness and mental illness in a Housing First multi-site trial: Looking beyond housing to social outcomes and well-being.

O'Campo P., Nisenbaum R., Crocker A. G., Nicholls T., Eiboff F., Adair C.E.

O'Campo, P., Centre for Urban Health Solutions, St Michael's Hospital. Dalla Lana School of Public Health, University of Toronto

Nisenbaum, R., Centre for Urban Health Solutions, Applied Health Research Centre, Li Ka Shing Knowledge Institute, St Michael's Hospital. Dalla Lana School of Public Health, University of Toronto

Crocker A. G., Director, Research & Academics, Institut national de psychiatrie légale Philippe-Pinel & Full professor, Department of Psychiatry & Addictions, and School of Criminology, Université de Montréal

Nicholls, T. L., Department of Psychiatry, University of British Columbia and BC Mental Health and Substance Use Services

Eiboff, F., Interdisciplinary Studies, School of Population and Public Health, University of British Columbia

Adair, C.E., Department of Psychiatry and Department of Community Health Sciences, Cumming School of Medicine, University of Calgary

Abstract

Objective: There is scant research on the effectiveness of permanent supportive housing for homeless women with mental illness. This study examines the effectiveness of Housing First with an unprecedentedly large sample of homeless women from five Canadian cities, and explore baseline risk factors that predict social, health and well-being outcomes over a 24 month-period.

Methods: The At Home/Chez Soi multi-site randomized controlled Housing First trial recruited over 600 women between October 2009 and July 2011. This is a post-hoc subgroup analysis of self-identified women with at least one follow-up interview who were randomized to Housing First (HF) (n=374) or treatment-as-usual (TAU) (n=279) and had at least one follow-up interview. Linear mixed models and generalized estimating equations were used after multiple imputation was applied to address missing data.

Results: At the end of follow-up, the mean percentage of days spent stably housed was higher for women in the intervention 74.8% (95%CI=71.7%–77.8%) compared with women in the treatment-as-usual group, 37.9% (95%CI=34.4%–41.3%), p<0.001. With few exceptions, social and mental health outcomes were similar for both groups at 6-, 12-, 18- and 24-months post-enrollment. Suicidality was a consistent predictor of increased mental health symptoms (beta=2.85, 95% CI 1.59-4.11, p<0.001), decreased quality of life (beta=-3.99, 95% CI -6.49 to -1.49, p<0.001), decreased community functioning (beta= -1.16, 95% CI -2.10 to -0.22, p=0.015) and more emergency department visits (rate ratio = 1.44, 95% CI 1.10-1.87, p<0.001) over the study period. Lower education was a predictor of lower community functioning (beta=-1.32, 95% CI -2.27 to -0.37, p=0.006) and higher substance use problems (rate ratio=1.27, 95% CI 1.06-1.52, p=0.009) during the study.

Conclusions: Housing First interventions ensured that women experiencing homelessness are quickly and consistently stably housed. However, they did not differentially impact health and social measures compared to treatment as usual. Ensuring positive health and social outcomes may require greater supports at enrolment for subgroups such as those with low educational attainment, and additional attention to severity of baseline mental health challenges, such as suicidality.

Key Words: women, homelessness, Housing First, randomized trial, mental health, suicidality, quality of life

Funding: The At Home/Chez Soi demonstration project was funded by Health Canada through the Mental Health Commission of Canada. Trial Registration: International Standard Randomized Control Trial Number Register Identifier: ISRCTN42520374 http://www.controlled-trials.com/ISRCTN42520374. PO was supported by a research chair in Intersectoral Solutions to Urban Health Problems.TN held a CIHR New Investigator award.

Introduction

Homelessness in Canada is primarily experienced by men, yet data show that over one-third of the homeless people in this country are women and close to 90% of the single parents who are homeless are women[1-3]. Mental health and substance use disproportionately affect women experiencing homelessness. Between 20-50% experience major depressive disorder and major depressive episodes as demonstrated in systematic reviews [4], between 30-40% experience post-traumatic stress disorder [5, 6], as many as one in five report moderate-to-high levels of suicidality [6], and a large proportion experience exceptional vulnerability to an array of health and social risks [6-11], including premature mortality [12]. A recent meta-analysis of observational studies in high-income countries reports that three-quarter of homeless individuals have a mental illness diagnosis with no differences by gender[13].

Women who are homeless have different health and social vulnerabilities compared to homeless men. They have higher rates of distress including suicidal ideation [14], greater experiences of trauma prior to and during episodes of homelessness including more adverse childhood events (ACEs) such as physical and sexual abuse and trauma-related disorders [6, 15-22], few options for effective woman-centred services and treatments for these traumatic events and other ailments, all of which predispose them to homelessness [14, 23-27]. They are also substantially more likely than homeless men to engage in survival sex work and are thus more vulnerable to exploitation, violence, infectious disease, gynecological problems and unwanted pregnancies [6, 28]. Homeless women with children express considerable traumatic distress because of frequent child apprehensions [29]. Because women who are homeless often seek shelter in informal settings such as couch surfing or sleeping rough, they are less visible and

have less access to services [14, 30, 31] and have received less attention in research and policy and practice.

Program planners and policy makers operate in a context of too little gender-specific data on programs and interventions and weak studies addressing the experiences of women [3, 4],[32]. As such, there have been calls for more research that moves away from gender-neutral approaches (i.e., examining men's and women's experiences with the same lens, as if there are no gender differences) [14, 32-34] towards more gender-sensitive frameworks, including women-only analyses that capture the variability around the unique challenges women who are homeless face and accelerating evidence-informed practice with this population[3, 30, 35, 36]. For interventions, there is a need for studies with large enough samples to examine whether and how programs work for women[4, 37-42].

Currently, with some exceptions [43], what we do know about housing interventions for women experiencing homelessness comes from small subsamples in longitudinal observational studies, [35, 44] randomized trial pilot, [45] or qualitative evaluations [33, 46]. A large multi-site supportive housing demonstration project for chronically homeless adults in 11 US communities, with 173 women as part of the sample, reported substantial improvements in housing stability but no gender differences in this and other service utilization or clinical outcomes over two years of follow-up [47]. In an observational study of an integrated service system initiative in 18 sites across the US, women (N=2727) demonstrated greater improvements in family relationships and social support compared to men (N=4502) over 18-months of follow-up[43].

Housing First, a supportive housing approach with demonstrated success in transitioning individuals experiencing chronic homelessness and mental illness into permanent housing, has negligible evidence on outcomes for women[48-51]. The Housing First model is based on harm

reduction principles of choice and self-determination, and provides immediate access to scattered-site housing through the provision of rent supplements and flexible recovery-oriented supports[50, 52]. Participation in Housing First is not contingent on participation in services or requirements of 'housing readiness' (e.g., sobriety or medication compliance) [50]. The At Home/Chez Soi randomized trial conducted in five cities across Canada provides an unprecedented opportunity to examine the impact of a housing first initiative among women for several reasons. First, as the largest randomized trial of a Housing First initiative and includes an unprecedented sample of several hundred women. This large sample size facilitates a woman only analysis to focus on variability within this subsample and factors that are relevant for women living with homelessness such as sources of trauma or parental roles. A woman only analysis, too rarely seen in studies of homelessness, ensures that the focus remains on women within the sample as gender analyses largely focus on comparing experiences with those of men. With 24 months of follow-up and novel risk variables not previously examined in a Housing First analysis we can provide unique point-in-time and longitudinal evidence not previously considered because of prior sample size and variable availability limitations.

The present study employs the largest longitudinal sample of women experiencing homelessness and mental illness in a Housing First randomized controlled trial (RCT) and extends the follow up duration typically seen to 24 months. Our objectives were: (i) To compare change at the end of the study in health and social outcomes between women who received and did not receive the Housing First intervention; ii) To examine whether the Housing First intervention leads to better housing stability over 24 months, and health and social and outcomes between 6- to 24-months post enrolment; and, (iii) To explore baseline demographic, social, health measures, and ACEs as predictors of key study outcomes during the study period.

Methods

Methods and measures for this analysis have been extensively described elsewhere [49, 53-55]. Briefly, the Canadian At Home/Chez Soi five city RCT randomized Housing First among adults experiencing homelessness and serious mental illness [54]. Participants randomized to the Housing First (HF) intervention received scattered-site housing and either moderate or high needs-based services to support their recovery and integration into the community (i.e., Intensive Case Management or Assertive Community Treatment). Participants randomized to treatment-as-usual (TAU) had access to housing and services through other community programs. Each site had additional focus on a unique population (e.g., ethno-racial populations in Toronto and Indigenous populations in Winnipeg) and had specialized services to address their needs. Fidelity assessments ensured strong adherence to program theory of change[56]. Information about the study, such as the randomization process and a description of the intervention, was provided to all study participants prior to enrolment. An informed consent process was created to ensure that participants had the time and information to understand the complexities of the trial and follow-up activities. All participants provided written informed consent prior to enrolment. All enrolment and consent documents were harmonized across sites, however, each site had separate approval from relevant university/site Research Ethics Boards.

Over 24 months, participants were interviewed every 3 months to assess housing stability and every 6 months to evaluate health and social outcomes. Previous results from 2,148 participants (including 683 women) demonstrated that those in the HF arm showed significant improvements in housing stability, quality of life, and community functioning compared to participants in usual care [47, 49, 53]. This paper reports on a subgroup analysis of 653

participants who self-identified as women and had at least one follow-up after their baseline assessment (96%), including 588 followed to 24 months. Across all sites, 374 participants were from the HF group and 279 from the TAU group (Supplemental Figure S1).

Outcomes and other study variables

We describe in this section the outcome variables and any other scales used in this analysis. Stable housing was defined as "living in one's own room, apartment, or house, or with family, with an expected duration of residence of 6 months or more or tenancy rights" while enrolled in the study, taking into account participants' move in/out dates for each type of residence (e.g., street place, unstable residence, stable residence, institution)[57]. The *percentage of days stably housed during the 24-month period* was calculated as the total number of days stably housed divided by the total number of days for which any type of residence data were provided by the participant over 24 months [49, 54]. Unlike the other outcomes, housing stability was measured at the end of the follow-up period for several reasons. First, the treatment group establishes stable housing within the first 1-3 months of enrolment and housing status rarely changes after that point in time. A small proportion of the treatment group was not consistently stably housed throughout the duration of the program; thus, this single cumulative measure captures housing stability better than if we had measured it at each follow-up time point. In sum, this cumulative housing stability is the best measure for the outcome of housing as it does not demonstrate incremental changes over the 24-month follow-up period.

The other outcomes were assessed via in-person interviews at baseline, 6-, 12-, 18, and 24 months post-enrollment.

The Lehman Quality of Life Interview 20 (QoLI-20) measured participants' self-reported life satisfaction on a 7-point Likert scale in the domains of family, finance, leisure, living, safety,

social, and overall quality of life. Scores range from 7 to 140, with higher scores indicating better quality of life [58].

Community functioning was measured using the Multnomah Community Ability Scale (MCAS), a 17-item scale capturing degree of clinician/observer-rated functional ability in health, adaptation, social skills, and behavior, with higher scores indicating less impairment.

The Colorado Symptom Index (CSI) assessed how often specific psychiatric symptoms were experienced, from 'at least every day' to 'not at all'. Fourteen self-report items were summed (range 14 to 40), with lower scores reflecting fewer symptoms [59, 60].

Emergency department (ED) visits were measured using self-reported counts in the past 6 months. Past-month substance-related problems were measured via the 5-item Global Assessment of Individual Needs Short Screener (GAIN-SS); higher values correspond to greater problem severity [54].

The Community Integration Scale (CIS) measured self-reported psychological integration with the immediate community on a scale from 4-20; physical integration used counts of previous month social activities (e.g., attending a concert, meeting people for coffee) on a scale of 0-7. Higher scores on both reflect greater integration [61].

Baseline and predictor variables included in analyses included data collected on traumatic events that occurred before the age of 18 years using the Adverse Childhood Experiences module assessing ten early childhood adversities (yes/no items): emotional, physical or sexual abuse; emotional or physical neglect; household substance use, household mental illness and household criminal justice involvement; mother treated violently, and parental separation or divorce [62]. Scores ranged from 0 to 10 with higher scores indicating greater childhood adversity. We created two categories representing high levels of reported ACEs, ≥5 adversities, and four or fewer

including none [63, 64]. Mental health diagnoses and levels of suicidality were assessed via the MINI Neuropsychiatric Interview 6.0 administered as part of the baseline enrolment interview[65]. No, low, moderate and high levels of suicidality were dichotomized into 'no/low' and 'moderate/high' suicidality. Information about all other variables (e.g., demographics, financial support of children, sources of income) was obtained via self-report during the in-person baseline interviews.

Statistical Analyses

Descriptive statistics for a wide range of descriptors were selected to characterize the sample by treatment group. Longitudinal outcomes were analyzed using linear mixed analysis of repeated measures models for continuous outcomes and generalized estimating equations for count outcomes (Poisson distribution for CIS-Physical Integration and negative binomial distribution for GAIN-SS and ED Visits), assuming an unstructured correlation matrix for the repeated measures.

For the first objective, for each group, the adjusted change from baseline to 24-months follow-up was estimated by mean differences and 95% confidence intervals (continuous outcomes), and rate ratios and 95% confidence intervals (count outcomes). We fit models that included treatment group (fixed effect, HF vs TAU), categorical time (24-months vs baseline), interaction of treatment x time, and study city and need level at baseline (high vs. moderate) as covariates.

For the second objective, in a more detailed examination of possible differential effect over time, we tested for differences between HF and TAU at each follow-up time point from 6- to 24-months, adjusting for baseline outcomes. Models included treatment group, categorical time (6-, 12-, 18-, 24-months), interaction of treatment x time, and city, need level and baseline

outcome values as covariates. At each time point, we compared HF vs TAU by estimating mean differences and 95% confidence intervals or rate ratios and 95% confidence intervals. We modeled the total percentage of days stably housed over 24-months as a continuous outcome and fitted a linear regression model including treatment group, and city, need level and percentage of days stably housed in the three months prior to baseline as covariates

For the third objective, the differences between HF and TAU were no longer the focus. Rather we took advantage of the large sample of women in a study on homelessness to identify predictors of our outcomes, uncoupling the participants from their assigned trial arm. To accomplish this we expanded the models from the second objective and added the following baseline predictors: age at enrolment (continuous), education (1=less than high school, 0=high school or higher), duration of homelessness (1=≥3 ,0= <3 years), whether minor children were being supported by the participant (1=yes, 0=no), level of suicidality (1=moderate or high, 0=mild or none), and high level of ACEs at baseline (1=≥5 , 0=<5 ACEs). These predictors were chosen because they represent a knowledge gap or have yielded inconsistent findings on longitudinal studies of women experiencing homelessness. We were unable to include ethnicity or Indigenous identity due to multicollinearity with study city.

Multiple imputation with chained equations (MICE) was used to handle missing data from loss to follow-up, withdrawal, skipped interviews, nonresponse on specific items, and systematically measured lack of interviewer confidence in participant responses. We imputed all outcomes simultaneously and MICE imputation models included all variables needed to address three objectives[66] . Although outcome missing rates varied between 0.53% to 31.9% (Supplemental Table S1), approximately 70% of the participants had at least one missing value in at least one outcome in at least one point in time. Therefore, we chose 70 imputations

following guidelines given by White et al. (2010) that the percentage of cases with incomplete data should be similar to the number of imputations generated [66]. Comparisons between observed and imputed data via diagnostic plots were used to assess the quality and plausibility of the imputations[67]

Data were imputed using Stata software (mi impute chained, Stata Statistical Software: Release 13. College Station, TX: StataCorp LP) and analyzed using SAS (PROC MIXED, GENMOD , PROC MIANALYZE, SAS 9.4, SAS Institute Inc, Cary, NC) Figures were created using the ggplot2 package in R software version 3.4.0 (R Core Team (2017) [68]. All statistical tests were two-tailed and significance was defined as $p<.05$. No adjustments for multiple testing were applied [69].

Results

We took advantage of the rich data collected in this study and present a broad array of participant demographic, social, and health characteristics at enrolment (Table 1). Fifteen percent of the women were born outside of Canada (median time in Canada: TAU=25yrs; HF = 21yrs). Three percent of women were employed at baseline and 90% received social assistance of some type as a source of income. Around 40% (*n*=274) reported having minor children, and of these, 22% (*n*=60) were providing full or partial financial support to the child(ren). The most prevalent mental illnesses were depressive episodes, PTSD, and alcohol or substance dependence. Approximately 40% had moderate-to-high suicidality, around 40% had post-traumatic stress disorder, about 60% reported ≥5 ACEs, and the majority had three or more co-morbid medical conditions.

Table 2 displays results from models examining changes from baseline to 24 months for those in HF and TAU arms for each of our social, well-being and, and comparing groups with respect to housing stability over the 24-month period. Over the 24-month follow-up period, substantial improvements were demonstrated for all the outcomes for both HF and TAU participants. The mean percentage of days spent stably housed during follow-up for women receiving HF was 74.8% (95%CI=71.7%–77.8%) compared with 37.9% (95%CI=34.4%–41.3%) TAU, a difference of 36.9% (95%CI=32.4%–41.4%, $p<0.001$). Of note, mean total QoLI-20 scores improved 16.0 points (95%CI=13.6–18.4) and 13.4 points (95%CI=10.6–16.2) in the HF and TAU groups, respectively, and number of ED visits decreased by approximately 60% in both groups.

These improvements over the 24-month period are more clearly illustrated in Figure 1 for the HF intervention and TAU groups for all social and health unadjusted outcomes at *each observation point* from baseline to 24-months. For all outcomes, except CIS-Physical where scores remained flat throughout the follow-up, both groups experienced similar significant change from baseline to 24-months (Figure 1).

Figure 1 – Health and social outcomes among Women who are Homeless and Mentally Ill, unadjusted means and 95% confidence intervals over 24 months of follow-up by study time point and treatment group

Table 3 displays changes over time in greater detail through results of the models to assessing whether social and health outcomes were better in HF vs. TAU groups *at each follow-up point*. For condition-specific quality of life, significant improvements of 7.1 and 6.1 points

were observed in the HF group at the 6- and 12-month follow-ups, respectively. The HF group also showed significant improvements in psychological community integration by 0.7 points at the 6-month follow-up. However, in the last year of the trial, the ED visits fell faster for the TAU group (0.6 visits) compared to the HF group (1.1 visits) at 18 months, with a rate ratio of 1.81 (95%CI=1.26–2.60).

Table 4 no longer focuses on differences between HF and TAU as results presented here uncouple participants from their assigned treatment arms and identify predictors of our outcomes. Moderate to high suicidality had the most consistent association to poorer health and service utilization outcomes at 24 months follow-up. Compared to those with no or low suicidality at baseline, moderate-to-high suicidality at baseline was associated with nearly three points on the CSI indicating greater mental health symptom severity (beta=2.85, 95%CI=1.59–4.11, $p<0.001$), QoLI-20 mean scores were almost four points lower indicating reduced quality of life (beta=-3.99, 95%CI=-6.49 to -1.49, $p=0.002$), and almost 1.5 more ED visits (rate ratio=1.44, 95%CI=1.10–1.87, $p=0.007$). Low education was also associated with several outcomes. Compared to those with higher education, women with less than a high school level of education at baseline predicted lower community functioning (MCAS) scores (beta=-1.32, 95%CI=-2.27 to -0.37, $p=0.006$), 15% fewer social activities (CIS) in the previous month (rate ratio=0.85, 95%CI=0.77–0.93, $p<0.001$), and more severe substance-related problems at 24 months follow-up (rate ratio=1.27, 95%CI=1.06–1.52, $p=0.009$). Having been homeless for ≥3 years at baseline was associated with lower percentage days stably housed at 24-months (beta=-6.1%, 95%CI=-11.0% to -1.3%, $p=0.013$). An ACE score of 5 or more was associated with a 1.5 point increase in mental illness symptom severity (95%CI=0.25–2.76, $p=0.019$). Finally, we

explored and found no evidence that baseline levels of ACEs and suicidality, separately, were effect modifiers for any of the above analyses.

Discussion

This is the first analysis of women's outcomes in an RCT of Housing First. Prior studies and trials on housing interventions for people who experience homelessness often included very small numbers of women or few variables reflecting women's unique needs and experiences that have precluded documentation of the heterogeneity of experiences and in-depth understanding of this population.

In this Canadian multi-site study, the largest longitudinal sample of women experiencing homelessness to date, the intervention group experienced housing stability for 75% of the 24-month follow-up, as compared to 38% for women in the TAU group, a finding similar to previously published reports of At Home/Chez Soi participants, the majority of whom were men [49, 53]. Even if the large proportion of time spent stably housed for women who received Housing First compared to the TAU group did not translate into greater improvements in health and social outcomes by the end of the study, there were some early differences favoring the treatment group in the first year for quality of life and psychological community integration. Immediate placement in housing and early housing stability for HF compared to TAU [70]participants likely contributed to faster improvements in these outcomes.

While ED visits declined in a similar fashion for HF and TAU over the 24-month follow-up, an unanticipated finding was a significant reduction in ED visits of 0.5 visits at 18 months for the TAU group over the HF group. For this rare count outcome, the dip in ED visits for TAU at 18 months could be a chance improvement as both groups had similar levels of ED visits by 24

months. Analyses of the full At Home/Chez Soi trial data did not show differences for this outcome[49]. One possibility is that uncertainty around continued housing as the trial was ending may have created high levels of stress in the Housing First group, but if this were the case, we would have likely seen similar changes in other study outcomes or among men in the analyses of the full sample.

Since improvements in outcomes over the 24-month period were similar for both the HF and TAU groups, we moved beyond the intention-to-treat analysis and examined baseline predictors for our outcomes across the full sample. We focused on baseline suicidality, ACEs, and parental responsibilities as predictors- variables rarely examined in past studies of supported housing or Housing First [35, 43-45, 71, 72]. As might be expected, women with ≥5 ACEs or moderate-to-high levels of suicidality at baseline showed poorer improvement by 24 months compared to those with low ACE or suicidality scores. Moderate-to-high suicidality was a strong predictor of poorer outcomes at 24 months, including mental health, quality of life, community integration, and ED visits. Past cross-sectional studies of combined samples of homelessness men and women have demonstrated similar links between suicidality, childhood and adult trauma, substance abuse, and mental health [20, 21, 73]. However, while cross-sectional associations between suicidality and substance among women who are homeless have been reported in the literature[19, 74] our study is the first to examine these associations longitudinally.

Contrary to what we might have anticipated on the importance of ACEs in predicting multiple study outcomes given past research[75], we found that high levels of ACEs only predicted significantly worse scores on mental illness severity (CSI). This is consistent with a recent study of the full sample of At Home/Chez Soi where ACEs total scores predicted several

mental health conditions at 24-months[51]. Our choice of a cutoff representing high numbers of ACEs, while informed by existing literature, may have limited our ability to detect an association. Alternatively, ACEs may be limited in capturing relevant adverse conditions. A recent study has suggested that an expanded ACEs tool might be used to capture important domains currently missing such as severe poverty or family loss and separation[76], which might be particularly relevant in capturing sources of trauma and adversity for women experiencing homelessness. Our findings also demonstrated that low educational attainment at baseline was a significant predictor of worse mental health, community integration, and substance use outcomes independently of the other variables in the model. Low educational attainment may be a proxy for other factors such as low health literacy which might impact management of health conditions and requires further investigation [77]. Providing economic support for dependent children was not related to any of our study outcomes.

Although the At Home/Chez Soi support services were important for intervention participants to remain stably housed and make gains in health and social standing, these supports and services were not designed in this Housing First program to be tailored to women's unique needs. Psychological housing stability which includes considerations of safety, security and feelings of home, is particularly important for women while the At Home/Chez Soi focused predominantly on material housing stability[78]. Women who are unstably housed or homeless need services that address trauma and promote informal peer support networks [33, 79]. Although staff were trained to be responsive to individual needs, the At Home/Chez Soi intervention was not specifically designed to provide the latter two resources. In addition, more than 40% of our participants were homeless for more than three years at enrolment. Two years of stable housing with mental health supports may not have been sufficient to resolve chronic

distress from being homeless with co-morbid health, trauma and substance use problems, particularly given the high rates of reported PTSD and ACEs[14].

The lack of greater improvements in the HF arm in this analysis might have been due to the intensive follow-up schedule that could have led to a Hawthorne effect among the TAU group, and higher loss to follow-up in the TAU group might have contributed to smaller differences between groups at 24 months. Despite our sample of homeless women being relatively large, it may not have been sufficient to detect small effects. The At Home/Chez Soi study did not capture gender-sensitive outcomes specific to women experiencing homelessness and therefore we may have missed important changes, such as those associated with positive social support, increased safety from crime and victimization, or strengthening of relationships with children, family or friends[3, 80].

Our study adds to the growing yet sparse literature on the characteristics and needs of women experiencing homelessness and the effectiveness of interventions to eliminate homelessness. Given that women make up more than one third of the homeless population, and their needs differ from that of men[3, 14, 27, 32], it is important that their experiences with homelessness and recovery receive ample focus to better inform the design and implementation of interventions that meet their unique needs. Moreover, the homelessness epidemic has only worsened in the context of the COVID pandemic so the issues remain relevant and these new analyses can provide insights[81].

References

1. Gaetz S, Dej E, Richter T, Redman M. The State of Homelessness in Canada 2016. Toronto: 2016.
2. Employment and Social Development Canada. Everyone Counts 2018: Highlights - Preliminary results from the second nationally coordinated Point-in-Time count of homelessness in Canadian communities. 2019.
3. Schwan K, Versteegh A, Perri M, Caplan R, Baig K, Dej E, et al. The State of Women's Housing Need & Homelessness in Canada: A Literature Review. Hache, A., Nelson, A., Kratochvil, E., & Malenfant, J. (Eds). Toronto, ON: Canadian Observatory on Homelessness Press. 2020.
4. Duke A, Searby A. Mental Ill Health in Homeless Women: A Review. Issues Ment Health Nurs. 2019;40(7):605-12. doi: 10.1080/01612840.2019.1565875. PubMed PMID: WOS:000474047300001.
5. Ferguson KM, Bender K, Thompson SJ. Gender, coping strategies, homelessness stressors, and income generation among homeless young adults in three cities. Social science & medicine (1982). 2015;135:47-55. Epub 2015/05/06. doi: 10.1016/j.socscimed.2015.04.028. PubMed PMID: 25942470.
6. Strehlau V, Torchalla I, Kathy L, Schuetz C, Krausz M. Mental health, concurrent disorders, and health care utilization in homeless women. Journal of psychiatric practice. 2012;18(5):349-60. Epub 2012/09/22. doi: 10.1097/01.pra.0000419819.60505.dc. PubMed PMID: 22995962.
7. Bassuk EL, Buckner JC, Perloff JN, Bassuk SS. Prevalence of mental health and substance use disorders among homeless and low-income housed mothers. The American journal of psychiatry. 1998;155(11):1561-4. Epub 1998/11/13. doi: 10.1176/ajp.155.11.1561. PubMed PMID: 9812118.
8. Shinn M, Knickman JR, Weitzman BC. Social relationships and vulnerability to becoming homeless among poor families. The American psychologist. 1991;46(11):1180-7. Epub 1991/11/01. PubMed PMID: 1772155.
9. Banyard VL, Graham-Bermann SA. Surviving poverty: stress and coping in the lives of housed and homeless mothers. The American journal of orthopsychiatry. 1998;68(3):479-89. Epub 1998/08/01. PubMed PMID: 9686299.
10. Nelson G, Patterson M, Kirst M, Macnaughton E, Isaak CA, Nolin D, et al. Life changes among homeless persons with mental illness: a longitudinal study of housing first and usual treatment. Psychiatric services (Washington, DC). 2015;66(6):592-7. Epub 2015/02/18. doi: 10.1176/appi.ps.201400201. PubMed PMID: 25686813.
11. Riley ED, Weiser SD, Sorensen JL, Dilworth S, Cohen J, Neilands TB. Housing Patterns and Correlates of Homelessness Differ by Gender among Individuals Using San Francisco Free Food Programs. Journal of Urban Health. 2007;84(3):415-22. doi: 10.1007/s11524-006-9153-3.
12. Cheung AM, Hwang SW. Risk of death among homeless women: a cohort study and review of the literature. CMAJ : Canadian Medical Association journal = journal de l'Association medicale canadienne. 2004;170(8):1243-7. Epub 2004/04/14. PubMed PMID: 15078846; PubMed Central PMCID: PMCPMC385354.
13. Gutwinski S, Schreiter S, Deutscher K, Fazel S. The prevalence of mental disorders among homeless people in high-income countries: An updated systematic review and meta-regression analysis. PLoS Med. 2021;18(8):e1003750. Epub 2021/08/24. doi: 10.1371/journal.pmed.1003750. PubMed PMID: 34424908; PubMed Central PMCID: PMCPMC8423293.
14. Milaney K, Williams N, Lockerbie SL, Dutton DJ, Hyshka E. Recognizing and responding to women experiencing homelessness with gendered and trauma-informed care. Bmc Public Health. 2020;20(1). doi: 10.1186/s12889-020-8353-1. PubMed PMID: WOS:000522413900001.
15. Brunette M, Drake RE. Gender differences in homeless persons with schizophrenia and substance abuse. Community mental health journal. 1998;34(6):627-42. Epub 1998/12/02. PubMed PMID: 9833202.

16. Rich AR, Clark C. Gender differences in response to homelessness services. Evaluation and Program Planning. 2005;28:69-81.
17. Roll CN, Toro PA, GL. O. Characteristics and experiences of homeless adults: A comparison of single men, single women, and women with children. Journal of Community Psychology. 1999;27:189-98.
18. Torchalla I, Strehlau V, Li K, Aube Linden I, Noel F, Krausz M. Posttraumatic stress disorder and substance use disorder comorbidity in homeless adults: Prevalence, correlates, and sex differences. Psychology of addictive behaviors : journal of the Society of Psychologists in Addictive Behaviors. 2014;28(2):443-52. Epub 2013/08/07. doi: 10.1037/a0033674. PubMed PMID: 23915373.
19. Benda BB. Gender differences in predictors of suicidal thoughts and attempts among homeless veterans that abuse substances. Suicide & life-threatening behavior. 2005;35(1):106-16. Epub 2005/04/22. doi: 10.1521/suli.35.1.106.59262. PubMed PMID: 15843327.
20. Desai RA, Liu-Mares W, Dausey DJ, Rosenheck RA. Suicidal ideation and suicide attempts in a sample of homeless people with mental illness. The Journal of nervous and mental disease. 2003;191(6):365-71. Epub 2003/06/27. doi: 10.1097/01.nmd.0000071584.88965.e1. PubMed PMID: 12826917.
21. Eynan R, Langley J, Tolomiczenko G, Rhodes AE, Links P, Wasylenki D, et al. The association between homelessness and suicidal ideation and behaviors: results of a cross-sectional survey. Suicide & life-threatening behavior. 2002;32(4):418-27. Epub 2002/12/28. PubMed PMID: 12501966.
22. Rodriguez-Moreno S, Vázquez JJ, Roca P, Panadero S. Differences in stressful life events between men and women experiencing homelessness. Journal of Community Psychology. 2021;49(2):375-89. doi: https://doi.org/10.1002/jcop.22465.
23. Herman DB, Susser ES, Struening EL, Link BL. Adverse childhood experiences: are they risk factors for adult homelessness? American journal of public health. 1997;87(2):249-55. Epub 1997/02/01. PubMed PMID: 9103105; PubMed Central PMCID: PMCPMC1380802.
24. Montgomery AE, Cutuli JJ, Evans-Chase M, Treglia D, Culhane DP. Relationship among adverse childhood experiences, history of active military service, and adult outcomes: homelessness, mental health, and physical health. American journal of public health. 2013;103 Suppl 2:S262-8. Epub 2013/10/24. doi: 10.2105/ajph.2013.301474. PubMed PMID: 24148064; PubMed Central PMCID: PMCPMC3969137.
25. Roos LE, Mota N, Afifi TO, Katz LY, Distasio J, Sareen J. Relationship between adverse childhood experiences and homelessness and the impact of axis I and II disorders. American journal of public health. 2013;103 Suppl 2:S275-81. Epub 2013/10/24. doi: 10.2105/ajph.2013.301323. PubMed PMID: 24148049; PubMed Central PMCID: PMCPMC3969113.
26. Patterson ML, Moniruzzaman A, Somers JM. Setting the stage for chronic health problems: cumulative childhood adversity among homeless adults with mental illness in Vancouver, British Columbia. BMC Public Health. 2014;14:350. Epub 2014/04/15. doi: 10.1186/1471-2458-14-350. PubMed PMID: 24726046; PubMed Central PMCID: PMCPMC3991866.
27. Schwan K, Vaccaro M, Reid L, Ali N, Baig K. The Pan-Canadian Women's Housing & Homelessness Survey. Toronto, ON: Canadian Observatory on Homelessness, 2021.
28. Milaney K, Williams N, Lockerbie SL, Dutton DJ, Hyshka E. Recognizing and responding to women experiencing homelessness with gendered and trauma-informed care. BMC Public Health. 2020;20(1):397. Epub 2020/03/29. doi: 10.1186/s12889-020-8353-1. PubMed PMID: 32216770; PubMed Central PMCID: PMCPMC7099814.
29. Torchalla I, Linden IA, Strehlau V, Neilson EK, Krausz M. "Like a lots happened with my whole childhood": violence, trauma, and addiction in pregnant and postpartum women from Vancouver's Downtown Eastside. Harm Reduction Journal. 2015;12(1):1. doi: 10.1186/1477-7517-12-1.

30. Shier ML, Jones ME, Graham JR. Sociocultural Factors to Consider When Addressing the Vulnerability of Social Service Users: Insights From Women Experiencing Homelessness. Affilia. 2011;26(4):367-81. doi: 10.1177/0886109911428262.
31. Winetrobe H, Wenzel S, Rhoades H, Henwood B, Rice E, Harris T. Differences in Health and Social Support between Homeless Men and Women Entering Permanent Supportive Housing. Women's Health Issues. 2017;27(3):286-93. doi: https://doi.org/10.1016/j.whi.2016.12.011.
32. Perri M, O'Campo P. A gap in knowledge surrounding urban housing interventions: a call for gender redistribution. Health Promot Int. 2021;36(4):908-12. Epub 2021/05/12. doi: 10.1093/heapro/daab062. PubMed PMID: 33975348.
33. Oudshoorn A, Forchuk C, Hall J, Smith-Carrier T, Van Berkum A. An evaluation of a Housing First program for chronically homeless women. Journal of Social Inclusion. 2018;9(2):34-50. doi: 10.36251/josi.136. PubMed PMID: WOS:000454531300004.
34. Perri M, Metheny N, Matheson FI, Potvin K, O'Campo P. Finding opportunity in the COVID-19 crisis: prioritizing gender in the design of social protection policies. Health Promot Int. 2022;37(1). Epub 2021/05/17. doi: 10.1093/heapro/daab045. PubMed PMID: 33993289; PubMed Central PMCID: PMCPMC8194510.
35. Cameron A, Abrahams H, Morgan K, Williamson E, Henry L. From pillar to post: homeless women's experiences of social care. Health Soc Care Community. 2016;24:345-32.
36. Fotheringham S, Walsh CA, Burrowes A. 'A place to rest': the role of transitional housing in ending homelessness for women in Calgary, Canada. Gender, Place & Culture. 2014;21(7):834-53. doi: 10.1080/0966369X.2013.810605.
37. Blehar MC. Public health context of women's mental health research. The Psychiatric clinics of North America. 2003;26(3):781-99. Epub 2003/10/18. doi: 10.1016/s0193-953x(03)00039-x. PubMed PMID: 14563109.
38. Institute of Medicine. Sex-Specific Reporting of Scientific Research: A Workshop Summary. Washington, DC: The National Academies Press. https://doi.org/10.17226/13307. 2012.
39. Jahn I, Bornhorst C, Gunther F, Brand T. Examples of sex/gender sensitivity in epidemiological research: results of an evaluation of original articles published in JECH 2006-2014. Health research policy and systems. 2017;15(1):11. Epub 2017/02/17. doi: 10.1186/s12961-017-0174-z. PubMed PMID: 28202078; PubMed Central PMCID: PMCPmc5312447.
40. Klap R, Humphreys K. Designing Studies for Sex and Gender Analyses: How Research Can Derive Clinically Useful Knowledge for Women's Health. Women's health issues : official publication of the Jacobs Institute of Women's Health. 2019;29 Suppl 1:S12-s4. Epub 2019/06/30. doi: 10.1016/j.whi.2019.05.002. PubMed PMID: 31253235.
41. Welch V, Doull M, Yoganathan M, Jull J, Boscoe M, Coen SE, et al. Reporting of sex and gender in randomized controlled trials in Canada: a cross-sectional methods study. Res Integr Peer Rev. 2017;2:15-. doi: 10.1186/s41073-017-0039-6. PubMed PMID: 29451565.
42. Tsai J, Byrne TH. National Utilization Patterns of Veterans Affairs Homelessness Programs in the Era of Housing First. Psychiatric Services. 2019;70(4):309-15. doi: 10.1176/appi.ps.201800393. PubMed PMID: WOS:000462848600008.
43. Cheng AL, Kelly PJ. Impact of an integrated service system on client outcomes by gender in a national sample of a mentally ill homeless population. Gender medicine. 2008;5(4):395-404. Epub 2008/12/26. doi: 10.1016/j.genm.2008.10.003. PubMed PMID: 19108812.
44. Nesse L, Gonzalez Marianne T, Aamodt G, Raanaas Ruth K. Recovery, quality of life and issues in supported housing among residents with co-occurring problems: a cross-sectional study. Advances in Dual Diagnosis. 2020;13(2):73-87. doi: 10.1108/ADD-10-2019-0014.
45. Tinland A, Zemmour K, Auquier P, Boucekine M, Girard V, Loubiere S, et al. Homeless women with schizophrenia reported lower adherence to their medication than men: results from the French

Housing First experience. Social Psychiatry and Psychiatric Epidemiology. 2017;52(9):1113-22. doi: 10.1007/s00127-017-1411-z. PubMed PMID: WOS:000408956600005.
46. Adame AL, Perry C, Pierce E. Community and Housing First: A qualitative analysis of USA residents' perspectives. Health Soc Care Community. 2020;28(4):1292-300. Epub 2020/03/05. doi: 10.1111/hsc.12962. PubMed PMID: 32128910.
47. Edens EL, Mares AS, Rosenheck RA. Chronically homeless women report high rates of substance use problems equivalent to chronically homeless men. Women's health issues : official publication of the Jacobs Institute of Women's Health. 2011;21(5):383-9. Epub 2011/06/28. doi: 10.1016/j.whi.2011.03.004. PubMed PMID: 21703865.
48. Aubry T, Tsemberis S, Adair CE, Veldhuizen S, Streiner D, Latimer E, et al. One-Year Outcomes of a Randomized Controlled Trial of Housing First With ACT in Five Canadian Cities. Psychiatric Services. 2015;66(5):463-9. doi: 10.1176/appi.ps.201400167.
49. Stergiopoulos V, Hwang SW, Gozdzik A, Nisenbaum R, Latimer E, Rabouin D, et al. Effect of scattered-site housing using rent supplements and intensive case management on housing stability among homeless adults with mental illness: a randomized trial. Jama. 2015;313(9):905-15. Epub 2015/03/04. doi: 10.1001/jama.2015.1163. PubMed PMID: 25734732.
50. Tsemberis S, Gulcur L, Nakae M. Housing First, consumer choice, and harm reduction for homeless individuals with a dual diagnosis. American journal of public health. 2004;94(4):651-6. Epub 2004/04/01. PubMed PMID: 15054020; PubMed Central PMCID: PMCPmc1448313.
51. Liu M, Mejia-Lancheros C, Lachaud J, Latimer E, Aubry T, Somers J, et al. Overall and Gender-specific Associations between Dimensions of Adverse Childhood Experiences and Mental Health Outcomes among Homeless Adults: Associations Générales et Sexospécifiques Entre les Dimensions des Expériences Défavorables de L'enfance et les Résultats de Santé Mentale Chez les Adultes Sans Abri. Can J Psychiatry. 2021;66(10):906-17. Epub 2021/01/28. doi: 10.1177/0706743721989158. PubMed PMID: 33502253; PubMed Central PMCID: PMCPMC8573704.
52. Paquette K, Pannella Winn LA. The Role of Recovery Housing: Prioritizing Choice in Homeless Services. Journal of dual diagnosis. 2016;12(2):153-62. Epub 2016/04/12. doi: 10.1080/15504263.2016.1175262. PubMed PMID: 27064834.
53. Aubry T, Goering P, Veldhuizen S, Adair CE, Bourque J, Distasio J, et al. A Multiple-City RCT of Housing First With Assertive Community Treatment for Homeless Canadians With Serious Mental Illness. Psychiatric services (Washington, DC). 2016;67(3):275-81. Epub 2015/12/02. doi: 10.1176/appi.ps.201400587. PubMed PMID: 26620289.
54. Goering PN, Streiner DL, Adair C, Aubry T, Barker J, Distasio J, et al. The At Home/Chez Soi trial protocol: a pragmatic, multi-site, randomised controlled trial of a Housing First intervention for homeless individuals with mental illness in five Canadian cities. BMJ open. 2011;1(2):e000323. Epub 2011/11/22. doi: 10.1136/bmjopen-2011-000323. PubMed PMID: 22102645; PubMed Central PMCID: PMCPmc3221290.
55. Aquin JP, Roos LE, Distasio J, Katz LY, Bourque J, Bolton JM, et al. Effect of Housing First on Suicidal Behaviour: A Randomised Controlled Trial of Homeless Adults with Mental Disorders. Can J Psychiatry. 2017;62(7):473-81. Epub 2017/07/07. doi: 10.1177/0706743717694836. PubMed PMID: 28683228; PubMed Central PMCID: PMCPMC5528985.
56. Nelson G, Macnaughton E, Caplan R, MacLeod T, Townley G., Piat M, et al. Follow-up Implementation and fidelity Evaluation of the mental Health Commission of Canada's At Home/Chez Soi Project: Cross-site Report. Mental Health Commission of Canada, 2013.
57. Tsemberis S., McHugo G., Williams V., Hanrahan P., Stefancic A. Measuring homelessness and residential stability: The residential time-line follow-back inventory. Journal of Community Psychology. 2007;35:29-42.

58. Uttaro T., Lehman A. Graded response modeling of the Quality of Life Interview. Evaluation and Program Planning. 1999;22:41-52.
59. Barker S, Barron N, McFarland BH, Bigelow DA, Carnahan T. A community ability scale for chronically mentally ill consumers: Part II. Applications. Community Ment Health Journal. 1994;30(5):459-72.
60. Dickerson FB, Origoni AE, Pater A, Friedman BK, Kordonski WM. An expanded version of the Multnomah Community Ability Scale: anchors and interview probes for the assessment of adults with serious mental illness. Community Ment Health Journal. 2003;39(2):131-7.
61. Conrad KJ, Yagelka JR, Matters MD, Rich A.R., Williams V., Buchanan M. Reliability and validity of a modified Colorado Symptom Index in a national homeless sample. Mental Health Services Research. 2001;3:141-53.
62. Felitti VJ, Anda RF, Nordenberg D, Williamson DF, Spitz AM, Edwards V, et al. Relationship of childhood abuse and household dysfunction to many of the leading causes of death in adults. The Adverse Childhood Experiences (ACE) Study. Am J Prev Med. 1998;14(4):245-58. Epub 1998/06/23. doi: 10.1016/s0749-3797(98)00017-8. PubMed PMID: 9635069.
63. Chapman DP, Whitfield CL, Felitti VJ, Dube SR, Edwards VJ, Anda RF. Adverse childhood experiences and the risk of depressive disorders in adulthood. J Affect Disord. 2004;82(2):217-25. Epub 2004/10/19. doi: 10.1016/j.jad.2003.12.013. PubMed PMID: 15488250.
64. Messina N, Grella C. Childhood trauma and women's health outcomes in a California prison population. American journal of public health. 2006;96(10):1842-8. Epub 2006/09/30. doi: 10.2105/ajph.2005.082016. PubMed PMID: 17008581; PubMed Central PMCID: PMCPMC1586137.
65. Lecrubier Y, Sheehan DV, Weiller E, Amorim P, Bonora I, Harnett Sheehan K, et al. The Mini International Neuropsychiatric Interview (MINI). A short diagnostic structured interview: reliability and validity according to the CIDI. European Psychiatry. 1997;12(5):224-31. doi: https://doi.org/10.1016/S0924-9338(97)83296-8.
66. White IR, Royston P, Wood AM. Multiple imputation using chained equations: Issues and guidance for practice. Statistics in Medicine 2010;30:377-99.
67. van Buuren S. Flexible Imputation of Missing Data: CRC Press; 2012.
68. R: A language and environment for statistical computing. R Foundation for Statistical Computing V, Austria (https://www.R-project.org/).
69. Streiner DL, Norman GR. Correction for multiple testing: is there a resolution? . Chest. 2011;140(1):16-18.
70. Aubry T, Nelson G, Tsemberis S. Housing First for People with Severe Mental Illness Who are Homeless: A Review of the Research and Findings from the at Home—Chez soi Demonstration Project. The Canadian Journal of Psychiatry. 2015;60(11):467-74. doi: 10.1177/070674371506001102.
71. Hall G, Walters S, Gould H, Lim S. Housing versus treatment first for supportive housing participants with substance use disorders: A comparison of housing and public service use outcomes. Subst Abus. 2020;41(1):70-6. Epub 2018/03/13. doi: 10.1080/08897077.2018.1449049. PubMed PMID: 29528786.
72. Clifasefi SL, Collins SE. The life-enhancing alcohol-management program: Results from a 6-month nonrandomized controlled pilot study assessing a community based participatory research program in housing first. J Community Psychol. 2020;48(3):763-76. Epub 2019/11/30. doi: 10.1002/jcop.22291. PubMed PMID: 31778585.
73. Dietz TL. Substance Misuse, Suicidal Ideation, and Suicide Attempts Among a National Sample of Homeless. Journal of Social Service Research. 2010;37(1):1-18. doi: 10.1080/01488376.2011.524511.
74. Guillén AI, Marín C, Panadero S, Vázquez JJ. Substance use, stressful life events and mental health: A longitudinal study among homeless women in Madrid (Spain). Addictive Behaviors. 2020;103:106246. doi: https://doi.org/10.1016/j.addbeh.2019.106246.

75. Edalati H, Nicholls TL, Schütz CG, Somers JM, Distasio J, Aubry T, et al. Examining the Relationships between Cumulative Childhood Adversity and the Risk of Criminal Justice Involvement and Victimization among Homeless Adults with Mental Illnesses after Receiving Housing First Intervention. Can J Psychiatry. 2020;65(6):409-17. Epub 2020/01/30. doi: 10.1177/0706743720902616. PubMed PMID: 31994918; PubMed Central PMCID: PMCPMC7265607.
76. Mersky JP, Janczewski CE, Topitzes J. Rethinking the Measurement of Adversity. Child Maltreat. 2017;22(1):58-68. Epub 2016/12/07. doi: 10.1177/1077559516679513. PubMed PMID: 27920222.
77. Farrell SJ, Dunn M, Huff J. Examining Health Literacy Levels in Homeless Persons and Vulnerably Housed Persons with Mental Health Disorders. Community mental health journal. 2020;56(4):645-51. Epub 2019/12/21. doi: 10.1007/s10597-019-00525-2. PubMed PMID: 31858332.
78. O'Campo P, Daoud N, Hamilton-Wright S, Dunn J. Conceptualizing Housing Instability: Experiences with Material and Psychological Instability Among Women Living with Partner Violence. Housing Studies. 2016;31(1):1-19. doi: 10.1080/02673037.2015.1021768.
79. Hopper EK, Bassuk EL, Olivet J. Shelter from the storm: Trauma-informed care in homelessness services settings. The Open Health Services and Policy Journal. 2010;3(2), 80-100.
80. Bingham B, Moniruzzaman A, Patterson M, Sareen J, Distasio J, O'Neil J, et al. Gender differences among Indigenous Canadians experiencing homelessness and mental illness. BMC psychology. 2019;7(1):57. Epub 2019/08/29. doi: 10.1186/s40359-019-0331-y. PubMed PMID: 31455404; PubMed Central PMCID: PMCPmc6712855.
81. Yakubovich AR, Maki K. Preventing Gender-Based Homelessness in Canada During the COVID-19 Pandemic and Beyond: The Need to Account for Violence Against Women. Violence Against Women. 2021:10778012211034202. doi: 10.1177/10778012211034202.

Table 1: Baseline characteristics of women participants in the Canadian At Home/Chez Soi study by randomization group (*N* = 653)

Characteristics	Treatment as Usual (N = 279)		Housing First (N = 374)	
	N	%	N	%
Age (M ± SD), y	40.2 ± 11.7		39.7 ± 11.2	
Ethnic or cultural identity				
Aboriginal	76	27.2	95	25.4
Ethno-racial	67	24.0	81	21.7
White	136	48.8	198	52.9
Education				
Less than high school	130	46.9	202	54.3
Completed high school	57	20.6	61	16.4
Some post-secondary school	90	32.5	109	29.3
Marital status				
Married/Partnered	22	7.9	16	4.3
Divorced/Separated/Widowed	74	26.5	106	28.4
Single/Never married	183	65.6	251	67.3
Country of birth, & length of time in Canada for immigrants				
Born in Canada	235	84.8	315	84.2
Immigrant, 10 years or less	8	2.8	12	3.2

**Characteristics**	**Treatment as Usual (N = 279)**		**Housing First (N = 374)**	
	N	%	N	%
Immigrant, more than 10 years	34	12.3	47	12.6
Employment status				
Unemployed (unemployed, volunteer work, retired, student, housewife/husband, other)	270	96.8	362	97.3
Employed (including self-employed and special work program)	9	3.2	10	2.7
Sources of income				
Earnings from regular/casual work or busking	16	5.7	18	4.8
Unemployment insurance	22	7.9	26	7.0
Social assistance (disability, welfare, pension, personal needs allowance)	245	87.8	338	90.4
Selling papers, crafts, pan-handling, squeegee, or $ from recycling (bottles, scrap metal)	16	5.7	22	5.9
Other (loans, theft, sex work, help from family or friends)	12	4.3	10	2.7
Housing status				
Absolutely Homeless	215	77.1	296	79.1
Precariously housed	64	22.9	78	20.9
Lifetime duration of homelessness (M ± SD)[a], y	3.8 ± 4.6		3.8 ± 4.8	

**Characteristics**	**Treatment as Usual (N = 279)**		**Housing First (N = 374)**	
	N	%	N	%
Less than 3 years duration	155	57.8	211	58.0
3 years or more duration	113	42.2	153	42.0
Has minor children (≤18 yrs of age)				
0	154	56.0	221	59.1
1-2	81	29.4	110	29.4
3 or more	40	14.6	43	11.5
Has minor children (≤18 yrs of age) & provides full/partial support				
0	88	72.7	125	82.2
1	20	16.5	16	10.5
2 or more	13	10.7	11	7.2
Relationship to minor children (≤ 18 yrs of age)				
Biological parent	121	100.0	151	98.7
Partner's, adoptive or other adult relative's child	0	0	5	3.4
MCAS[b] score (M ± SD)	60.7 ± 8.0		61.8 ± 7.8	
MINI[c] diagnostic categories				
Depressive episode	170	60.9	218	58.3
Manic or hypomanic episode	50	17.9	55	14.7
Post-traumatic stress disorder	126	45.3	145	38.8
Panic disorder	94	33.7	102	27.3

Characteristics	Treatment as Usual (N = 279)		Housing First (N = 374)	
	N	%	N	%
Mood disorder with psychotic features	54	19.4	54	14.5
Psychotic disorder	81	29.0	95	25.4
Alcohol dependence	97	34.8	108	28.9
Substance dependence	134	48.0	167	44.7
Suicidality				
Not suicidal	46	16.5	62	16.6
Low	119	42.7	170	45.5
Moderate	50	17.9	70	18.7
High	64	22.9	72	19.3
Adverse Childhood Experiences Scale, mean (SD)	5.1	3.0	5.2	3.2
Less than 5	95	39.9	137	40.5
5 or more	143	60.1	201	59.5
Comorbid medical conditions				
Less than 3	51	18.6	100	27.0
3 or more	223	81.4	270	73.0
Study Component				
Moderate Needs	144	51.6	232	62.0
High Needs	135	48.4	142	38.0

[a] Median (IQR), y 2.0 (0.8-5.0) for Treatment as Usual and 2.0 (0.7-5.0) for Housing First ; [b] MCAS: Multnomah Community Ability Scale. Possible scores range from 17 to 85, with lower scores indicating more disability.;

[c] MINI: The Mini-International Neuropsychiatric Interview

Table 2. Post-Imputation Pooled change in health and social measures from Baseline to 24 Months among Women living with Mental illness and Homelessness, Adjusted for Study City, and Need Level (ACT or ICM).

	Housing First (HF) (N=374)		Treatment as Usual (TAU) (N=279)		p-value for differences between HF and TAU
**Housing Stability**					
Mean percentage of days spent stably housed over the 24 month follow-up.	74.8%	71.7%–77.8%	37.9%	34.4%–41.3%	0.001
**Continuous Outcomes (95% CI)**	Mean Change from Baseline to 24 Months	95% CI	Mean Change from Baseline to 24 Months	95% CI	p
Mental illness symptom severity (CSI[a])	-6.3	-7.5 to -5.0	-6.9	-8.4 to -5.4	0.534
Community functioning (MCAS[b])	3.8	2.8 - 4.9	4.8	3.6 - 6.0	0.236
Condition-specific QoL (QoLI-20[c])	16.0	13.6 - 18.4	13.4	10.6 - 16.2	0.178
Psychological community integration (CIS[d])	2.0	1.5 - 2.4	2.0	1.4 - 2.6	0.941

**Count Outcomes**	Rate Ratio Comparing 24 Months with Baseline	95% CI	Rate Ratio Comparing 24 Months with Baseline	95% CI	p
Physical community integration in past month (CIS[d])	0.97	0.86 - 1.08	1.03	0.92 - 1.14	0.439
Severity of substance problems in past month (GAIN-SS[e])	0.79	0.67 - 0.93	0.83	0.70 - 0.98	0.688
Number of emergency department visits in past 6 months	0.38	0.29 - 0.50	0.37	0.27 - 0.51	0.926

Note. Housing outcome is mean proportion of days spent stably housed.

[a] CSI: Colorado Symptom Index. Possible scores range from 14 to 70, with higher values indicating greater symptom severity.

[b] MCAS: Multnomah Community Ability Scale. Possible scores range from 17 to 85, with lower values indicating more disability.

[c] QoLI-20: Lehman Quality of Life Interview 20. Possible scores range from 20 to 140, with higher values indicating greater quality of life.

[d] CIS: Community Integration Scale. Possible scores range from 4 to 20 for the Psychological component and 0-7 for the Physical component, with higher values indicating greater integration.

[e] GAIN-SS: Global Assessment of Individual Needs Short Screener. Possible scores range from 0 to 5 with higher values indicating greater problem severity.

Table 3. Post-Imputation Pooled Comparisons between Housing First and Treatment as Usual at 6, 12, 18 and 24 months for Women in the At Home/Chez Soi Trial Adjusted for Study City, Need Level (ICM or ACT) and Baseline Outcome Values[f]

	Month	Housing First		Treatment as Usual		Comparison		
**Continuous Outcome**		**Mean**	**95% CI**	**Mean**	**95% CI**	**Mean Difference**	**95% CI**	**p**
Mental illness symptom severity (CSI[a])	6	35.8	34.8 - 36.8	36.8	35.6 - 38.0	-1.0	-2.5 to 0.5	0.183
	12	34.6	33.6 - 35.6	35.3	34.1 - 36.6	-0.7	-2.3 to 0.8	0.364
	18	33.8	32.7 - 35.0	34.1	32.7 - 35.4	-0.2	-2.0 to 1.5	0.785
	24	33.7	32.6 - 34.8	33.4	32.1 - 34.7	0.3	-1.4 to 2.0	0.737
Community functioning (MCAS[b])	6	64.9	64.1 - 65.7	64.9	64.0 - 65.9	-0.02	-1.2 to 1.2	0.975
	12	65.0	64.2 - 65.8	64.3	63.4 - 65.3	0.7	-0.6 to 1.9	0.299
	18	65.6	64.8 - 66.5	64.9	63.8 - 65.9	0.8	-0.6 to 2.1	0.267

	24	65.5	64.7 - 66.4	65.9	64.9 - 66.9	-0.4	-1.7 to 0.9	0.593
Condition-specific QoL (QoLI-20[c])	6	84.4	82.4 - 86.4	77.3	75.0 - 79.6	7.1	4.1 - 10.1	<.001
	12	87.4	85.4 - 89.4	81.3	79.0 - 83.7	6.1	3.1 - 9.2	<.001
	18	84.8	82.8 - 86.9	82.2	79.9 - 84.6	2.6	-0.4 to 5.6	0.091
	24	86.8	84.6 - 89.0	84.3	81.8 - 86.7	2.5	-0.7 to 5.8	0.124
Psychological community integration (CIS[d])	6	12.4	12.0 - 12.7	11.8	11.4 - 12.3	0.5	-0.1 to 1.1	0.077
	12	12.8	12.4 - 13.2	12.1	11.6 - 12.5	0.7	0.1 to 1.3	0.017
	18	12.7	12.3 - 13.1	12.3	11.8 - 12.7	0.4	-0.2 to 1.0	0.194
	24	12.9	12.5 - 13.2	12.7	12.2 - 13.1	0.2	-0.4 to 0.8	0.458
**Count Outcome**		**Rate**	**95% CI**	**Rate**	**95% CI**	**Rate Ratio**	**95% CI**	**p**
Physical community integration in past month (CIS[d])	6	2.1	1.9 - 2.3	2.1	1.9 - 2.3	0.98	0.87 - 1.12	0.789

	12	1.9 1.7 - 2.1	2.0 1.8 - 2.2	0.95 0.84 - 1.09	0.492
	18	2.0 1.8 - 2.1	2.0 1.8 - 2.2	0.99 0.87 - 1.12	0.858
	24	1.9 1.7 - 2.0	2.0 1.8 - 2.2	0.91 0.80 - 1.03	0.148
Severity of substance problems in past month (GAIN-SS[e])	6	1.2 1.0 - 1.4	1.2 1.0 - 1.5	0.97 0.78 - 1.20	0.767
	12	1.3 1.1 - 1.5	1.2 1.0 - 1.4	1.09 0.87 - 1.37	0.452
	18	1.1 0.9 - 1.3	1.2 0.9 - 1.4	0.74 1.20 - 0.63	0.628
	24	1.1 0.9 - 1.2	1.0 0.9 - 1.2	1.00 0.79 - 1.27	0.977
Number of emergency department visits in past 6 months	6	1.2 0.9 - 1.4	1.4 1.0 - 1.9	0.80 0.55 - 1.16	0.241
	12	1.1 0.7 - 1.4	1.1 0.7 - 1.6	0.96 0.58 - 1.57	0.858
	18	1.1 0.8 - 1.4	0.6 0.5 - 0.8	1.81 1.26 - 2.60	0.001
	24	0.8 0.6 - 0.9	0.7 0.5 - 0.9	1.13 0.83 - 1.55	0.426

a CSI: Colorado Symptom Index. Possible scores range from 14 to 70, with higher values indicating greater symptom severity.

b MCAS: Multnomah Community Ability Scale. Possible scores range from 17 to 85, with lower values indicating more disability.

c QoLI-20: Lehman Quality of Life Interview 20. Possible scores range from 20 to 140, with higher values indicating greater quality of life.

d CIS: Community Integration Scale. Possible scores range from 4 to 20 for the Psychological component and 0-7 for the Physical component, with higher values indicating greater integration.

e GAIN-SS: Global Assessment of Individual Needs Short Screener. Possible scores range from 0 to 5 with higher values indicating greater problem severity.

f Housing stability is not included in this table as we did not examine it at each follow-up point.

Table 4. Post-Imputation Pooled coefficient estimates (95% Confidence Interval) for Predictors in Linear Mixed Models for Repeated Measures for continuous outcomes and Rate Ratios (95% Confidence Interval) for Generalized Estimating Equations Models for count outcomes for the sample of women in the At Home/Chez Soi study (N=653).

	Mental illness symptom severity (CSI)			Community functioning (MCAS)			Condition-specific QoL (QoLI-20)			Psychological community integration (CIS)		
	Est	95% CI	p-val	Est	95% CI	p-val	Est	95% CI	p-val	Est	95% CI	p-val
Age (years)	-0.01	-0.06- 0.04	0.680	-0.02	-0.060-.02	0.310	-0.06	-0.17- 0.04	0.250	-0.01	-0.03- 0.00	0.140
Less than high school	0.21	-0.98- 1.40	0.731	**-1.32**	**-2.27 to -0.37**	**0.006**	0.87	-1.56- 3.30	0.480	0.15	-0.26- 0.55	0.476
3 or more years of homelessness	0.91	-0.27- 2.09	0.132	**-1.75**	**-2.72 to -0.78**	**<.001**	-0.75	-3.14- 1.64	0540	-0.07	-0.48- 0.33	0.728
Supports ≥1 child under 18 years	0.11	-1.89- 2.10	0.917	0.62	-0.96- 2.20	0.442	0.47	-3.52- 4.47	0.820	0.48	-0.20- 1.17	0.168
Moderate or high suicidality at baseline	**2.85**	**1.59- 4.11**	**<.001**	**-1.16**	**-2.10 to -0.22**	**0.015**	**-3.99**	**-6.49 to -1.49**	**<.001**	-0.30	-0.71- 0.11	0.150
5 or more ACE	**1.50**	**0.25- 2.76**	**0.019**	0.21	-0.75- 1.17	0.666	-0.56	-3.14- 2.02	0.670	0.31	-0.12- 0.73	0.157

	Physical community integration in past month (CIS) (N=653)			Severity of substance problems in past month (GAIN-SS) (N=653)			Number of emergency department visits in past 6 months (N=653)			Stable housing (N=653)		
		95% CI	p-val	RR	95% CI	p-val	RR	95% CI	p-val		95% CI	p-val
Age (years)	1.0	1.0- 1.0	0.834	**0.99**	**0.98-1.00**	**0.003**	1.00	0.99- 1.01	0.920	0.16	-0.04-0.36	0.122
Less than high school	**0.85**	**0.77- 0.93**	**<.001**	**1.27**	**1.06-1.52**	**0.009**	1.11	0.83-1.49	0.474	-2.78	-7.43-1.88	0.242
3 or more years of homelessness	0.98	0.93- 1.11	0.720	**1.21**	**1.03- 1.43**	**0.021**	0.99	0.76-1.29	0.951	**-6.14**	**-10.9--1.32**	**0.013**
Supports ≥1 child under 18 years	1.10	0.96-1.27	0.176	1.05	0.80- 1.39	0.715	0.92	0.67-1.26	0.598	5.57	-2.17-13.3	0.158
Moderate or high suicidality at baseline	1.02	0.93-1.12	0.634	1.06	0.90- 1.27	0.465	**1.44**	**1.10-1.87**	**<0.001**	-1.82	-6.49-2.80	0.440
5 or more ACE	1.04	0.95-1.15	0.356	1.16	0.96- 1.39	0.114	1.05	0.77-1.42	0.772	-2.93	-7.92-2.05	0.249

Each model includes treatment group, categorical time, interaction of treatment x time, city, need level, baseline outcome values and all baseline predictors.

Supporting Information

Figure S1 – Flow of participant enrolment, assignment to treatment arm, and inclusion in analysis

Supplemental Table S1– Missing Rates (%) for Outcomes by Group, Time Period and Overall

Click here to access/download;Figure;Figure 1.docx

Figure 1 – Health and social outcomes among Women who are Homeless and Mentally Ill, unadjusted means and 95% confidence intervals over 24 months of follow-up by study time point and treatment group

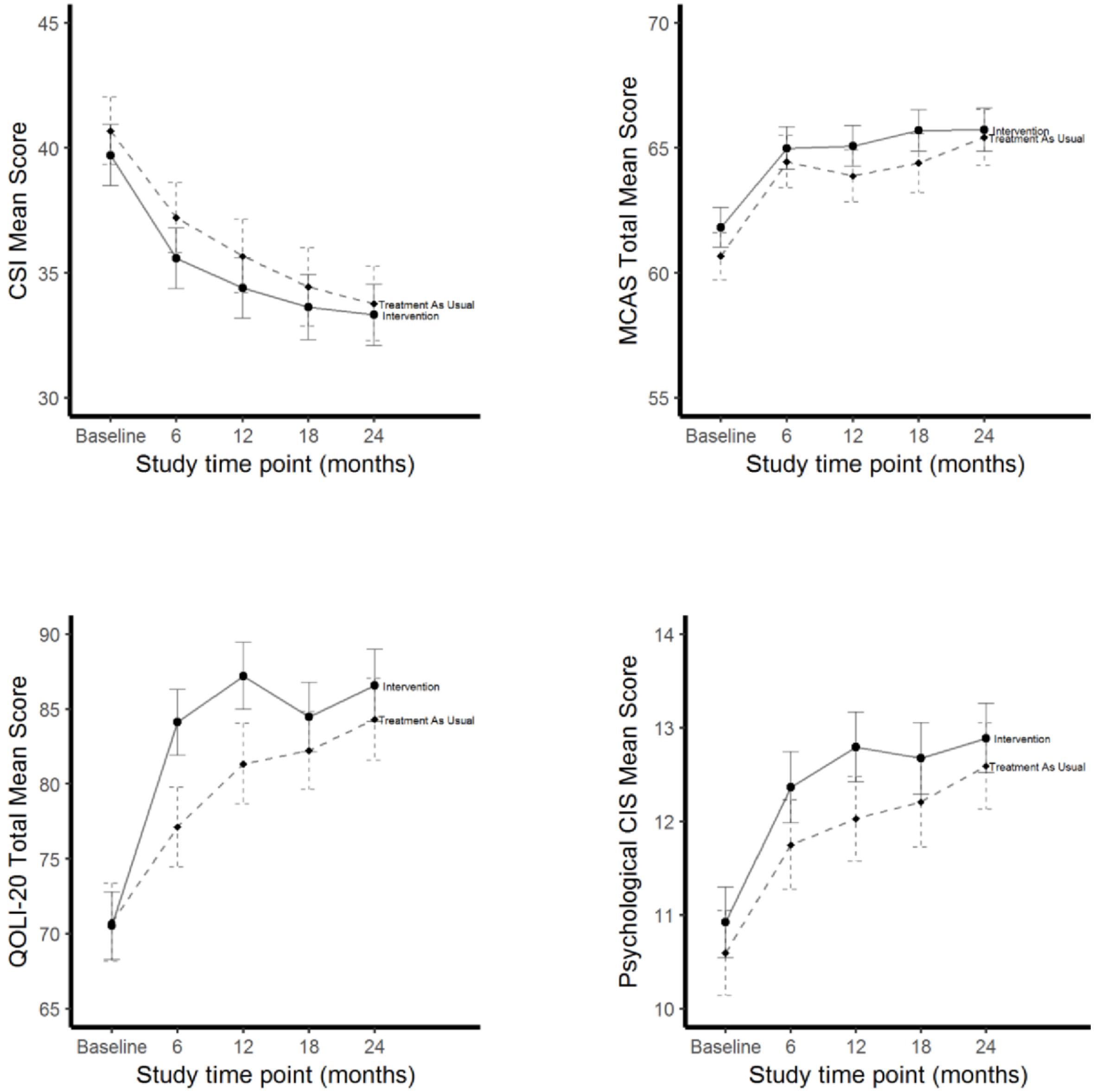

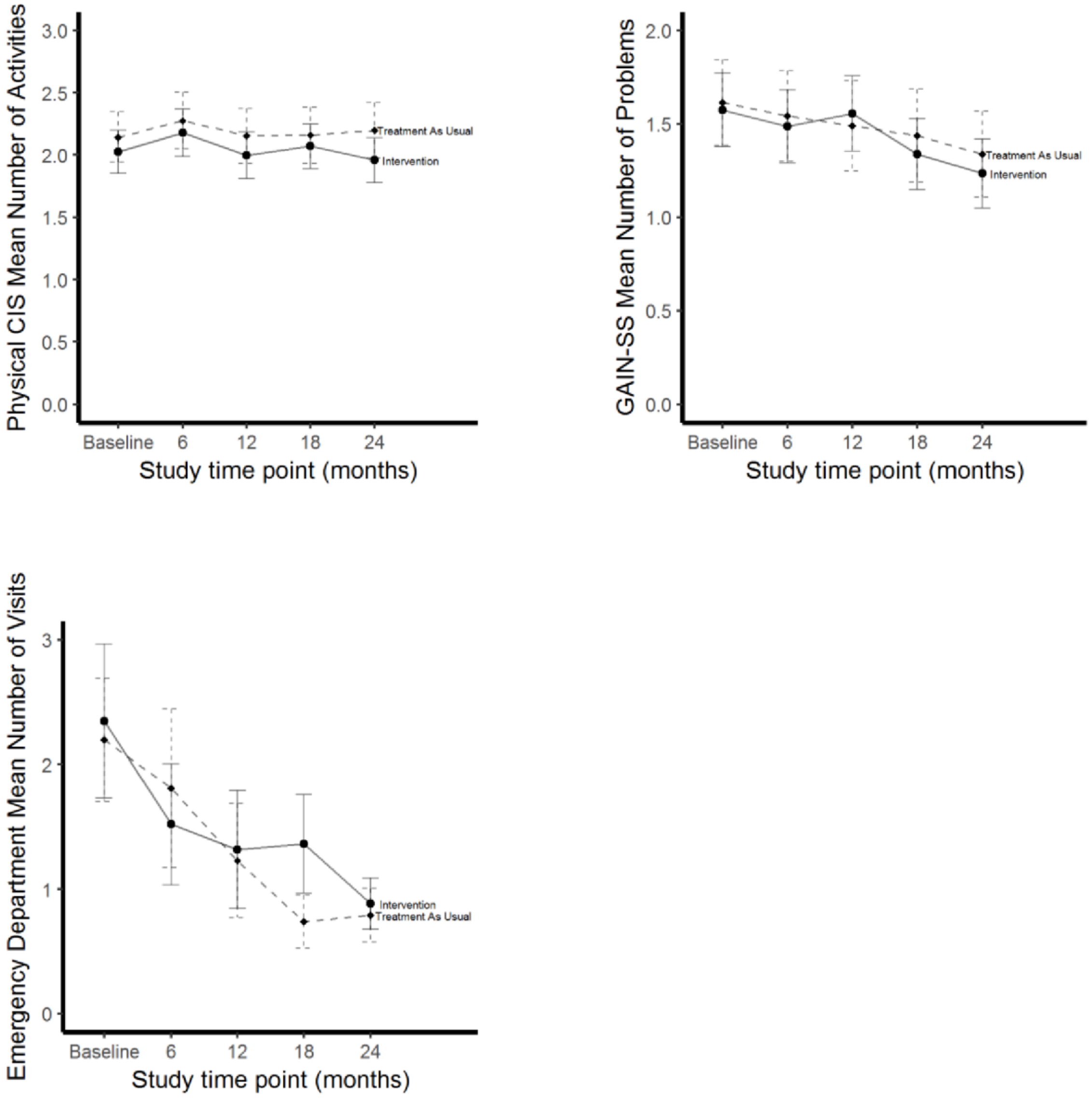

Note. *N* = 653. CSI = Colorado Symptom Index; MCAS = Multnomah Community Ability Scale; Lehman Quality of Life Interview 20 = QoLI-20; CIS = Community Integration Scale; GAIN-SS = Global Assessment of Individual Needs Short Screener.

Click here to access/download
Supporting Information
Supporting Information.docx

Women experiencing homelessness and mental illness in a Housing First multi-site trial: Looking beyond housing to social outcomes and well-being.

O'Campo P., Nisenbaum R., Crocker A. G., Nicholls T., Eiboff F., Adair C.E.

O'Campo, P., Centre for Urban Health Solutions, St Michael's Hospital. Dalla Lana School of Public Health, University of Toronto

Nisenbaum, R., Centre for Urban Health Solutions, Applied Health Research Centre, Li Ka Shing Knowledge Institute, St Michael's Hospital. Dalla Lana School of Public Health, University of Toronto

Crocker A. G., Director, Research & Academics, Institut national de psychiatrie légale Philippe-Pinel & Full professor, Department of Psychiatry & Addictions, and School of Criminology, Université de Montréal

Nicholls, T. L., Department of Psychiatry, University of British Columbia and BC Mental Health and Substance Use Services

Eiboff, F., Interdisciplinary Studies, School of Population and Public Health, University of British Columbia

Adair, C.E., Department of Psychiatry and Department of Community Health Sciences, Cumming School of Medicine, University of Calgary

Abstract (277)

Objective: There is scant research on the effectiveness of permanent supportive housing for homeless women with mental illness. This study examines the effectiveness of Housing First with an unprecedentedly large sample of homeless women from five Canadian cities, and explore baseline risk factors that predict social, health and well-being outcomes over a 24 month-period.

Methods: The At Home/Chez Soi multi-site randomized controlled Housing First trial recruited over 600 women between October 2009 and July 2011. This is a post-hoc subgroup analysis of self-identified women with at least one follow-up interview who were randomized to Housing First (HF) (*n*=374) or treatment-as-usual (TAU) (*n*=279) and had at least one follow-up interview. Linear mixed models and generalized estimating equations were used after multiple imputation was applied to address~~handle~~ missing data.

Results: At the end of follow-up, the mean percentage of days spent stably housed was higher for women in the intervention 74.8% (95%CI=71.7%–77.8%) compared with women in the treatment-as-usual group, 37.9% (95%CI=34.4%–41.3%), *p*<0.001. With few exceptions, social and mental health outcomes were similar for both groups at 6-, 12-, 18- and 24-months post-enrollment. Suicidality was a consistent predictor of increased mental health symptoms (beta=2.85, 95% CI 1.59-4.11, p<0.001), decreased quality of life (beta=-3.99, 95% CI -6.49 to -1.49, p<0.001), decreased community functioning (beta= -1.16, 95% CI -2.10 to -0.22, p=0.015) and more emergency department visits (rate ratio = 1.44, 95% CI 1.10-1.87, p<0.001) over the study period. Lower education was a predictor of lower community functioning (beta=-1.32, 95% CI -2.27 to -0.37, p=0.006) and higher substance use problems (rate ratio=1.27, 95% CI 1.06-1.52, p=0.009) during the study.~~Suicidality was a consistent predictor of mental health symptoms, quality of life, community functioning and emergency department visits over the~~

study period. Lower education was a predictor of lower community functioning and higher substance use problems during the study.

Conclusions: Housing First interventions ensured that women experiencing homelessness areare quickly and consistently stably housed. However, they did not differentially impact health and social measures compared to treatment as usual., yielding similar results for Housing First and treatment as usual groups. Ensuring positive health and social outcomes may require greater supports at enrolment for subgroups such as those with low educational attainment, and additional attention to severity of baseline mental health challenges, such as suicidality.

Key Words: women, homelessness, Housing First, randomized trial, mental health, suicidality, quality of life

Funding: The At Home/Chez Soi demonstration project was funded by Health Canada through the Mental Health Commission of Canada. Trial Registration: International Standard Randomized Control Trial Number Register Identifier: ISRCTN42520374 http://www.controlled-trials.com/ISRCTN42520374. PO was supported by a research chair in Intersectoral Solutions to Urban Health Problems.TN held a CIHR New Investigator award.

Introduction (word count 3476)

Homelessness in Canada is primarily experienced by men, yet data show that over one-third of the homeless people in this country are women and close to 90% of the single parents who are homeless are women [1-3]. Mental health and substance use disproportionately affect women experiencing homelessnessCompared to women in the general population, mental health and substance use problems disproportionately affect women experiencing homelessness.. Between 20-50% experience major depressive disorder and major depressive episodes as demonstrated in systematic reviews [4], between 30-40% experience post-traumatic stress disorder [5,6], as many as one in five report moderate-to-high levels of suicidality [6], and a large proportion experience exceptional vulnerability to an array of health and social risks [6-11], including premature mortality [12]. A recent meta-analysis of observational studies in high-income countries reports that three-quarter of homeless individuals have a mental illness diagnosis with no differences by gender[13].

Women who are homeless have different health and social vulnerabilities compared to homeless men. They have higher rates of distress including suicidal ideation [14], greater experiences of trauma prior to and during episodes of homelessness including more adverse childhood events (ACEs) such as physical and sexual abuse and trauma-related disorders [6,15-22], few options for effective woman-centred services and treatments for these traumatic events and other ailments, all of which also predispose them to homelessness [14,23-27]. They are also substantially more likely than homeless men to engage in survival sex work and are thus more vulnerable to exploitation, violence, infectious disease, gynecological problems and unwanted pregnancies [6,28]. Homeless women with children express considerable traumatic distress because

of frequent child apprehensions [29]. Because women who are homeless often seek shelter in informal settings such as couch surfing or sleeping rough, they are less visible and have less access to services [14,30,31]. and have received less attention in research and policy and practice.

Despite the complex challenges of homelessness among women, P program planners and policy makers are operateing in a context of too little gender-specific data on programs and interventions and weak studies addressing the experiences of womenthis population [3,4,32]. As such, there have been calls for more research that moves away from gender-neutral approaches (i.e., examining men's and women's experiences with the same lens, as if there are no gender differences) [14,32-34] towards more gender-sensitive frameworks, including women-only analyses that capture the variability around the unique challenges women who are homeless face and acceleratinge evidence-informed practice with this population[3,30,35,36]. For interventions, there is a need for studies with large enough samples to examine whether and how programs work for women[4,37-42].

Currently, with some exceptions [43], what we do know about housing interventions for women experiencing homelessness comes from small subsamples in longitudinal observational studies, [35,44] randomized trial pilots, [45] or qualitative evaluations [33,46]. A large multi-site supportive housing demonstration project for chronically homeless adults in 11 US communities, with 173 women as part of the sample, reported substantial improvements in housing stability but no gender differences in this and other service utilization or clinical outcomes over two years of follow-up [47]. In an observational study of an integrated service system initiative in 18 sites across the US, women (N=2727) demonstrated greater improvements in family relationships and social support compared to men (N=4502) over 18-months of follow-up[43].

Housing First, a supportive housing approach with demonstrated success in transitioning individuals experiencing chronic homelessness and mental illness into permanent housing, has negligible evidence on outcomes for women [48-51]. The Housing First model is based on harm reduction principles of choice and self-determination, and provides immediate access to scattered-site housing through the provision of rent supplements and flexible recovery-oriented supports [50,52]. Participation in Housing First is not contingent on participation in services or requirements of 'housing readiness' (e.g., sobriety or medication compliance) [50]. The At Home/Chez Soi randomized trial conducted in five cities across Canada provides an unprecedented opportunity to examine the impact of a housing first initiative among women for several reasons. First, as the largest randomized trial of a Housing First initiative and includes an unprecedented sample of several hundred women. This large sample size facilitates a woman only analysis to focus on variability within this subsample and factors that are relevant for women living with homelessness such as sources of trauma or parental roles. A woman only analysis, too rarely seen in studies of homelessness, ensures that the focus remains on women within the sample as gender analyses largely focus on comparing experiences with those of men. With 24 months of follow-up and novel risk variables not previously examined in a Housing First analysis we can provide unique point-in-time and longitudinal evidence not previously considered because of prior sample size and variable availability limitations.

~~Supportive housing interventions for homeless individuals with mental illness examining gender have reported positive housing stability outcomes with few gender differences. In a study of an integrated service delivery system policy initiative in 18 sites across the US, women (N=2727) and men (N=4502) showed similar improvements in days stably housed over 18-months and women showed better outcomes in social and victimization measures, although~~

women with children demonstrated significantly less improvement in substance use . A large multi-site supportive housing demonstration project for chronically homeless adults with medical, psychiatric, and/or substance use problems in 11 US communities reported no gender differences over two years across a range of outcomes . Moreover, while trauma, mental health and ACEs are important predictors of homelessness, only the At Home/Chez Soi intervention study examined the impact of ACEs on housing and health outcomes but did not examine the intervention impacts of ACEs and gender .

The present study employs the largest longitudinal sample of women experiencing homelessness and mental illness in a Housing First randomized controlled trial (RCT) and extends the follow up duration typically seen to 24 months. Our objectives were: (i) To compare change at the end of the study in health and social outcomes, between women who received and did not receive the Housing First intervention; ii) To examine whether the Housing First intervention leads to better housing stability over 24 months, and health and social and outcomes between 6- to 24-months post enrolment; and, (iii) To explore baseline demographic, social, health measures, and ACEs as predictors of key study outcomes during the study period.

Methods

Methods and measures for this analysis have been extensively described elsewhere [49,53-55]. Briefly, the Canadian At Home/Chez Soi study was conducted in five cities and is the largest five city multi-site RCT randomized to examine Housing First amongfor adults experiencing homelessness and serious mental illness [54]. Participants randomized to the Housing First (HF) intervention received scattered-site housing and either moderate or high needs-based services to support their recovery and integration into the community (i.e., Intensive Case Management or

Assertive Community Treatment). Participants randomized to treatment-as-usual (TAU) had access to housing and services through other community programs. Each site had additional focus on a unique population (e.g., ethno-racial populations in Toronto and Indigenous populations in Winnipeg) and had specialized services to address their needs. Fidelity assessments ensured strong adherence to program theory of change[56]. Information about the study, such as the randomization process and a description of the intervention, was provided to a~~A~~ll study participants prior to enrolment. An informed consent process was created to ensure that participants had the time and information to understand the complexities of the trial and follow-up activities. All participants provided written informed consent prior to enrolment. All enrolment and consent documents were harmonized across sites, however, each site had separate ~~and Ethics Board~~ approval ~~was received~~ from ~~each of the~~ relevant university/site Research Ethics Board~~REB~~s.

Over 24 months, participants were interviewed every 3 months to assess housing stability and every 6 months to evaluate health and social outcomes. Previous r~~R~~esults from 2,148 participants (including 683 women) demonstrated that those in the HF arm showed significant improvements in housing stability, quality of life, and community functioning compared to participants in usual care [47,49,53]. This paper reports on ~~is~~ a subgroup analysis of 653 participants who self-identified as women and had at least one follow-up after their baseline assessment (96%), including 588 followed to 24 months. Across all sites, 374 participants were from the HF group and 279 from the TAU group (Supplemental Figure S1).

Outcomes and other study variables

We describe in this section the outcome variables and any other scales used in this analysis. Stable housing was defined as “living in one’s own room, apartment, or house, or with

family, with an expected duration of residence of 6 months or more or tenancy rights" while enrolled in the study, taking into account participants' move in/out dates for each type of residence (e.g., street place, unstable residence, stable residence, institution)[57]. The *percentage of days stably housed during the 24-month period* was calculated as the total number of days stably housed divided by the total number of days for which any type of residence data were provided by the participant over 24 months [49,54]). Unlike the other outcomes, housing stability was measured at the end of the follow-up period for several reasons. First, the treatment group establishes stable housing within the first 1-3 months of enrolment and housing status rarely changes after that point in time. A small proportion of the treatment group was not consistently stably housed throughout the duration of the program; thus, this single cumulative measure captures housing stability better than if we had measured it at each follow-up time point. In sum, this cumulative housing stability is the best measure for the outcome of housing as it does not demonstrate incremental changes over the 24-month follow-up period.

The other outcomes were assessed via in-person interviews at baseline, 6-, 12-, 18, and 24 months post-enrollment.

The Lehman Quality of Life Interview 20 (QoLI-20) measured participants' self-reported life satisfaction on a 7-point Likert scale in the domains of family, finance, leisure, living, safety, social, and overall quality of life. Scores range from 7 to 140, with higher scores indicating better quality of life [58].

Community functioning was measured using the Multnomah Community Ability Scale (MCAS), a 17-item scale capturing degree of clinician/observer-ratedself-reported functional ability in health, adaptation, social skills, and behavior, with higher scores indicating less impairment.

Formatted: English (United States)
Formatted: English (United States)
Formatted: English (United States)
Formatted: Font: (Default) Times New Roman, 12 pt, Font color: Black
Formatted: Font: (Default) Times New Roman, 12 pt, Font color: Black
Formatted: Font: (Default) Times New Roman, 12 pt, Font color: Black
Formatted: Font: (Default) Times New Roman, 12 pt, Font color: Black
Formatted: Font: (Default) Times New Roman, 12 pt, Font color: Black
Formatted: Font: (Default) Times New Roman, 12 pt, Font color: Black
Formatted: Font: (Default) Times New Roman, 12 pt, Font color: Black

The Colorado Symptom Index (CSI) assessed how often specific psychiatric symptoms were experienced, from 'at least every day' to 'not at all'. Fourteen self-report items were summed (range 14 to 40), with lower scores reflecting fewer symptoms [59,60].

Emergency department (ED) visits were measured using self-reported counts in the past 6 months. Past-month substance-related problems were measured via the 5-item Global Assessment of Individual Needs Short Screener (GAIN-SS); higher values correspond to greater problem severity [54].

The Community Integration Scale (CIS) measured self-reported psychological integration with the immediate community on a scale from 4-20; physical integration used counts of previous month social activities (e.g., attending a concert, meeting people for coffee) on a scale of 0-7. Higher scores on both reflect greater integration [61].

We collected data using the Adverse Childhood Experiences module encompassing ten early childhood adversities such as emotional or sexual abuse, neglect, household criminal justice involvement and parental separation or divorce [62]. We created two categories representing high levels of reported ACEs, ≥5 adversities, and four or fewer ACEs including none. Levels of suicidality was assessed via the MINI Neuropsychiatric Interview 6.0 administered as part of the baseline enrolment interview[63]. Information about all other variables (e.g., demographics, financial support of children, sources of income) was obtained via self-report during the in-person interviews.

Statistical Analyses

Descriptive statistics for a wide range of descriptors were selected to characterize the sample by treatment group. Longitudinal outcomes were analyzed using linear mixed analysis of repeated measures models for continuous outcomes and generalized estimating equations for

Formatted: Indent: First line: 0.5"

count outcomes (Poisson distribution for CIS-Physical Integration and negative binomial distribution for GAIN-SS and ED Visits), assuming an unstructured correlation matrix for the repeated measures.

For the first objective, for each group, the adjusted change from baseline to 24-months follow-up was estimated by mean differences and 95% confidence intervals (continuous outcomes), and rate ratios and 95% confidence intervals (count outcomes). We fit models that included treatment group (fixed effect, HF vs TAU), categorical time (24-months vs baseline), interaction of treatment x time, and study city and need level at baseline (high vs. moderate) as covariates.

For the second objective, in a more detailed examination of possible differential effect over time, we tested for differences between HF and TAU at each follow-up time point from 6- to 24-months, adjusting for baseline outcomes. Models included treatment group, categorical time (6-, 12-, 18-, 24-months), interaction of treatment x time, and city, need level and baseline outcome values as covariates. At each time point, we compared HF vs TAU by estimating mean differences and 95% confidence intervals or rate ratios and 95% confidence intervals. We modeled the total percentage of days stably housed over 24-months as a continuous outcome and fitted a linear regression model including treatment group, and city, need level and percentage of days stably housed in the three months prior to baseline as covariates

For the third objective, the differences between HF and TAU were no longer the focus. Rather we took advantage of the large sample of women in a study on homelessness to identify predictors of our outcomes, uncoupling the participants from their assigned trial arm. To accomplish this we expanded the models from the second objective and added the following

Formatted: Indent: First line: 0.5"

Formatted: No underline

baseline predictors: age at enrolment (continuous), education (1=less than high school, 0=high school or higher), duration of homelessness (1=≥3 ,0= <3 years), whether minor children were being supported by the participant (1=yes, 0=no), level of suicidality (1=moderate or high, 0=mild or none), and high level of ACEs at baseline (1=≥5 , 0=<5 ACEs). less than high school education, ≥3 years homeless, whether minor children were being supported by the participant, moderate or high level of suicidality, and high level of ACEs at baseline (≥5 ACEs). We were unable to include ethnicity or Indigenous identity due to multicollinearity with study city. These predictors were chosen because they represent a knowledge gap or have yielded inconsistent findings on longitudinal studies of women experiencing homelessness. We were unable to include ethnicity or Indigenous identity due to multicollinearity with study city.

Formatted: Indent: First line: 0.5"

Multiple imputation with chained equations (MICE) was used to handle missing data from loss to follow-up, withdrawal, skipped interviews, nonresponse on specific items, and systematically measured lack of interviewer confidence in participant responses. We imputed all outcomes simultaneously and MICE imputation models included all variables needed to address three objectives[64] . Although outcome missing rates varied between 0.53% to 31.9% (Supplemental Table S1), approximately 70% of the participants had at least one missing value in at least one outcome in at least one point in time. Therefore, we chose 70 imputations following guidelines given by White et al. (2010) that the percentage of cases with incomplete data should be similar to the number of imputations generated [64]. Comparisons between observed and imputed data via diagnostic plots were used to assess the quality and plausibility of the imputations[65]

Data were imputed using Stata software (mi impute chained, Stata Statistical Software: Release 13. College Station, TX: StataCorp LP) and analyzed using SAS (PROC MIXED, GENMOD , PROC MIANALYZE, SAS 9.4, SAS Institute Inc, Cary, NC) Figures were created using the ggplot2 package in R software version 3.4.0 (R Core Team (2017) [66]. All statistical tests were two-tailed and significance was defined as $p<.05$. No adjustments for multiple testing were applied [67].

Formatted: Indent: First line: 0.5"

Results

We took advantage of the rich data collected in this study and present a broad array of pParticipant demographic, social, and health characteristics at enrolment are presented in (Table 1).

Fifteen percent of the women were born outside of Canada (median time in Canada: TAU=25yrs; HF = 21yrs). Three percent of women were employed at baseline and 90% received social assistance of some type as a source of income. Around 40% (*n*=274) reported having minor children, and of these, 22% (*n*=60) were providing full or partial financial support to the child(ren). The most prevalent mental illnesses were depressive episodes, PTSD, and alcohol or substance dependence. Approximately 40% had moderate-to-high suicidality, around 40% had post-traumatic stress disorder, about 60% reported ≥5 ACEs, and the majority had three or more co-morbid medical conditions.

Table 2 displays results from models examining changes from baseline to 24 months for those in HF and TAU arms for each of our social, well-being and, and comparing groups with respect to housing stability over the 24-month period. Over the 24-month follow-up period,

substantial improvements were demonstrated for all the outcomes for both HF and TAU participants. The mean percentage of days spent stably housed during follow-up for women receiving HF was 74.8% (95%CI=71.7%–77.8%) compared with 37.9% (95%CI=34.4%–41.3%) TAU, a difference of 36.9% (95%CI=32.4%–41.4%, $p<0.001$). Of note, mean total QoLI-20 scores improved 16.0 points (95%CI=13.6–18.4) and 13.4 points (95%CI=10.6–16.2) in the HF and TAU groups, respectively, and number of ED visits decreased by approximately 60% in both groups.

These improvements over the 24-month period are more clearly illustrated in Figure 1 for illustrates the HF intervention and TAU groups profiles for all social and health unadjusted outcomes at *each observation point* from baseline to 24-months. For all outcomes, except CIS-Physical where scores remained flat throughout the follow-up, both groups experienced similar significant change from baseline to 24-months (Figure 1).

Table 3 -displays changes over time in greater detail through results of the for models to assessing whether social and health outcomes were better in HF vs. TAU groups *at each follow-up point*-. For condition-specific quality of life, significant improvements of 7.1 and 6.1 points were observed in the HF group at the 6- and 12-month follow-ups, respectively. The HF group also showed significant improvements in psychological community integration by 0.7 points at the 6-month follow-up. However, in the last year of the trial, the ED visits fell faster for the TAU group (0.6 visits) compared to the HF group (1.1 visits) -at 18 months, with a rate ratio of 1.81 (95%CI=1.26–2.60).

Table 4 no longer focuses on differences between HF and TAU as results presented here uncouple participants from their assigned treatment arms and identify predictors of our outcomes. Mshows that moderate to high suicidality had the most consistent association to

poorer health and service utilization outcomes at 24 months follow-up. Compared to those with no or low suicidality at baseline, moderate-to-high suicidality at baseline was associated with nearly three points on the CSI indicating greater mental health symptom severity (beta=2.85, 95%CI=1.59–4.11, p<0.001), QoLI-20 mean scores were~~was~~ ~~reduced by~~ almost four points lower indicating reduced quality of life (beta=-3.99, 95%CI=-6.49 to -1.49, p=0.002), and almost 1.5 more ED visits (rate ratio=1.44, 95%CI=1.10–1.87, p=0.007). Low education was also associated with several outcomes. Compared to those with higher education, women with~~:~~ less than a high school level of education at baseline predicted lower community functioning (MCAS) scores (beta=-1.32, 95%CI=-2.27 to -0.37, p=0.006), ~~those with less than high school education had~~ 15% fewer social activities (CIS) in the previous month (rate ratio=0.85, 95%CI=0.77–0.93, p<0.001)~~ than those with higher levels of education~~, and more severe ~~low education predicted higher~~ substance-related problems at 24 months follow-up (rate ratio=1.27, 95%CI=1.06–1.52, p=0.009). Having been homeless for ≥3 years at baseline was associated with lower percentage days stably housed at 24-months (beta=-6.1%, 95%CI=-11.0% to -1.3%, p=0.013). An ACE score of 5 or more was associated with a 1.5 point increase in mental illness symptom severity (95%CI=0.25–2.76, p=0.019). Finally, we explored and found no evidence that baseline levels of ACEs and suicidality, separately, were effect modifiers for any of the above analyses .

Discussion

This is the first analysis of women's outcomes in an RCT of Housing First. Prior studies and trials on housing interventions for people who experience homelessness often included very small numbers of ~~women which~~women or few variables reflecting women's unique needs and

experiences that have precluded documentation of the heterogeneity of experiences and in-depth understanding of this population.

In this Canadian multi-site study, the largest longitudinal sample of women experiencing homelessness to date, the intervention group experienced housing stability for 75% of the 24-month follow-up, as compared to ~~just over~~ 38~~0~~% for women in the TAU group, a finding similar to previously published reports of At Home/Chez Soi participants, the majority of whom were men [49,53]. Even if ~~However,~~ the large proportion of time spent stably housed for women who received Housing First~~HF~~ compared to the TAU group ~~over the two-year follow-up~~ did not translate into greater improvements in health and social outcomes by the end of the study, there were some early differences favoring the treatment group in the first year for quality of life and psychological community integration. Immediate placement in housing and early housing stability for HF compared to TAU [68]participants likely contributed to faster improvements in these outcomes. ~~.~~

While ED visits declined in a similar fashion for HF and TAU over the 24-month follow-up, an unanticipated finding was a significant reduction in ED visits of 0.5 visits at 18 months for the TAU group over the HF group. For this rare count outcome, the dip in ED visits for TAU at 18 months could be a chance improvement as ~~More data are needed to determine why the drop in the TAU group surpassed reductions in the HF arm although~~ both groups had similar levels of ED visits by 24 months. Analyses of the full At Home/Chez soi trial data did not show differences for this outcome [49]. One possibility is that uncertainty around continued housing as the trial was ending may have created high levels of stress in the Housing First group, but if this were the case, we would have likely seen similar changes~~jumps~~ in other study outcomes or among men in the analyses of the full sample.

Since improvements in outcomes over the 24 month24-month period were similar for both the HF and TAU groups, we moved beyond the intention-to-treat analysis and examined baseline predictors for our outcomes across the full sample. We focused on baseline suicidality, ACEs, and parental responsibilities as predictors- variables rarely examined in past studies of supported housing or Housing First [35,43-45,69,70]. As might be expected, women with >5 high ACE scores or moderate-to-high levels of suicidality at baseline showed poorer improvement by 24 months compared to those with low ACE or suicidality scores. Moderate-to-high suicidality was a strong predictor of poorer outcomes at 24 months, including mental health, quality of life, community integration, and ED visits. Past cross-sectional studies of combined samples of homelessness men and women have demonstrated similar links between suicidality, childhood and adult trauma, substance abuse, and mental health [20,21,71]. However, while cross-sectional associations we found no association between suicidality and substance among women who are homeless have been reported in the literatureuse in our longitudinal analysis which differs from Benda et al., (2005) [19,72] our study is the first to examine these associations longitudinally. who reported an odds ratio of 1.20 for the association between drug abuse and contemplation of suicide in a cross sectional analysis of 310 women veterans.

Contrary to what we might have anticipated on the importance of ACEs in predictingon multiple study outcomes based upon prior demonstrating the association of ACEs with victimization and criminal justice given past researchinvolvement [73], we found that high levels of ACEs only predicted significantly worse scores on mental illness severity (CSI). This is consistent with a recent study of the full sample of At Home/Chez Soi where ACEs total scores predicted several mental health conditions at 24-months [51]. Our choice of a cutoff representing high numbers of ACEs, while informed by existing literature, may have limited our ability to

detect an association. Alternatively, ACEs may be limited in capturing relevant adverse conditions. A recent study has suggested that an expanded ACEs tool might be used to capture important domains currently missing such as severe poverty or family loss and separation[74], which might be particularly relevant in capturing sources of trauma and adversity for women experiencing homelessness. in our study high baseline ACE scores only predicted significantly worse scores on mental illness severity (CSI). Providing economic support for dependent children was not related to any of our study outcomes. Our findings also demonstrated that low educational attainment at baseline was a significant predictor of worse mental health, community integration, and substance use outcomes independently of the other variables in the model. Low educational attainment may be a proxy for other factors such as low health literacy which might impact management of health conditions and requires further investigation [75]. Providing economic support for dependent children was not related to any of our study outcomes.

Although While the the At Home/Chez Soi support services were important for intervention participants to remain stably housed and make gains in health and social standing, these supports and services were not designed in this Housing First program to be tailored to women's unique needs. Psychological housing stability is particularly important for women and which includes considerations of safety, security and feelings of home, is particularly important for women while yet the At Home/Chez Soi focused predominantly on material housing stability[76]. Women who are unstably housed or homeless need services that address trauma and promote informal peer support networks [33,77]. Although staff were trained to be responsive to individual needs, the At Home/Chez Soi intervention was not specifically designed to provide the latter two resources. In addition, more than 40% of our participants were homeless for more than three years at enrolment. Two years of stable housing with mental health supports may not

have been sufficient to resolve chronic distress from being homeless with co-morbid health, trauma and substance use problems, particularly given the high rates of reported PTSD and ACEs[14].

The lack of greater improvements in the HF arm in this analysis might have been due to the intensive follow-up schedule that could have led to a Hawthorne effect among the TAU group, and higher loss to follow-up in the TAU group might have contributed to smaller differences between groups at 24 months. Despite our sample of homeless women being relatively large, it may not have been sufficient to detect small effects. The At Home/Chez Soi study did not capture gender-sensitive outcomes specific to women experiencing homelessness and therefore we may have missed important changes, such as those associated with positive social support, increased safety from crime and victimization, or strengthening of relationships with children, family or friends[3,78].

Our study adds to the growing yet sparse literature on the characteristics and needs of women experiencing homelessness and the effectiveness of interventions to eliminate homelessness. Given that women make up more than one third of the homeless population, and their needs differ from that of men[3,14,27,32], it is important that their experiences with homelessness and recovery receive ample focus to better inform the design and implementation of interventions that meet their unique needs. Moreover, the homelessness epidemic has only worsened in the context of the COVID pandemic so the issues remain relevant and these new analyses can provide insights[79].

Formatted: Font: (Default) Times New Roman, 12 pt, Font color: Black, English (United States)

Formatted: Font: (Default) Times New Roman, 12 pt, Font color: Black, English (United States)

References

1. Gaetz S, Dej E, Richter T, Redman M. The State of Homelessness in Canada 2016. Toronto2016.
2. Employment and Social Development Canada. Everyone Counts 2018: Highlights - Preliminary results from the second nationally coordinated Point-in-Time count of homelessness in Canadian communities. 2019.
3. Schwan K, Versteegh A, Perri M, et al. The State of Women’s Housing Need & Homelessness in Canada: A Literature Review. Hache, A., Nelson, A., Kratochvil, E., & Malenfant, J. (Eds). Toronto, ON: Canadian Observatory on Homelessness Press. 2020.
4. Duke A, Searby A. Mental Ill Health in Homeless Women: A Review. Issues Ment Health Nurs 2019;40:605-12.
5. Ferguson KM, Bender K, Thompson SJ. Gender, coping strategies, homelessness stressors, and income generation among homeless young adults in three cities. Social science & medicine (1982) 2015;135:47-55.
6. Strehlau V, Torchalla I, Kathy L, Schuetz C, Krausz M. Mental health, concurrent disorders, and health care utilization in homeless women. Journal of psychiatric practice 2012;18:349-60.
7. Bassuk EL, Buckner JC, Perloff JN, Bassuk SS. Prevalence of mental health and substance use disorders among homeless and low-income housed mothers. The American journal of psychiatry 1998;155:1561-4.
8. Shinn M, Knickman JR, Weitzman BC. Social relationships and vulnerability to becoming homeless among poor families. The American psychologist 1991;46:1180-7.
9. Banyard VL, Graham-Bermann SA. Surviving poverty: stress and coping in the lives of housed and homeless mothers. The American journal of orthopsychiatry 1998;68:479-89.
10. Nelson G, Patterson M, Kirst M, et al. Life changes among homeless persons with mental illness: a longitudinal study of housing first and usual treatment. Psychiatric services (Washington, DC) 2015;66:592-7.
11. Riley ED, Weiser SD, Sorensen JL, Dilworth S, Cohen J, Neilands TB. Housing Patterns and Correlates of Homelessness Differ by Gender among Individuals Using San Francisco Free Food Programs. Journal of Urban Health 2007;84:415-22.
12. Cheung AM, Hwang SW. Risk of death among homeless women: a cohort study and review of the literature. CMAJ : Canadian Medical Association journal = journal de l'Association medicale canadienne 2004;170:1243-7.
13. Gutwinski S, Schreiter S, Deutscher K, Fazel S. The prevalence of mental disorders among homeless people in high-income countries: An updated systematic review and meta-regression analysis. PLoS Med 2021;18:e1003750.
14. Milaney K, Williams N, Lockerbie SL, Dutton DJ, Hyshka E. Recognizing and responding to women experiencing homelessness with gendered and trauma-informed care. Bmc Public Health 2020;20.
15. Brunette M, Drake RE. Gender differences in homeless persons with schizophrenia and substance abuse. Community mental health journal 1998;34:627-42.
16. Rich AR, Clark C. Gender differences in response to homelessness services. Evaluation and Program Planning 2005;28:69-81.
17. Roll CN, Toro PA, GL. O. Characteristics and experiences of homeless adults: A comparison of single men, single women, and women with children. Journal of Community Psychology 1999;27:189-98.
18. Torchalla I, Strehlau V, Li K, Aube Linden I, Noel F, Krausz M. Posttraumatic stress disorder and substance use disorder comorbidity in homeless adults: Prevalence, correlates, and sex differences. Psychology of addictive behaviors : journal of the Society of Psychologists in Addictive Behaviors 2014;28:443-52.

19. Benda BB. Gender differences in predictors of suicidal thoughts and attempts among homeless veterans that abuse substances. Suicide & life-threatening behavior 2005;35:106-16.
20. Desai RA, Liu-Mares W, Dausey DJ, Rosenheck RA. Suicidal ideation and suicide attempts in a sample of homeless people with mental illness. The Journal of nervous and mental disease 2003;191:365-71.
21. Eynan R, Langley J, Tolomiczenko G, et al. The association between homelessness and suicidal ideation and behaviors: results of a cross-sectional survey. Suicide & life-threatening behavior 2002;32:418-27.
22. Rodriguez-Moreno S, Vázquez JJ, Roca P, Panadero S. Differences in stressful life events between men and women experiencing homelessness. Journal of Community Psychology 2021;49:375-89.
23. Herman DB, Susser ES, Struening EL, Link BL. Adverse childhood experiences: are they risk factors for adult homelessness? American journal of public health 1997;87:249-55.
24. Montgomery AE, Cutuli JJ, Evans-Chase M, Treglia D, Culhane DP. Relationship among adverse childhood experiences, history of active military service, and adult outcomes: homelessness, mental health, and physical health. American journal of public health 2013;103 Suppl 2:S262-8.
25. Roos LE, Mota N, Afifi TO, Katz LY, Distasio J, Sareen J. Relationship between adverse childhood experiences and homelessness and the impact of axis I and II disorders. American journal of public health 2013;103 Suppl 2:S275-81.
26. Patterson ML, Moniruzzaman A, Somers JM. Setting the stage for chronic health problems: cumulative childhood adversity among homeless adults with mental illness in Vancouver, British Columbia. BMC Public Health 2014;14:350.
27. Schwan K, Vaccaro M, Reid L, Ali N, Baig K. The Pan-Canadian Women's Housing & Homelessness Survey. Toronto, ON: Canadian Observatory on Homelessness; 2021.
28. Milaney K, Williams N, Lockerbie SL, Dutton DJ, Hyshka E. Recognizing and responding to women experiencing homelessness with gendered and trauma-informed care. BMC Public Health 2020;20:397.
29. Torchalla I, Linden IA, Strehlau V, Neilson EK, Krausz M. "Like a lots happened with my whole childhood": violence, trauma, and addiction in pregnant and postpartum women from Vancouver's Downtown Eastside. Harm Reduction Journal 2015;12:1.
30. Shier ML, Jones ME, Graham JR. Sociocultural Factors to Consider When Addressing the Vulnerability of Social Service Users: Insights From Women Experiencing Homelessness. Affilia 2011;26:367-81.
31. Winetrobe H, Wenzel S, Rhoades H, Henwood B, Rice E, Harris T. Differences in Health and Social Support between Homeless Men and Women Entering Permanent Supportive Housing. Women's Health Issues 2017;27:286-93.
32. Perri M, O'Campo P. A gap in knowledge surrounding urban housing interventions: a call for gender redistribution. Health Promot Int 2021;36:908-12.
33. Oudshoorn A, Forchuk C, Hall J, Smith-Carrier T, Van Berkum A. An evaluation of a Housing First program for chronically homeless women. Journal of Social Inclusion 2018;9:34-50.
34. Perri M, Metheny N, Matheson FI, Potvin K, O'Campo P. Finding opportunity in the COVID-19 crisis: prioritizing gender in the design of social protection policies. Health Promot Int 2022;37.
35. Cameron A, Abrahams H, Morgan K, Williamson E, Henry L. From pillar to post: homeless women's experiences of social care. Health Soc Care Community 2016;24:345-32.
36. Fotheringham S, Walsh CA, Burrowes A. 'A place to rest': the role of transitional housing in ending homelessness for women in Calgary, Canada. Gender, Place & Culture 2014;21:834-53.
37. Blehar MC. Public health context of women's mental health research. The Psychiatric clinics of North America 2003;26:781-99.

38. Institute of Medicine. Sex-Specific Reporting of Scientific Research: A Workshop Summary. Washington, DC: The National Academies Press. https://doi.org/10.17226/13307.2012.
39. Jahn I, Bornhorst C, Gunther F, Brand T. Examples of sex/gender sensitivity in epidemiological research: results of an evaluation of original articles published in JECH 2006-2014. Health research policy and systems 2017;15:11.
40. Klap R, Humphreys K. Designing Studies for Sex and Gender Analyses: How Research Can Derive Clinically Useful Knowledge for Women's Health. Women's health issues : official publication of the Jacobs Institute of Women's Health 2019;29 Suppl 1:S12-s4.
41. Welch V, Doull M, Yoganathan M, et al. Reporting of sex and gender in randomized controlled trials in Canada: a cross-sectional methods study. Res Integr Peer Rev 2017;2:15-.
42. Tsai J, Byrne TH. National Utilization Patterns of Veterans Affairs Homelessness Programs in the Era of Housing First. Psychiatric Services 2019;70:309-15.
43. Cheng AL, Kelly PJ. Impact of an integrated service system on client outcomes by gender in a national sample of a mentally ill homeless population. Gender medicine 2008;5:395-404.
44. Nesse L, Gonzalez Marianne T, Aamodt G, Raanaas Ruth K. Recovery, quality of life and issues in supported housing among residents with co-occurring problems: a cross-sectional study. Advances in Dual Diagnosis 2020;13:73-87.
45. Tinland A, Zemmour K, Auquier P, et al. Homeless women with schizophrenia reported lower adherence to their medication than men: results from the French Housing First experience. Social Psychiatry and Psychiatric Epidemiology 2017;52:1113-22.
46. Adame AL, Perry C, Pierce E. Community and Housing First: A qualitative analysis of USA residents' perspectives. Health Soc Care Community 2020;28:1292-300.
47. Edens EL, Mares AS, Rosenheck RA. Chronically homeless women report high rates of substance use problems equivalent to chronically homeless men. Women's health issues : official publication of the Jacobs Institute of Women's Health 2011;21:383-9.
48. Aubry T, Tsemberis S, Adair CE, et al. One-Year Outcomes of a Randomized Controlled Trial of Housing First With ACT in Five Canadian Cities. Psychiatric Services 2015;66:463-9.
49. Stergiopoulos V, Hwang SW, Gozdzik A, et al. Effect of scattered-site housing using rent supplements and intensive case management on housing stability among homeless adults with mental illness: a randomized trial. Jama 2015;313:905-15.
50. Tsemberis S, Gulcur L, Nakae M. Housing First, consumer choice, and harm reduction for homeless individuals with a dual diagnosis. American journal of public health 2004;94:651-6.
51. Liu M, Mejia-Lancheros C, Lachaud J, et al. Overall and Gender-specific Associations between Dimensions of Adverse Childhood Experiences and Mental Health Outcomes among Homeless Adults: Associations Générales et Sexospécifiques Entre les Dimensions des Expériences Défavorables de L'enfance et les Résultats de Santé Mentale Chez les Adultes Sans Abri. Can J Psychiatry 2021;66:906-17.
52. Paquette K, Pannella Winn LA. The Role of Recovery Housing: Prioritizing Choice in Homeless Services. Journal of dual diagnosis 2016;12:153-62.
53. Aubry T, Goering P, Veldhuizen S, et al. A Multiple-City RCT of Housing First With Assertive Community Treatment for Homeless Canadians With Serious Mental Illness. Psychiatric services (Washington, DC) 2016;67:275-81.
54. Goering PN, Streiner DL, Adair C, et al. The At Home/Chez Soi trial protocol: a pragmatic, multi-site, randomised controlled trial of a Housing First intervention for homeless individuals with mental illness in five Canadian cities. BMJ open 2011;1:e000323.
55. Aquin JP, Roos LE, Distasio J, et al. Effect of Housing First on Suicidal Behaviour: A Randomised Controlled Trial of Homeless Adults with Mental Disorders. Can J Psychiatry 2017;62:473-81.

56. Nelson G, Macnaughton E, Caplan R, et al. Follow-up Implementation and fidelity Evaluation of the mental Health Commission of Canada's At Home/Chez Soi Project: Cross-site Report: Mental Health Commission of Canada; 2013.
57. Tsemberis S., McHugo G., Williams V., Hanrahan P., Stefancic A. Measuring homelessness and residential stability: The residential time-line follow-back inventory. Journal of Community Psychology 2007;35:29-42.
58. Uttaro T., Lehman A. Graded response modeling of the Quality of Life Interview. Evaluation and Program Planning 1999;22:41-52.
59. Barker S, Barron N, McFarland BH, Bigelow DA, Carnahan T. A community ability scale for chronically mentally ill consumers: Part II. Applications. Community Ment Health Journal 1994;30:459-72.
60. Dickerson FB, Origoni AE, Pater A, Friedman BK, Kordonski WM. An expanded version of the Multnomah Community Ability Scale: anchors and interview probes for the assessment of adults with serious mental illness. Community Ment Health Journal 2003;39:131-7.
61. Conrad KJ, Yagelka JR, Matters MD, Rich A.R., Williams V., Buchanan M. Reliability and validity of a modified Colorado Symptom Index in a national homeless sample. Mental Health Services Research 2001;3:141-53.
62. Felitti VJ, Anda RF, Nordenberg D, et al. Relationship of childhood abuse and household dysfunction to many of the leading causes of death in adults. The Adverse Childhood Experiences (ACE) Study. Am J Prev Med 1998;14:245-58.
63. Lecrubier Y, Sheehan DV, Weiller E, et al. The Mini International Neuropsychiatric Interview (MINI). A short diagnostic structured interview: reliability and validity according to the CIDI. European Psychiatry 1997;12:224-31.
64. White IR, Royston P, Wood AM. Multiple imputation using chained equations: Issues and guidance for practice. Statistics in Medicine 2010;30:377-99.
65. van Buuren S. Flexible Imputation of Missing Data: CRC Press; 2012.
66. R: A language and environment for statistical computing. R Foundation for Statistical Computing V, Austria (https://www.R-project.org/).
67. Streiner DL, Norman GR. Correction for multiple testing: is there a resolution? . Chest 2011;140(1):16-18.
68. Aubry T, Nelson G, Tsemberis S. Housing First for People with Severe Mental Illness Who are Homeless: A Review of the Research and Findings from the at Home—Chez soi Demonstration Project. The Canadian Journal of Psychiatry 2015;60:467-74.
69. Hall G, Walters S, Gould H, Lim S. Housing versus treatment first for supportive housing participants with substance use disorders: A comparison of housing and public service use outcomes. Subst Abus 2020;41:70-6.
70. Clifasefi SL, Collins SE. The life-enhancing alcohol-management program: Results from a 6-month nonrandomized controlled pilot study assessing a community based participatory research program in housing first. J Community Psychol 2020;48:763-76.
71. Dietz TL. Substance Misuse, Suicidal Ideation, and Suicide Attempts Among a National Sample of Homeless. Journal of Social Service Research 2010;37:1-18.
72. Guillén AI, Marín C, Panadero S, Vázquez JJ. Substance use, stressful life events and mental health: A longitudinal study among homeless women in Madrid (Spain). Addictive Behaviors 2020;103:106246.
73. Edalati H, Nicholls TL, Schütz CG, et al. Examining the Relationships between Cumulative Childhood Adversity and the Risk of Criminal Justice Involvement and Victimization among Homeless Adults with Mental Illnesses after Receiving Housing First Intervention. Can J Psychiatry 2020;65:409-17.

74. Mersky JP, Janczewski CE, Topitzes J. Rethinking the Measurement of Adversity. Child Maltreat 2017;22:58-68.
75. Farrell SJ, Dunn M, Huff J. Examining Health Literacy Levels in Homeless Persons and Vulnerably Housed Persons with Mental Health Disorders. Community mental health journal 2020;56:645-51.
76. O'Campo P, Daoud N, Hamilton-Wright S, Dunn J. Conceptualizing Housing Instability: Experiences with Material and Psychological Instability Among Women Living with Partner Violence. Housing Studies 2016;31:1-19.
77. Hopper EK, Bassuk EL, Olivet J. Shelter from the storm: Trauma-informed care in homelessness services settings. The Open Health Services and Policy Journal 2010;3(2), 80-100.
78. Bingham B, Moniruzzaman A, Patterson M, et al. Gender differences among Indigenous Canadians experiencing homelessness and mental illness. BMC psychology 2019;7:57.
79. Yakubovich AR, Maki K. Preventing Gender-Based Homelessness in Canada During the COVID-19 Pandemic and Beyond: The Need to Account for Violence Against Women. Violence Against Women 2021:10778012211034202.

Table 1: Baseline characteristics of women participants in the Canadian At Home/Chez Soi study by randomization group (*N* = 653)

Formatted Table

Characteristics	Treatment as Usual (N = 279)		Housing First (N = 374)	
	N	%	N	%
Age (M ± SD), y	40.2 ± 11.7		39.7 ± 11.2	
Ethnic or cultural identity				
Aboriginal	76	27.2	95	25.4
Ethno-racial	67	24.0	81	21.7
White	136	48.8	198	52.9
Education				
Less than high school	130	46.9	202	54.3
Completed high school	57	20.6	61	16.4
Some post-secondary school	90	32.5	109	29.3
Marital status				
Married/Partnered	22	7.9	16	4.3
Divorced/Separated/Widowed	74	26.5	106	28.4
Single/Never married	183	65.6	251	67.3
Country of birth, & length of time in Canada for immigrants				
Born in Canada	235	84.8	315	84.2
Immigrant, 10 years or less	8	2.8	12	3.2

**Characteristics**	**Treatment as Usual (N = 279)**		**Housing First (N = 374)**	
	N	%	N	%
Immigrant, more than 10 years	34	12.3	47	12.6
Employment status				
Unemployed (unemployed, volunteer work, retired, student, housewife/husband, other)	270	96.8	362	97.3
Employed (including self-employed and special work program)	9	3.2	10	2.7
Sources of income				
Earnings from regular/casual work or busking	16	5.7	18	4.8
Unemployment insurance	22	7.9	26	7.0
Social assistance (disability, welfare, pension, personal needs allowance)	245	87.8	338	90.4
Selling papers, crafts, pan-handling, squeegee, or $ from recycling (bottles, scrap metal)	16	5.7	22	5.9
Other (loans, theft, sex work, help from family or friends)	12	4.3	10	2.7
Housing status				
Absolutely Homeless	215	77.1	296	79.1

Formatted Table

**Characteristics**	**Treatment as Usual (N = 279)**		**Housing First (N = 374)**	
	N	%	N	%
Precariously housed	64	22.9	78	20.9
Lifetime duration of homelessness (M ± SD)[a], y	3.8 ± 4.6		3.8 ± 4.8	
Less than 3 years duration	155	57.8	211	58.0
3 years or more duration	113	42.2	153	42.0
Has minor children (≤18 yrs of age)				
0	154	56.0	221	59.1
1-2	81	29.4	110	29.4
3 or more	40	14.6	43	11.5
Has minor children (≤18 yrs of age) & provides full/partial support				
0	88	72.7	125	82.2
1	20	16.5	16	10.5
2 or more	13	10.7	11	7.2
Relationship to minor children (≤ 18 yrs of age)				
Biological parent	121	100.0	151	98.7
Partner's, adoptive or other adult relative's child	0	0	5	3.4
MCAS[b] score (M ± SD)	60.7 ± 8.0		61.8 ± 7.8	
MINI[c] diagnostic categories				
Depressive episode	170	60.9	218	58.3
Manic or hypomanic episode	50	17.9	55	14.7

Formatted Table

Characteristics	Treatment as Usual (N = 279)		Housing First (N = 374)	
	N	%	N	%
Post-traumatic stress disorder	126	45.3	145	38.8
Panic disorder	94	33.7	102	27.3
Mood disorder with psychotic features	54	19.4	54	14.5
Psychotic disorder	81	29.0	95	25.4
Alcohol dependence	97	34.8	108	28.9
Substance dependence	134	48.0	167	44.7
Suicidality				
Not suicidal	46	16.5	62	16.6
Low	119	42.7	170	45.5
Moderate	50	17.9	70	18.7
High	64	22.9	72	19.3
Adverse Childhood Experiences Scale, mean (SD)	5.1	3.0	5.2	3.2
Less than 5	95	39.9	137	40.5
5 or more	143	60.1	201	59.5
Comorbid medical conditions				
Less than 3	51	18.6	100	27.0
3 or more	223	81.4	270	73.0
Study Component				
Moderate Needs	144	51.6	232	62.0
High Needs	135	48.4	142	38.0

[a] Median (IQR), y 2.0 (0.8-5.0) for Treatment as Usual and 2.0 (0.7-5.0) for Housing First ; [b] MCAS: Multnomah Community Ability Scale. Possible scores range from 17 to 85, with lower scores indicating more disability.;

[c] MINI: The Mini-International Neuropsychiatric Interview

Table 2. Post-Imputation Pooled change in health and social measures from Baseline to 24 Months among Women living with Mental illness and Homelessness, Adjusted for Study City, and Need Level (ACT or ICM).

	**Housing First (HF) (N=374)**		**Treatment as Usual (TAU) (N=279)**		p-value for differences between HF and TAU
**Housing Stability**					
Mean percentage of days spent stably housed over the 24 month follow-up.	74.8%	71.7%–77.8%	37.9%	34.4%–41.3%	0.001
**Continuous Outcomes (95% CI)**	Mean Change from Baseline to 24 Months	95% CI	Mean Change from Baseline to 24 Months	95% CI	p
Mental illness symptom severity (CSI[a])	-6.3	-7.5 to -5.0	-6.9	-8.4 to -5.4	0.534
Community functioning (MCAS[b])	3.8	2.8 - 4.9	4.8	3.6 - 6.0	0.236
Condition-specific QoL (QoLI-20[c])	16.0	13.6 - 18.4	13.4	10.6 - 16.2	0.178

Formatted Table

Formatted Table

Formatted Table

Psychological community integration (CIS[d])	2.0	1.5 - 2.4	2.0	1.4 - 2.6	0.941
**Count Outcomes**	Rate Ratio Comparing 24 Months with Baseline	95% CI	Rate Ratio Comparing 24 Months with Baseline	95% CI	p
Physical community integration in past month (CIS[d])	0.97	0.86 - 1.08	1.03	0.92 - 1.14	0.439
Severity of substance problems in past month (GAIN-SS[e])	0.79	0.67 - 0.93	0.83	0.70 - 0.98	0.688
Number of emergency department visits in past 6 months	0.38	0.29 - 0.50	0.37	0.27 - 0.51	0.926

Note. Housing outcome is mean proportion of days spent stably housed.

[a] CSI: Colorado Symptom Index. Possible scores range from 14 to 70, with higher values indicating greater symptom severity.

[b] MCAS: Multnomah Community Ability Scale. Possible scores range from 17 to 85, with lower values indicating more disability.

[c] QoLI-20: Lehman Quality of Life Interview 20. Possible scores range from 20 to 140, with higher values indicating greater quality of life.

[d] CIS: Community Integration Scale. Possible scores range from 4 to 20 for the Psychological component and 0-7 for the Physical component, with higher values indicating greater integration.

[e] GAIN-SS: Global Assessment of Individual Needs Short Screener. Possible scores range from 0 to 5 with higher values indicating greater problem severity.

Formatted Table

Table 3. Post-Imputation Pooled Comparisons between Housing First and Treatment as Usual at 6, 12, 18 and 24 months for Women in the At Home/Chez Soi Trial Adjusted for Study City, Need Level (ICM or ACT) and Baseline Outcome Values[f]

	Month	Housing First		Treatment as Usual		Comparison		
**Continuous Outcome**		**Mean**	**95% CI**	**Mean**	**95% CI**	**Mean Difference**	**95% CI**	**p**
Mental illness symptom severity (CSI[a])	6	35.8	34.8 - 36.8	36.8	35.6 - 38.0	-1.0	-2.5 to 0.5	0.183
	12	34.6	33.6 - 35.6	35.3	34.1 - 36.6	-0.7	-2.3 to 0.8	0.364
	18	33.8	32.7 - 35.0	34.1	32.7 - 35.4	-0.2	-2.0 to 1.5	0.785
	24	33.7	32.6 - 34.8	33.4	32.1 - 34.7	0.3	-1.4 to 2.0	0.737
Community functioning (MCAS[b])	6	64.9	64.1 - 65.7	64.9	64.0 - 65.9	-0.02	-1.2 to 1.2	0.975
	12	65.0	64.2 - 65.8	64.3	63.4 - 65.3	0.7	-0.6 to 1.9	0.299
	18	65.6	64.8 - 66.5	64.9	63.8 - 65.9	0.8	-0.6 to 2.1	0.267

Formatted Table

Formatted Table

	24	65.5	64.7 - 66.4	65.9	64.9 - 66.9	-0.4	-1.7 to 0.9	0.593
Condition-specific QoL (QoLI-20[c])	6	84.4	82.4 - 86.4	77.3	75.0 - 79.6	7.1	4.1 - 10.1	<.001
	12	87.4	85.4 - 89.4	81.3	79.0 - 83.7	6.1	3.1 - 9.2	<.001
	18	84.8	82.8 - 86.9	82.2	79.9 - 84.6	2.6	-0.4 to 5.6	0.091
	24	86.8	84.6 - 89.0	84.3	81.8 - 86.7	2.5	-0.7 to 5.8	0.124
Psychological community integration (CIS[d])	6	12.4	12.0 - 12.7	11.8	11.4 - 12.3	0.5	-0.1 to 1.1	0.077
	12	12.8	12.4 - 13.2	12.1	11.6 - 12.5	0.7	0.1 to 1.3	0.017
	18	12.7	12.3 - 13.1	12.3	11.8 - 12.7	0.4	-0.2 to 1.0	0.194
	24	12.9	12.5 - 13.2	12.7	12.2 - 13.1	0.2	-0.4 to 0.8	0.458
**Count Outcome**		**Rate**	**95% CI**	**Rate**	**95% CI**	**Rate Ratio**	**95% CI**	**p**
Physical community integration in past month (CIS[d])	6	2.1	1.9 - 2.3	2.1	1.9 - 2.3	0.98	0.87 - 1.12	0.789

Formatted Table

Formatted Table

Formatted Table

	12	1.9 1.7 - 2.1	2.0 1.8 - 2.2	0.95 0.84 - 1.09	0.492
	18	2.0 1.8 - 2.1	2.0 1.8 - 2.2	0.99 0.87 - 1.12	0.858
	24	1.9 1.7 - 2.0	2.0 1.8 - 2.2	0.91 0.80 - 1.03	0.148
Severity of substance problems in past month (GAIN-SS[e])	6	1.2 1.0 - 1.4	1.2 1.0 - 1.5	0.97 0.78 - 1.20	0.767
	12	1.3 1.1 - 1.5	1.2 1.0 - 1.4	1.09 0.87 - 1.37	0.452
	18	1.1 0.9 - 1.3	1.2 0.9 - 1.4	0.74 1.20 - 0.63	0.628
	24	1.1 0.9 - 1.2	1.0 0.9 - 1.2	1.00 0.79 - 1.27	0.977
Number of emergency department visits in past 6 months	6	1.2 0.9 - 1.4	1.4 1.0 - 1.9	0.80 0.55 - 1.16	0.241
	12	1.1 0.7 - 1.4	1.1 0.7 - 1.6	0.96 0.58 - 1.57	0.858
	18	1.1 0.8 - 1.4	0.6 0.5 - 0.8	1.81 1.26 - 2.60	0.001
	24	0.8 0.6 - 0.9	0.7 0.5 - 0.9	1.13 0.83 - 1.55	0.426

Formatted Table

a CSI: Colorado Symptom Index. Possible scores range from 14 to 70, with higher values indicating greater symptom severity.

b MCAS: Multnomah Community Ability Scale. Possible scores range from 17 to 85, with lower values indicating more disability.

c QoLI-20: Lehman Quality of Life Interview 20. Possible scores range from 20 to 140, with higher values indicating greater quality of life.

d CIS: Community Integration Scale. Possible scores range from 4 to 20 for the Psychological component and 0-7 for the Physical component, with higher values indicating greater integration.

e GAIN-SS: Global Assessment of Individual Needs Short Screener. Possible scores range from 0 to 5 with higher values indicating greater problem severity.

f Housing stability is not included in this table as we did not examine it at each follow-up point.

Table 4. Post-Imputation Pooled coefficient estimates (95% Confidence Interval) for Predictors in Linear Mixed Models for Repeated Measures for continuous outcomes and Rate Ratios (95% Confidence Interval) for Generalized Estimating Equations Models– for count outcomes for the sample of women in the At Home/Chez Soi study (N=653).

	Mental illness symptom severity (CSI)			Community functioning (MCAS)			Condition-specific QoL (QoLI-20)			Psychological community integration (CIS)		
	Est	95% CI	p-val	Est	95% CI	p-val	Est	95% CI	p-val	Est	95% CI	p-val
Age (years)	-0.01	-0.06-0.04	0.680	-0.02	-0.060-.02	0.310	-0.06	-0.17- 0.04	0.250	-0.01	-0.03- 0.00	0.140
Less than high school	0.21	-0.98-1.40	0.731	**-1.32**	**-2.27 to -0.37**	**0.006**	0.87	-1.56- 3.30	0.480	0.15	-0.26- 0.55	0.476
3 or more years of homelessness	0.91	-0.27-2.09	0.132	**-1.75**	**-2.72 to -0.78**	**<.001**	-0.75	-3.14- 1.64	0540	-0.07	-0.48- 0.33	0.728
Supports ≥1 child under 18 years	0.11	-1.89-2.10	0.917	0.62	-0.96- 2.20	0.442	0.47	-3.52- 4.47	0.820	0.48	-0.20- 1.17	0.168
Moderate or high suicidality at baseline	**2.85**	**1.59- 4.11**	**<.001**	**-1.16**	**-2.10 to -0.22**	**0.015**	**-3.99**	**-6.49 to -1.49**	**<.001**	-0.30	-0.71- 0.11	0.150
5 or more ACE	**1.50**	**0.25- 2.76**	**0.019**	0.21	-0.75- 1.17	0.666	-0.56	-3.14- 2.02	0.670	0.31	-0.12- 0.73	0.157

Formatted Table

Formatted Table

Formatted Table

	Physical community integration in past month (CIS) (N=653)			Severity of substance problems in past month (GAIN-SS) (N=653)			Number of emergency department visits in past 6 months (N=653)			Stable housing (N=653)		
		95% CI	p-val	RR	95% CI	p-val	RR	95% CI	p-val		95% CI	p-val
Age (years)	1.0	1.0- 1.0	0.834	**0.99**	**0.98-1.00**	**0.003**	1.00	0.99- 1.01	0.920	0.16	-0.04-0.36	0.122
Less than high school	**0.85**	**0.77- 0.93**	**<.001**	**1.27**	**1.06-1.52**	**0.009**	1.11	0.83-1.49	0.474	-2.78	-7.43-1.88	0.242
3 or more years of homelessness	0.98	0.93- 1.11	0.720	**1.21**	**1.03- 1.43**	**0.021**	0.99	0.76-1.29	0.951	**-6.14**	**-10.9--1.32**	**0.013**
Supports ≥1 child under 18 years	1.10	0.96-1.27	0.176	1.05	0.80- 1.39	0.715	0.92	0.67-1.26	0.598	5.57	-2.17-13.3	0.158
Moderate or high suicidality at baseline	1.02	0.93-1.12	0.634	1.06	0.90- 1.27	0.465	**1.44**	**1.10-1.87**	**<0.001**	-1.82	-6.49-2.80	0.440
5 or more ACE	1.04	0.95-1.15	0.356	1.16	0.96- 1.39	0.114	1.05	0.77-1.42	0.772	-2.93	-7.92-2.05	0.249

- Each model includes treatment group, categorical time, interaction of treatment x time, city, need level, baseline outcome values and all baseline predictors.

Figure 1 – Figure 1 – Health and social outcomes among Women who are Homeless and Mentally Ill, unadjusted means and 95% confidence intervals over 24 months of follow-up by study time point and treatment group

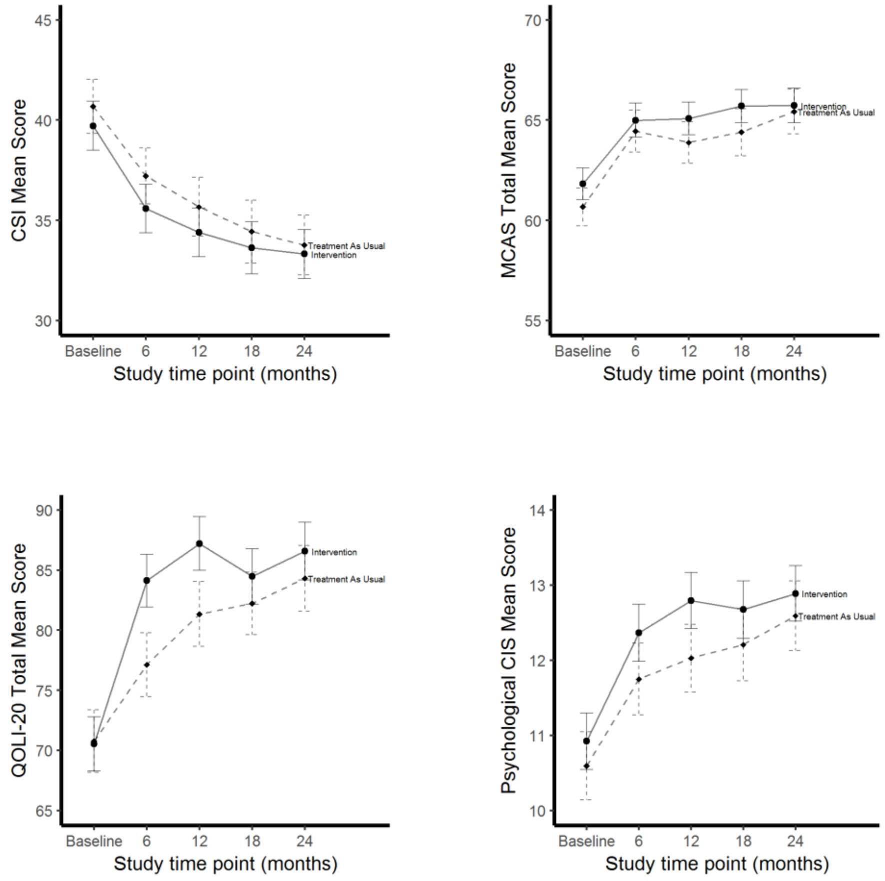

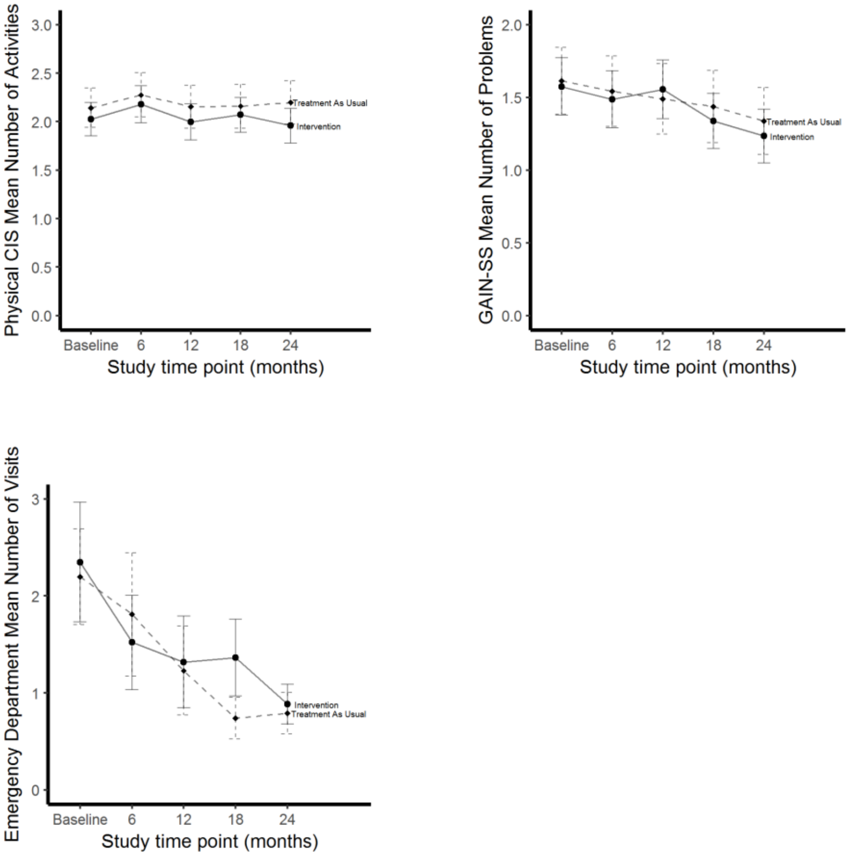

Note. *N* = 653. CSI = Colorado Symptom Index; MCAS = Multnomah Community Ability Scale; Lehman Quality of Life Interview 20 = QoLI-20; CIS = Community Integration Scale; GAIN-SS = Global Assessment of Individual Needs Short Screener.

	Editor or Reviewer comments	Author responses
Editor comments		
1	Regarding missingness, it's important to address the missing data mechanisms in more depth than currently, and also whether these mechanisms result in data that are missing at random, missing completely at random, or even not missing at random. Given the diverse reasons for missingness, it is possible that more than one type of missingness is present in the data.	Missing data mechanisms have been widely presented and discussed in the literature. Although the Missing Completely at Random mechanism simplifies analyses, it is quite difficult to find real life applications that satisfy its assumption [VanBuuren2012], and therefore we did not consider it in our data.<br><br>We did however consider the Missing at Random (MAR) Mechanism, where the probability of missingness depends only on observed data but not on the data that are missing. That is, after controlling for or stratifying by observed variables, missingness is random. We followed recommendation from Van Buuren 2012 and Barnard 1999 to include in our imputation models a high the number of predictors of the incomplete variables in order to make the MAR assumptions more plausible.<br><br>Van Buuren, S. (2012), Flexible Imputation of Missing Data. Chapman & Hall/CRC, Boca Raton, FL.<br><br>Barnard J, Meng XL Applications of multiple imputation in medical studies: from AIDS to NHANES. Statistical Methods in Medical Research. 8:17-36, 1999
2	70% of data had at least one missing value. With such a large percent like 70%, a reader might think that is in the entire dataset. It seems from context and supplementary table 1 that this was all variables that are used in this paper's analysis, so please say that more clearly. Also given that 70% of data used in the study had at least one missing value, please cite research about the implications of MI in data with such a large percent missing.	We followed recommendations from White et al, 2010 and Horton et al. 2007 who suggest that depending on the number and type of variables, and amount of missing data, 50 to 100 imputations yield better estimates. However, a rule of thumb was provided by White et al. 2010 that the number of imputations should be at least 100 x (percent of incomplete cases) and we applied this recommendation to our data analysis approach.<br><br>Horton, N. & Kleinman, K.P. (2007). Much ado about nothing: A comparison of missing data methods and software to fit incomplete data regression models. The American Statistician 61(1): 79-90. Software appendix: http://www.math.smith.edu/muchado-appendix.pdf<br><br>White, I.R., Royston, P., and Wood, A.M. (2010). Multiple imputation using chained equations: Issues and guidance for practice. Statistics in Medicine 30:377-399. (Includes Stata codes)
3	One reviewer asked for sensitivity analysis for imputed versus non-imputed. Diagnostics of MI method may be appropriate.	As requested, we provide a sample of multiple imputation diagnostic plots as suggested by Van Buuren, 2012 at the end of this document.

<table>
<tr><td></td><td></td><td>We also added the following sentence to the methods: Comparisons between observed and imputed data via diagnostic plots were used to assess the quality and plausibility of the imputations.

Van Buuren, S. (2012), Flexible Imputation of Missing Data. Chapman & Hall/CRC, Boca Raton, FL.</td></tr>
<tr><td>4</td><td>With regard to sample size calculation, an alternative to a standard power calculation would be Gelman and Carlin type-M and type-S errors, which is less centered on p-values.</td><td>Because ours was a post-hoc subgroup exploratory analysis of the main study, the sample size calculation which was predetermined for the main hypotheses for the main study involving the full sample, does not apply here. As such, for the analyses we present here, we would like to emphasize that our study was hypothesis generating and therefore power calculations do not apply. The original sample sizes were calculated for the original study, please refer to protocol in Goering et al.

Goering PN, Streiner DL, Adair C, Aubry T, Barker J, Distasio J, Hwang SW, Komaroff J, Latimer E, Somers J, Zabkiewicz DM. The At Home/Chez Soi trial protocol: a pragmatic, multi-site, randomised controlled trial of a Housing First intervention for homeless individuals with mental illness in five Canadian cities. BMJ Open. 2011 Nov 14;1(2):e000323</td></tr>
<tr><td>5</td><td>The effects of the experiment have been displayed as p-values. An effect size measure such as Hedge’s g or Cohen’s d in addition to the differences that are in the tables 2-4. These effect size measures may give a clearer picture of the true results of the experiment.</td><td>We are familiar with Cohen’s d and the Hedge’s g correction as alternate ways of illustrating differences between treatment and control groups. We did not use Cohen’s d in any of our dozens of publications that emerged from this randomized trial as in our experience it is not as common in health or medical studies as it would be in psychology. In fact, a quick review of evaluations or randomized trials published in Plos One over a one-year period (back to Dec 2020, see citations below) demonstrated that these studies report findings in a similar way to which we presented our findings. Evaluations presented mean values of outcomes for treatment and controls groups along with 95% confidence intervals and p-values. Some studies add in the t-statistics. As such, we feel that adding Cohen’s d values were not necessary.

Hajek P, Przulj D, Pesola F, McRobbie H, Peerbux S, Phillips-Waller A, et al. (2021) A randomised controlled trial of the 5:2 diet. PLoS ONE 16(11): e0258853.

Ngugi P, Babic A, Were MC (2021) A multivariate statistical evaluation of actual use of electronic health record systems implementations in Kenya. PLoS ONE 16(9): e0256799.

Müller SA, Diallo AOK, Rocha C, Wood R, Landsmann L, Camara BS, et al. (2021) Mixed methods study evaluating the implementation of the WHO hand hygiene strategy focusing on alcohol based handrub and training among health care workers in Faranah, Guinea. PLoS ONE 16(8): e0256760.</td></tr>
</table>

		Andersen TO, Dissing AS, Varga TV, Rod NH (2021) The SmartSleep Experiment: Evaluation of changes in night-time smartphone behavior following a mass media citizen science campaign. PLoS ONE 16(7): e0253783.<br><br>Greene MC, Likindikoki S, Rees S, Bonz A, Kaysen D, Misinzo L, et al. (2021) Evaluation of an integrated intervention to reduce psychological distress and intimate partner violence in refugees: Results from the Nguvu cluster randomized feasibility trial. PLoS ONE 16(6): e0252982.<br><br>Heimgartner N, Meier S, Grolimund S, Ponti S, Arpagaus S, Kappeler F, et al. (2021) Randomized controlled evaluation of the psychophysiological effects of social support stress management in healthy women. PLoS ONE 16(6): e0252568.<br><br>Dardas A, Williams A, Wang L (2021) Evaluating changes in workplace culture: Effectiveness of a caregiver-friendly workplace program in a public post-secondary educational institution. PLoS ONE 16(5): e0250978.<br><br>O'Donnell A, Wilson L, Bosch JA, Borrows R (2020) Life satisfaction and happiness in patients shielding from the COVID-19 global pandemic: A randomised controlled study of the 'mood as information' theory. PLoS ONE 15(12): e0243278.
6	P-values have been displayed to 3 digits in all tables. That's too many digits and gives false precision. Just one significant digit is fine. For instance, in table 2 the p-values would be 0.001 (as is currently there), 0.5, 0.2, 0.2, 0.9, 0.4, 0.7, 0.9.	We respectfully disagree with this suggestion to report p-values using 1 significant digit. We not only feel this is imprecise but this practice is not aligned with what we discovered in our review of studies published in Plos One. Studies reviewed (see item above) reported p-values using 3 digits or sometimes more significant digits. Therefore, we have not reduced the number of significant digits we report for our p-values in our tables and text.
7	Figure 1 is really nice display of results, but the treated and control groups should be shifted slightly so the confidence intervals are clearer and don't overlap— e.g., intervention at 0.5 months below each time point and control at 0.5 months above each time point. Many of these continuous variable outcomes in figure 1 probably have complex distributions, and it may be helpful to show the distributions of these variables for treatment versus control, such as using a violin plot or similar data display, rather than the point and CI shown in figure 1.	Thank you for your appreciation of our figures. The figures are intended to augment the study findings in the tables as Table 3 presents results of multivariate analyses. The Figures with the unadjusted results are intended to be a visual supplement to Table 3 as the differences or lack thereof are easier to grasp quickly in the figures. While CIs are included in the Figures to help identify whether the values at any one time point differ between treatment and TAU, the data in the Tables reporting multivariate regression findings should be the primary source of information about differences and significance of those differences. To change the scales on the figures to further separate the trend lines for the treatment and TAU would create larger figures that would take up too much space and is not necessary given that the multivariate findings are presented in Table 3.
8	The tables are somewhat hard to read with many lines, and they may benefit from using	We have added formats to the table to increase readability. In particular, we have shaded every other row in Tables 1, 3 and 4. We have added lines between each

	data displays such as dumbbell plots for comparisons of the treatment vs control.	outcome to more clearly delineate the findings for each outcome and co-variate. We believe the format changes have considerably increased the readability of the Tables. Moreover the figures we provided as supplementary information about Table 3 is a visual aid to rapidly understanding the findings from the analysis of these trial data.
9	The mixed models should report evaluation of assumptions of the models, such as linear regression, Poisson regression.	To respond to this suggestion we have added the following text in the Statistical analysis section: Normality of residuals was graphically checked for linear mixed models. The decision to fit a negative binomial rather than a Poisson model was based on whether over-dispersion (, ie, if the variance was much larger than the mean) was present in the count outcome data.
10	Table 2 shows percent of the time housed over all visits. However, the other measures in table 2 are a comparison of baseline to 24 months adjusted for city and need level, which leaves out the middle 3 follow-up visits. It seems like these observations in table 2 could be more completely displayed as an adjusted version of Figure 1 that includes all time points adjusted for city and need level.	There were several reasons why our study measured housing success as *cumulative stability at 24 months* and we now state this in the methods. First, given the Housing First Theory of Change and Program Manual, stable housing is established within the first 1-3 months of enrolment for those in the treatment arm and housing status rarely changes after that point in time. Thus, we wanted to align with the program's theory of change. Second, to measure housing status at different time points over the 24 months means that we would have seen very little change for those in the treatment arm and a single cumulative measure made more sense for this outcome. This measure captures overall stability and instability in that a small proportion of the treatment group was not stably housed throughout and this single measure captures that information. Third, this single measure captures the overall housing stability or instability across the two groups over a long period of time and for this outcome, knowing the housing status at different time points in the study is less relevant to the overall goals of the program. In sum, we chose the best measure for the outcome of housing even if it did not match how we approached other outcomes such as quality of life which is expected to demonstrate incremental changes over the 24-month follow-up period.
11	Table 4 shows mixed model effects that leaves out the treatment effect and treatment x time, among other variables. It seems that this table should at least include treatment effect coefficients. Omitting the main effect of treatment doesn't avoid table 2 fallacy. The authors should address whether table 2 fallacy may apply here.	Table 4 presents the findings for the third objective of the paper (see bottom of page 7 of the manuscript for the objective) intended to "explore baseline demographic, social, health measures, and ACEs as predictors of key study outcomes during the study period." This objective is not concerned with differences between treatment and TAU. In fact, we include that objective because few differences between treatment and TAU were reported and we can, therefore, take advantage of this large sample of homeless women to learn more about predictors of our key outcomes. Consequently, our analysis for this objective does not account for treatment group membership by design. We do not address the issue of type 2 fallacy for this objective as it does not apply. However, we have made our intent clearer in the statistical analyses, see top of page 11 and in the results on page 13
12	3. Please provide additional details regarding participant consent. In the ethics statement in the Methods and online submission information, please ensure that you have specified what type you obtained (for instance, written or verbal, and if verbal, how it was documented and witnessed). If your study included minors, state whether you obtained consent from parents or guardians. If the need	We have expanded the description of the consent process and Ethics Board approval process on page 8 of the revised manuscript.

	for consent was waived by the ethics committee, please include this information.	
13	4. We note that the grant information you provided in the 'Funding Information' and 'Financial Disclosure' sections do not match.	We tried upon our initial submission to create a new entry online for our funding agency, the Mental Health Commission of Canada. However, when we try to add this new funding agency to your database the system freezes and cannot seem to complete the task. Therefore we did not do this task but are willing if we get assistance to do so. We did also try to email the generic help email but received an unhelpful response that we should enter the information into the field with no guidance on how to overcome our problem.
14	When you resubmit, please ensure that you provide the correct grant numbers for the awards you received for your study in the 'Funding Information' section.	There was no grant number for this award.
Reviewer comments		
15	Reviewer #1: Overall, the study sheds important light on the effectiveness of Housing First intervention among women with mental illness. However, there were a few areas that could be strengthened. In the introduction section, beyond the scant literature on women in permanent supportive housing programs, the authors should consider strengthening the significance of the study. A clearly stated rationale is warranted for the study.	We have inserted a strong rationale for this study that focuses on the unique features of the data and the rationale for focusing on just women in this analysis.
16	The methods section includes instruments that have strong psychometric properties in the literature. However, the authors do not report any reliability coefficient of the instruments. Further, no testing of group differences on demographic characteristics were reported. The reasons for selecting predictor and control variables are unclear.	We chose instruments with high reliability and validity for this multi-city randomized trial. In this paper, we described the instruments as we have previously done in our published work (see below for example papers). Unfortunately we did not calculate the reliability of coefficients for the individual scales using our data.<br><br>Stergiopoulos V, Hwang SW, Gozdzik A, Nisenbaum R, Latimer E, Rabouin D, Adair CE, Bourque J, Connelly J, Frankish J, Katz LY, Mason K, Misir V, O'Brien K, Sareen J, Schütz CG, Singer A, Streiner DL, Vasiliadis HM, Goering PN; At Home/Chez Soi Investigators. Effect of scattered-site housing using rent supplements and intensive case management on housing stability among homeless adults with mental illness: a randomized trial. JAMA. 2015 Mar 3;313(9):905-15. doi: 10.1001/jama.2015.1163. PMID: 25734732.

		Aubry T, Goering P, Veldhuizen S, Adair CE, Bourque J, Distasio J, Latimer E, Stergiopoulos V, Somers J, Streiner DL, Tsemberis S. A Multiple-City RCT of Housing First With Assertive Community Treatment for Homeless Canadians With Serious Mental Illness. Psychiatr Serv. 2016 Mar;67(3):275-81. doi: 10.1176/appi.ps.201400587. Epub 2015 Dec 1. PMID: 26620289.<br><br>Following guidelines from CONSORT 2010 Explanation and Elaboration<br><br>Moher D,Hopewell S,Schulz K F, Montori V, GÃ¸tzsche P C, Devereaux P J et al. CONSORT 2010 Explanation and Elaboration: updated guidelines for reporting parallel group randomized<br>trials BMJ 2010; 340 :c869 doi:10.1136/bmj.c869<br><br>We did not include p-values in Table 1, as any differences in our two groups from this randomized sample are expected to occur by chance. In addition, many of the variables in Table 1 are included for descriptive purposes only. For the purposes of this reply to reviewer suggestions, however, we did perform tests for selected variables and none had p <0.05, except study component (p=0.0077) and number of Comorbid medical conditions (P=0.0127). We have provided this information below in a table for the purposes of this response.
17	Reviewer #2: This manuscript reports a secondary analysis of the original Canadian five city Housing First trial focused solely on female participants. The authors largely repeat the analyses previously reported, but with a specific focus on women; they also add an examination of specific risk factors that might be associated with differential outcomes for women.<br><br>Because of the size and innovation of the original study, the secondary analysis is still able to provide some new insights and considerations in an important subsample. As the authors note, many investigators have argued for separate studies or at least distinguishing analyses to understand the numerous differences reported among men and women experiencing homelessness. Moreover, the homelessness epidemic has only worsened in the context of the COVID pandemic so the	Thank you for this summary and suggestion. We have inserted a strong rationale for this study that focuses on the unique features of the data and the rationale for focusing on just women in this analysis.

	issues remain relevant if these new analyses can provide insights.<br><br>Several strengths are evident besides the original sample size and randomization. First, the authors do include novel risk/resilience factors that might alter the outcomes for women experiencing homelessness such as the caretaking status of dependent children, exposure to ACES, and others. These factors have not been sufficiently considered in earlier studies of Housing First. Secondly, the authors examined group by time effects and group differences at each interval to assess the relative changes from the intervention. Thirdly, they provide detailed reporting on the individual outcomes and the relative importance or size of the changes.	
18	Some changes would also strengthen the manuscript. The most important is better clarity and continuity among the introduction, the results and the discussion. Para 3 of the intro makes a strong argument for providing data for women experiencing homelessness. However, para 5 points to studies that tried and found no differences. Why will this study be different than those studies?	Thank you for pointing out this inconsistency. We have re-written this section of the Introduction. We now make it clear that those studies that examined women had very small sample sizes. Those large studies were observational and also were based upon comparing genders and not focusing on heterogeneity of the women in the sample. With the additional of text we distinguish our study from what is currently in the literature.
19	Similarly, in the results, the authors found intervention women did NOT differ on most assessments with the exception of stably housed days (the primary intervention). However, this is different than the original finding of the five city study where all participants (men and women together) had improved quality of life and integration. No comment or discussion on this 'lessening' of effects for women is provided.<br><br>This theme continues into the discussion where the authors conclude with the need for more studies of women even though they find no	We have clarified this point in the paper. In fact, the results for this subsample of women and those for the full sample align and we have noted this now in the discussion.

	clear benefit from separate analysis in this example nor in the cited studies in their introduction. Reconciling their results, introduction and discussion seem like a critical piece because it is the main contribution of the study rather than some of the ancillary factors they look at.	
20	Tables 3 and 4 are difficult to read given the multitude of comparisons that are made.	We have applied better formatting for the tables making them easier to read. Specific changes are described above.
21	Reviewer #3: Thank you for the opportunity to review this important work. This study examined the social, health, and housing needs of women experiencing homelessness. It will make an important contribution to the evidence base as the experiences of this population have received little attention. The strengths of this study include a large sample of women experiencing homelessness in the context of a multi-site randomized controlled Housing First trial, a women-only analysis, and baseline factors and outcomes that have not been fully explored in past research. However, there are some comments that need to be addressed, including questions about the statistical analysis, and adding to the discussion section.<br><br>Is the manuscript technically sound? And do the data support the conclusions?<br><br>Yes, the manuscript is technically sound, and the methods and analysis are clearly described to allow for replication. Data are also consistent with discussion section and conclusions. There are a few items that needs to be addressed:<br>- Please describe baseline predictors and how they were coded in the methods section<br>- For women with children in the sample, were any living with their children? What type of support was provided to children? Financial? Social?<br>- Study findings are very interesting and surprising, particularly the lack of significant findings associated with health and social outcomes. Can prior research provide an	We have added more information about our study variables. Specifically we added information to the methods about any scales we used (e.g., ACEs). We state in the results that support of children refers to partial or full financial support. We did not find any prior studies to explain the small difference in favor of TAU at 18 months for ED visits. We also explain in the methods the rationale for our selection of predictor variables for the statistical models for our third objective which did not include variables such as marital status. We have rewritten the Discussion so that it is more closely linked to the existing literature and is less like a repeat of the results section. We have also fixed the sentence that was unclear (e.g., Contrary to what….).<br><br>We have added the results of the linear regression to the abstract. Regarding sample sizes and sensitivity analyses we have provided clarification above in an earlier response.<br><br>We added the specific estimates in the sentence below of the abstract<br><br>Suicidality was a consistent predictor of increased mental health symptoms (beta=2.85, 95% CI 1.59-4.11, p<0.001), decreased quality of life (beta=-3.99, 95% CI -6.49 to -1.49, p<0.001), decreased community functioning (beta= -1.16, 95% CI -2.10 to -0.22, p=0.015) and more emergency department visits (rate ratio = 1.44, 95% CI 1.10-1.87, p<0.001) over the study period. Lower education was a predictor of lower community functioning (beta=-1.32, 95% CI -2.27 to -0.37, p=0.006) and higher substance use problems (rate ratio=1.27, 95% CI 1.06-1.52, p=0.009) during the study.<br><br>No adjustment for multiple testing was applied as we followed the guidelines in the reference below and which were observed in all of our previous publications

	explanation for the decline in ED visits for the TAU group? Were there any demographic characteristics that could have influenced the findings (marital status, racial/ethnic group, sources of income, having children, mental health diagnosis, comorbid conditions)?<br><br>- The first half of the discussion section is a bit thin and reads more like a results section than a discussion section. Consider cutting down sentences that restate the results and incorporate more explanation of findings using past research.<br>- This sentence is confusing: "Contrary to what we might have anticipated on the importance of ACEs on multiple study outcomes based upon prior demonstrating the association of ACEs with victimization and criminal justice involvement, in our study high baseline ACE scores only predicted significantly worse scores on mental illness severity (CSI)." (Line 298-300)<br><br>Has the statistical analysis been performed appropriately and rigorously?<br><br>Yes, the statistical analysis has been performed appropriately and rigorously. Please address the following comments:<br>- Please add statistical results for linear mixed models to the abstract<br>- Consider running a sensitivity analysis comparing imputed vs. non-imputed data<br>- Why were no adjustments made to account for multiple tests?<br>- Please include sample size calculations<br><br>Have the authors made all data underlying the findings in their manuscript fully available?<br><br>Yes, all data used in the manuscript has been made fully available.	Streiner DL, Norman GR. Correction for multiple testing: is there a resolution? *Chest*. 2011; 140(1):16-18.<br><br>We do not have information about whether women with children were living with them or which type of support was provided to the children. The information we have is reported in Table 1: 121/275 (44%) women in the TAU and 153/374 (41%) in the HF group reported having at least 1 child under the age of 18. Among those women who had at least 1 child under 18, 33/121 (27.3%) in the TAU and 27/152 (17.8%) in the HF group reported providing full or partial support to at least 1 child.

	Is the manuscript presented in an intelligible fashion and written in standard English?<br><br>Yes, the manuscript is clear and well-written.	

Supplemental Material for Response to Editor and Reviewers PONE-D-13748
Selected Diagnostic Plots for Intervention Group and Outcomes at 24 months
Multiple Imputation First 10 Imputations

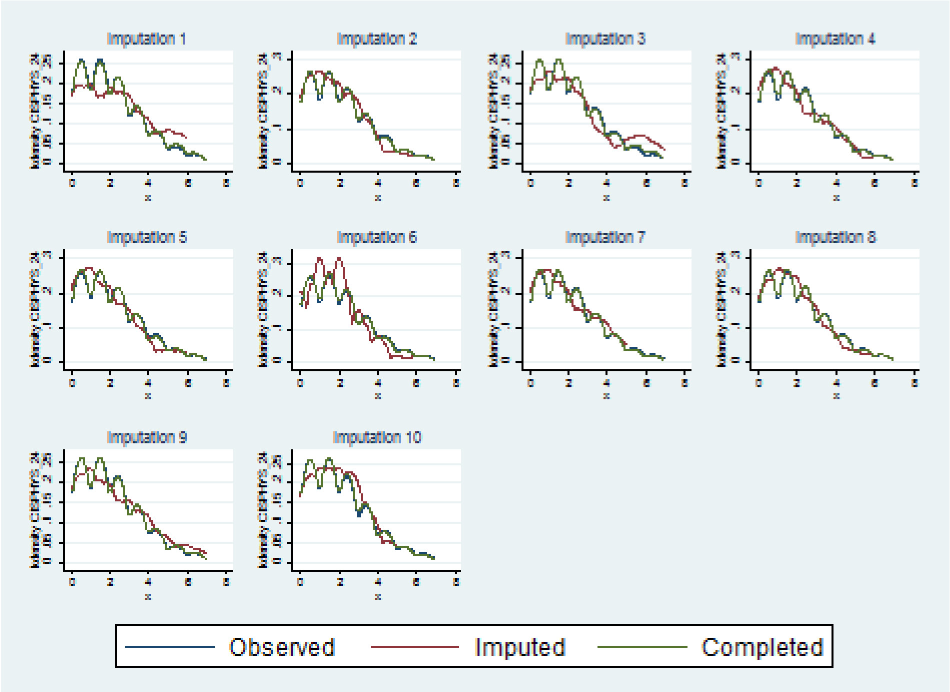

Community Physical Integration

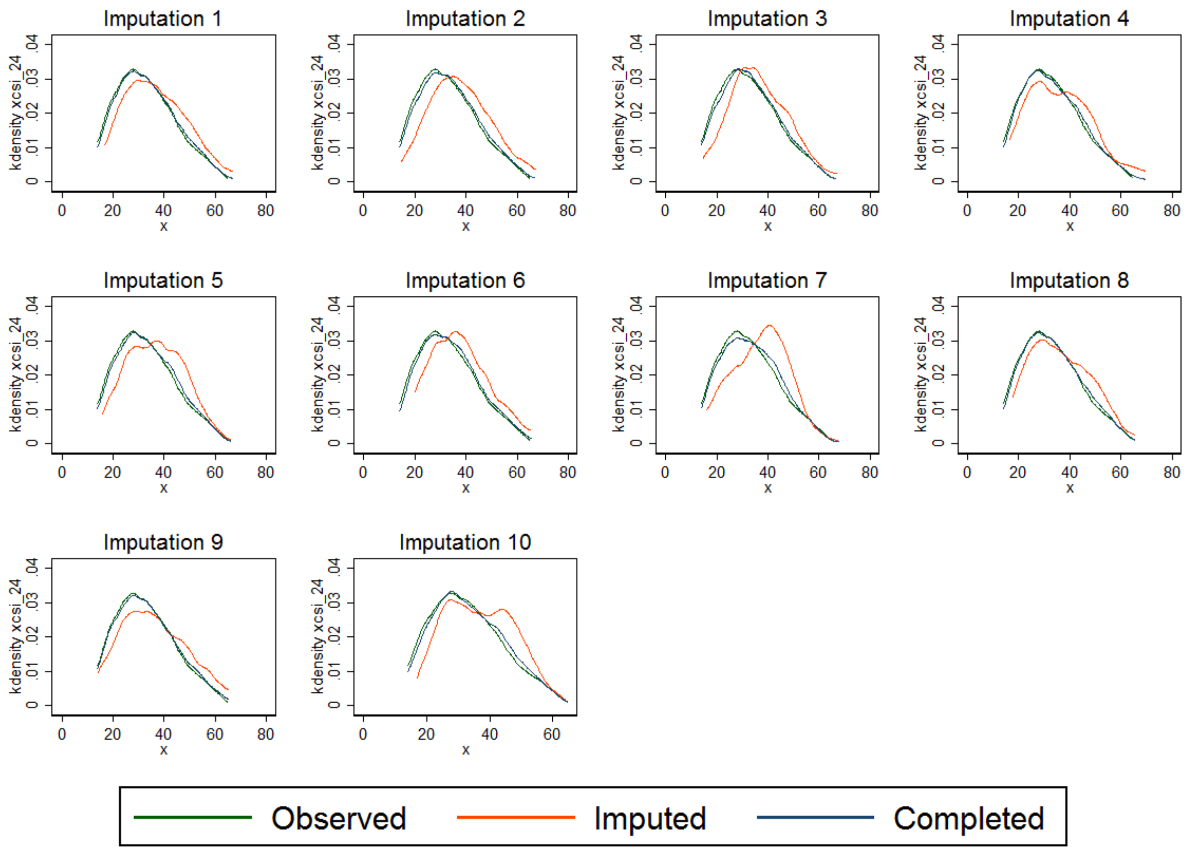

Colorado Symptom Index scores

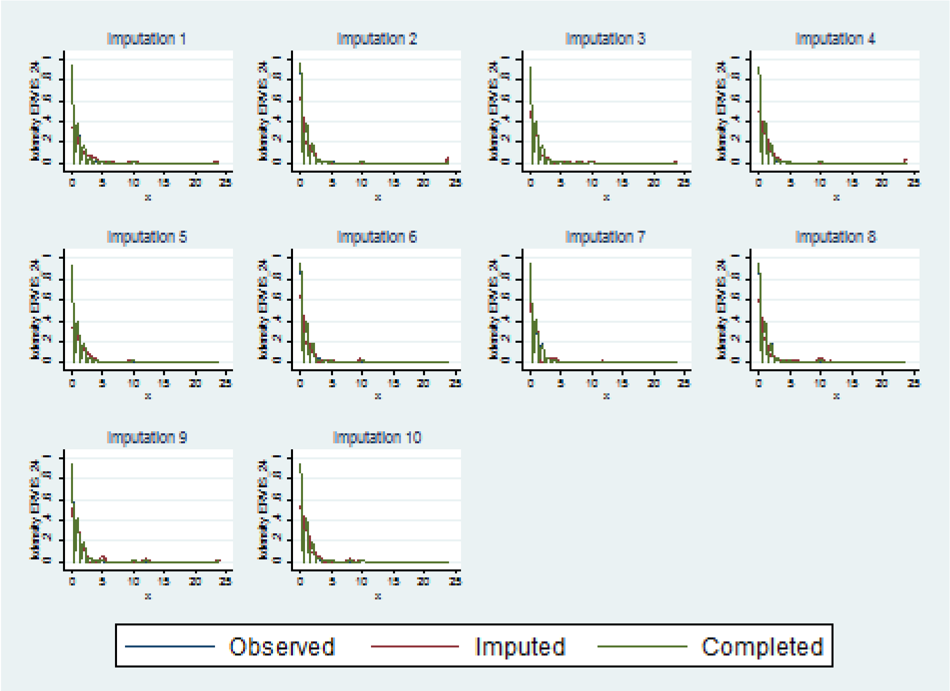

Number of Emergency Room Visits in past 6 months

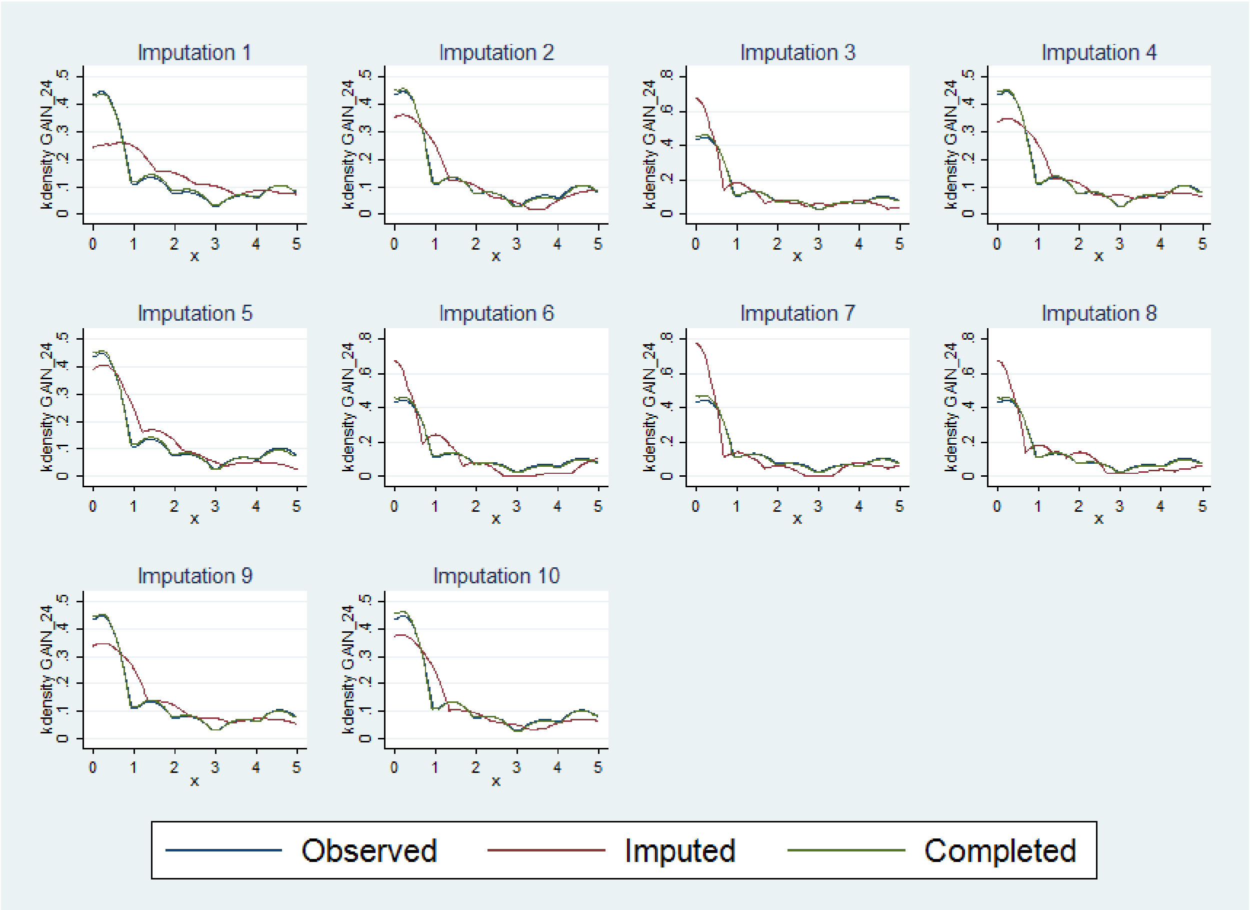

Global Assessment of Individual Needs Short Screener scores

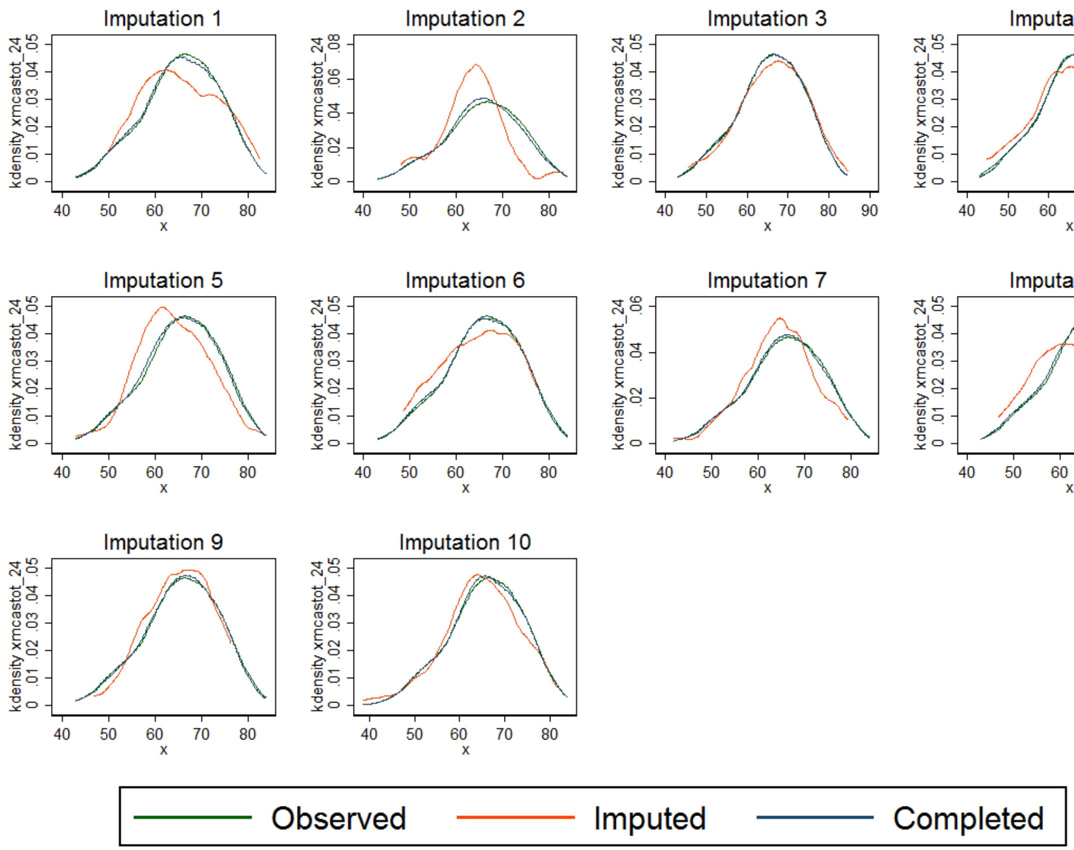

Multnomah Community Ability Scale scores

Supplemental information regarding p-values for Table 1.

Table 1: Baseline characteristics of women participants in the Canadian At Home/Chez Soi study by randomization group (*N* = 653)

**Characteristics**	**Treatment as Usual (N = 279)**		**Housing First (N = 374)**		**p-value**
	N	%	N	%	
Age (M ± SD), y	40.2 ± 11.7		39.7 ± 11.2		0.5922
Ethnic or cultural identity					0.5633
Aboriginal	76	27.2	95	25.4	
Ethno-racial	67	24.0	81	21.7	
White	136	48.8	198	52.9	
Education					0.1543
Less than high school	130	46.9	202	54.3	
Completed high school	57	20.6	61	16.4	
Some post-secondary school	90	32.5	109	29.3	
Marital status					0.1482
Married/Partnered	22	7.9	16	4.3	
Divorced/Separated/Widowed	74	26.5	106	28.4	
Single/Never married	183	65.6	251	67.3	
Country of birth, & length of time in Canada for immigrants					0.9650
Born in Canada	235	84.8	315	84.2	
Immigrant, 10 years or less	8	2.8	12	3.2	
Immigrant, more than 10 years	34	12.3	47	12.6	
Employment status					0.6867
Unemployed (unemployed, volunteer work, retired, student, housewife/husband, other)	270	96.8	362	97.3	
Employed (including self-employed and special work program)	9	3.2	10	2.7	

Characteristics	Treatment as Usual (N = 279)		Housing First (N = 374)		p-value
Sources of income					not applicable, just descriptive
Earnings from regular/casual work or busking	16	5.7	18	4.8	
Unemployment insurance	22	7.9	26	7.0	
Social assistance (disability, welfare, pension, personal needs allowance)	245	87.8	338	90.4	
Selling papers, crafts, pan-handling, squeegee, or $ from recycling (bottles, scrap metal)	16	5.7	22	5.9	
Other (loans, theft, sex work, help from family or friends)	12	4.3	10	2.7	
Housing status					0.5232
Absolutely Homeless	215	77.1	296	79.1	
Precariously housed	64	22.9	78	20.9	
Lifetime duration of homelessness (M ± SD)[a], y	3.8 ± 4.6		3.8 ± 4.8		0.5822
Less than 3 years duration	155	57.8	211	58.0	
3 years or more duration	113	42.2	153	42.0	
Has minor children (≤18 yrs of age)					0.6995
0	154	56.0	221	59.1	
1-2	81	29.4	110	29.4	
3 or more	40	14.6	43	11.5	
Has minor children (≤18 yrs of age) & provides full/partial support					0.1683
0	88	72.7	125	82.2	
1	20	16.5	16	10.5	
2 or more	13	10.7	11	7.2	
Relationship to minor children (≤ 18 yrs of age)					descriptive
Biological parent	121	100.0	151	98.7	
Partner's, adoptive or other adult relative's child	0	0	5 3.4		

Characteristics	Treatment as Usual (N = 279)		Housing First (N = 374)		p-value
MCAS[b] score (M ± SD)	60.7 ± 8.0		61.8 ± 7.8		0.0825
MINI[c] diagnostic categories					
Depressive episode	170	60.9	218	58.3	0.4962
Manic or hypomanic episode	50	17.9	55	14.7	0.2685
Post-traumatic stress disorder	126	45.3	145	38.8	0.0931
Panic disorder	94	33.7	102	27.3	0.0767
Mood disorder with psychotic features	54	19.4	54	14.5	0.0974
Psychotic disorder	81	29.0	95	25.4	0.3009
Alcohol dependence	97	34.8	108	28.9	0.1086
Substance dependence	134	48.0	167	44.7	0.3919
Suicidality					0.7096
Not suicidal	46	16.5	62	16.6	
Low	119	42.7	170	45.5	
Moderate	50	17.9	70	18.7	
High	64	22.9	72	19.3	
Adverse Childhood Experiences Scale, mean (SD)	5.1	3.0	5.2	3.2	0.8382
Less than 5	95	39.9	137	40.5	
5 or more	143	60.1	201	59.5	
Comorbid medical conditions					0.0127
Less than 3	51	18.6	100	27.0	
3 or more	223	81.4	270	73.0	
Study Component					
Moderate Needs	144	51.6	232	62.0	0.0077
High Needs	135	48.4	142	38.0	

[a] Median (IQR), y 2.0 (0.8-5.0) for Treatment as Usual and 2.0 (0.7-5.0) for Housing First ; [b] MCAS: Multnomah Community Ability Scale. Possible scores range from 17 to 85, with lower scores indicating more disability.;
[c] MINI: The Mini-International Neuropsychiatric Interview
