# Supplementary material for: Women experiencing homelessness and mental illness in a Housing First multi-site trial: Looking beyond housing to social outcomes and well-being
Source: PLoS One. 2023 Feb 10;18(2):e0277074. doi: 10.1371/journal.pone.0277074 (PMC9916643; doi:10.1371/journal.pone.0277074)
Supplement: Attachment — Submitted filename: PONE-D-21-13748_R2_reviewer-R2.pdf [file pone.0277074.s005.pdf]

7	(page 11 statistical analyses) The outcome "days spent stably housed" is continuous but bounded (between 0 and 1) so a Gaussian family is not appropriate. I suggest either to clearly state and stress that the results are to be taken as not robust or to fit a model using the beta distribution, which is appropriate for proportions/percentages.	As stated above, the percentage of days stably housed varies between 0 and 100%. While we appreciate the reviewer's suggestion to use the beta distribution, this approach has its own limitations since the beta distribution is defined for values greater than 0 and lower than 1 (assuming we would work with proportions). One of the approaches to circumvent this problem is to add a small amount, say 0.005, to zero and subtract a small amount from 1. However, in our case, 0's and 1's are structural, and we did not feel comfortable with this approach. Even in the case where we might fit such a model, we could not guarantee that it will fit the data well.<br><br>Therefore, we used the linear model which has other limitations but is easier to interpret and communicate results. Given the exploratory objective with our study, we felt comfortable with this decision.
	If there are 0s and 1s that are structurally important, then a zero-one inflated beta regression (ZOIB) is the most appropriate choice.	
8	(page 13 reference 69) I was not familiar with this paper. I strongly believe that, given the exploratory nature of this study, it is of outmost importance that multiple testing is taken into consideration. In fact, I read the paper cited here and I was appalled by what I read.<br><br>The definition of p-values given in the paper is just utterly wrong, and the rest of the paper provides very general recommendations.<br><br>I disagree with the authors here in having chosen not to correct for multiple testing. Testing was done with 7 different outcome variables at 3 different time point pairs. Meaning that 21 comparisons where done. At least one comparison is bound to be significant, just because of the alpha level = 0.05. Correction MUST be applied, by at least dividing alpha by 21, alpha_corrected = 0.0023.	We do appreciate the reviewer's opinion, but also would like to point out that there is no consensus on the issue of adjusting for multiple testing in the literature. While some experts provide compelling reasons for not adjusting [Althouse, 2016; Parker & Weir 2020], others provide a rationale for both adjusting and not adjusting [Howard, 2018; Cook and Farewell, 1996] Following the rationale provided in Althouse's points (1), (2) and (3), we decided to not adjust for multiple testing because ours is an exploratory substudy from a larger RCT. Following his recommendation we "(1) described what was done in a study; (2) reported effect sizes, confidence intervals, and p values; and (3) allow readers use their own judgment about the relative weight of the conclusions."<br><br>We have edited our statistical methods section to refer to his paper as the reference for our decision to opt out of multiple testing adjustment.

	I am not sure then what is the use of p-values if they are clearly affected by multiple testing and if they are not relevant. Perhaps it is worth dropping them altogether. Up to the authors to decide what to do.	
9	In any case, as far as I understand, t-tests were performed to test for differences between each follow- up time point. This method is not appropriate, among other things because each time-point is correlated with the next, this being a time series. This means that less inter-time-point variance is to be expected, which increases Type I error rates due to increased autocorrelation.<br><br>A better approach is to used techniques designed to work with time series data, such as generalised additive models.<br><br>If the authors don't have the skills or resources to apply such methods, I suggest them not to do significance testing at all and just report and discuss the patterns that can be observed in the plots (which is basically what is done in the paper already, so they would just have to drop the significance testing parts).	We completely agree with the reviewer and therefore we never conducted t-tests. Rather, for longitudinal outcomes, we did use methods to accommodate correlation between repeated measures. In the methods sections we state<br><br>"Longitudinal outcomes were analyzed using linear mixed analysis of repeated measures models for continuous outcomes and generalized estimating equations for count outcomes (Poisson distribution for CIS-Physical Integration and negative binomial distribution for GAIN- SS and ED Visits), assuming an unstructured correlation matrix for the repeated measures."<br><br>With respect to the reviewer's opinion about using GAM, we also note our models are valid given the design of our study and the nature of our outcomes. We chose to use these longitudinal models that are easier to interpret and communicate results.<br><br>Regarding the last comment in this set, we feel it is unnecessary and disrespectful to disparage the skills or resources of our authorship team. We are open to receiving respectful feedback and opportunities to respond to perceived limitations of our approach. Raising questions about our skills falls outside of respectful scientific dialogue.

	I am sorry that the wording was ambiguous and percieved as disrespectful. What I meant to say was that although I believe a GAM analysis would be best for this kind of data, I don't want to impose this being aware that in some cases the authors don't have access to resources or skills to apply a specific method and it would be unfair to demand that. I did not mean to disparage your skills or resources (since I have no idea what those are). I was simply not wanting to assume that you are in a position to be able to perform such analyses. (To draw a parallel from another aspect of academic writing, it is better to recommend the authors check their grammar and style rather than saying they should get a native speaker check it, because one cannot assume that the authors are not native speakers. That is where I was coming from when saying that using GAMs is not meant to be an imposition).<br><br>However, if it is the case that linear models where used to analyse the longitudinal data, then I am afraid I cannot reconstruct what was done based on the highlighted sentence. It is necessary to include a full account of how the models were constructed, in details.<br><br>If the authors do have the resources to run GAMs on the longitudinal data (as I gather from their response) then they should proceed with it.	
10	Nothing is said about where one can find the data and the analysis scripts/instructions. Please specify where these can be found.	We have answered this issue above.
	I still cannot find in the manuscript any mention to where the analysis scripts/software instructions to reproduce the analysis can be found. (I understand the data are not available, but that doesn't prevent one to share analysis scripts/instructions)	
