# Supplementary material for: Women experiencing homelessness and mental illness in a Housing First multi-site trial: Looking beyond housing to social outcomes and well-being
Source: PLoS One. 2023 Feb 10;18(2):e0277074. doi: 10.1371/journal.pone.0277074 (PMC9916643; doi:10.1371/journal.pone.0277074)
Supplement: Attachment — Submitted filename: PONE-D-21-13748_R3_reviewer.pdf [file pone.0277074.s007.pdf]

PLOS ONE

Women experiencing homelessness and mental illness in a Housing First multi-site trial: Looking beyond housing to social outcomes and well-being.

--Manuscript Draft--

**Manuscript Number:**	PONE-D-21-13748R3
**Article Type:**	Research Article
**Full Title:**	Women experiencing homelessness and mental illness in a Housing First multi-site trial: Looking beyond housing to social outcomes and well-being.
**Short Title:**	Women's experiences with Housing First
**Corresponding Author:**	Patricia O'Campo<br>St Michael's Hospital<br>Toronto, Ontario CANADA
**Keywords:**	women, homelessness, Housing First, randomized trial, mental health, suicidality, quality of life
**Abstract:**	Objective: There is scant research on the effectiveness of permanent supportive housing for homeless women with mental illness. This study examines the effectiveness of Housing First with an unprecedentedly large sample of homeless women from five Canadian cities, and explore baseline risk factors that predict social, health and well-being outcomes over a 24 month-period.<br>Methods: The At Home/Chez Soi multi-site randomized controlled Housing First trial recruited over 600 women between October 2009 and July 2011. This is a post-hoc subgroup exploratory analysis of self-identified women with at least one follow-up interview who were randomized to Housing First (HF) (n =374) or treatment-as-usual (TAU) (n =279) and had at least one follow-up interview. Linear mixed models and generalized estimating equations were used after multiple imputation was applied to address missing data.<br>Results: At the end of follow-up, the mean percentage of days spent stably housed was higher for women in the intervention 74.8% (95%CI=71.7%–77.8%) compared with women in the treatment-as-usual group, 37.9% (95%CI=34.4%–41.3%), p <0.001. With few exceptions, social and mental health outcomes were similar for both groups at 6-, 12-, 18- and 24-months post-enrollment. Suicidality was a consistent predictor of increased mental health symptoms (beta=2.85, 95% CI 1.59-4.11, p<0.001), decreased quality of life (beta=-3.99, 95% CI -6.49 to -1.49, p<0.001), decreased community functioning (beta= -1.16, 95% CI -2.10 to -0.22, p=0.015) and more emergency department visits (rate ratio = 1.44, 95% CI 1.10-1.87, p<0.001) over the study period. Lower education was a predictor of lower community functioning (beta=-1.32, 95% CI -2.27 to -0.37, p=0.006) and higher substance use problems (rate ratio=1.27, 95% CI 1.06-1.52, p=0.009) during the study.<br>Conclusions: Housing First interventions ensured that women experiencing homelessness are quickly and consistently stably housed. However, they did not differentially impact health and social measures compared to treatment as usual. Ensuring positive health and social outcomes may require greater supports at enrolment for subgroups such as those with low educational attainment, and additional attention to severity of baseline mental health challenges, such as suicidality.
**Order of Authors:**	Patricia O'Campo
	Rosane Nisenbaum
	Anne G. Crocker
	Tonia L. Nicholls
	Faith Eiboff
	Carol Adair
**Response to Reviewers:**	Please see the Response to Reviewer documents for all our responses to the Reviewer comments.
**Additional Information:**	

Powered by Editorial Manager® and ProduXion Manager® from Aries Systems Corporation

Question	Response
**Financial Disclosure**<br><br>Enter a financial disclosure statement that describes the sources of funding for the work included in this submission. Review the submission guidelines for detailed requirements. View published research articles from *PLOS ONE* for specific examples.<br><br>This statement is required for submission and **will appear in the published article** if the submission is accepted. Please make sure it is accurate.<br><br>**Unfunded studies**<br>Enter: *The author(s) received no specific funding for this work.*<br><br>**Funded studies**<br>Enter a statement with the following details:<br>• Initials of the authors who received each award<br>• Grant numbers awarded to each author<br>• The full name of each funder<br>• URL of each funder website<br>• Did the sponsors or funders play any role in the study design, data collection and analysis, decision to publish, or preparation of the manuscript?<br>• **NO** - Include this sentence at the end of your statement: *The funders had no role in study design, data collection and analysis, decision to publish, or preparation of the manuscript.*<br>• **YES** - Specify the role(s) played.<br><br>* typeset	This research has been made possible through a financial contribution from Health Canada (https://www.canada.ca/en/health-canada.html). The views expressed herein solely represent the authors. The Mental Health Commission of Canada (https://www.mentalhealthcommission.ca/English) oversaw the conduct of the study and provided training and technical support to the service teams and research staff throughout the project. However, the funder had no role in the analysis and interpretation of the data, preparation, review or approval of the manuscript.
**Competing Interests**<br><br>Use the instructions below to enter a competing interest statement for this submission. On behalf of all authors, disclose any competing interests that could be perceived to bias this work—acknowledging all financial support and any other relevant financial or non-financial competing interests.	The authors have declared that no competing interests exist.

Powered by Editorial Manager® and ProduXion Manager® from Aries Systems Corporation

This statement is required for submission and **will appear in the published article** if the submission is accepted. Please make sure it is accurate and that any funding sources listed in your Funding Information later in the submission form are also declared in your Financial Disclosure statement.<br><br>View published research articles from *PLOS ONE* for specific examples.<br><br>**NO authors have competing interests**<br><br>Enter: *The authors have declared that no competing interests exist.*<br><br>**Authors with competing interests**<br><br>Enter competing interest details beginning with this statement:<br><br>*I have read the journal's policy and the authors of this manuscript have the following competing interests: [insert competing interests here]*<br><br>* typeset	
**Ethics Statement**<br><br>Enter an ethics statement for this submission. This statement is required if the study involved:<br><br>• Human participants<br>• Human specimens or tissue<br>• Vertebrate animals or cephalopods<br>• Vertebrate embryos or tissues<br>• Field research<br><br>Write "N/A" if the submission does not require an ethics statement.<br><br>General guidance is provided below. Consult the submission guidelines for detailed instructions. **Make sure that all information entered here is included in the Methods section of the manuscript.**	Information about the study, such as the randomization process and a description of the intervention, was provided to all participants study prior to enrolment. A process was created to ensure that participants understood the complexities of the trial and follow-up activities. All participants provided informed consent prior to enrolment. All enrolment and consent documents were harmonized across sites, however, each site had a separate approvals from relevant university/site Research Ethics Boards.

Powered by Editorial Manager® and ProduXion Manager® from Aries Systems Corporation

**Format for specific study types**<br><br>**Human Subject Research (involving human participants and/or tissue)**<br>• Give the name of the institutional review board or ethics committee that approved the study<br>• Include the approval number and/or a statement indicating approval of this research<br>• Indicate the form of consent obtained (written/oral) or the reason that consent was not obtained (e.g. the data were analyzed anonymously)<br><br>**Animal Research (involving vertebrate animals, embryos or tissues)**<br>• Provide the name of the Institutional Animal Care and Use Committee (IACUC) or other relevant ethics board that reviewed the study protocol, and indicate whether they approved this research or granted a formal waiver of ethical approval<br>• Include an approval number if one was obtained<br>• If the study involved *non-human primates*, add *additional details* about animal welfare and steps taken to ameliorate suffering<br>• If anesthesia, euthanasia, or any kind of animal sacrifice is part of the study, include briefly which substances and/or methods were applied<br><br>**Field Research**<br><br>Include the following details if this study involves the collection of plant, animal, or other materials from a natural setting:<br>• Field permit number<br>• Name of the institution or relevant body that granted permission	
**Data Availability**<br><br>Authors are required to make all data underlying the findings described fully available, without restriction, and from the time of publication. PLOS allows rare exceptions to address legal and ethical concerns. See the PLOS Data Policy and FAQ for detailed information.	No - some restrictions will apply

A Data Availability Statement describing where the data can be found is required at submission. Your answers to this question constitute the Data Availability Statement and **will be published in the article**, if accepted.<br><br>**Important:** Stating 'data available on request from the author' is not sufficient. If your data are only available upon request, select 'No' for the first question and explain your exceptional situation in the text box.<br><br>Do the authors confirm that all data underlying the findings described in their manuscript are fully available without restriction?	
**Describe where the data may be found in full sentences. If you are copying our sample text, replace any instances of XXX with the appropriate details.**<br><br>• If the data are **held or will be held in a public repository**, include URLs, accession numbers or DOIs. If this information will only be available after acceptance, indicate this by ticking the box below. For example: *All XXX files are available from the XXX database (accession number(s) XXX, XXX.*).<br>• If the data are all contained **within the manuscript and/or Supporting Information files**, enter the following: *All relevant data are within the manuscript and its Supporting Information files.*<br>• If neither of these applies but you are able to provide **details of access elsewhere**, with or without limitations, please do so. For example:<br><br>*Data cannot be shared publicly because of [XXX]. Data are available from the XXX Institutional Data Access / Ethics Committee (contact via XXX) for researchers who meet the criteria for access to confidential data.*<br><br>*The data underlying the results presented in the study are available*	The data used in this paper from four of the five sites were made available for use by our team via a data sharing agreement for analysis purposes only and not for making the data publicly available. As such, data for all five sites is available from a third party. Researchers may contact the Observational Epidemiology & Qualitative Research unit at the Health Research Solutions HUB at Unity Health Toronto (https://www.hubresearch.ca/services/observational-epidemiology-qualitative-research/) where requests for the data can be submitted.

Powered by Editorial Manager® and ProduXion Manager® from Aries Systems Corporation

from (include the name of the third party and contact information or URL).<br>• This text is appropriate if the data are owned by a third party and authors do not have permission to share the data.<br><br>* typeset	
Additional data availability information:	

 Click here to access/download;Manuscript;Title Page.docx

Women experiencing homelessness and mental illness in a Housing First multi-site trial. Looking beyond housing to social outcomes and well-being.

Patricia O'Campo[1,*], Rosane Nisenbaum[1,2,¶], Anne G. Crocker[3,¶], Tonia L. Nicholls[4,¶], Faith Eiboff[5,¶], Carol Adair[6,¶]

[1]Centre for Urban Health Solutions, St Michael's Hospital, Dalla Lana School of Public Health, University of Toronto, Toronto, Ontario, Canada

[2]Applied Health Research Centre, Li Ka Shing Knowledge Institute, Toronto, Ontario, Canada

[3]Research & Academics, Institut national de psychiatrie légale Philippe-Pinel, Department of Psychiatry & Addictions, and School of Criminology, Université de Montréal, Montreal, Quebec, Canada

[4]Department of Psychiatry, University of British Columbia and BC Mental Health and Substance Use Services, Vancouver, British Columbia, Canada

[5]Interdisciplinary Studies, School of Population and Public Health, University of British Columbia, Vancouver, British Columbia, Canada

[6]Department of Psychiatry and Department of Community Health Sciences, Cumming School of Medicine, University of Calgary, Calgary, Alberta, Canada

[*]Corresponding author

E-mail: patricia.ocampo@unityhealth.to (PO)

[¶]These authors contributed equally to this work.

Manuscript

Click here to access/download;Manuscript;Manuscript PONE-D-21-13748.docx

Women experiencing homelessness and mental illness in a Housing First multi-site trial: Looking beyond housing to social outcomes and well-being.

O'Campo P., Nisenbaum R., Crocker A. G., Nicholls T., Eiboff F., Adair C.E.

O'Campo, P., Centre for Urban Health Solutions, St Michael's Hospital. Dalla Lana School of Public Health, University of Toronto

Nisenbaum, R., Centre for Urban Health Solutions, Applied Health Research Centre, Li Ka Shing Knowledge Institute, St Michael's Hospital. Dalla Lana School of Public Health, University of Toronto

Crocker A. G., Director, Research & Academics, Institut national de psychiatrie légale Philippe-Pinel & Full professor, Department of Psychiatry & Addictions, and School of Criminology, Université de Montréal

Nicholls, T. L., Department of Psychiatry, University of British Columbia and BC Mental Health and Substance Use Services

Eiboff, F., Interdisciplinary Studies, School of Population and Public Health, University of British Columbia

Adair, C.E., Department of Psychiatry and Department of Community Health Sciences, Cumming School of Medicine, University of Calgary

Abstract

Objective: There is scant research on the effectiveness of permanent supportive housing for homeless women with mental illness. This study examines the effectiveness of Housing First with an unprecedentedly large sample of homeless women from five Canadian cities, and explore baseline risk factors that predict social, health and well-being outcomes over a 24 month-period.

Methods: The At Home/Chez Soi multi-site randomized controlled Housing First trial recruited over 600 women between October 2009 and July 2011. This is a post-hoc subgroup exploratory analysis of self-identified women with at least one follow-up interview who were randomized to Housing First (HF) (n=374) or treatment-as-usual (TAU) (n=279) and had at least one follow-up interview. Linear mixed models and generalized estimating equations were used after multiple imputation was applied to address missing data.

Results: At the end of follow-up, the mean percentage of days spent stably housed was higher for women in the intervention 74.8% (95%CI=71.7%–77.8%) compared with women in the treatment-as-usual group, 37.9% (95%CI=34.4%–41.3%), $p<0.001$. With few exceptions, social and mental health outcomes were similar for both groups at 6-, 12-, 18- and 24-months post-enrollment. Suicidality was a consistent predictor of increased mental health symptoms (beta=2.85, 95% CI 1.59-4.11, p<0.001), decreased quality of life (beta=-3.99, 95% CI -6.49 to -1.49, p<0.001), decreased community functioning (beta= -1.16, 95% CI -2.10 to -0.22, p=0.015) and more emergency department visits (rate ratio = 1.44, 95% CI 1.10-1.87, p<0.001) over the study period. Lower education was a predictor of lower community functioning (beta=-1.32, 95% CI -2.27 to -0.37, p=0.006) and higher substance use problems (rate ratio=1.27, 95% CI 1.06-1.52, p=0.009) during the study.

Conclusions: Housing First interventions ensured that women experiencing homelessness are quickly and consistently stably housed. However, they did not differentially impact health and social measures compared to treatment as usual. Ensuring positive health and social outcomes may require greater supports at enrolment for subgroups such as those with low educational attainment, and additional attention to severity of baseline mental health challenges, such as suicidality.

Key Words: women, homelessness, Housing First, randomized trial, mental health, suicidality, quality of life

Trial Registration: International Standard Randomized Control Trial Number Register Identifier: ISRCTN42520374 http://www.controlled-trials.com/ISRCTN42520374.

Introduction

Homelessness in Canada is primarily experienced by men, yet data show that over one-third of the homeless people in this country are women and close to 90% of the single parents who are homeless are women[1-3]. Mental health and substance use disproportionately affect women experiencing homelessness. Between 20-50% experience major depressive disorder and major depressive episodes as demonstrated in systematic reviews [4], between 30-40% experience post-traumatic stress disorder [5, 6], as many as one in five report moderate-to-high levels of suicidality [6], and a large proportion experience exceptional vulnerability to an array of health and social risks [6-11], including premature mortality [12]. A recent meta-analysis of observational studies in high-income countries reports that three-quarter of homeless individuals have a mental illness diagnosis with no differences by gender[13].

Women who are homeless have different health and social vulnerabilities compared to homeless men. They have higher rates of distress including suicidal ideation [14], greater experiences of trauma prior to and during episodes of homelessness including more adverse childhood events (ACEs) such as physical and sexual abuse and trauma-related disorders [6, 15-22], few options for effective woman-centred services and treatments for these traumatic events and other ailments, all of which predispose them to homelessness [14, 23-27]. They are also substantially more likely than homeless men to engage in survival sex work and are thus more vulnerable to exploitation, violence, infectious disease, gynecological problems and unwanted pregnancies [6, 28]. Homeless women with children express considerable traumatic distress because of frequent child apprehensions [29]. Because women who are homeless often seek shelter in informal settings such as couch surfing or sleeping rough, they are less visible and

have less access to services [14, 30, 31] and have received less attention in research and policy and practice.

Program planners and policy makers operate in a context of too little gender-specific data on programs and interventions and weak studies addressing the experiences of women [3, 4],[32]. As such, there have been calls for more research that moves away from gender-neutral approaches (i.e., examining men's and women's experiences with the same lens, as if there are no gender differences) [14, 32-34] towards more gender-sensitive frameworks, including women-only analyses that capture the variability around the unique challenges women who are homeless face and accelerating evidence-informed practice with this population[3, 30, 35, 36]. For interventions, there is a need for studies with large enough samples to examine whether and how programs work for women[4, 37-42].

Currently, with some exceptions [43], what we do know about housing interventions for women experiencing homelessness comes from small subsamples in longitudinal observational studies, [35, 44] randomized trial pilot, [45] or qualitative evaluations [33, 46]. A large multi-site supportive housing demonstration project for chronically homeless adults in 11 US communities, with 173 women as part of the sample, reported substantial improvements in housing stability but no gender differences in this and other service utilization or clinical outcomes over two years of follow-up [47]. In an observational study of an integrated service system initiative in 18 sites across the US, women (N=2727) demonstrated greater improvements in family relationships and social support compared to men (N=4502) over 18-months of follow-up[43].

Housing First, a supportive housing approach with demonstrated success in transitioning individuals experiencing chronic homelessness and mental illness into permanent housing, has negligible evidence on outcomes for women[48-51]. The Housing First model is based on harm

reduction principles of choice and self-determination, and provides immediate access to scattered-site housing through the provision of rent supplements and flexible recovery-oriented supports[50, 52]. Participation in Housing First is not contingent on participation in services or requirements of 'housing readiness' (e.g., sobriety or medication compliance) [50].

The At Home/Chez Soi randomized trial conducted in five cities across Canada provides an unprecedented opportunity to examine the impact of a housing first initiative among women for several reasons. First, it is the largest randomized trial of a Housing First initiative and includes an unprecedented sample of several hundred women. This large sample size facilitates a woman only analysis to focus on variability within this subsample and factors that are relevant for women living with homelessness such as sources of trauma or parental roles. A woman only analysis, too rarely seen in studies of homelessness, ensures that the focus remains on women within the sample as gender analyses largely focus on comparing experiences with those of men. With 24 months of follow-up and novel risk variables not previously examined in a Housing First analysis we can provide unique point-in-time and longitudinal evidence not previously considered because of prior sample size and variable availability limitations.

The present study employs the largest longitudinal sample of women experiencing homelessness and mental illness in a Housing First randomized controlled trial (RCT) and extends the follow up duration typically seen to 24 months. Our objectives were: (i) To compare change at the end of the study in health and social outcomes between women who received and did not receive the Housing First intervention; ii) To examine whether the Housing First intervention leads to better housing stability over 24 months, and health and social and outcomes between 6- to 24-months post enrolment; and, (iii) To explore baseline demographic, social, health measures, and ACEs as predictors of key study outcomes during the study period.

Methods

Methods and measures for this analysis have been extensively described elsewhere [49, 53-55]. Briefly, the Canadian At Home/Chez Soi five city RCT randomized Housing First among adults experiencing homelessness and serious mental illness [54]. Participants randomized to the Housing First (HF) intervention received scattered-site housing and either moderate or high needs-based services to support their recovery and integration into the community (i.e., Intensive Case Management or Assertive Community Treatment). Participants randomized to treatment-as-usual (TAU) had access to housing and services through other community programs. Each site had additional focus on a unique population (e.g., ethno-racial populations in Toronto and Indigenous populations in Winnipeg) and had specialized services to address their needs. Fidelity assessments ensured strong adherence to program theory of change[56]. Information about the study, such as the randomization process and a description of the intervention, was provided to all study participants prior to enrolment. An informed consent process was created to ensure that participants had the time and information to understand the complexities of the trial and follow-up activities. All participants provided written informed consent prior to enrolment. All enrolment and consent documents were harmonized across sites, however, each site had separate approval from relevant university/site Research Ethics Boards.

Over 24 months, participants were interviewed every 3 months to assess housing stability and every 6 months to evaluate health and social outcomes. Previous results from 2,148 participants (including 683 women) demonstrated that those in the HF arm showed positive improvements in housing stability, quality of life, and community functioning compared to participants in usual care [47, 49, 53]. This paper reports on an exploratory analysis of 653

participants who self-identified as women and had at least one follow-up after their baseline assessment (96%), including 588 followed to 24 months. Across all sites, 374 participants were from the HF group and 279 from the TAU group (Supplemental Figure S1).

Outcomes and other study variables

We describe in this section the outcome variables and any other scales used in this analysis. Stable housing was defined as "living in one's own room, apartment, or house, or with family, with an expected duration of residence of 6 months or more or tenancy rights" while enrolled in the study, taking into account participants' move in/out dates for each type of residence (e.g., street place, unstable residence, stable residence, institution)[57]. The *percentage of days stably housed during the 24-month period* was calculated as the total number of days stably housed divided by the total number of days for which any type of residence data were provided by the participant over 24 months and varied between 0% and 100% [49, 54]. Unlike the other outcomes, housing stability was measured at the end of the follow-up period for several reasons. First, the treatment group establishes stable housing within the first 1-3 months of enrolment and housing status rarely changes after that point in time. A small proportion of the treatment group was not consistently stably housed throughout the duration of the program; thus, this single cumulative measure captures housing stability better than if we had measured it at each follow-up time point. In sum, this cumulative housing stability is the best measure for the outcome of housing as it does not demonstrate incremental changes over the 24-month follow-up period.

The other outcomes were assessed via in-person interviews at baseline, 6-, 12-, 18, and 24 months post-enrollment.

The Lehman Quality of Life Interview 20 (QoLI-20) measured participants' condition-specific quality of life. Twenty items scored on a 7-point Likert scale define a total quality of life and domains of family, finance, leisure, living, safety, social, and overall quality of life. In this work, we used the total score calculated as the sum of all 20 items, which ranges from 20 to 140, with higher scores indicating better quality of life [58].

Community functioning was measured using the Multnomah Community Ability Scale (MCAS), a 17-item scale capturing total community functioning and degree of clinician/observer-rated functional ability in domains of health, adaptation, social skills, and behavior. In this work, we used the total score calculated as the sum of all 17 times, ranging from 17-85, with higher scores indicating less impairment [49].

The Colorado Symptom Index (CSI) assessed how often specific psychiatric symptoms were experienced, from 'at least every day' to 'not at all'. Fourteen self-report items were summed (range 14 to 40), with lower scores reflecting fewer symptoms [59, 60].

Emergency department (ED) visits were measured using self-reported counts in the past 6 months. The number of past-month substance-related problems were measured via the 5-item Global Assessment of Individual Needs Short Screener (GAIN-SS); higher counts correspond to greater problem severity [54].

The Community Integration Scale (CIS) measured self-reported psychological integration with the immediate community on a scale from 4-20; physical integration used counts of previous month social activities (e.g., attending a concert, meeting people for coffee) on a scale of 0-7. Higher scores on both reflect greater integration [61].

Baseline and predictor variables included data collected on traumatic events that occurred before the age of 18 years using the Adverse Childhood Experiences module assessing ten early

childhood adversities (yes/no items): emotional, physical or sexual abuse; emotional or physical neglect; household substance use, household mental illness and household criminal justice involvement; mother treated violently, and parental separation or divorce [62]. Scores ranged from 0 to 10 with higher scores indicating greater childhood adversity. We created two categories representing high levels of reported ACEs, ≥5 adversities, and four or fewer including none [63, 64]. Mental health diagnoses and levels of suicidality were assessed via the MINI Neuropsychiatric Interview 6.0 administered as part of the baseline enrolment interview[65]. No, low, moderate and high levels of suicidality were dichotomized into 'no/low' and 'moderate/high' suicidality. Information about all other variables (e.g., demographics, financial support of children, sources of income) was obtained via self-report during the in-person baseline interviews.

Statistical Analyses

Descriptive statistics for a wide range of descriptors were selected to characterize the sample by treatment group. Longitudinal outcomes were analyzed using linear mixed analysis of repeated measures models for continuous outcomes and generalized estimating equations for count outcomes (Poisson distribution for CIS-Physical Integration and negative binomial distribution for GAIN-SS and ED Visits), assuming an unstructured correlation matrix for the repeated measures. Percentage of days stably housed over the 24-months follow-up (range 0-100%) was modeled as a continuous outcome in a linear model. We also performed a sensitivity analysis by considering the proportion (0-1) of days stably housed and fitting a fractional logistic model. The fractional logistic model, which uses a quasi-likelihood function for estimation, was introduced by Papke and Woolridge (1996)[66] and is appropriate for proportions when the values of 0 and 1 are admissible and observable. The fractional logistic model predicts the mean

of the proportion conditional on covariates. To ensure that the predicted mean is also between 0 and 1, the logit model for the mean is considered. The parameters estimated from this model can be exponentiated to yield odds ratios and 95% confidence intervals for the association between stable housing during the 24 months follow-up and covariates.

For the first objective, for each group, the adjusted change from baseline to 24-months follow-up was estimated by mean differences and 95% confidence intervals (continuous outcomes), and rate ratios and 95% confidence intervals (count outcomes). We fit models that included treatment group (fixed effect, HF vs TAU), categorical time (24-months vs baseline), interaction of treatment x time, and study city and need level at baseline (high vs. moderate) as covariates.

For the second objective, in a more detailed examination of possible differential effect over time, we tested for differences between HF and TAU at each follow-up time point from 6- to 24-months, adjusting for baseline outcomes. Except for percentage (or proportion) of days stably housed over 24 months, models included treatment group, categorical time (6-, 12-, 18-, 24-months), interaction of treatment x time, and city, need level and baseline outcome values as covariates. At each time point, we compared HF vs TAU by estimating mean differences and 95% confidence intervals or rate ratios and 95% confidence intervals. For percentage (or proportion) of days stably housed over 24-months we included treatment group, city, need level and percentage of days stably housed in the three months prior to baseline as covariates.

For the third objective, the differences between HF and TAU were no longer the focus. Rather we took advantage of the large sample of women in a study on homelessness to identify predictors of our outcomes. To accomplish this we expanded the models from the second

objective and added the following baseline predictors: age at enrolment (continuous), education (1=less than high school, 0=high school or higher), duration of homelessness (1=≥3 ,0= <3 years), whether minor children were being supported by the participant (1=yes, 0=no), level of suicidality (1=moderate or high, 0=mild or none), and high level of ACEs at baseline (1=≥5 , 0=<5 ACEs). These predictors were chosen because they represent a knowledge gap or have yielded inconsistent findings on longitudinal studies of women experiencing homelessness. We were unable to include ethnicity or Indigenous identity due to multicollinearity with study city.

Multiple imputation with chained equations (MICE) was used to handle missing data from loss to follow-up, withdrawal, skipped interviews, nonresponse on specific items, and systematically measured lack of interviewer confidence in participant responses. We imputed all outcomes simultaneously and MICE imputation models included all variables needed to address three objectives[67] . Although outcome missing rates varied between 0.53% to 31.9% (Supplemental Table S1), approximately 70% of the participants had at least one missing value in at least one outcome in at least one point in time. Therefore, we chose 70 imputations following guidelines given by White et al. (2010) that the percentage of cases with incomplete data should be similar to the number of imputations generated [67]. Comparisons between observed and imputed data via diagnostic plots were used to assess the quality and plausibility of the imputations[68]

Data were imputed using Stata software (mi impute chained, Stata Statistical Software: Release 13. College Station, TX: StataCorp LP) and analyzed using SAS (PROC MIXED, GENMOD , GLIMMIX, PROC MIANALYZE, SAS 9.4, SAS Institute Inc, Cary, NC). SAS Syntax is provided in Appendix X. Figures were created using the ggplot2 package in R software

version 3.4.0 (R Core Team (2017) [69]. All statistical tests were two-tailed and significance was defined as $p<.05$. No adjustments for multiple testing were applied [70].

Results

We took advantage of the rich data collected in this study and present a broad array of participant demographic, social, and health characteristics at enrolment (Table 1). Fifteen percent of the women were born outside of Canada (median time in Canada: TAU=25yrs; HF = 21yrs). Three percent of women were employed at baseline and 90% received social assistance of some type as a source of income. Around 40% (n=274) reported having minor children, and of these, 22% (n=60) were providing full or partial financial support to the child(ren). The most prevalent mental illnesses were depressive episodes, PTSD, and alcohol or substance dependence. Approximately 40% had moderate-to-high suicidality, around 40% had post-traumatic stress disorder, about 60% reported ≥5 ACEs, and the majority had three or more co-morbid medical conditions.

Table 2 displays results from models examining changes from baseline to 24 months for those in HF and TAU arms for each of our social and well-being outcomes, and comparing groups with respect to housing stability over the 24-month period. Over the 24-month follow-up period, substantial improvements were observed for all the outcomes for both HF and TAU participants. Of note, mean total QoLI-20 scores improved 16.0 points (95%CI=13.6–18.4) and 13.4 points (95%CI=10.6–16.2) in the HF and TAU groups, respectively, and number of ED visits decreased by approximately 60% in both groups.

These improvements over the 24-month period are more clearly illustrated in Figure 1 for the HF intervention and TAU groups for all social and health unadjusted outcomes at *each*

observation point from baseline to 24-months. For all outcomes, except CIS-Physical where scores remained flat throughout the follow-up, both groups experienced similar significant change from baseline to 24-months (Figure 1).

Figure 1 – Health and social outcomes among Women who are Homeless and Mentally Ill, unadjusted means and 95% confidence intervals over 24 months of follow-up by study time point and treatment group

The mean percentage of days spent stably housed during follow-up for women receiving HF was 74.8% (95%CI=71.7%–77.8%) compared with 37.9% (95%CI=34.4%–41.3%) TAU, a difference of 36.9% (95%CI=32.4%–41.4%, p<0.001). In addition, the odds of stable housing during follow-up was over 5 times higher among those in the HF group than in the TAU group (OR=5.09, 95% CI= 4.08-6.35, p<0.001). Table 3 displays changes over time in greater detail through results of the models to assessing whether social and health outcomes were better in HF vs. TAU groups *at each follow-up point*. For condition-specific quality of life, significant improvements of 7.1 and 6.1 points were observed in the HF group at the 6- and 12-month follow-ups, respectively. The HF group also showed significant improvements in psychological community integration by 0.7 points at the 6-month follow-up. However, in the last year of the trial, the ED visits fell faster for the TAU group (0.6 visits) compared to the HF group (1.1 visits) at 18 months, with a rate ratio of 1.81 (95%CI=1.26–2.60).

Table 4 no longer focuses on differences between HF and TAU as results presented here identify predictors of our outcomes. Moderate to high suicidality had the most consistent association to poorer health and service utilization outcomes at 24 months follow-up. Compared

to those with no or low suicidality at baseline, moderate-to-high suicidality at baseline was associated with nearly three points on the CSI indicating greater mental health symptom severity (beta=2.85, 95%CI=1.59–4.11, $p<0.001$), QoLI-20 mean scores were almost four points lower indicating reduced quality of life (beta=-3.99, 95%CI=-6.49 to -1.49, $p=0.002$), and almost 1.5 more ED visits (rate ratio=1.44, 95%CI=1.10–1.87, $p=0.007$). Low education was also associated with several outcomes. Compared to those with higher education, women with less than a high school level of education at baseline predicted lower community functioning (MCAS) scores (beta=-1.32, 95%CI=-2.27 to -0.37, $p=0.006$), 15% fewer social activities (CIS) in the previous month (rate ratio=0.85, 95%CI=0.77–0.93, $p<0.001$), and more severe substance-related problems at 24 months follow-up (rate ratio=1.27, 95%CI=1.06–1.52, $p=0.009$). Having been homeless for ≥3 years at baseline was associated with lower percentage days stably housed at 24-months (beta=-6.1%, 95%CI=-11.0% to -1.3%, $p=0.013$; OR=0.74, 95% CI 0.58-0.94, p=0.0132). An ACE score of 5 or more was associated with a 1.5 point increase in mental illness symptom severity (95%CI=0.25–2.76, $p=0.019$). Finally, we explored and found no evidence that baseline levels of ACEs and suicidality, separately, were effect modifiers for any of the above analyses.

Discussion

This is the first analysis of women's outcomes in an RCT of Housing First. Prior studies and trials on housing interventions for people who experience homelessness often included very small numbers of women or few variables reflecting women's unique needs and experiences that have precluded documentation of the heterogeneity of experiences and in-depth understanding of this population.

In this Canadian multi-site study, the largest longitudinal sample of women experiencing homelessness to date, the intervention group experienced housing stability for 75% of the 24-month follow-up, as compared to 38% for women in the TAU group, a finding similar to previously published reports of At Home/Chez Soi participants, the majority of whom were men [49, 53]. Even if the large proportion of time spent stably housed for women who received Housing First compared to the TAU group did not translate into greater improvements in health and social outcomes by the end of the study, there were some early differences favoring the treatment group in the first year for quality of life and psychological community integration. Immediate placement in housing and early housing stability for HF compared to TAU [71]participants likely contributed to faster improvements in these outcomes.

While ED visits declined in a similar fashion for HF and TAU over the 24-month follow-up, an unanticipated finding was a significant reduction in ED visits of 0.5 visits at 18 months for the TAU group over the HF group. For this rare count outcome, the dip in ED visits for TAU at 18 months could be a chance improvement as both groups had similar levels of ED visits by 24 months. Analyses of the full At Home/Chez Soi trial data did not show differences for this outcome[49]. One possibility is that uncertainty around continued housing as the trial was ending may have created high levels of stress in the Housing First group, but if this were the case, we would have likely seen similar changes in other study outcomes or among men in the analyses of the full sample.

Since improvements in outcomes over the 24-month period were similar for both the HF and TAU groups, we moved beyond the intention-to-treat analysis and examined baseline predictors for our outcomes across the full sample. We focused on baseline suicidality, ACEs, and parental responsibilities as predictors- variables rarely examined in past studies of supported

housing or Housing First [35, 43-45, 72, 73]. As might be expected, women with ≥5 ACEs or moderate-to-high levels of suicidality at baseline showed poorer improvement by 24 months compared to those with low ACE or suicidality scores. Moderate-to-high suicidality was a strong predictor of poorer outcomes at 24 months, including mental health, quality of life, community integration, and ED visits. Past cross-sectional studies of combined samples of homelessness men and women have demonstrated similar links between suicidality, childhood and adult trauma, substance abuse, and mental health [20, 21, 74]. However, while cross-sectional associations between suicidality and substance among women who are homeless have been reported in the literature[19, 75] our study is the first to examine these associations longitudinally.

Contrary to what we might have anticipated on the importance of ACEs in predicting multiple study outcomes given past research[76], we found that high levels of ACEs only predicted significantly worse scores on mental illness severity (CSI). This is consistent with a recent study of the full sample of At Home/Chez Soi where ACEs total scores predicted several mental health conditions at 24-months[51]. Our choice of a cutoff representing high numbers of ACEs, while informed by existing literature, may have limited our ability to detect an association. Alternatively, ACEs may be limited in capturing relevant adverse conditions. A recent study has suggested that an expanded ACEs tool might be used to capture important domains currently missing such as severe poverty or family loss and separation[77], which might be particularly relevant in capturing sources of trauma and adversity for women experiencing homelessness. Our findings also suggested that low educational attainment at baseline was a significant predictor of worse mental health, community integration, and substance use outcomes independently of the other variables in the model. Low educational attainment may be a proxy

for other factors such as low health literacy which might impact management of health conditions and requires further investigation [78]. Providing economic support for dependent children was not related to any of our study outcomes.

Although the At Home/Chez Soi support services were important for intervention participants to remain stably housed and make gains in health and social standing, these supports and services were not designed in this Housing First program to be tailored to women's unique needs. Psychological housing stability which includes considerations of safety, security and feelings of home, is particularly important for women while the At Home/Chez Soi focused predominantly on material housing stability[79]. Women who are unstably housed or homeless need services that address trauma and promote informal peer support networks [33, 80]. Although staff were trained to be responsive to individual needs, the At Home/Chez Soi intervention was not specifically designed to provide the latter two resources. In addition, more than 40% of our participants were homeless for more than three years at enrolment. Two years of stable housing with mental health supports may not have been sufficient to resolve chronic distress from being homeless with co-morbid health, trauma and substance use problems, particularly given the high rates of reported PTSD and ACEs[14].

The lack of greater improvements in the HF arm in this analysis might have been due to the intensive follow-up schedule that could have led to a Hawthorne effect among the TAU group, and higher loss to follow-up in the TAU group might have contributed to smaller differences between groups at 24 months. Despite our sample of homeless women being relatively large, it may not have been sufficient to detect small effects. The At Home/Chez Soi study did not capture gender-sensitive outcomes specific to women experiencing homelessness and therefore we may have missed important changes, such as those associated with positive

social support, increased safety from crime and victimization, or strengthening of relationships with children, family or friends[3, 81].

Our study adds to the growing yet sparse literature on the characteristics and needs of women experiencing homelessness and the effectiveness of interventions to eliminate homelessness. Given that women make up more than one third of the homeless population, and their needs differ from that of men[3, 14, 27, 32], it is important that their experiences with homelessness and recovery receive ample focus to better inform the design and implementation of interventions that meet their unique needs. Moreover, the homelessness epidemic has only worsened in the context of the COVID pandemic so the issues remain relevant and these new analyses can provide insights[82].

References

1. Gaetz S, Dej E, Richter T, Redman M. The State of Homelessness in Canada 2016. Toronto: 2016.
2. Employment and Social Development Canada. Everyone Counts 2018: Highlights - Preliminary results from the second nationally coordinated Point-in-Time count of homelessness in Canadian communities. 2019.
3. Schwan K, Versteegh A, Perri M, Caplan R, Baig K, Dej E, et al. The State of Women's Housing Need & Homelessness in Canada: A Literature Review. Hache, A., Nelson, A., Kratochvil, E., & Malenfant, J. (Eds). Toronto, ON: Canadian Observatory on Homelessness Press. 2020.
4. Duke A, Searby A. Mental Ill Health in Homeless Women: A Review. Issues Ment Health Nurs. 2019;40(7):605-12. doi: 10.1080/01612840.2019.1565875. PubMed PMID: WOS:000474047300001.
5. Ferguson KM, Bender K, Thompson SJ. Gender, coping strategies, homelessness stressors, and income generation among homeless young adults in three cities. Social science & medicine (1982). 2015;135:47-55. Epub 2015/05/06. doi: 10.1016/j.socscimed.2015.04.028. PubMed PMID: 25942470.
6. Strehlau V, Torchalla I, Kathy L, Schuetz C, Krausz M. Mental health, concurrent disorders, and health care utilization in homeless women. Journal of psychiatric practice. 2012;18(5):349-60. Epub 2012/09/22. doi: 10.1097/01.pra.0000419819.60505.dc. PubMed PMID: 22995962.
7. Bassuk EL, Buckner JC, Perloff JN, Bassuk SS. Prevalence of mental health and substance use disorders among homeless and low-income housed mothers. The American journal of psychiatry. 1998;155(11):1561-4. Epub 1998/11/13. doi: 10.1176/ajp.155.11.1561. PubMed PMID: 9812118.
8. Shinn M, Knickman JR, Weitzman BC. Social relationships and vulnerability to becoming homeless among poor families. The American psychologist. 1991;46(11):1180-7. Epub 1991/11/01. PubMed PMID: 1772155.
9. Banyard VL, Graham-Bermann SA. Surviving poverty: stress and coping in the lives of housed and homeless mothers. The American journal of orthopsychiatry. 1998;68(3):479-89. Epub 1998/08/01. PubMed PMID: 9686299.
10. Nelson G, Patterson M, Kirst M, Macnaughton E, Isaak CA, Nolin D, et al. Life changes among homeless persons with mental illness: a longitudinal study of housing first and usual treatment. Psychiatric services (Washington, DC). 2015;66(6):592-7. Epub 2015/02/18. doi: 10.1176/appi.ps.201400201. PubMed PMID: 25686813.
11. Riley ED, Weiser SD, Sorensen JL, Dilworth S, Cohen J, Neilands TB. Housing Patterns and Correlates of Homelessness Differ by Gender among Individuals Using San Francisco Free Food Programs. Journal of Urban Health. 2007;84(3):415-22. doi: 10.1007/s11524-006-9153-3.
12. Cheung AM, Hwang SW. Risk of death among homeless women: a cohort study and review of the literature. CMAJ : Canadian Medical Association journal = journal de l'Association medicale canadienne. 2004;170(8):1243-7. Epub 2004/04/14. PubMed PMID: 15078846; PubMed Central PMCID: PMCPMC385354.
13. Gutwinski S, Schreiter S, Deutscher K, Fazel S. The prevalence of mental disorders among homeless people in high-income countries: An updated systematic review and meta-regression analysis. PLoS Med. 2021;18(8):e1003750. Epub 2021/08/24. doi: 10.1371/journal.pmed.1003750. PubMed PMID: 34424908; PubMed Central PMCID: PMCPMC8423293.
14. Milaney K, Williams N, Lockerbie SL, Dutton DJ, Hyshka E. Recognizing and responding to women experiencing homelessness with gendered and trauma-informed care. Bmc Public Health. 2020;20(1). doi: 10.1186/s12889-020-8353-1. PubMed PMID: WOS:000522413900001.
15. Brunette M, Drake RE. Gender differences in homeless persons with schizophrenia and substance abuse. Community mental health journal. 1998;34(6):627-42. Epub 1998/12/02. PubMed PMID: 9833202.

16. Rich AR, Clark C. Gender differences in response to homelessness services. Evaluation and Program Planning. 2005;28:69-81.
17. Roll CN, Toro PA, GL. O. Characteristics and experiences of homeless adults: A comparison of single men, single women, and women with children. Journal of Community Psychology. 1999;27:189-98.
18. Torchalla I, Strehlau V, Li K, Aube Linden I, Noel F, Krausz M. Posttraumatic stress disorder and substance use disorder comorbidity in homeless adults: Prevalence, correlates, and sex differences. Psychology of addictive behaviors : journal of the Society of Psychologists in Addictive Behaviors. 2014;28(2):443-52. Epub 2013/08/07. doi: 10.1037/a0033674. PubMed PMID: 23915373.
19. Benda BB. Gender differences in predictors of suicidal thoughts and attempts among homeless veterans that abuse substances. Suicide & life-threatening behavior. 2005;35(1):106-16. Epub 2005/04/22. doi: 10.1521/suli.35.1.106.59262. PubMed PMID: 15843327.
20. Desai RA, Liu-Mares W, Dausey DJ, Rosenheck RA. Suicidal ideation and suicide attempts in a sample of homeless people with mental illness. The Journal of nervous and mental disease. 2003;191(6):365-71. Epub 2003/06/27. doi: 10.1097/01.nmd.0000071584.88965.e1. PubMed PMID: 12826917.
21. Eynan R, Langley J, Tolomiczenko G, Rhodes AE, Links P, Wasylenki D, et al. The association between homelessness and suicidal ideation and behaviors: results of a cross-sectional survey. Suicide & life-threatening behavior. 2002;32(4):418-27. Epub 2002/12/28. PubMed PMID: 12501966.
22. Rodriguez-Moreno S, Vázquez JJ, Roca P, Panadero S. Differences in stressful life events between men and women experiencing homelessness. Journal of Community Psychology. 2021;49(2):375-89. doi: https://doi.org/10.1002/jcop.22465.
23. Herman DB, Susser ES, Struening EL, Link BL. Adverse childhood experiences: are they risk factors for adult homelessness? American journal of public health. 1997;87(2):249-55. Epub 1997/02/01. PubMed PMID: 9103105; PubMed Central PMCID: PMCPMC1380802.
24. Montgomery AE, Cutuli JJ, Evans-Chase M, Treglia D, Culhane DP. Relationship among adverse childhood experiences, history of active military service, and adult outcomes: homelessness, mental health, and physical health. American journal of public health. 2013;103 Suppl 2:S262-8. Epub 2013/10/24. doi: 10.2105/ajph.2013.301474. PubMed PMID: 24148064; PubMed Central PMCID: PMCPMC3969137.
25. Roos LE, Mota N, Afifi TO, Katz LY, Distasio J, Sareen J. Relationship between adverse childhood experiences and homelessness and the impact of axis I and II disorders. American journal of public health. 2013;103 Suppl 2:S275-81. Epub 2013/10/24. doi: 10.2105/ajph.2013.301323. PubMed PMID: 24148049; PubMed Central PMCID: PMCPMC3969113.
26. Patterson ML, Moniruzzaman A, Somers JM. Setting the stage for chronic health problems: cumulative childhood adversity among homeless adults with mental illness in Vancouver, British Columbia. BMC Public Health. 2014;14:350. Epub 2014/04/15. doi: 10.1186/1471-2458-14-350. PubMed PMID: 24726046; PubMed Central PMCID: PMCPMC3991866.
27. Schwan K, Vaccaro M, Reid L, Ali N, Baig K. The Pan-Canadian Women's Housing & Homelessness Survey. Toronto, ON: Canadian Observatory on Homelessness, 2021.
28. Milaney K, Williams N, Lockerbie SL, Dutton DJ, Hyshka E. Recognizing and responding to women experiencing homelessness with gendered and trauma-informed care. BMC Public Health. 2020;20(1):397. Epub 2020/03/29. doi: 10.1186/s12889-020-8353-1. PubMed PMID: 32216770; PubMed Central PMCID: PMCPMC7099814.
29. Torchalla I, Linden IA, Strehlau V, Neilson EK, Krausz M. "Like a lots happened with my whole childhood": violence, trauma, and addiction in pregnant and postpartum women from Vancouver's Downtown Eastside. Harm Reduction Journal. 2015;12(1):1. doi: 10.1186/1477-7517-12-1.

30. Shier ML, Jones ME, Graham JR. Sociocultural Factors to Consider When Addressing the Vulnerability of Social Service Users: Insights From Women Experiencing Homelessness. Affilia. 2011;26(4):367-81. doi: 10.1177/0886109911428262.
31. Winetrobe H, Wenzel S, Rhoades H, Henwood B, Rice E, Harris T. Differences in Health and Social Support between Homeless Men and Women Entering Permanent Supportive Housing. Women's Health Issues. 2017;27(3):286-93. doi: https://doi.org/10.1016/j.whi.2016.12.011.
32. Perri M, O'Campo P. A gap in knowledge surrounding urban housing interventions: a call for gender redistribution. Health Promot Int. 2021;36(4):908-12. Epub 2021/05/12. doi: 10.1093/heapro/daab062. PubMed PMID: 33975348.
33. Oudshoorn A, Forchuk C, Hall J, Smith-Carrier T, Van Berkum A. An evaluation of a Housing First program for chronically homeless women. Journal of Social Inclusion. 2018;9(2):34-50. doi: 10.36251/josi.136. PubMed PMID: WOS:000454531300004.
34. Perri M, Metheny N, Matheson FI, Potvin K, O'Campo P. Finding opportunity in the COVID-19 crisis: prioritizing gender in the design of social protection policies. Health Promot Int. 2022;37(1). Epub 2021/05/17. doi: 10.1093/heapro/daab045. PubMed PMID: 33993289; PubMed Central PMCID: PMCPMC8194510.
35. Cameron A, Abrahams H, Morgan K, Williamson E, Henry L. From pillar to post: homeless women's experiences of social care. Health Soc Care Community. 2016;24:345-32.
36. Fotheringham S, Walsh CA, Burrowes A. 'A place to rest': the role of transitional housing in ending homelessness for women in Calgary, Canada. Gender, Place & Culture. 2014;21(7):834-53. doi: 10.1080/0966369X.2013.810605.
37. Blehar MC. Public health context of women's mental health research. The Psychiatric clinics of North America. 2003;26(3):781-99. Epub 2003/10/18. doi: 10.1016/s0193-953x(03)00039-x. PubMed PMID: 14563109.
38. Institute of Medicine. Sex-Specific Reporting of Scientific Research: A Workshop Summary. Washington, DC: The National Academies Press. https://doi.org/10.17226/13307. 2012.
39. Jahn I, Bornhorst C, Gunther F, Brand T. Examples of sex/gender sensitivity in epidemiological research: results of an evaluation of original articles published in JECH 2006-2014. Health research policy and systems. 2017;15(1):11. Epub 2017/02/17. doi: 10.1186/s12961-017-0174-z. PubMed PMID: 28202078; PubMed Central PMCID: PMCPmc5312447.
40. Klap R, Humphreys K. Designing Studies for Sex and Gender Analyses: How Research Can Derive Clinically Useful Knowledge for Women's Health. Women's health issues : official publication of the Jacobs Institute of Women's Health. 2019;29 Suppl 1:S12-s4. Epub 2019/06/30. doi: 10.1016/j.whi.2019.05.002. PubMed PMID: 31253235.
41. Welch V, Doull M, Yoganathan M, Jull J, Boscoe M, Coen SE, et al. Reporting of sex and gender in randomized controlled trials in Canada: a cross-sectional methods study. Res Integr Peer Rev. 2017;2:15-. doi: 10.1186/s41073-017-0039-6. PubMed PMID: 29451565.
42. Tsai J, Byrne TH. National Utilization Patterns of Veterans Affairs Homelessness Programs in the Era of Housing First. Psychiatric Services. 2019;70(4):309-15. doi: 10.1176/appi.ps.201800393. PubMed PMID: WOS:000462848600008.
43. Cheng AL, Kelly PJ. Impact of an integrated service system on client outcomes by gender in a national sample of a mentally ill homeless population. Gender medicine. 2008;5(4):395-404. Epub 2008/12/26. doi: 10.1016/j.genm.2008.10.003. PubMed PMID: 19108812.
44. Nesse L, Gonzalez Marianne T, Aamodt G, Raanaas Ruth K. Recovery, quality of life and issues in supported housing among residents with co-occurring problems: a cross-sectional study. Advances in Dual Diagnosis. 2020;13(2):73-87. doi: 10.1108/ADD-10-2019-0014.
45. Tinland A, Zemmour K, Auquier P, Boucekine M, Girard V, Loubiere S, et al. Homeless women with schizophrenia reported lower adherence to their medication than men: results from the French

Housing First experience. Social Psychiatry and Psychiatric Epidemiology. 2017;52(9):1113-22. doi: 10.1007/s00127-017-1411-z. PubMed PMID: WOS:000408956600005.
46. Adame AL, Perry C, Pierce E. Community and Housing First: A qualitative analysis of USA residents' perspectives. Health Soc Care Community. 2020;28(4):1292-300. Epub 2020/03/05. doi: 10.1111/hsc.12962. PubMed PMID: 32128910.
47. Edens EL, Mares AS, Rosenheck RA. Chronically homeless women report high rates of substance use problems equivalent to chronically homeless men. Women's health issues : official publication of the Jacobs Institute of Women's Health. 2011;21(5):383-9. Epub 2011/06/28. doi: 10.1016/j.whi.2011.03.004. PubMed PMID: 21703865.
48. Aubry T, Tsemberis S, Adair CE, Veldhuizen S, Streiner D, Latimer E, et al. One-Year Outcomes of a Randomized Controlled Trial of Housing First With ACT in Five Canadian Cities. Psychiatric Services. 2015;66(5):463-9. doi: 10.1176/appi.ps.201400167.
49. Stergiopoulos V, Hwang SW, Gozdzik A, Nisenbaum R, Latimer E, Rabouin D, et al. Effect of scattered-site housing using rent supplements and intensive case management on housing stability among homeless adults with mental illness: a randomized trial. Jama. 2015;313(9):905-15. Epub 2015/03/04. doi: 10.1001/jama.2015.1163. PubMed PMID: 25734732.
50. Tsemberis S, Gulcur L, Nakae M. Housing First, consumer choice, and harm reduction for homeless individuals with a dual diagnosis. American journal of public health. 2004;94(4):651-6. Epub 2004/04/01. PubMed PMID: 15054020; PubMed Central PMCID: PMCPmc1448313.
51. Liu M, Mejia-Lancheros C, Lachaud J, Latimer E, Aubry T, Somers J, et al. Overall and Gender-specific Associations between Dimensions of Adverse Childhood Experiences and Mental Health Outcomes among Homeless Adults: Associations Générales et Sexospécifiques Entre les Dimensions des Expériences Défavorables de L'enfance et les Résultats de Santé Mentale Chez les Adultes Sans Abri. Can J Psychiatry. 2021;66(10):906-17. Epub 2021/01/28. doi: 10.1177/0706743721989158. PubMed PMID: 33502253; PubMed Central PMCID: PMCPMC8573704.
52. Paquette K, Pannella Winn LA. The Role of Recovery Housing: Prioritizing Choice in Homeless Services. Journal of dual diagnosis. 2016;12(2):153-62. Epub 2016/04/12. doi: 10.1080/15504263.2016.1175262. PubMed PMID: 27064834.
53. Aubry T, Goering P, Veldhuizen S, Adair CE, Bourque J, Distasio J, et al. A Multiple-City RCT of Housing First With Assertive Community Treatment for Homeless Canadians With Serious Mental Illness. Psychiatric services (Washington, DC). 2016;67(3):275-81. Epub 2015/12/02. doi: 10.1176/appi.ps.201400587. PubMed PMID: 26620289.
54. Goering PN, Streiner DL, Adair C, Aubry T, Barker J, Distasio J, et al. The At Home/Chez Soi trial protocol: a pragmatic, multi-site, randomised controlled trial of a Housing First intervention for homeless individuals with mental illness in five Canadian cities. BMJ open. 2011;1(2):e000323. Epub 2011/11/22. doi: 10.1136/bmjopen-2011-000323. PubMed PMID: 22102645; PubMed Central PMCID: PMCPmc3221290.
55. Aquin JP, Roos LE, Distasio J, Katz LY, Bourque J, Bolton JM, et al. Effect of Housing First on Suicidal Behaviour: A Randomised Controlled Trial of Homeless Adults with Mental Disorders. Can J Psychiatry. 2017;62(7):473-81. Epub 2017/07/07. doi: 10.1177/0706743717694836. PubMed PMID: 28683228; PubMed Central PMCID: PMCPMC5528985.
56. Nelson G, Macnaughton E, Caplan R, MacLeod T, Townley G., Piat M, et al. Follow-up Implementation and fidelity Evaluation of the mental Health Commission of Canada's At Home/Chez Soi Project: Cross-site Report. Mental Health Commission of Canada, 2013.
57. Tsemberis S., McHugo G., Williams V., Hanrahan P., Stefancic A. Measuring homelessness and residential stability: The residential time-line follow-back inventory. Journal of Community Psychology. 2007;35:29-42.

58. Uttaro T., Lehman A. Graded response modeling of the Quality of Life Interview. Evaluation and Program Planning. 1999;22:41-52.
59. Barker S, Barron N, McFarland BH, Bigelow DA, Carnahan T. A community ability scale for chronically mentally ill consumers: Part II. Applications. Community Ment Health Journal. 1994;30(5):459-72.
60. Dickerson FB, Origoni AE, Pater A, Friedman BK, Kordonski WM. An expanded version of the Multnomah Community Ability Scale: anchors and interview probes for the assessment of adults with serious mental illness. Community Ment Health Journal. 2003;39(2):131-7.
61. Conrad KJ, Yagelka JR, Matters MD, Rich A.R., Williams V., Buchanan M. Reliability and validity of a modified Colorado Symptom Index in a national homeless sample. Mental Health Services Research. 2001;3:141-53.
62. Felitti VJ, Anda RF, Nordenberg D, Williamson DF, Spitz AM, Edwards V, et al. Relationship of childhood abuse and household dysfunction to many of the leading causes of death in adults. The Adverse Childhood Experiences (ACE) Study. Am J Prev Med. 1998;14(4):245-58. Epub 1998/06/23. doi: 10.1016/s0749-3797(98)00017-8. PubMed PMID: 9635069.
63. Chapman DP, Whitfield CL, Felitti VJ, Dube SR, Edwards VJ, Anda RF. Adverse childhood experiences and the risk of depressive disorders in adulthood. J Affect Disord. 2004;82(2):217-25. Epub 2004/10/19. doi: 10.1016/j.jad.2003.12.013. PubMed PMID: 15488250.
64. Messina N, Grella C. Childhood trauma and women's health outcomes in a California prison population. American journal of public health. 2006;96(10):1842-8. Epub 2006/09/30. doi: 10.2105/ajph.2005.082016. PubMed PMID: 17008581; PubMed Central PMCID: PMCPMC1586137.
65. Lecrubier Y, Sheehan DV, Weiller E, Amorim P, Bonora I, Harnett Sheehan K, et al. The Mini International Neuropsychiatric Interview (MINI). A short diagnostic structured interview: reliability and validity according to the CIDI. European Psychiatry. 1997;12(5):224-31. doi: https://doi.org/10.1016/S0924-9338(97)83296-8.
66. Papke LE, Wooldridge JM. Econometric Methods for Fractional Response Variables With an Application to 401 (K) Plan Participation Rates. Journal of Applied Econometrics. 1996;11(6):619-32.
67. White IR, Royston P, Wood AM. Multiple imputation using chained equations: Issues and guidance for practice. Statistics in Medicine 2010;30:377-99.
68. van Buuren S. Flexible Imputation of Missing Data: CRC Press; 2012.
69. R: A language and environment for statistical computing. R Foundation for Statistical Computing V, Austria (https://www.R-project.org/).
70. Streiner DL, Norman GR. Correction for multiple testing: is there a resolution? . Chest. 2011;140(1):16-18.
71. Aubry T, Nelson G, Tsemberis S. Housing First for People with Severe Mental Illness Who are Homeless: A Review of the Research and Findings from the at Home—Chez soi Demonstration Project. The Canadian Journal of Psychiatry. 2015;60(11):467-74. doi: 10.1177/070674371506001102.
72. Hall G, Walters S, Gould H, Lim S. Housing versus treatment first for supportive housing participants with substance use disorders: A comparison of housing and public service use outcomes. Subst Abus. 2020;41(1):70-6. Epub 2018/03/13. doi: 10.1080/08897077.2018.1449049. PubMed PMID: 29528786.
73. Clifasefi SL, Collins SE. The life-enhancing alcohol-management program: Results from a 6-month nonrandomized controlled pilot study assessing a community based participatory research program in housing first. J Community Psychol. 2020;48(3):763-76. Epub 2019/11/30. doi: 10.1002/jcop.22291. PubMed PMID: 31778585.
74. Dietz TL. Substance Misuse, Suicidal Ideation, and Suicide Attempts Among a National Sample of Homeless. Journal of Social Service Research. 2010;37(1):1-18. doi: 10.1080/01488376.2011.524511.

75. Guillén AI, Marín C, Panadero S, Vázquez JJ. Substance use, stressful life events and mental health: A longitudinal study among homeless women in Madrid (Spain). Addictive Behaviors. 2020;103:106246. doi: https://doi.org/10.1016/j.addbeh.2019.106246.
76. Edalati H, Nicholls TL, Schütz CG, Somers JM, Distasio J, Aubry T, et al. Examining the Relationships between Cumulative Childhood Adversity and the Risk of Criminal Justice Involvement and Victimization among Homeless Adults with Mental Illnesses after Receiving Housing First Intervention. Can J Psychiatry. 2020;65(6):409-17. Epub 2020/01/30. doi: 10.1177/0706743720902616. PubMed PMID: 31994918; PubMed Central PMCID: PMCPMC7265607.
77. Mersky JP, Janczewski CE, Topitzes J. Rethinking the Measurement of Adversity. Child Maltreat. 2017;22(1):58-68. Epub 2016/12/07. doi: 10.1177/1077559516679513. PubMed PMID: 27920222.
78. Farrell SJ, Dunn M, Huff J. Examining Health Literacy Levels in Homeless Persons and Vulnerably Housed Persons with Mental Health Disorders. Community mental health journal. 2020;56(4):645-51. Epub 2019/12/21. doi: 10.1007/s10597-019-00525-2. PubMed PMID: 31858332.
79. O'Campo P, Daoud N, Hamilton-Wright S, Dunn J. Conceptualizing Housing Instability: Experiences with Material and Psychological Instability Among Women Living with Partner Violence. Housing Studies. 2016;31(1):1-19. doi: 10.1080/02673037.2015.1021768.
80. Hopper EK, Bassuk EL, Olivet J. Shelter from the storm: Trauma-informed care in homelessness services settings. The Open Health Services and Policy Journal. 2010;3(2), 80-100.
81. Bingham B, Moniruzzaman A, Patterson M, Sareen J, Distasio J, O'Neil J, et al. Gender differences among Indigenous Canadians experiencing homelessness and mental illness. BMC psychology. 2019;7(1):57. Epub 2019/08/29. doi: 10.1186/s40359-019-0331-y. PubMed PMID: 31455404; PubMed Central PMCID: PMCPmc6712855.
82. Yakubovich AR, Maki K. Preventing Gender-Based Homelessness in Canada During the COVID-19 Pandemic and Beyond: The Need to Account for Violence Against Women. Violence Against Women. 2021:10778012211034202. doi: 10.1177/10778012211034202.

Table 1: Baseline characteristics of women participants in the Canadian At Home/Chez Soi study by randomization group (*N* = 653)

Characteristics	Treatment as Usual (N = 279)		Housing First (N = 374)	
	N	%	N	%
Age (M ± SD), y	40.2 ± 11.7		39.7 ± 11.2	
Ethnic or cultural identity				
Aboriginal	76	27.2	95	25.4
Ethno-racial	67	24.0	81	21.7
White	136	48.8	198	52.9
Education				
Less than high school	130	46.9	202	54.3
Completed high school	57	20.6	61	16.4
Some post-secondary school	90	32.5	109	29.3
Marital status				
Married/Partnered	22	7.9	16	4.3
Divorced/Separated/Widowed	74	26.5	106	28.4
Single/Never married	183	65.6	251	67.3
Country of birth, & length of time in Canada for immigrants				
Born in Canada	235	84.8	315	84.2
Immigrant, 10 years or less	8	2.8	12	3.2

Characteristics	Treatment as Usual (N = 279)		Housing First (N = 374)	
	N	%	N	%
Immigrant, more than 10 years	34	12.3	47	12.6
Employment status				
Unemployed (unemployed, volunteer work, retired, student, housewife/husband, other)	270	96.8	362	97.3
Employed (including self-employed and special work program)	9	3.2	10	2.7
Sources of income				
Earnings from regular/casual work or busking	16	5.7	18	4.8
Unemployment insurance	22	7.9	26	7.0
Social assistance (disability, welfare, pension, personal needs allowance)	245	87.8	338	90.4
Selling papers, crafts, pan-handling, squeegee, or $ from recycling (bottles, scrap metal)	16	5.7	22	5.9
Other (loans, theft, sex work, help from family or friends)	12	4.3	10	2.7
Housing status				
Absolutely Homeless	215	77.1	296	79.1
Precariously housed	64	22.9	78	20.9
Lifetime duration of homelessness (M ± SD)[a], y	3.8 ± 4.6		3.8 ± 4.8	

Characteristics	Treatment as Usual (N = 279)		Housing First (N = 374)	
	N	%	N	%
Less than 3 years duration	155	57.8	211	58.0
3 years or more duration	113	42.2	153	42.0
Has minor children (≤18 yrs of age)				
0	154	56.0	221	59.1
1-2	81	29.4	110	29.4
3 or more	40	14.6	43	11.5
Has minor children (≤18 yrs of age) & provides full/partial support				
0	88	72.7	125	82.2
1	20	16.5	16	10.5
2 or more	13	10.7	11	7.2
Relationship to minor children (≤ 18 yrs of age)				
Biological parent	121	100.0	151	98.7
Partner's, adoptive or other adult relative's child	0	0	5	3.4
MCAS[b] score (M ± SD)	60.7 ± 8.0		61.8 ± 7.8	
MINI[c] diagnostic categories				
Depressive episode	170	60.9	218	58.3
Manic or hypomanic episode	50	17.9	55	14.7
Post-traumatic stress disorder	126	45.3	145	38.8
Panic disorder	94	33.7	102	27.3

Characteristics	Treatment as Usual (N = 279)		Housing First (N = 374)	
	N	%	N	%
Mood disorder with psychotic features	54	19.4	54	14.5
Psychotic disorder	81	29.0	95	25.4
Alcohol dependence	97	34.8	108	28.9
Substance dependence	134	48.0	167	44.7
Suicidality				
Not suicidal	46	16.5	62	16.6
Low	119	42.7	170	45.5
Moderate	50	17.9	70	18.7
High	64	22.9	72	19.3
Adverse Childhood Experiences Scale, mean (SD)	5.1	3.0	5.2	3.2
Less than 5	95	39.9	137	40.5
5 or more	143	60.1	201	59.5
Comorbid medical conditions				
Less than 3	51	18.6	100	27.0
3 or more	223	81.4	270	73.0
Study Component				
Moderate Needs	144	51.6	232	62.0
High Needs	135	48.4	142	38.0

[a] Median (IQR), y 2.0 (0.8-5.0) for Treatment as Usual and 2.0 (0.7-5.0) for Housing First ; [b] MCAS: Multnomah Community Ability Scale. Possible scores range from 17 to 85, with lower scores indicating more disability.;

[c] MINI: The Mini-International Neuropsychiatric Interview

Table 2. Post-Imputation Pooled change in health and social measures from Baseline to 24 Months among Women living with Mental illness and Homelessness, Adjusted for Study City, and Need Level (ACT or ICM).

	**Housing First (HF) (N=374)**		**Treatment as Usual (TAU) (N=279)**		**p-value for differences between HF and TAU**
**Continuous Outcomes (95% CI)**	Mean Change from Baseline to 24 Months	95% CI	Mean Change from Baseline to 24 Months	95% CI	p
Mental illness symptom severity (CSI[a])	-6.3	-7.5 to -5.0	-6.9	-8.4 to -5.4	0.534
Community functioning (MCAS[b])	3.8	2.8 - 4.9	4.8	3.6 - 6.0	0.236
Condition-specific QoL (QoLI-20[c])	16.0	13.6 - 18.4	13.4	10.6 - 16.2	0.178
Psychological community integration (CIS[d])	2.0	1.5 - 2.4	2.0	1.4 - 2.6	0.941

**Count Outcomes**	Rate Ratio Comparing 24 Months with Baseline	95% CI	Rate Ratio Comparing 24 Months with Baseline	95% CI	p
Physical community integration in past month (CIS[d])	0.97	0.86 - 1.08	1.03	0.92 - 1.14	0.439
Severity of substance problems in past month (GAIN-SS[e])	0.79	0.67 - 0.93	0.83	0.70 - 0.98	0.688
Number of emergency department visits in past 6 months	0.38	0.29 - 0.50	0.37	0.27 - 0.51	0.926

Note. Housing outcome is mean proportion of days spent stably housed.

[a] CSI: Colorado Symptom Index. Possible scores range from 14 to 70, with higher values indicating greater symptom severity.

[b] MCAS: Multnomah Community Ability Scale. Possible scores range from 17 to 85, with lower values indicating more disability.

[c] QoLI-20: Lehman Quality of Life Interview 20. Possible scores range from 20 to 140, with higher values indicating greater quality of life.

[d] CIS: Community Integration Scale. Possible scores range from 4 to 20 for the Psychological component and 0-7 for the Physical component, with higher values indicating greater integration.

[e] GAIN-SS: Global Assessment of Individual Needs Short Screener. Possible scores range from 0 to 5 with higher values indicating greater problem severity.

Table 3. Post-Imputation Pooled Comparisons between Housing First and Treatment as Usual at 6, 12, 18 and 24 months for Women in the At Home/Chez Soi Trial Adjusted for Study City, Need Level (ICM or ACT) and Baseline Outcome Values[f]

	Month	Housing First		Treatment as Usual		Comparison		
**Continuous Outcome**		**Mean**	**95% CI**	**Mean**	**95% CI**	**Mean Difference**	**95% CI**	**p**
Mental illness symptom severity (CSI[a])	6	35.8	34.8 - 36.8	36.8	35.6 - 38.0	-1.0	-2.5 to 0.5	0.183
	12	34.6	33.6 - 35.6	35.3	34.1 - 36.6	-0.7	-2.3 to 0.8	0.364
	18	33.8	32.7 - 35.0	34.1	32.7 - 35.4	-0.2	-2.0 to 1.5	0.785
	24	33.7	32.6 - 34.8	33.4	32.1 - 34.7	0.3	-1.4 to 2.0	0.737
Community functioning (MCAS[b])	6	64.9	64.1 - 65.7	64.9	64.0 - 65.9	-0.02	-1.2 to 1.2	0.975
	12	65.0	64.2 - 65.8	64.3	63.4 - 65.3	0.7	-0.6 to 1.9	0.299
	18	65.6	64.8 - 66.5	64.9	63.8 - 65.9	0.8	-0.6 to 2.1	0.267

	24	65.5	64.7 - 66.4	65.9	64.9 - 66.9	-0.4	-1.7 to 0.9	0.593
Condition-specific QoL (QoLI-20[c])	6	84.4	82.4 - 86.4	77.3	75.0 - 79.6	7.1	4.1 - 10.1	<.001
	12	87.4	85.4 - 89.4	81.3	79.0 - 83.7	6.1	3.1 - 9.2	<.001
	18	84.8	82.8 - 86.9	82.2	79.9 - 84.6	2.6	-0.4 to 5.6	0.091
	24	86.8	84.6 - 89.0	84.3	81.8 - 86.7	2.5	-0.7 to 5.8	0.124
Psychological community integration (CIS[d])	6	12.4	12.0 - 12.7	11.8	11.4 - 12.3	0.5	-0.1 to 1.1	0.077
	12	12.8	12.4 - 13.2	12.1	11.6 - 12.5	0.7	0.1 to 1.3	0.017
	18	12.7	12.3 - 13.1	12.3	11.8 - 12.7	0.4	-0.2 to 1.0	0.194
	24	12.9	12.5 - 13.2	12.7	12.2 - 13.1	0.2	-0.4 to 0.8	0.458
**Count Outcome**		**Rate**	**95% CI**	**Rate**	**95% CI**	**Rate Ratio**	**95% CI**	**p**
Physical community integration in past month (CIS[d])	6	2.1	1.9 - 2.3	2.1	1.9 - 2.3	0.98	0.87 - 1.12	0.789

	12	1.9 1.7 - 2.1	2.0 1.8 - 2.2	0.95 0.84 - 1.09	0.492
	18	2.0 1.8 - 2.1	2.0 1.8 - 2.2	0.99 0.87 - 1.12	0.858
	24	1.9 1.7 - 2.0	2.0 1.8 - 2.2	0.91 0.80 - 1.03	0.148
Severity of substance problems in past month (GAIN-SS[e])	6	1.2 1.0 - 1.4	1.2 1.0 - 1.5	0.97 0.78 - 1.20	0.767
	12	1.3 1.1 - 1.5	1.2 1.0 - 1.4	1.09 0.87 - 1.37	0.452
	18	1.1 0.9 - 1.3	1.2 0.9 - 1.4	0.74 1.20 - 0.63	0.628
	24	1.1 0.9 - 1.2	1.0 0.9 - 1.2	1.00 0.79 - 1.27	0.977
Number of emergency department visits in past 6 months	6	1.2 0.9 - 1.4	1.4 1.0 - 1.9	0.80 0.55 - 1.16	0.241
	12	1.1 0.7 - 1.4	1.1 0.7 - 1.6	0.96 0.58 - 1.57	0.858
	18	1.1 0.8 - 1.4	0.6 0.5 - 0.8	1.81 1.26 - 2.60	0.001
	24	0.8 0.6 - 0.9	0.7 0.5 - 0.9	1.13 0.83 - 1.55	0.426

a CSI: Colorado Symptom Index. Possible scores range from 14 to 70, with higher values indicating greater symptom severity.

b MCAS: Multnomah Community Ability Scale. Possible scores range from 17 to 85, with lower values indicating more disability.

c QoLI-20: Lehman Quality of Life Interview 20. Possible scores range from 20 to 140, with higher values indicating greater quality of life.

d CIS: Community Integration Scale. Possible scores range from 4 to 20 for the Psychological component and 0-7 for the Physical component, with higher values indicating greater integration.

e GAIN-SS: Global Assessment of Individual Needs Short Screener. Possible scores range from 0 to 5 with higher values indicating greater problem severity.

f Housing stability is not included in this table as we did not examine it at each follow-up point.

Table 4. Post-Imputation Pooled coefficient estimates (95% Confidence Interval) for Predictors in Linear Mixed Models for Repeated Measures for continuous outcomes and Rate Ratios (95% Confidence Interval) for Generalized Estimating Equations Models for count outcomes for the sample of women in the At Home/Chez Soi study (N=653).

	Mental illness symptom severity (CSI)			Community functioning (MCAS)			Condition-specific QoL (QoLI-20)			Psychological community integration (CIS)		
	Est	95% CI	p-val	Est	95% CI	p-val	Est	95% CI	p-val	Est	95% CI	p-val
Age (years)	-0.01	-0.06-0.04	0.680	-0.02	-0.060-.02	0.310	-0.06	-0.17- 0.04	0.250	-0.01	-0.03- 0.00	0.140
Less than high school	0.21	-0.98-1.40	0.731	**-1.32**	**-2.27 to -0.37**	**0.006**	0.87	-1.56- 3.30	0.480	0.15	-0.26- 0.55	0.476
3 or more years of homelessness	0.91	-0.27-2.09	0.132	**-1.75**	**-2.72 to -0.78**	**<.001**	-0.75	-3.14- 1.64	0540	-0.07	-0.48- 0.33	0.728
Supports ≥1 child under 18 years	0.11	-1.89-2.10	0.917	0.62	-0.96- 2.20	0.442	0.47	-3.52- 4.47	0.820	0.48	-0.20- 1.17	0.168
Moderate or high suicidality at baseline	**2.85**	**1.59- 4.11**	**<.001**	**-1.16**	**-2.10 to -0.22**	**0.015**	**-3.99**	**-6.49 to -1.49**	**<.001**	-0.30	-0.71- 0.11	0.150
5 or more ACE	**1.50**	**0.25- 2.76**	**0.019**	0.21	-0.75- 1.17	0.666	-0.56	-3.14- 2.02	0.670	0.31	-0.12- 0.73	0.157

	Physical community integration in past month (CIS) (N=653)			Severity of substance problems in past month (GAIN-SS) (N=653)			Number of emergency department visits in past 6 months (N=653)			Stable housing (N=653)		
		95% CI	p-val	RR	95% CI	p-val	RR	95% CI	p-val	beta* or Odds ratio **	95% CI	p-val
Age (years)	1.0	1.0- 1.0	0.834	**0.99**	**0.98-1.00**	**0.003**	1.00	0.99- 1.01	0.920	0.16<br>1.01	-0.04-0.36<br>1.00-1,02	0.122<br>0.1021
Less than high school	**0.85**	**0.77- 0.93**	**<.001**	**1.27**	**1.06-1.52**	**0.009**	1.11	0.83-1.49	0.474	-2.78<br>0.87	-7.43-1.88<br>0.69-1.09	0.242<br>0.222
3 or more years of homelessness	0.98	0.93- 1.11	0.720	**1.21**	**1.03- 1.43**	**0.021**	0.99	0.76-1.29	0.951	**-6.14**<br>**0.74**	**-10.9--1.32**<br>**0.58-0.94**	**0.013**<br>**0.013**
Supports ≥1 child under 18 years	1.10	0.96-1.27	0.176	1.05	0.80- 1.39	0.715	0.92	0.67-1.26	0.598	5.57<br>1.31	-2.17-13.3<br>0.89-1.92	0.158<br>0.171
Moderate or high suicidality at baseline	1.02	0.93-1.12	0.634	1.06	0.90- 1.27	0.465	**1.44**	**1.10-1.87**	**<0.001**	-1.82<br>0.91	-6.49-2.80<br>0.73-1.15	0.440<br>0.432

5 or more ACE	1.04	0.95-1.15	0.356	1.16	0.96- 1.39	0.114	1.05	0.77-1.42	0.772	2.93<br>1.16	2.05-7.92<br>0.90-1.48	0.249<br>0.245

Each model includes treatment group, categorical time, interaction of treatment x time, city, need level, baseline outcome values and all baseline predictors. For percentage or proportion of days stably housed over 24 months, time and interaction treatment by time are not included in linear* and fractional logistic models**

Supporting Information

Figure S1 – Flow of participant enrolment, assignment to treatment arm, and inclusion in analysis

Supplemental Table S1– Missing Rates (%) for Outcomes by Group, Time Period and Overall

Supplemental Table S2- SAS Syntax for Regression Models

Figure Click here to access/download;Figure;Figure 1.docx

Figure 1 – Health and social outcomes among Women who are Homeless and Mentally Ill, unadjusted means and 95% confidence intervals over 24 months of follow-up by study time point and treatment group

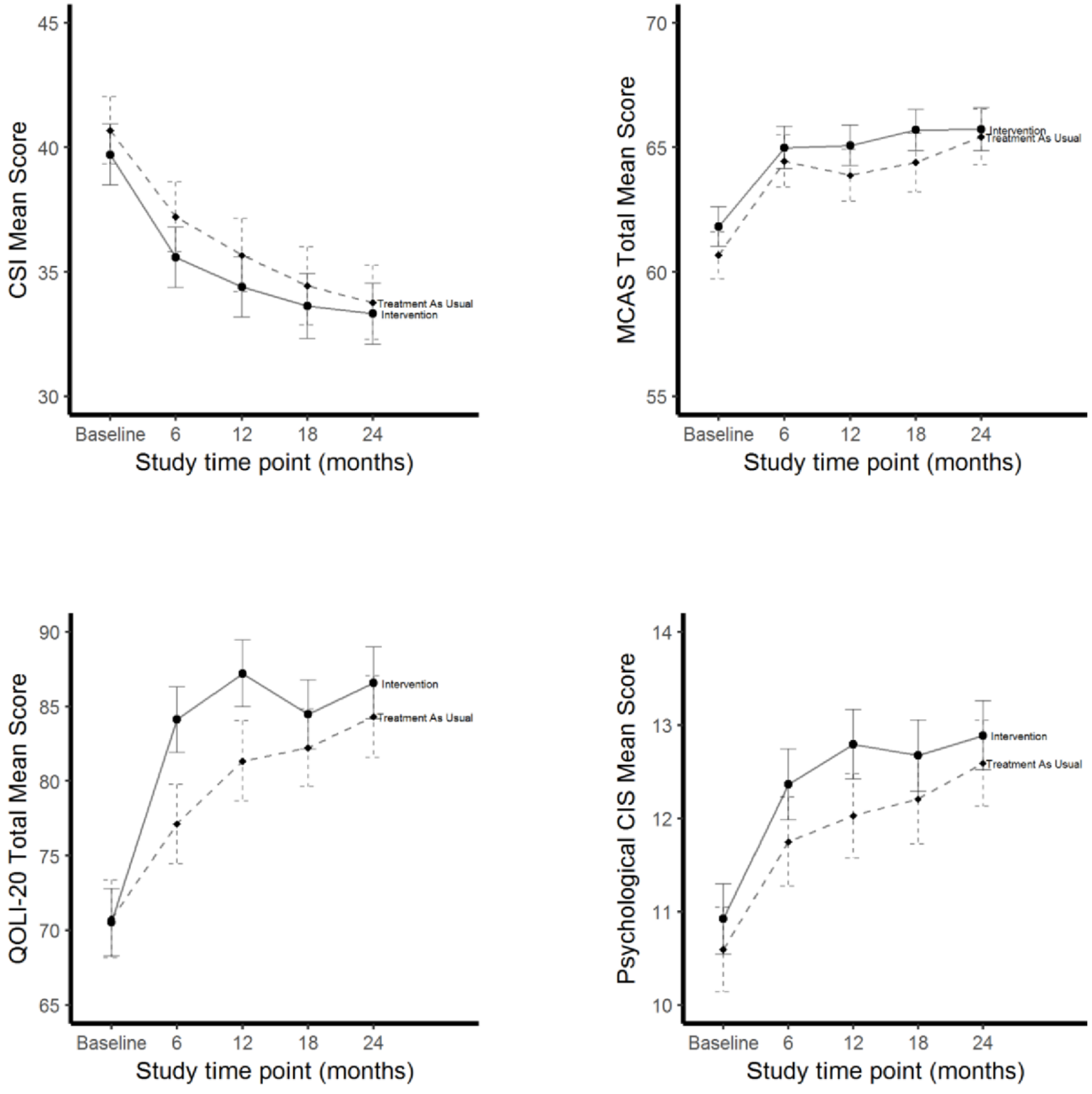

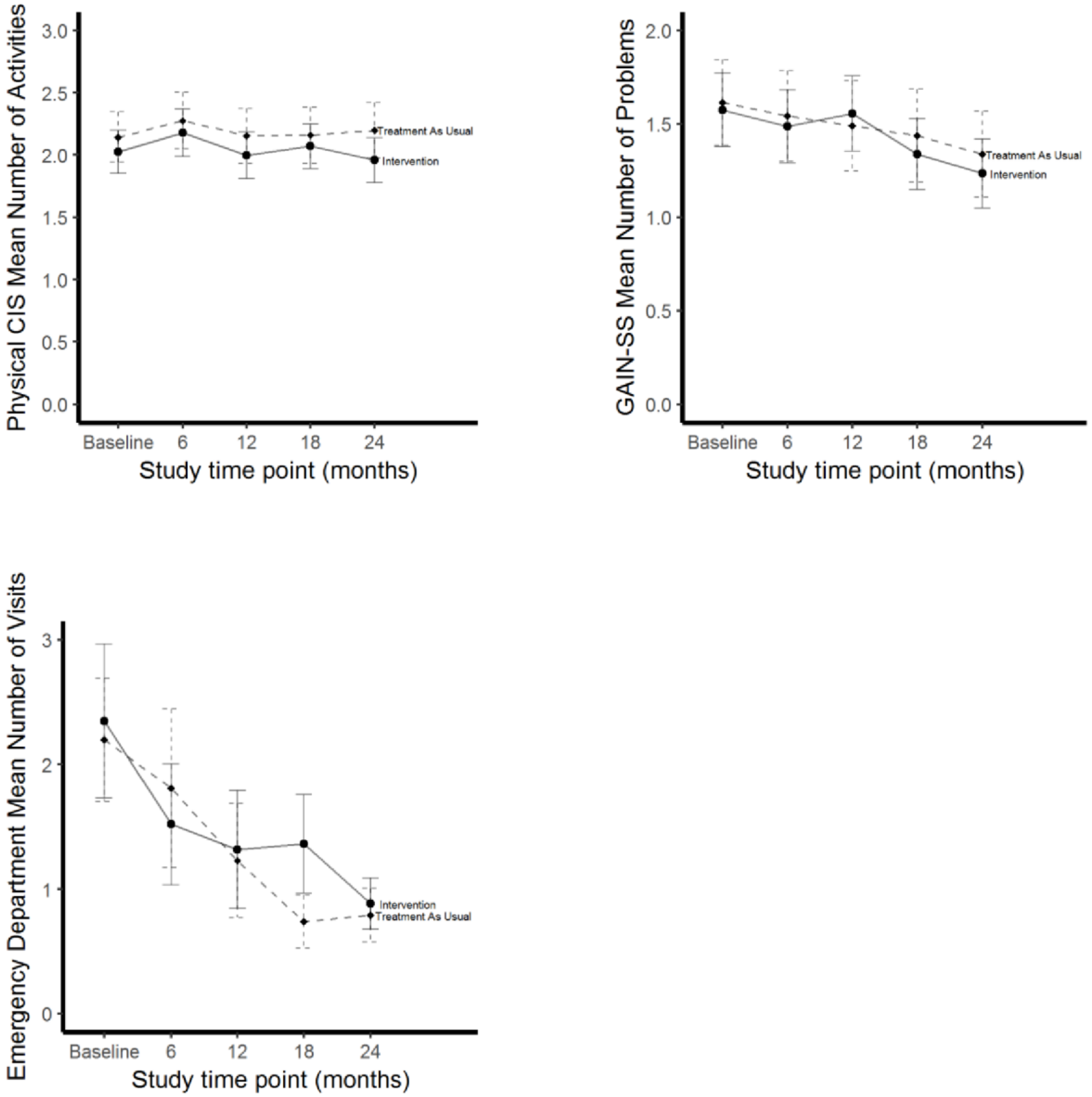

Note. N = 653. CSI = Colorado Symptom Index; MCAS = Multnomah Community Ability Scale; Lehman Quality of Life Interview 20 = QoLI-20; CIS = Community Integration Scale; GAIN-SS = Global Assessment of Individual Needs Short Screener.

Click here to access/download
Supporting Information
Supporting Information.docx

Women experiencing homelessness and mental illness in a Housing First multi-site trial: Looking beyond housing to social outcomes and well-being.

O'Campo P., Nisenbaum R., Crocker A. G., Nicholls T., Eiboff F., Adair C.E.

O'Campo, P., Centre for Urban Health Solutions, St Michael's Hospital. Dalla Lana School of Public Health, University of Toronto

Nisenbaum, R., Centre for Urban Health Solutions, Applied Health Research Centre, Li Ka Shing Knowledge Institute, St Michael's Hospital. Dalla Lana School of Public Health, University of Toronto

Crocker A. G., Director, Research & Academics, Institut national de psychiatrie légale Philippe-Pinel & Full professor, Department of Psychiatry & Addictions, and School of Criminology, Université de Montréal

Nicholls, T. L., Department of Psychiatry, University of British Columbia and BC Mental Health and Substance Use Services

Eiboff, F., Interdisciplinary Studies, School of Population and Public Health, University of British Columbia

Adair, C.E., Department of Psychiatry and Department of Community Health Sciences, Cumming School of Medicine, University of Calgary

Abstract

Objective: There is scant research on the effectiveness of permanent supportive housing for homeless women with mental illness. This study examines the effectiveness of Housing First with an unprecedentedly large sample of homeless women from five Canadian cities, and explore baseline risk factors that predict social, health and well-being outcomes over a 24 month-period.

Methods: The At Home/Chez Soi multi-site randomized controlled Housing First trial recruited over 600 women between October 2009 and July 2011. This is a post-hoc subgroup exploratory analysis of self-identified women with at least one follow-up interview who were randomized to Housing First (HF) (n=374) or treatment-as-usual (TAU) (n=279) and had at least one follow-up interview. Linear mixed models and generalized estimating equations were used after multiple imputation was applied to address missing data.

Results: At the end of follow-up, the mean percentage of days spent stably housed was higher for women in the intervention 74.8% (95%CI=71.7%–77.8%) compared with women in the treatment-as-usual group, 37.9% (95%CI=34.4%–41.3%), $p<0.001$. With few exceptions, social and mental health outcomes were similar for both groups at 6-, 12-, 18- and 24-months post-enrollment. Suicidality was a consistent predictor of increased mental health symptoms (beta=2.85, 95% CI 1.59-4.11, p<0.001), decreased quality of life (beta=-3.99, 95% CI -6.49 to -1.49, p<0.001), decreased community functioning (beta= -1.16, 95% CI -2.10 to -0.22, p=0.015) and more emergency department visits (rate ratio = 1.44, 95% CI 1.10-1.87, p<0.001) over the study period. Lower education was a predictor of lower community functioning (beta=-1.32, 95% CI -2.27 to -0.37, p=0.006) and higher substance use problems (rate ratio=1.27, 95% CI 1.06-1.52, p=0.009) during the study.

Conclusions: Housing First interventions ensured that women experiencing homelessness are quickly and consistently stably housed. However, they did not differentially impact health and social measures compared to treatment as usual. Ensuring positive health and social outcomes may require greater supports at enrolment for subgroups such as those with low educational attainment, and additional attention to severity of baseline mental health challenges, such as suicidality.

Key Words: women, homelessness, Housing First, randomized trial, mental health, suicidality, quality of life

Trial Registration: International Standard Randomized Control Trial Number Register Identifier: ISRCTN42520374 http://www.controlled-trials.com/ISRCTN42520374.

Introduction

Homelessness in Canada is primarily experienced by men, yet data show that over one-third of the homeless people in this country are women and close to 90% of the single parents who are homeless are women[1-3]. Mental health and substance use disproportionately affect women experiencing homelessness. Between 20-50% experience major depressive disorder and major depressive episodes as demonstrated in systematic reviews [4], between 30-40% experience post-traumatic stress disorder [5, 6], as many as one in five report moderate-to-high levels of suicidality [6], and a large proportion experience exceptional vulnerability to an array of health and social risks [6-11], including premature mortality [12]. A recent meta-analysis of observational studies in high-income countries reports that three-quarter of homeless individuals have a mental illness diagnosis with no differences by gender[13].

Women who are homeless have different health and social vulnerabilities compared to homeless men. They have higher rates of distress including suicidal ideation [14], greater experiences of trauma prior to and during episodes of homelessness including more adverse childhood events (ACEs) such as physical and sexual abuse and trauma-related disorders [6, 15-22], few options for effective woman-centred services and treatments for these traumatic events and other ailments, all of which predispose them to homelessness [14, 23-27]. They are also substantially more likely than homeless men to engage in survival sex work and are thus more vulnerable to exploitation, violence, infectious disease, gynecological problems and unwanted pregnancies [6, 28]. Homeless women with children express considerable traumatic distress because of frequent child apprehensions [29]. Because women who are homeless often seek shelter in informal settings such as couch surfing or sleeping rough, they are less visible and

have less access to services [14, 30, 31] and have received less attention in research and policy and practice.

Program planners and policy makers operate in a context of too little gender-specific data on programs and interventions and weak studies addressing the experiences of women [3, 4],[32]. As such, there have been calls for more research that moves away from gender-neutral approaches (i.e., examining men's and women's experiences with the same lens, as if there are no gender differences) [14, 32-34] towards more gender-sensitive frameworks, including women-only analyses that capture the variability around the unique challenges women who are homeless face and accelerating evidence-informed practice with this population[3, 30, 35, 36]. For interventions, there is a need for studies with large enough samples to examine whether and how programs work for women[4, 37-42].

Currently, with some exceptions [43], what we do know about housing interventions for women experiencing homelessness comes from small subsamples in longitudinal observational studies, [35, 44] randomized trial pilot, [45] or qualitative evaluations [33, 46]. A large multi-site supportive housing demonstration project for chronically homeless adults in 11 US communities, with 173 women as part of the sample, reported substantial improvements in housing stability but no gender differences in this and other service utilization or clinical outcomes over two years of follow-up [47]. In an observational study of an integrated service system initiative in 18 sites across the US, women (N=2727) demonstrated greater improvements in family relationships and social support compared to men (N=4502) over 18-months of follow-up[43].

Housing First, a supportive housing approach with demonstrated success in transitioning individuals experiencing chronic homelessness and mental illness into permanent housing, has negligible evidence on outcomes for women[48-51]. The Housing First model is based on harm

reduction principles of choice and self-determination, and provides immediate access to scattered-site housing through the provision of rent supplements and flexible recovery-oriented supports[50, 52]. Participation in Housing First is not contingent on participation in services or requirements of ‘housing readiness’ (e.g., sobriety or medication compliance) [50].

The At Home/Chez Soi randomized trial conducted in five cities across Canada provides an unprecedented opportunity to examine the impact of a housing first initiative among women for several reasons. First, it is the largest randomized trial of a Housing First initiative and includes an unprecedented sample of several hundred women. This large sample size facilitates a woman only analysis to focus on variability within this subsample and factors that are relevant for women living with homelessness such as sources of trauma or parental roles. A woman only analysis, too rarely seen in studies of homelessness, ensures that the focus remains on women within the sample as gender analyses largely focus on comparing experiences with those of men. With 24 months of follow-up and novel risk variables not previously examined in a Housing First analysis we can provide unique point-in-time and longitudinal evidence not previously considered because of prior sample size and variable availability limitations.

The present study employs the largest longitudinal sample of women experiencing homelessness and mental illness in a Housing First randomized controlled trial (RCT) and extends the follow up duration typically seen to 24 months. Our objectives were: (i) To compare change at the end of the study in health and social outcomes between women who received and did not receive the Housing First intervention; ii) To examine whether the Housing First intervention leads to better housing stability over 24 months, and health and social and outcomes between 6- to 24-months post enrolment; and, (iii) To explore baseline demographic, social, health measures, and ACEs as predictors of key study outcomes during the study period.

Methods

Methods and measures for this analysis have been extensively described elsewhere [49, 53-55]. Briefly, the Canadian At Home/Chez Soi five city RCT randomized Housing First among adults experiencing homelessness and serious mental illness [54]. Participants randomized to the Housing First (HF) intervention received scattered-site housing and either moderate or high needs-based services to support their recovery and integration into the community (i.e., Intensive Case Management or Assertive Community Treatment). Participants randomized to treatment-as-usual (TAU) had access to housing and services through other community programs. Each site had additional focus on a unique population (e.g., ethno-racial populations in Toronto and Indigenous populations in Winnipeg) and had specialized services to address their needs. Fidelity assessments ensured strong adherence to program theory of change[56]. Information about the study, such as the randomization process and a description of the intervention, was provided to all study participants prior to enrolment. An informed consent process was created to ensure that participants had the time and information to understand the complexities of the trial and follow-up activities. All participants provided written informed consent prior to enrolment. All enrolment and consent documents were harmonized across sites, however, each site had separate approval from relevant university/site Research Ethics Boards.

Over 24 months, participants were interviewed every 3 months to assess housing stability and every 6 months to evaluate health and social outcomes. Previous results from 2,148 participants (including 683 women) demonstrated that those in the HF arm showed positive improvements in housing stability, quality of life, and community functioning compared to participants in usual care [47, 49, 53]. This paper reports on an exploratory analysis of 653

participants who self-identified as women and had at least one follow-up after their baseline assessment (96%), including 588 followed to 24 months. Across all sites, 374 participants were from the HF group and 279 from the TAU group (Supplemental Figure S1).

Outcomes and other study variables

We describe in this section the outcome variables and any other scales used in this analysis. Stable housing was defined as "living in one's own room, apartment, or house, or with family, with an expected duration of residence of 6 months or more or tenancy rights" while enrolled in the study, taking into account participants' move in/out dates for each type of residence (e.g., street place, unstable residence, stable residence, institution)[57]. The *percentage of days stably housed during the 24-month period* was calculated as the total number of days stably housed divided by the total number of days for which any type of residence data were provided by the participant over 24 months and varied between 0% and 100% [49, 54]. Unlike the other outcomes, housing stability was measured at the end of the follow-up period for several reasons. First, the treatment group establishes stable housing within the first 1-3 months of enrolment and housing status rarely changes after that point in time. A small proportion of the treatment group was not consistently stably housed throughout the duration of the program; thus, this single cumulative measure captures housing stability better than if we had measured it at each follow-up time point. In sum, this cumulative housing stability is the best measure for the outcome of housing as it does not demonstrate incremental changes over the 24-month follow-up period.

The other outcomes were assessed via in-person interviews at baseline, 6-, 12-, 18, and 24 months post-enrollment.

The Lehman Quality of Life Interview 20 (QoLI-20) measured participants' condition-specific quality of life. Twenty items scored on a 7-point Likert scale define a total quality of life and domains of family, finance, leisure, living, safety, social, and overall quality of life. In this work, we used the total score calculated as the sum of all 20 items, which ranges from 20 to 140, with higher scores indicating better quality of life [58].

Community functioning was measured using the Multnomah Community Ability Scale (MCAS), a 17-item scale capturing total community functioning and degree of clinician/observer-rated functional ability in domains of health, adaptation, social skills, and behavior. In this work, we used the total score calculated as the sum of all 17 times, ranging from 17-85, with higher scores indicating less impairment [49].

The Colorado Symptom Index (CSI) assessed how often specific psychiatric symptoms were experienced, from 'at least every day' to 'not at all'. Fourteen self-report items were summed (range 14 to 40), with lower scores reflecting fewer symptoms [59, 60].

Emergency department (ED) visits were measured using self-reported counts in the past 6 months. The number of past-month substance-related problems were measured via the 5-item Global Assessment of Individual Needs Short Screener (GAIN-SS); higher counts correspond to greater problem severity [54].

The Community Integration Scale (CIS) measured self-reported psychological integration with the immediate community on a scale from 4-20; physical integration used counts of previous month social activities (e.g., attending a concert, meeting people for coffee) on a scale of 0-7. Higher scores on both reflect greater integration [61].

Baseline and predictor variables included data collected on traumatic events that occurred before the age of 18 years using the Adverse Childhood Experiences module assessing ten early

childhood adversities (yes/no items): emotional, physical or sexual abuse; emotional or physical neglect; household substance use, household mental illness and household criminal justice involvement; mother treated violently, and parental separation or divorce [62]. Scores ranged from 0 to 10 with higher scores indicating greater childhood adversity. We created two categories representing high levels of reported ACEs, ≥5 adversities, and four or fewer including none [63, 64]. Mental health diagnoses and levels of suicidality were assessed via the MINI Neuropsychiatric Interview 6.0 administered as part of the baseline enrolment interview[65]. No, low, moderate and high levels of suicidality were dichotomized into 'no/low' and 'moderate/high' suicidality. Information about all other variables (e.g., demographics, financial support of children, sources of income) was obtained via self-report during the in-person baseline interviews.

Statistical Analyses

Descriptive statistics for a wide range of descriptors were selected to characterize the sample by treatment group. Longitudinal outcomes were analyzed using linear mixed analysis of repeated measures models for continuous outcomes and generalized estimating equations for count outcomes (Poisson distribution for CIS-Physical Integration and negative binomial distribution for GAIN-SS and ED Visits), assuming an unstructured correlation matrix for the repeated measures. Percentage of days stably housed over the 24-months follow-up (range 0-100%) was modeled as a continuous outcome in a linear model. We also performed a sensitivity analysis by considering the proportion (0-1) of days stably housed and fitting a fractional logistic model. The fractional logistic model, which uses a quasi-likelihood function for estimation, was introduced by Papke and Woolridge (1996)[66]* and is appropriate for proportions when the values of 0 and 1 are admissible and observable. The fractional logistic model predicts the mean

of the proportion conditional on covariates. To ensure that the predicted mean is also between 0 and 1, the logit model for the mean is considered. The parameters estimated from this model can be exponentiated to yield odds ratios and 95% confidence intervals for the association between stable housing during the 24 months follow-up and covariates.

For the first objective, for each group, the adjusted change from baseline to 24-months follow-up was estimated by mean differences and 95% confidence intervals (continuous outcomes), and rate ratios and 95% confidence intervals (count outcomes). We -fit models that included treatment group (fixed effect, HF vs TAU), categorical time (24-months vs baseline), interaction of treatment x time, and study city and need level at baseline (high vs. moderate) as covariates. We modeled the total percentage of days stably housed over 24-months as a continuous outcome and fitted a linear regression model including treatment group, and city, need level and percentage of days stably housed in the three months prior to baseline as covariates

For the second objective, in a more detailed examination of possible differential effect over time, we tested for differences between HF and TAU at each follow-up time point from 6- to 24-months, adjusting for baseline outcomes. Except for percentage (or proportion) of days stably housed over 24 months, Models models included treatment group, categorical time (6-, 12-, 18-, 24-months), interaction of treatment x time, and city, need level and baseline outcome values as covariates. At each time point, we compared HF vs TAU by estimating mean differences and 95% confidence intervals or rate ratios and 95% confidence intervals. For percentage (or proportion) of days stably housed over 24-months we included treatment group, city, need level and percentage of days stably housed in the three months prior to baseline as covariates.

~~We modeled the total percentage of days stably housed over 24-months as a continuous outcome and fitted a linear regression model including treatment group, and city, need level and percentage of days stably housed in the three months prior to baseline as covariates~~

For the third objective, the differences between HF and TAU were no longer the focus. Rather we took advantage of the large sample of women in a study on homelessness to identify predictors of our outcomes. To accomplish this we expanded the models from the second objective and added the following baseline predictors: age at enrolment (continuous), education (1=less than high school, 0=high school or higher), duration of homelessness (1=≥3 ,0= <3 years), whether minor children were being supported by the participant (1=yes, 0=no), level of suicidality (1=moderate or high, 0=mild or none), and high level of ACEs at baseline (1=≥5 , 0=<5 ACEs). These predictors were chosen because they represent a knowledge gap or have yielded inconsistent findings on longitudinal studies of women experiencing homelessness. We were unable to include ethnicity or Indigenous identity due to multicollinearity with study city.

Multiple imputation with chained equations (MICE) was used to handle missing data from loss to follow-up, withdrawal, skipped interviews, nonresponse on specific items, and systematically measured lack of interviewer confidence in participant responses. We imputed all outcomes simultaneously and MICE imputation models included all variables needed to address three objectives[67] . Although outcome missing rates varied between 0.53% to 31.9% (Supplemental Table S1), approximately 70% of the participants had at least one missing value in at least one outcome in at least one point in time. Therefore, we chose 70 imputations following guidelines given by White et al. (2010) that the percentage of cases with incomplete data should be similar to the number of imputations generated [67]. Comparisons between

observed and imputed data via diagnostic plots were used to assess the quality and plausibility of the imputations[68]

Data were imputed using Stata software (mi impute chained, Stata Statistical Software: Release 13. College Station, TX: StataCorp LP) and analyzed using SAS (PROC MIXED, GENMOD , GLIMMIX, PROC MIANALYZE, SAS 9.4, SAS Institute Inc, Cary, NC). SAS Syntax is provided in Appendix X. Figures were created using the ggplot2 package in R software version 3.4.0 (R Core Team (2017) [69]. All statistical tests were two-tailed and significance was defined as $p<.05$. No adjustments for multiple testing were applied [70].

Results

We took advantage of the rich data collected in this study and present a broad array of participant demographic, social, and health characteristics at enrolment (Table 1). Fifteen percent of the women were born outside of Canada (median time in Canada: TAU=25yrs; HF = 21yrs). Three percent of women were employed at baseline and 90% received social assistance of some type as a source of income. Around 40% (*n*=274) reported having minor children, and of these, 22% (*n*=60) were providing full or partial financial support to the child(ren). The most prevalent mental illnesses were depressive episodes, PTSD, and alcohol or substance dependence. Approximately 40% had moderate-to-high suicidality, around 40% had post-traumatic stress disorder, about 60% reported ≥5 ACEs, and the majority had three or more co-morbid medical conditions.

Table 2 displays results from models examining changes from baseline to 24 months for those in HF and TAU arms for each of our social and well-being outcomes, and comparing groups with respect to housing stability over the 24-month period. Over the 24-month follow-up

period, substantial improvements were observed for all the outcomes for both HF and TAU participants. The mean percentage of days spent stably housed during follow-up for women receiving HF was 74.8% (95%CI=71.7%–77.8%) compared with 37.9% (95%CI=34.4%–41.3%) TAU, a difference of 36.9% (95%CI=32.4%–41.4%, *p*<0.001). . Of note, mean total QoLI-20 scores improved 16.0 points (95%CI=13.6–18.4) and 13.4 points (95%CI=10.6–16.2) in the HF and TAU groups, respectively, and number of ED visits decreased by approximately 60% in both groups.

These improvements over the 24-month period are more clearly illustrated in Figure 1 for the HF intervention and TAU groups for all social and health unadjusted outcomes at *each observation point* from baseline to 24-months. For all outcomes, except CIS-Physical where scores remained flat throughout the follow-up, both groups experienced similar significant change from baseline to 24-months (Figure 1).

Figure 1 – Health and social outcomes among Women who are Homeless and Mentally Ill, unadjusted means and 95% confidence intervals over 24 months of follow-up by study time point and treatment group

The mean percentage of days spent stably housed during follow-up for women receiving HF was 74.8% (95%CI=71.7%–77.8%) compared with 37.9% (95%CI=34.4%–41.3%) TAU, a difference of 36.9% (95%CI=32.4%–41.4%, *p*<0.001). In addition, the odds of stable housing during follow-up was over 5 times times higher among those in the HF group than in the TAU group (OR=5.09, 95% CI= 4.08-6.35, p<0.001). Table 3 displays changes over time in greater detail through results of the models to assessing whether social and health outcomes were better

in HF vs. TAU groups *at each follow-up point*. For condition-specific quality of life, significant improvements of 7.1 and 6.1 points were observed in the HF group at the 6- and 12-month follow-ups, respectively. The HF group also showed significant improvements in psychological community integration by 0.7 points at the 6-month follow-up. However, in the last year of the trial, the ED visits fell faster for the TAU group (0.6 visits) compared to the HF group (1.1 visits) at 18 months, with a rate ratio of 1.81 (95%CI=1.26–2.60).

Table 4 no longer focuses on differences between HF and TAU as results presented here identify predictors of our outcomes. Moderate to high suicidality had the most consistent association to poorer health and service utilization outcomes at 24 months follow-up. Compared to those with no or low suicidality at baseline, moderate-to-high suicidality at baseline was associated with nearly three points on the CSI indicating greater mental health symptom severity (beta=2.85, 95%CI=1.59–4.11, $p<0.001$), QoLI-20 mean scores were almost four points lower indicating reduced quality of life (beta=-3.99, 95%CI=-6.49 to -1.49, $p=0.002$), and almost 1.5 more ED visits (rate ratio=1.44, 95%CI=1.10–1.87, $p=0.007$). Low education was also associated with several outcomes. Compared to those with higher education, women with less than a high school level of education at baseline predicted lower community functioning (MCAS) scores (beta=-1.32, 95%CI=-2.27 to -0.37, $p=0.006$), 15% fewer social activities (CIS) in the previous month (rate ratio=0.85, 95%CI=0.77–0.93, $p<0.001$), and more severe substance-related problems at 24 months follow-up (rate ratio=1.27, 95%CI=1.06–1.52, $p=0.009$). Having been homeless for ≥3 years at baseline was associated with lower percentage days stably housed at 24-months (beta=-6.1%, 95%CI=-11.0% to -1.3%, $p=0.013$; OR=0.74, 95% CI 0.58-0.94, p=0.0132). An ACE score of 5 or more was associated with a 1.5 point increase in mental illness symptom severity (95%CI=0.25–2.76, $p=0.019$). Finally, we explored and found no evidence

that baseline levels of ACEs and suicidality, separately, were effect modifiers for any of the above analyses.

Discussion

This is the first analysis of women's outcomes in an RCT of Housing First. Prior studies and trials on housing interventions for people who experience homelessness often included very small numbers of women or few variables reflecting women's unique needs and experiences that have precluded documentation of the heterogeneity of experiences and in-depth understanding of this population.

In this Canadian multi-site study, the largest longitudinal sample of women experiencing homelessness to date, the intervention group experienced housing stability for 75% of the 24-month follow-up, as compared to 38% for women in the TAU group, a finding similar to previously published reports of At Home/Chez Soi participants, the majority of whom were men [49, 53]. Even if the large proportion of time spent stably housed for women who received Housing First compared to the TAU group did not translate into greater improvements in health and social outcomes by the end of the study, there were some early differences favoring the treatment group in the first year for quality of life and psychological community integration. Immediate placement in housing and early housing stability for HF compared to TAU [71]participants likely contributed to faster improvements in these outcomes.

While ED visits declined in a similar fashion for HF and TAU over the 24-month follow-up, an unanticipated finding was a significant reduction in ED visits of 0.5 visits at 18 months for the TAU group over the HF group. For this rare count outcome, the dip in ED visits for TAU at 18 months could be a chance improvement as both groups had similar levels of ED visits by 24

months. Analyses of the full At Home/Chez Soi trial data did not show differences for this outcome[49]. One possibility is that uncertainty around continued housing as the trial was ending may have created high levels of stress in the Housing First group, but if this were the case, we would have likely seen similar changes in other study outcomes or among men in the analyses of the full sample.

Since improvements in outcomes over the 24-month period were similar for both the HF and TAU groups, we moved beyond the intention-to-treat analysis and examined baseline predictors for our outcomes across the full sample. We focused on baseline suicidality, ACEs, and parental responsibilities as predictors- variables rarely examined in past studies of supported housing or Housing First [35, 43-45, 72, 73]. As might be expected, women with ≥5 ACEs or moderate-to-high levels of suicidality at baseline showed poorer improvement by 24 months compared to those with low ACE or suicidality scores. Moderate-to-high suicidality was a strong predictor of poorer outcomes at 24 months, including mental health, quality of life, community integration, and ED visits. Past cross-sectional studies of combined samples of homelessness men and women have demonstrated similar links between suicidality, childhood and adult trauma, substance abuse, and mental health [20, 21, 74]. However, while cross-sectional associations between suicidality and substance among women who are homeless have been reported in the literature[19, 75] our study is the first to examine these associations longitudinally.

Contrary to what we might have anticipated on the importance of ACEs in predicting multiple study outcomes given past research[76], we found that high levels of ACEs only predicted significantly worse scores on mental illness severity (CSI). This is consistent with a recent study of the full sample of At Home/Chez Soi where ACEs total scores predicted several

mental health conditions at 24-months[51]. Our choice of a cutoff representing high numbers of ACEs, while informed by existing literature, may have limited our ability to detect an association. Alternatively, ACEs may be limited in capturing relevant adverse conditions. A recent study has suggested that an expanded ACEs tool might be used to capture important domains currently missing such as severe poverty or family loss and separation[77], which might be particularly relevant in capturing sources of trauma and adversity for women experiencing homelessness. Our findings also suggested that low educational attainment at baseline was a significant predictor of worse mental health, community integration, and substance use outcomes independently of the other variables in the model. Low educational attainment may be a proxy for other factors such as low health literacy which might impact management of health conditions and requires further investigation [78]. Providing economic support for dependent children was not related to any of our study outcomes.

Although the At Home/Chez Soi support services were important for intervention participants to remain stably housed and make gains in health and social standing, these supports and services were not designed in this Housing First program to be tailored to women's unique needs. Psychological housing stability which includes considerations of safety, security and feelings of home, is particularly important for women while the At Home/Chez Soi focused predominantly on material housing stability[79]. Women who are unstably housed or homeless need services that address trauma and promote informal peer support networks [33, 80]. Although staff were trained to be responsive to individual needs, the At Home/Chez Soi intervention was not specifically designed to provide the latter two resources. In addition, more than 40% of our participants were homeless for more than three years at enrolment. Two years of stable housing with mental health supports may not have been sufficient to resolve chronic

distress from being homeless with co-morbid health, trauma and substance use problems, particularly given the high rates of reported PTSD and ACEs[14].

The lack of greater improvements in the HF arm in this analysis might have been due to the intensive follow-up schedule that could have led to a Hawthorne effect among the TAU group, and higher loss to follow-up in the TAU group might have contributed to smaller differences between groups at 24 months. Despite our sample of homeless women being relatively large, it may not have been sufficient to detect small effects. The At Home/Chez Soi study did not capture gender-sensitive outcomes specific to women experiencing homelessness and therefore we may have missed important changes, such as those associated with positive social support, increased safety from crime and victimization, or strengthening of relationships with children, family or friends[3, 81].

Our study adds to the growing yet sparse literature on the characteristics and needs of women experiencing homelessness and the effectiveness of interventions to eliminate homelessness. Given that women make up more than one third of the homeless population, and their needs differ from that of men[3, 14, 27, 32], it is important that their experiences with homelessness and recovery receive ample focus to better inform the design and implementation of interventions that meet their unique needs. Moreover, the homelessness epidemic has only worsened in the context of the COVID pandemic so the issues remain relevant and these new analyses can provide insights[82].

References

1. Gaetz S, Dej E, Richter T, Redman M. The State of Homelessness in Canada 2016. Toronto: 2016.
2. Employment and Social Development Canada. Everyone Counts 2018: Highlights - Preliminary results from the second nationally coordinated Point-in-Time count of homelessness in Canadian communities. 2019.
3. Schwan K, Versteegh A, Perri M, Caplan R, Baig K, Dej E, et al. The State of Women's Housing Need & Homelessness in Canada: A Literature Review. Hache, A., Nelson, A., Kratochvil, E., & Malenfant, J. (Eds). Toronto, ON: Canadian Observatory on Homelessness Press. 2020.
4. Duke A, Searby A. Mental Ill Health in Homeless Women: A Review. Issues Ment Health Nurs. 2019;40(7):605-12. doi: 10.1080/01612840.2019.1565875. PubMed PMID: WOS:000474047300001.
5. Ferguson KM, Bender K, Thompson SJ. Gender, coping strategies, homelessness stressors, and income generation among homeless young adults in three cities. Social science & medicine (1982). 2015;135:47-55. Epub 2015/05/06. doi: 10.1016/j.socscimed.2015.04.028. PubMed PMID: 25942470.
6. Strehlau V, Torchalla I, Kathy L, Schuetz C, Krausz M. Mental health, concurrent disorders, and health care utilization in homeless women. Journal of psychiatric practice. 2012;18(5):349-60. Epub 2012/09/22. doi: 10.1097/01.pra.0000419819.60505.dc. PubMed PMID: 22995962.
7. Bassuk EL, Buckner JC, Perloff JN, Bassuk SS. Prevalence of mental health and substance use disorders among homeless and low-income housed mothers. The American journal of psychiatry. 1998;155(11):1561-4. Epub 1998/11/13. doi: 10.1176/ajp.155.11.1561. PubMed PMID: 9812118.
8. Shinn M, Knickman JR, Weitzman BC. Social relationships and vulnerability to becoming homeless among poor families. The American psychologist. 1991;46(11):1180-7. Epub 1991/11/01. PubMed PMID: 1772155.
9. Banyard VL, Graham-Bermann SA. Surviving poverty: stress and coping in the lives of housed and homeless mothers. The American journal of orthopsychiatry. 1998;68(3):479-89. Epub 1998/08/01. PubMed PMID: 9686299.
10. Nelson G, Patterson M, Kirst M, Macnaughton E, Isaak CA, Nolin D, et al. Life changes among homeless persons with mental illness: a longitudinal study of housing first and usual treatment. Psychiatric services (Washington, DC). 2015;66(6):592-7. Epub 2015/02/18. doi: 10.1176/appi.ps.201400201. PubMed PMID: 25686813.
11. Riley ED, Weiser SD, Sorensen JL, Dilworth S, Cohen J, Neilands TB. Housing Patterns and Correlates of Homelessness Differ by Gender among Individuals Using San Francisco Free Food Programs. Journal of Urban Health. 2007;84(3):415-22. doi: 10.1007/s11524-006-9153-3.
12. Cheung AM, Hwang SW. Risk of death among homeless women: a cohort study and review of the literature. CMAJ : Canadian Medical Association journal = journal de l'Association medicale canadienne. 2004;170(8):1243-7. Epub 2004/04/14. PubMed PMID: 15078846; PubMed Central PMCID: PMCPMC385354.
13. Gutwinski S, Schreiter S, Deutscher K, Fazel S. The prevalence of mental disorders among homeless people in high-income countries: An updated systematic review and meta-regression analysis. PLoS Med. 2021;18(8):e1003750. Epub 2021/08/24. doi: 10.1371/journal.pmed.1003750. PubMed PMID: 34424908; PubMed Central PMCID: PMCPMC8423293.
14. Milaney K, Williams N, Lockerbie SL, Dutton DJ, Hyshka E. Recognizing and responding to women experiencing homelessness with gendered and trauma-informed care. Bmc Public Health. 2020;20(1). doi: 10.1186/s12889-020-8353-1. PubMed PMID: WOS:000522413900001.
15. Brunette M, Drake RE. Gender differences in homeless persons with schizophrenia and substance abuse. Community mental health journal. 1998;34(6):627-42. Epub 1998/12/02. PubMed PMID: 9833202.

16. Rich AR, Clark C. Gender differences in response to homelessness services. Evaluation and Program Planning. 2005;28:69-81.
17. Roll CN, Toro PA, GL. O. Characteristics and experiences of homeless adults: A comparison of single men, single women, and women with children. Journal of Community Psychology. 1999;27:189-98.
18. Torchalla I, Strehlau V, Li K, Aube Linden I, Noel F, Krausz M. Posttraumatic stress disorder and substance use disorder comorbidity in homeless adults: Prevalence, correlates, and sex differences. Psychology of addictive behaviors : journal of the Society of Psychologists in Addictive Behaviors. 2014;28(2):443-52. Epub 2013/08/07. doi: 10.1037/a0033674. PubMed PMID: 23915373.
19. Benda BB. Gender differences in predictors of suicidal thoughts and attempts among homeless veterans that abuse substances. Suicide & life-threatening behavior. 2005;35(1):106-16. Epub 2005/04/22. doi: 10.1521/suli.35.1.106.59262. PubMed PMID: 15843327.
20. Desai RA, Liu-Mares W, Dausey DJ, Rosenheck RA. Suicidal ideation and suicide attempts in a sample of homeless people with mental illness. The Journal of nervous and mental disease. 2003;191(6):365-71. Epub 2003/06/27. doi: 10.1097/01.nmd.0000071584.88965.e1. PubMed PMID: 12826917.
21. Eynan R, Langley J, Tolomiczenko G, Rhodes AE, Links P, Wasylenki D, et al. The association between homelessness and suicidal ideation and behaviors: results of a cross-sectional survey. Suicide & life-threatening behavior. 2002;32(4):418-27. Epub 2002/12/28. PubMed PMID: 12501966.
22. Rodriguez-Moreno S, Vázquez JJ, Roca P, Panadero S. Differences in stressful life events between men and women experiencing homelessness. Journal of Community Psychology. 2021;49(2):375-89. doi: https://doi.org/10.1002/jcop.22465.
23. Herman DB, Susser ES, Struening EL, Link BL. Adverse childhood experiences: are they risk factors for adult homelessness? American journal of public health. 1997;87(2):249-55. Epub 1997/02/01. PubMed PMID: 9103105; PubMed Central PMCID: PMCPMC1380802.
24. Montgomery AE, Cutuli JJ, Evans-Chase M, Treglia D, Culhane DP. Relationship among adverse childhood experiences, history of active military service, and adult outcomes: homelessness, mental health, and physical health. American journal of public health. 2013;103 Suppl 2:S262-8. Epub 2013/10/24. doi: 10.2105/ajph.2013.301474. PubMed PMID: 24148064; PubMed Central PMCID: PMCPMC3969137.
25. Roos LE, Mota N, Afifi TO, Katz LY, Distasio J, Sareen J. Relationship between adverse childhood experiences and homelessness and the impact of axis I and II disorders. American journal of public health. 2013;103 Suppl 2:S275-81. Epub 2013/10/24. doi: 10.2105/ajph.2013.301323. PubMed PMID: 24148049; PubMed Central PMCID: PMCPMC3969113.
26. Patterson ML, Moniruzzaman A, Somers JM. Setting the stage for chronic health problems: cumulative childhood adversity among homeless adults with mental illness in Vancouver, British Columbia. BMC Public Health. 2014;14:350. Epub 2014/04/15. doi: 10.1186/1471-2458-14-350. PubMed PMID: 24726046; PubMed Central PMCID: PMCPMC3991866.
27. Schwan K, Vaccaro M, Reid L, Ali N, Baig K. The Pan-Canadian Women's Housing & Homelessness Survey. Toronto, ON: Canadian Observatory on Homelessness, 2021.
28. Milaney K, Williams N, Lockerbie SL, Dutton DJ, Hyshka E. Recognizing and responding to women experiencing homelessness with gendered and trauma-informed care. BMC Public Health. 2020;20(1):397. Epub 2020/03/29. doi: 10.1186/s12889-020-8353-1. PubMed PMID: 32216770; PubMed Central PMCID: PMCPMC7099814.
29. Torchalla I, Linden IA, Strehlau V, Neilson EK, Krausz M. "Like a lots happened with my whole childhood": violence, trauma, and addiction in pregnant and postpartum women from Vancouver's Downtown Eastside. Harm Reduction Journal. 2015;12(1):1. doi: 10.1186/1477-7517-12-1.

30. Shier ML, Jones ME, Graham JR. Sociocultural Factors to Consider When Addressing the Vulnerability of Social Service Users: Insights From Women Experiencing Homelessness. Affilia. 2011;26(4):367-81. doi: 10.1177/0886109911428262.
31. Winetrobe H, Wenzel S, Rhoades H, Henwood B, Rice E, Harris T. Differences in Health and Social Support between Homeless Men and Women Entering Permanent Supportive Housing. Women's Health Issues. 2017;27(3):286-93. doi: https://doi.org/10.1016/j.whi.2016.12.011.
32. Perri M, O'Campo P. A gap in knowledge surrounding urban housing interventions: a call for gender redistribution. Health Promot Int. 2021;36(4):908-12. Epub 2021/05/12. doi: 10.1093/heapro/daab062. PubMed PMID: 33975348.
33. Oudshoorn A, Forchuk C, Hall J, Smith-Carrier T, Van Berkum A. An evaluation of a Housing First program for chronically homeless women. Journal of Social Inclusion. 2018;9(2):34-50. doi: 10.36251/josi.136. PubMed PMID: WOS:000454531300004.
34. Perri M, Metheny N, Matheson FI, Potvin K, O'Campo P. Finding opportunity in the COVID-19 crisis: prioritizing gender in the design of social protection policies. Health Promot Int. 2022;37(1). Epub 2021/05/17. doi: 10.1093/heapro/daab045. PubMed PMID: 33993289; PubMed Central PMCID: PMCPMC8194510.
35. Cameron A, Abrahams H, Morgan K, Williamson E, Henry L. From pillar to post: homeless women's experiences of social care. Health Soc Care Community. 2016;24:345-32.
36. Fotheringham S, Walsh CA, Burrowes A. 'A place to rest': the role of transitional housing in ending homelessness for women in Calgary, Canada. Gender, Place & Culture. 2014;21(7):834-53. doi: 10.1080/0966369X.2013.810605.
37. Blehar MC. Public health context of women's mental health research. The Psychiatric clinics of North America. 2003;26(3):781-99. Epub 2003/10/18. doi: 10.1016/s0193-953x(03)00039-x. PubMed PMID: 14563109.
38. Institute of Medicine. Sex-Specific Reporting of Scientific Research: A Workshop Summary. Washington, DC: The National Academies Press. https://doi.org/10.17226/13307. 2012.
39. Jahn I, Bornhorst C, Gunther F, Brand T. Examples of sex/gender sensitivity in epidemiological research: results of an evaluation of original articles published in JECH 2006-2014. Health research policy and systems. 2017;15(1):11. Epub 2017/02/17. doi: 10.1186/s12961-017-0174-z. PubMed PMID: 28202078; PubMed Central PMCID: PMCPmc5312447.
40. Klap R, Humphreys K. Designing Studies for Sex and Gender Analyses: How Research Can Derive Clinically Useful Knowledge for Women's Health. Women's health issues : official publication of the Jacobs Institute of Women's Health. 2019;29 Suppl 1:S12-s4. Epub 2019/06/30. doi: 10.1016/j.whi.2019.05.002. PubMed PMID: 31253235.
41. Welch V, Doull M, Yoganathan M, Jull J, Boscoe M, Coen SE, et al. Reporting of sex and gender in randomized controlled trials in Canada: a cross-sectional methods study. Res Integr Peer Rev. 2017;2:15-. doi: 10.1186/s41073-017-0039-6. PubMed PMID: 29451565.
42. Tsai J, Byrne TH. National Utilization Patterns of Veterans Affairs Homelessness Programs in the Era of Housing First. Psychiatric Services. 2019;70(4):309-15. doi: 10.1176/appi.ps.201800393. PubMed PMID: WOS:000462848600008.
43. Cheng AL, Kelly PJ. Impact of an integrated service system on client outcomes by gender in a national sample of a mentally ill homeless population. Gender medicine. 2008;5(4):395-404. Epub 2008/12/26. doi: 10.1016/j.genm.2008.10.003. PubMed PMID: 19108812.
44. Nesse L, Gonzalez Marianne T, Aamodt G, Raanaas Ruth K. Recovery, quality of life and issues in supported housing among residents with co-occurring problems: a cross-sectional study. Advances in Dual Diagnosis. 2020;13(2):73-87. doi: 10.1108/ADD-10-2019-0014.
45. Tinland A, Zemmour K, Auquier P, Boucekine M, Girard V, Loubiere S, et al. Homeless women with schizophrenia reported lower adherence to their medication than men: results from the French

Housing First experience. Social Psychiatry and Psychiatric Epidemiology. 2017;52(9):1113-22. doi: 10.1007/s00127-017-1411-z. PubMed PMID: WOS:000408956600005.
46. Adame AL, Perry C, Pierce E. Community and Housing First: A qualitative analysis of USA residents' perspectives. Health Soc Care Community. 2020;28(4):1292-300. Epub 2020/03/05. doi: 10.1111/hsc.12962. PubMed PMID: 32128910.
47. Edens EL, Mares AS, Rosenheck RA. Chronically homeless women report high rates of substance use problems equivalent to chronically homeless men. Women's health issues : official publication of the Jacobs Institute of Women's Health. 2011;21(5):383-9. Epub 2011/06/28. doi: 10.1016/j.whi.2011.03.004. PubMed PMID: 21703865.
48. Aubry T, Tsemberis S, Adair CE, Veldhuizen S, Streiner D, Latimer E, et al. One-Year Outcomes of a Randomized Controlled Trial of Housing First With ACT in Five Canadian Cities. Psychiatric Services. 2015;66(5):463-9. doi: 10.1176/appi.ps.201400167.
49. Stergiopoulos V, Hwang SW, Gozdzik A, Nisenbaum R, Latimer E, Rabouin D, et al. Effect of scattered-site housing using rent supplements and intensive case management on housing stability among homeless adults with mental illness: a randomized trial. Jama. 2015;313(9):905-15. Epub 2015/03/04. doi: 10.1001/jama.2015.1163. PubMed PMID: 25734732.
50. Tsemberis S, Gulcur L, Nakae M. Housing First, consumer choice, and harm reduction for homeless individuals with a dual diagnosis. American journal of public health. 2004;94(4):651-6. Epub 2004/04/01. PubMed PMID: 15054020; PubMed Central PMCID: PMCPmc1448313.
51. Liu M, Mejia-Lancheros C, Lachaud J, Latimer E, Aubry T, Somers J, et al. Overall and Gender-specific Associations between Dimensions of Adverse Childhood Experiences and Mental Health Outcomes among Homeless Adults: Associations Générales et Sexospécifiques Entre les Dimensions des Expériences Défavorables de L'enfance et les Résultats de Santé Mentale Chez les Adultes Sans Abri. Can J Psychiatry. 2021;66(10):906-17. Epub 2021/01/28. doi: 10.1177/0706743721989158. PubMed PMID: 33502253; PubMed Central PMCID: PMCPMC8573704.
52. Paquette K, Pannella Winn LA. The Role of Recovery Housing: Prioritizing Choice in Homeless Services. Journal of dual diagnosis. 2016;12(2):153-62. Epub 2016/04/12. doi: 10.1080/15504263.2016.1175262. PubMed PMID: 27064834.
53. Aubry T, Goering P, Veldhuizen S, Adair CE, Bourque J, Distasio J, et al. A Multiple-City RCT of Housing First With Assertive Community Treatment for Homeless Canadians With Serious Mental Illness. Psychiatric services (Washington, DC). 2016;67(3):275-81. Epub 2015/12/02. doi: 10.1176/appi.ps.201400587. PubMed PMID: 26620289.
54. Goering PN, Streiner DL, Adair C, Aubry T, Barker J, Distasio J, et al. The At Home/Chez Soi trial protocol: a pragmatic, multi-site, randomised controlled trial of a Housing First intervention for homeless individuals with mental illness in five Canadian cities. BMJ open. 2011;1(2):e000323. Epub 2011/11/22. doi: 10.1136/bmjopen-2011-000323. PubMed PMID: 22102645; PubMed Central PMCID: PMCPmc3221290.
55. Aquin JP, Roos LE, Distasio J, Katz LY, Bourque J, Bolton JM, et al. Effect of Housing First on Suicidal Behaviour: A Randomised Controlled Trial of Homeless Adults with Mental Disorders. Can J Psychiatry. 2017;62(7):473-81. Epub 2017/07/07. doi: 10.1177/0706743717694836. PubMed PMID: 28683228; PubMed Central PMCID: PMCPMC5528985.
56. Nelson G, Macnaughton E, Caplan R, MacLeod T, Townley G., Piat M, et al. Follow-up Implementation and fidelity Evaluation of the mental Health Commission of Canada's At Home/Chez Soi Project: Cross-site Report. Mental Health Commission of Canada, 2013.
57. Tsemberis S., McHugo G., Williams V., Hanrahan P., Stefancic A. Measuring homelessness and residential stability: The residential time-line follow-back inventory. Journal of Community Psychology. 2007;35:29-42.

58. Uttaro T., Lehman A. Graded response modeling of the Quality of Life Interview. Evaluation and Program Planning. 1999;22:41-52.
59. Barker S, Barron N, McFarland BH, Bigelow DA, Carnahan T. A community ability scale for chronically mentally ill consumers: Part II. Applications. Community Ment Health Journal. 1994;30(5):459-72.
60. Dickerson FB, Origoni AE, Pater A, Friedman BK, Kordonski WM. An expanded version of the Multnomah Community Ability Scale: anchors and interview probes for the assessment of adults with serious mental illness. Community Ment Health Journal. 2003;39(2):131-7.
61. Conrad KJ, Yagelka JR, Matters MD, Rich A.R., Williams V., Buchanan M. Reliability and validity of a modified Colorado Symptom Index in a national homeless sample. Mental Health Services Research. 2001;3:141-53.
62. Felitti VJ, Anda RF, Nordenberg D, Williamson DF, Spitz AM, Edwards V, et al. Relationship of childhood abuse and household dysfunction to many of the leading causes of death in adults. The Adverse Childhood Experiences (ACE) Study. Am J Prev Med. 1998;14(4):245-58. Epub 1998/06/23. doi: 10.1016/s0749-3797(98)00017-8. PubMed PMID: 9635069.
63. Chapman DP, Whitfield CL, Felitti VJ, Dube SR, Edwards VJ, Anda RF. Adverse childhood experiences and the risk of depressive disorders in adulthood. J Affect Disord. 2004;82(2):217-25. Epub 2004/10/19. doi: 10.1016/j.jad.2003.12.013. PubMed PMID: 15488250.
64. Messina N, Grella C. Childhood trauma and women's health outcomes in a California prison population. American journal of public health. 2006;96(10):1842-8. Epub 2006/09/30. doi: 10.2105/ajph.2005.082016. PubMed PMID: 17008581; PubMed Central PMCID: PMCPMC1586137.
65. Lecrubier Y, Sheehan DV, Weiller E, Amorim P, Bonora I, Harnett Sheehan K, et al. The Mini International Neuropsychiatric Interview (MINI). A short diagnostic structured interview: reliability and validity according to the CIDI. European Psychiatry. 1997;12(5):224-31. doi: https://doi.org/10.1016/S0924-9338(97)83296-8.
66. Papke LE, Wooldridge JM. Econometric Methods for Fractional Response Variables With an Application to 401 (K) Plan Participation Rates. Journal of Applied Econometrics. 1996;11(6):619-32.
67. White IR, Royston P, Wood AM. Multiple imputation using chained equations: Issues and guidance for practice. Statistics in Medicine 2010;30:377-99.
68. van Buuren S. Flexible Imputation of Missing Data: CRC Press; 2012.
69. R: A language and environment for statistical computing. R Foundation for Statistical Computing V, Austria (https://www.R-project.org/).
70. Streiner DL, Norman GR. Correction for multiple testing: is there a resolution? . Chest. 2011;140(1):16-18.
71. Aubry T, Nelson G, Tsemberis S. Housing First for People with Severe Mental Illness Who are Homeless: A Review of the Research and Findings from the at Home—Chez soi Demonstration Project. The Canadian Journal of Psychiatry. 2015;60(11):467-74. doi: 10.1177/070674371506001102.
72. Hall G, Walters S, Gould H, Lim S. Housing versus treatment first for supportive housing participants with substance use disorders: A comparison of housing and public service use outcomes. Subst Abus. 2020;41(1):70-6. Epub 2018/03/13. doi: 10.1080/08897077.2018.1449049. PubMed PMID: 29528786.
73. Clifasefi SL, Collins SE. The life-enhancing alcohol-management program: Results from a 6-month nonrandomized controlled pilot study assessing a community based participatory research program in housing first. J Community Psychol. 2020;48(3):763-76. Epub 2019/11/30. doi: 10.1002/jcop.22291. PubMed PMID: 31778585.
74. Dietz TL. Substance Misuse, Suicidal Ideation, and Suicide Attempts Among a National Sample of Homeless. Journal of Social Service Research. 2010;37(1):1-18. doi: 10.1080/01488376.2011.524511.

75. Guillén AI, Marín C, Panadero S, Vázquez JJ. Substance use, stressful life events and mental health: A longitudinal study among homeless women in Madrid (Spain). Addictive Behaviors. 2020;103:106246. doi: https://doi.org/10.1016/j.addbeh.2019.106246.

76. Edalati H, Nicholls TL, Schütz CG, Somers JM, Distasio J, Aubry T, et al. Examining the Relationships between Cumulative Childhood Adversity and the Risk of Criminal Justice Involvement and Victimization among Homeless Adults with Mental Illnesses after Receiving Housing First Intervention. Can J Psychiatry. 2020;65(6):409-17. Epub 2020/01/30. doi: 10.1177/0706743720902616. PubMed PMID: 31994918; PubMed Central PMCID: PMCPMC7265607.

77. Mersky JP, Janczewski CE, Topitzes J. Rethinking the Measurement of Adversity. Child Maltreat. 2017;22(1):58-68. Epub 2016/12/07. doi: 10.1177/1077559516679513. PubMed PMID: 27920222.

78. Farrell SJ, Dunn M, Huff J. Examining Health Literacy Levels in Homeless Persons and Vulnerably Housed Persons with Mental Health Disorders. Community mental health journal. 2020;56(4):645-51. Epub 2019/12/21. doi: 10.1007/s10597-019-00525-2. PubMed PMID: 31858332.

79. O'Campo P, Daoud N, Hamilton-Wright S, Dunn J. Conceptualizing Housing Instability: Experiences with Material and Psychological Instability Among Women Living with Partner Violence. Housing Studies. 2016;31(1):1-19. doi: 10.1080/02673037.2015.1021768.

80. Hopper EK, Bassuk EL, Olivet J. Shelter from the storm: Trauma-informed care in homelessness services settings. The Open Health Services and Policy Journal. 2010;3(2), 80-100.

81. Bingham B, Moniruzzaman A, Patterson M, Sareen J, Distasio J, O'Neil J, et al. Gender differences among Indigenous Canadians experiencing homelessness and mental illness. BMC psychology. 2019;7(1):57. Epub 2019/08/29. doi: 10.1186/s40359-019-0331-y. PubMed PMID: 31455404; PubMed Central PMCID: PMCPmc6712855.

82. Yakubovich AR, Maki K. Preventing Gender-Based Homelessness in Canada During the COVID-19 Pandemic and Beyond: The Need to Account for Violence Against Women. Violence Against Women. 2021:10778012211034202. doi: 10.1177/10778012211034202.

Table 1: Baseline characteristics of women participants in the Canadian At Home/Chez Soi study by randomization group (*N* = 653)

Characteristics	Treatment as Usual (N = 279)		Housing First (N = 374)	
	N	%	N	%
Age (M ± SD), y	40.2 ± 11.7		39.7 ± 11.2	
Ethnic or cultural identity				
Aboriginal	76	27.2	95	25.4
Ethno-racial	67	24.0	81	21.7
White	136	48.8	198	52.9
Education				
Less than high school	130	46.9	202	54.3
Completed high school	57	20.6	61	16.4
Some post-secondary school	90	32.5	109	29.3
Marital status				
Married/Partnered	22	7.9	16	4.3
Divorced/Separated/Widowed	74	26.5	106	28.4
Single/Never married	183	65.6	251	67.3
Country of birth, & length of time in Canada for immigrants				
Born in Canada	235	84.8	315	84.2
Immigrant, 10 years or less	8	2.8	12	3.2

Characteristics	Treatment as Usual (N = 279)		Housing First (N = 374)	
	N	%	N	%
Immigrant, more than 10 years	34	12.3	47	12.6
Employment status				
Unemployed (unemployed, volunteer work, retired, student, housewife/husband, other)	270	96.8	362	97.3
Employed (including self-employed and special work program)	9	3.2	10	2.7
Sources of income				
Earnings from regular/casual work or busking	16	5.7	18	4.8
Unemployment insurance	22	7.9	26	7.0
Social assistance (disability, welfare, pension, personal needs allowance)	245	87.8	338	90.4
Selling papers, crafts, pan-handling, squeegee, or $ from recycling (bottles, scrap metal)	16	5.7	22	5.9
Other (loans, theft, sex work, help from family or friends)	12	4.3	10	2.7
Housing status				
Absolutely Homeless	215	77.1	296	79.1
Precariously housed	64	22.9	78	20.9
Lifetime duration of homelessness (M ± SD)[a], y	3.8 ± 4.6		3.8 ± 4.8	

Characteristics	Treatment as Usual (N = 279)		Housing First (N = 374)	
	N	%	N	%
Less than 3 years duration	155	57.8	211	58.0
3 years or more duration	113	42.2	153	42.0
Has minor children (≤18 yrs of age)				
0	154	56.0	221	59.1
1-2	81	29.4	110	29.4
3 or more	40	14.6	43	11.5
Has minor children (≤18 yrs of age) & provides full/partial support				
0	88	72.7	125	82.2
1	20	16.5	16	10.5
2 or more	13	10.7	11	7.2
Relationship to minor children (≤ 18 yrs of age)				
Biological parent	121	100.0	151	98.7
Partner's, adoptive or other adult relative's child	0	0	5	3.4
MCAS[b] score (M ± SD)	60.7 ± 8.0		61.8 ± 7.8	
MINI[c] diagnostic categories				
Depressive episode	170	60.9	218	58.3
Manic or hypomanic episode	50	17.9	55	14.7
Post-traumatic stress disorder	126	45.3	145	38.8
Panic disorder	94	33.7	102	27.3

Characteristics	Treatment as Usual (N = 279)		Housing First (N = 374)	
	N	%	N	%
Mood disorder with psychotic features	54	19.4	54	14.5
Psychotic disorder	81	29.0	95	25.4
Alcohol dependence	97	34.8	108	28.9
Substance dependence	134	48.0	167	44.7
Suicidality				
Not suicidal	46	16.5	62	16.6
Low	119	42.7	170	45.5
Moderate	50	17.9	70	18.7
High	64	22.9	72	19.3
Adverse Childhood Experiences Scale, mean (SD)	5.1	3.0	5.2	3.2
Less than 5	95	39.9	137	40.5
5 or more	143	60.1	201	59.5
Comorbid medical conditions				
Less than 3	51	18.6	100	27.0
3 or more	223	81.4	270	73.0
Study Component				
Moderate Needs	144	51.6	232	62.0
High Needs	135	48.4	142	38.0

[a] Median (IQR), y 2.0 (0.8-5.0) for Treatment as Usual and 2.0 (0.7-5.0) for Housing First ; [b] MCAS: Multnomah Community Ability Scale. Possible scores range from 17 to 85, with lower scores indicating more disability.;

[c] MINI: The Mini-International Neuropsychiatric Interview

Table 2. Post-Imputation Pooled change in health and social measures from Baseline to 24 Months among Women living with Mental illness and Homelessness, Adjusted for Study City, and Need Level (ACT or ICM).

	Housing First (HF) (N=374)		Treatment as Usual (TAU) (N=279)		p-value for differences between HF and TAU
~~Mean percentage of days spent stably housed over the 24 month follow-up.~~	~~74.8%~~	~~71.7%–77.8%~~	~~37.9%~~	~~34.4%–41.3%~~	~~0.001~~
**Continuous Outcomes (95% CI)**	Mean Change from Baseline to 24 Months	95% CI	Mean Change from Baseline to 24 Months	95% CI	p
Mental illness symptom severity (CSI[a])	-6.3	-7.5 to -5.0	-6.9	-8.4 to -5.4	0.534
Community functioning (MCAS[b])	3.8	2.8 - 4.9	4.8	3.6 - 6.0	0.236
Condition-specific QoL (QoLI-20[c])	16.0	13.6 - 18.4	13.4	10.6 - 16.2	0.178

Psychological community integration (CIS[d])	2.0	1.5 - 2.4	2.0	1.4 - 2.6	0.941
**Count Outcomes**	Rate Ratio Comparing 24 Months with Baseline	95% CI	Rate Ratio Comparing 24 Months with Baseline	95% CI	p
Physical community integration in past month (CIS[d])	0.97	0.86 - 1.08	1.03	0.92 - 1.14	0.439
Severity of substance problems in past month (GAIN-SS[e])	0.79	0.67 - 0.93	0.83	0.70 - 0.98	0.688
Number of emergency department visits in past 6 months	0.38	0.29 - 0.50	0.37	0.27 - 0.51	0.926

Note. Housing outcome is mean proportion of days spent stably housed.

[a] CSI: Colorado Symptom Index. Possible scores range from 14 to 70, with higher values indicating greater symptom severity.

[b] MCAS: Multnomah Community Ability Scale. Possible scores range from 17 to 85, with lower values indicating more disability.

[c] QoLI-20: Lehman Quality of Life Interview 20. Possible scores range from 20 to 140, with higher values indicating greater quality of life.

[d] CIS: Community Integration Scale. Possible scores range from 4 to 20 for the Psychological component and 0-7 for the Physical component, with higher values indicating greater integration.

[e] GAIN-SS: Global Assessment of Individual Needs Short Screener. Possible scores range from 0 to 5 with higher values indicating greater problem severity.

Table 3. Post-Imputation Pooled Comparisons between Housing First and Treatment as Usual at 6, 12, 18 and 24 months for Women in the At Home/Chez Soi Trial Adjusted for Study City, Need Level (ICM or ACT) and Baseline Outcome Values[f]

	Month	Housing First		Treatment as Usual		Comparison		
**Continuous Outcome**		**Mean**	**95% CI**	**Mean**	**95% CI**	**Mean Difference**	**95% CI**	**p**
Mental illness symptom severity (CSI[a])	6	35.8	34.8 - 36.8	36.8	35.6 - 38.0	-1.0	-2.5 to 0.5	0.183
	12	34.6	33.6 - 35.6	35.3	34.1 - 36.6	-0.7	-2.3 to 0.8	0.364
	18	33.8	32.7 - 35.0	34.1	32.7 - 35.4	-0.2	-2.0 to 1.5	0.785
	24	33.7	32.6 - 34.8	33.4	32.1 - 34.7	0.3	-1.4 to 2.0	0.737
Community functioning (MCAS[b])	6	64.9	64.1 - 65.7	64.9	64.0 - 65.9	-0.02	-1.2 to 1.2	0.975
	12	65.0	64.2 - 65.8	64.3	63.4 - 65.3	0.7	-0.6 to 1.9	0.299
	18	65.6	64.8 - 66.5	64.9	63.8 - 65.9	0.8	-0.6 to 2.1	0.267

	24	65.5	64.7 - 66.4	65.9	64.9 - 66.9	-0.4	-1.7 to 0.9	0.593
Condition-specific QoL (QoLI-20[c])	6	84.4	82.4 - 86.4	77.3	75.0 - 79.6	7.1	4.1 - 10.1	<.001
	12	87.4	85.4 - 89.4	81.3	79.0 - 83.7	6.1	3.1 - 9.2	<.001
	18	84.8	82.8 - 86.9	82.2	79.9 - 84.6	2.6	-0.4 to 5.6	0.091
	24	86.8	84.6 - 89.0	84.3	81.8 - 86.7	2.5	-0.7 to 5.8	0.124
Psychological community integration (CIS[d])	6	12.4	12.0 - 12.7	11.8	11.4 - 12.3	0.5	-0.1 to 1.1	0.077
	12	12.8	12.4 - 13.2	12.1	11.6 - 12.5	0.7	0.1 to 1.3	0.017
	18	12.7	12.3 - 13.1	12.3	11.8 - 12.7	0.4	-0.2 to 1.0	0.194
	24	12.9	12.5 - 13.2	12.7	12.2 - 13.1	0.2	-0.4 to 0.8	0.458
**Count Outcome**		**Rate**	**95% CI**	**Rate**	**95% CI**	**Rate Ratio**	**95% CI**	**p**
Physical community integration in past month (CIS[d])	6	2.1	1.9 - 2.3	2.1	1.9 - 2.3	0.98	0.87 - 1.12	0.789

	12	1.9 1.7 - 2.1	2.0 1.8 - 2.2	0.95 0.84 - 1.09	0.492
	18	2.0 1.8 - 2.1	2.0 1.8 - 2.2	0.99 0.87 - 1.12	0.858
	24	1.9 1.7 - 2.0	2.0 1.8 - 2.2	0.91 0.80 - 1.03	0.148
Severity of substance problems in past month (GAIN-SS[e])	6	1.2 1.0 - 1.4	1.2 1.0 - 1.5	0.97 0.78 - 1.20	0.767
	12	1.3 1.1 - 1.5	1.2 1.0 - 1.4	1.09 0.87 - 1.37	0.452
	18	1.1 0.9 - 1.3	1.2 0.9 - 1.4	0.74 1.20 - 0.63	0.628
	24	1.1 0.9 - 1.2	1.0 0.9 - 1.2	1.00 0.79 - 1.27	0.977
Number of emergency department visits in past 6 months	6	1.2 0.9 - 1.4	1.4 1.0 - 1.9	0.80 0.55 - 1.16	0.241
	12	1.1 0.7 - 1.4	1.1 0.7 - 1.6	0.96 0.58 - 1.57	0.858
	18	1.1 0.8 - 1.4	0.6 0.5 - 0.8	1.81 1.26 - 2.60	0.001
	24	0.8 0.6 - 0.9	0.7 0.5 - 0.9	1.13 0.83 - 1.55	0.426

a CSI: Colorado Symptom Index. Possible scores range from 14 to 70, with higher values indicating greater symptom severity.

b MCAS: Multnomah Community Ability Scale. Possible scores range from 17 to 85, with lower values indicating more disability.

c QoLI-20: Lehman Quality of Life Interview 20. Possible scores range from 20 to 140, with higher values indicating greater quality of life.

d CIS: Community Integration Scale. Possible scores range from 4 to 20 for the Psychological component and 0-7 for the Physical component, with higher values indicating greater integration.

e GAIN-SS: Global Assessment of Individual Needs Short Screener. Possible scores range from 0 to 5 with higher values indicating greater problem severity.

f Housing stability is not included in this table as we did not examine it at each follow-up point.

Table 4. Post-Imputation Pooled coefficient estimates (95% Confidence Interval) for Predictors in Linear Mixed Models for Repeated Measures for continuous outcomes and Rate Ratios (95% Confidence Interval) for Generalized Estimating Equations Models for count outcomes for the sample of women in the At Home/Chez Soi study (N=653).

	Mental illness symptom severity (CSI)			Community functioning (MCAS)			Condition-specific QoL (QoLI-20)			Psychological community integration (CIS)		
	Est	95% CI	p-val	Est	95% CI	p-val	Est	95% CI	p-val	Est	95% CI	p-val
Age (years)	-0.01	-0.06- 0.04	0.680	-0.02	-0.060-.02	0.310	-0.06	-0.17- 0.04	0.250	-0.01	-0.03- 0.00	0.140
Less than high school	0.21	-0.98- 1.40	0.731	**-1.32**	**-2.27 to -0.37**	**0.006**	0.87	-1.56- 3.30	0.480	0.15	-0.26- 0.55	0.476
3 or more years of homelessness	0.91	-0.27- 2.09	0.132	**-1.75**	**-2.72 to -0.78**	**<.001**	-0.75	-3.14- 1.64	0540	-0.07	-0.48- 0.33	0.728
Supports ≥1 child under 18 years	0.11	-1.89- 2.10	0.917	0.62	-0.96- 2.20	0.442	0.47	-3.52- 4.47	0.820	0.48	-0.20- 1.17	0.168
Moderate or high suicidality at baseline	**2.85**	**1.59- 4.11**	**<.001**	**-1.16**	**-2.10 to -0.22**	**0.015**	**-3.99**	**-6.49 to -1.49**	**<.001**	-0.30	-0.71- 0.11	0.150
5 or more ACE	**1.50**	**0.25- 2.76**	**0.019**	0.21	-0.75- 1.17	0.666	-0.56	-3.14- 2.02	0.670	0.31	-0.12- 0.73	0.157

	Physical community integration in past month (CIS) (N=653)			Severity of substance problems in past month (GAIN-SS) (N=653)			Number of emergency department visits in past 6 months (N=653)			Stable housing (N=653)		
		95% CI	p-val	RR	95% CI	p-val	RR	95% CI	p-val	beta* or Odds ratio **	95% CI	p-val
Age (years)	1.0	1.0- 1.0	0.834	**0.99**	**0.98-1.00**	**0.003**	1.00	0.99- 1.01	0.920	0.16<br>1.01	-0.04-0.36<br>1.00-1,02	0.122<br>0.1021
Less than high school	**0.85**	**0.77- 0.93**	**<.001**	**1.27**	**1.06-1.52**	**0.009**	1.11	0.83-1.49	0.474	-2.78<br>0.87	-7.43-1.88<br>0.69-1.09	0.242<br>0.222
3 or more years of homelessness	0.98	0.93- 1.11	0.720	**1.21**	**1.03- 1.43**	**0.021**	0.99	0.76-1.29	0.951	**-6.14**<br>**0.74**	**-10.9--1.32**<br>**0.58-0.94**	**0.013**<br>**0.013**
Supports ≥1 child under 18 years	1.10	0.96-1.27	0.176	1.05	0.80- 1.39	0.715	0.92	0.67-1.26	0.598	5.57<br>1.31	-2.17-13.3<br>0.89-1.92	0.158<br>0.171
Moderate or high suicidality at baseline	1.02	0.93-1.12	0.634	1.06	0.90- 1.27	0.465	**1.44**	**1.10-1.87**	**<0.001**	-1.82<br>0.91	-6.49-2.80<br>0.73-1.15	0.440<br>0.432

5 or more ACE	1.04	0.95-1.15	0.356	1.16	0.96- 1.39	0.114	1.05	0.77-1.42	0.772	-2.93<br>2.93<br>1.16	-7.92-2.05<br>-2.05-7.92<br>0.90-1.48	0.249<br>0.249<br>0.245

Each model includes treatment group, categorical time, interaction of treatment x time, city, need level, baseline outcome values and all baseline predictors. For percentage or proportion of days stably housed over 24 months, time and interaction treatment by time are not included in linear* and fractional logistic models**

Supporting Information

Figure S1 – Flow of participant enrolment, assignment to treatment arm, and inclusion in analysis

Supplemental Table S1– Missing Rates (%) for Outcomes by Group, Time Period and Overall

Supplemental Table S2- SAS Syntax for Regression Models

Reviewer comment	Author response.
(regarding "days spent stably housed)<br>If there are 0s and 1s that are structurally important, then a zero-one inflated beta regression (ZOIB) is the most appropriate choice.	We are surprised by how much effort this reviewer places on raising questions about our statistical modeling of the outcome related to housing stability as we have provided a sound rationale for our choice of our statistical model and the findings are so strong that the choice of model is not likely to change our conclusions. Nevertheless, to address the reviewer's concern, we fit an additional model, the fractional logistic model [(1)] for the proportion of days stably housed over 24 months instead of fitting the ZOIB model. The model recommended by the reviewer, the ZOIB, is not appropriate as it requires the assumption that 0's and 1's represent very distinct processes from the process which generated the continuous values, which we do not believe they do.<br><br>We are able to illustrate, that, for our data, the linear model and the fractional logistic model yield exactly the same conclusions.<br><br>The fractional logistic model [(1)] uses a quasi-likelihood function for estimation and is appropriate for proportion outcomes when the values of 0 and 1 are admissible and observable. The fractional logistic model predicts the mean of the proportion conditional on covariates. To ensure that the predicted mean is also between 0 and 1, the logit model for the mean is considered.<br><br>The parameters estimated from this model can be exponentiated to yield odds ratios. We now include both analyses in our paper. See the revisions in the Methods section and Tables of findings.
(regarding the issue of multiple testing)<br>I am not sure then what is the use of p-values if they are clearly affected by multiple testing and if they are not relevant. Perhaps it is worth dropping them altogether. Up to the authors to decide what to do.	We do appreciate the reviewer's comments, but we do stand by our position, that there is no consensus in the literature on the issue of multiple testing for exploratory studies, as indicated in the references we provided in our previous responses. For this reason, we believe that it is not a matter of who is right or wrong, but rather, which view one is more comfortable endorsing. Furthermore, we do have to focus on the intention of the paper that is to explore the wealth of data for homeless women with mental illness (rather than to test very specific hypotheses) and to generate hypothesis for future studies to test or use in their evaluation of interventions. Therefore, as permitted by this reviewer, we decided to keep the p-values, as well as confidence intervals, in our paper. We appreciate that the reviewer leaves the final decision on this point up to us.
However, if it is the case that linear models where used to analyse the longitudinal data, then	We do not believe GAMs are necessary in our case since our models include a categorical time variable and we are not interested in deriving complex trajectories.

I am afraid I cannot reconstruct what was done based on the highlighted sentence.* It is necessary to include a full account of how the models were constructed, in details. If the authors do have the resources to run GAMs on the longitudinal data (as I gather from their response) then they should proceed with it.<br><br>**highlighted sentence: "Longitudinal outcomes were analyzed using linear mixed analysis of repeated measures models for continuous outcomes and generalized estimating equations for count outcomes (Poisson distribution for CIS-Physical Integration and negative binomial distribution for GAIN- SS and ED Visits), assuming an unstructured correlation matrix for the repeated measures."*	Regarding the specification of the models, we have provided as much detail that is typically provided in journal articles. Space limitations preclude us describing the specifics of each and every model we fit. Thus, to help the reviewer and readers completely understand what was done, we now include the SAS syntax in the Supplemental information, S3. We do provide the code for each objective and each model presented in the results Tables so we hope this will make clear the details about each model.
I still cannot find in the manuscript any mention to where the analysis scripts/software instructions to reproduce the analysis can be found. (I understand the data are not available, but that doesn't prevent one to share analysis scripts/instructions)	The SAS syntax for objectives and outcomes is now included in Supplemental Table 3.

1. Papke LE, Wooldridge JM. Econometric Methods for Fractional Response Variables With an Application to 401 (K) Plan Participation Rates. Journal of Applied Econometrics. 1996;11(6):619-32.
