# Supplementary material for: Women experiencing homelessness and mental illness in a Housing First multi-site trial: Looking beyond housing to social outcomes and well-being
Source: PLoS One. 2023 Feb 10;18(2):e0277074. doi: 10.1371/journal.pone.0277074 (PMC9916643; doi:10.1371/journal.pone.0277074)
Supplement: Attachment — Submitted filename: Response to Reviewers PONE-D-21-13748.docx [file pone.0277074.s002.docx]

|  | Editor or Reviewer comments | Author responses |
| --- | --- | --- |
| Editor comments | | |
| 1 | Regarding missingness, it’s important to address the missing data mechanisms in more depth than currently, and also whether these mechanisms result in data that are missing at random, missing completely at random, or even not missing at random. Given the diverse reasons for missingness, it is possible that more than one type of missingness is present in the data. | Missing data mechanisms have been widely presented and discussed in the literature. Although the Missing Completely at Random mechanism simplifies analyses, it is quite difficult to find real life applications that satisfy its assumption [VanBuuren2012], and therefore we did not consider it in our data.  We did however consider the Missing at Random (MAR) Mechanism, where the probability of missingness depends only on observed data but not on the data that are missing. That is, after controlling for or stratifying by observed variables, missingness is random. We followed recommendation from Van Buuren 2012 and Barnard 1999 to include in our imputation models a high the number of predictors of the incomplete variables in order to make the MAR assumptions more plausible.  Van Buuren, S. (2012), Flexible Imputation of Missing Data. Chapman & Hall/CRC, Boca Raton, FL.  Barnard J, Meng XL Applications of multiple imputation in medical studies: from AIDS to NHANES. Statistical Methods in Medical Research. 8:17-36, 1999 |
| 2 | 70% of data had at least one missing value. With such a large percent like 70%, a reader might think that is in the entire dataset. It seems from context and supplementary table 1 that this was all variables that are used in this paper’s analysis, so please say that more clearly.  Also given that 70% of data used in the study had at least one missing value, please cite research about the implications of MI in data with such a large percent missing. | We followed recommendations from White et al, 2010 and Horton et al. 2007 who suggest that depending on the number and type of variables, and amount of missing data, 50 to 100 imputations yield better estimates. However, a rule of thumb was provided by White et al. 2010 that the number of imputations should be at least 100 x (percent of incomplete cases) and we applied this recommendation to our data analysis approach.  Horton, N. & Kleinman, K.P. (2007). Much ado about nothing: A comparison of missing data methods and software to fit incomplete data regression models. The American Statistician 61(1): 79-90. Software appendix: <http://www.math.smith.edu/muchado-appendix.pdf>  White, I.R., Royston, P., and Wood, A.M. (2010). Multiple imputation using chained equations: Issues and guidance for practice. Statistics in Medicine 30:377-399. (Includes Stata codes) |
| 3 | One reviewer asked for sensitivity analysis for imputed versus non-imputed. Diagnostics of MI method may be appropriate. | As requested, we provide a sample of multiple imputation diagnostic plots as suggested by Van Buuren, 2012 at the end of this document.  We also added the following sentence to the methods: Comparisons between observed and imputed data via diagnostic plots were used to assess the quality and plausibility of the imputations.  Van Buuren, S. (2012), Flexible Imputation of Missing Data. Chapman & Hall/CRC, Boca Raton, FL. |
| 4 | With regard to sample size calculation, an alternative to a standard power calculation would be Gelman and Carlin type-M and type-S errors, which is less centered on p-values. | Because ours was a post-hoc subgroup exploratory analysis of the main study, the sample size calculation which was predetermined for the main hypotheses for the main study involving the full sample, does not apply here. As such, for the analyses we present here, we would like to emphasize that our study was hypothesis generating and therefore power calculations do not apply. The original sample sizes were calculated for the original study, please refer to protocol in Goering et al.  Goering PN, Streiner DL, Adair C, Aubry T, Barker J, Distasio J, Hwang SW, Komaroff J, Latimer E, Somers J, Zabkiewicz DM. The At Home/Chez Soi trial protocol: a pragmatic, multi-site, randomised controlled trial of a Housing First intervention for homeless individuals with mental illness in five Canadian cities. BMJ Open. 2011 Nov 14;1(2):e000323 |
| 5 | The effects of the experiment have been displayed as p-values.  An effect size measure such as Hedge’s g or Cohen’s d in addition to the differences that are in the tables 2-4. These effect size measures may give a clearer picture of the true results of the experiment. | We are familiar with Cohen’s d and the Hedge’s g correction as alternate ways of illustrating differences between treatment and control groups. We did not use Cohen’s d in any of our dozens of publications that emerged from this randomized trial as in our experience it is not as common in health or medical studies as it would be in psychology. In fact, a quick review of evaluations or randomized trials published in Plos One over a one-year period (back to Dec 2020, see citations below) demonstrated that these studies report findings in a similar way to which we presented our findings. Evaluations presented mean values of outcomes for treatment and controls groups along with 95% confidence intervals and p-values. Some studies add in the t-statistics. As such, we feel that adding Cohen’s d values were not necessary.  Hajek P, Przulj D, Pesola F, McRobbie H, Peerbux S, Phillips-Waller A, et al. (2021) A randomised controlled trial of the 5:2 diet. PLoS ONE 16(11): e0258853.  Ngugi P, Babic A, Were MC (2021) A multivariate statistical evaluation of actual use of electronic health record systems implementations in Kenya. PLoS ONE 16(9): e0256799.  Müller SA, Diallo AOK, Rocha C, Wood R, Landsmann L, Camara BS, et al. (2021) Mixed methods study evaluating the implementation of the WHO hand hygiene strategy focusing on alcohol based handrub and training among health care workers in Faranah, Guinea. PLoS ONE 16(8): e0256760.  Andersen TO, Dissing AS, Varga TV, Rod NH (2021) The SmartSleep Experiment: Evaluation of changes in night-time smartphone behavior following a mass media citizen science campaign. PLoS ONE 16(7): e0253783.  Greene MC, Likindikoki S, Rees S, Bonz A, Kaysen D, Misinzo L, et al. (2021) Evaluation of an integrated intervention to reduce psychological distress and intimate partner violence in refugees: Results from the Nguvu cluster randomized feasibility trial. PLoS ONE 16(6): e0252982.  Heimgartner N, Meier S, Grolimund S, Ponti S, Arpagaus S, Kappeler F, et al. (2021) Randomized controlled evaluation of the psychophysiological effects of social support stress management in healthy women. PLoS ONE 16(6): e0252568.  Dardas A, Williams A, Wang L (2021) Evaluating changes in workplace culture: Effectiveness of a caregiver-friendly workplace program in a public post-secondary educational institution. PLoS ONE 16(5): e0250978.  O’Donnell A, Wilson L, Bosch JA, Borrows R (2020) Life satisfaction and happiness in patients shielding from the COVID-19 global pandemic: A randomised controlled study of the ‘mood as information’ theory. PLoS ONE 15(12): e0243278. |
| 6 | P-values have been displayed to 3 digits in all tables. That’s too many digits and gives false precision.  Just one significant digit is fine. For instance, in table 2 the p-values would be 0.001 (as is currently there), 0.5, 0.2, 0.2, 0.9, 0.4, 0.7, 0.9. | We respectfully disagree with this suggestion to report p-values using 1 significant digit. We not only feel this is imprecise but this practice is not aligned with what we discovered in our review of studies published in Plos One. Studies reviewed (see item above) reported p-values using 3 digits or sometimes more significant digits. Therefore, we have not reduced the number of significant digits we report for our p-values in our tables and text. |
| 7 | Figure 1 is really nice display of results, but the treated and control groups should be shifted slightly so the confidence intervals are clearer and don’t overlap— e.g., intervention at 0.5 months below each time point and control at 0.5 months above each time point. Many of these continuous variable outcomes in figure 1 probably have complex distributions, and it may be helpful to show the distributions of these variables for treatment versus control, such as using a violin plot or similar data display, rather than the point and CI shown in figure 1. | Thank you for your appreciation of our figures. The figures are intended to augment the study findings in the tables as Table 3 presents results of multivariate analyses. The Figures with the unadjusted results are intended to be a visual supplement to Table 3 as the differences or lack thereof are easier to grasp quickly in the figures. While CIs are included in the Figures to help identify whether the values at any one time point differ between treatment and TAU, the data in the Tables reporting multivariate regression findings should be the primary source of information about differences and significance of those differences. To change the scales on the figures to further separate the trend lines for the treatment and TAU would create larger figures that would take up too much space and is not necessary given that the multivariate findings are presented in Table 3. |
| 8 | The tables are somewhat hard to read with many lines, and they may benefit from using data displays such as dumbbell plots for comparisons of the treatment vs control. | We have added formats to the table to increase readability. In particular, we have shaded every other row in Tables 1, 3 and 4. We have added lines between each outcome to more clearly delineate the findings for each outcome and co-variate. We believe the format changes have considerably increased the readability of the Tables. Moreover the figures we provided as supplementary information about Table 3 is a visual aid to rapidly understanding the findings from the analysis of these trial data. |
| 9 | The mixed models should report evaluation of assumptions of the models, such as linear regression, Poisson regression. | To respond to this suggestion we have added the following text in the Statistical analysis section: Normality of residuals was graphically checked for linear mixed models. The decision to fit a negative binomial rather than a Poisson model was based on whether over-dispersion (, ie, if the variance was much larger than the mean) was present in the count outcome data. |
| 10 | Table 2 shows percent of the time housed over all visits. However, the other measures in table 2 are a comparison of baseline to 24 months adjusted for city and need level, which leaves out the middle 3 follow-up visits.  It seems like these observations in table 2 could be more completely displayed as an adjusted version of Figure 1 that includes all time points adjusted for city and need level. | There were several reasons why our study measured housing success as *cumulative stability at 24 months* and we now state this in the methods. First, given the Housing First Theory of Change and Program Manual, stable housing is established within the first 1-3 months of enrolment for those in the treatment arm and housing status rarely changes after that point in time. Thus, we wanted to align with the program’s theory of change. Second, to measure housing status at different time points over the 24 months means that we would have seen very little change for those in the treatment arm and a single cumulative measure made more sense for this outcome. This measure captures overall stability and instability in that a small proportion of the treatment group was not stably housed throughout and this single measure captures that information. Third, this single measure captures the overall housing stability or instability across the two groups over a long period of time and for this outcome, knowing the housing status at different time points in the study is less relevant to the overall goals of the program. In sum, we chose the best measure for the outcome of housing even if it did not match how we approached other outcomes such as quality of life which is expected to demonstrate incremental changes over the 24-month follow-up period. |
| 11 | Table 4 shows mixed model effects that leaves out the treatment effect and treatment x time, among other variables. It seems that this table should at least include treatment effect coefficients.  Omitting the main effect of treatment doesn’t avoid table 2 fallacy. The authors should address whether table 2 fallacy may apply here. | Table 4 presents the findings for the third objective of the paper (see bottom of page 7 of the manuscript for the objective) intended to “explore baseline demographic, social, health measures, and ACEs as predictors of key study outcomes during the study period.” This objective is not concerned with differences between treatment and TAU. In fact, we include that objective because few differences between treatment and TAU were reported and we can, therefore, take advantage of this large sample of homeless women to learn more about predictors of our key outcomes. Consequently, our analysis for this objective does not account for treatment group membership by design. We do not address the issue of type 2 fallacy for this objective as it does not apply. However, we have made our intent clearer in the statistical analyses, see top of page 11 and in the results on page 13 |
| 12 | 3. Please provide additional details regarding participant consent. In the ethics statement in the Methods and online submission information, please ensure that you have specified what type you obtained (for instance, written or verbal, and if verbal, how it was documented and witnessed). If your study included minors, state whether you obtained consent from parents or guardians. If the need for consent was waived by the ethics committee, please include this information. | We have expanded the description of the consent process and Ethics Board approval process on page 8 of the revised manuscript. |
| 13 | 4. We note that the grant information you provided in the ‘Funding Information’ and ‘Financial Disclosure’ sections do not match. | We tried upon our initial submission to create a new entry online for our funding agency, the Mental Health Commission of Canada. However, when we try to add this new funding agency to your database the system freezes and cannot seem to complete the task. Therefore we did not do this task but are willing if we get assistance to do so. We did also try to email the generic help email but received an unhelpful response that we should enter the information into the field with no guidance on how to overcome our problem. |
| 14 | When you resubmit, please ensure that you provide the correct grant numbers for the awards you received for your study in the ‘Funding Information’ section. | There was no grant number for this award. |
| Reviewer comments | | |
| 15 | Reviewer #1: Overall, the study sheds important light on the effectiveness of Housing First intervention among women with mental illness. However, there were a few areas that could be strengthened. In the introduction section, beyond the scant literature on women in permanent supportive housing programs, the authors should consider strengthening the significance of the study. A clearly stated rationale is warranted for the study. | We have inserted a strong rationale for this study that focuses on the unique features of the data and the rationale for focusing on just women in this analysis. |
| 16 | The methods section includes instruments that have strong psychometric properties in the literature. However, the authors do not report any reliability coefficient of the instruments. Further, no testing of group differences on demographic characteristics were reported. The reasons for selecting predictor and control variables are unclear. | We chose instruments with high reliability and validity for this multi-city randomized trial. In this paper, we described the instruments as we have previously done in our published work (see below for example papers). Unfortunately we did not calculate the reliability of coefficients for the individual scales using our data.  Stergiopoulos V, Hwang SW, Gozdzik A, Nisenbaum R, Latimer E, Rabouin D, Adair CE, Bourque J, Connelly J, Frankish J, Katz LY, Mason K, Misir V, O'Brien K, Sareen J, Schütz CG, Singer A, Streiner DL, Vasiliadis HM, Goering PN; At Home/Chez Soi Investigators. Effect of scattered-site housing using rent supplements and intensive case management on housing stability among homeless adults with mental illness: a randomized trial. JAMA. 2015 Mar 3;313(9):905-15. doi: 10.1001/jama.2015.1163. PMID: 25734732.  Aubry T, Goering P, Veldhuizen S, Adair CE, Bourque J, Distasio J, Latimer E, Stergiopoulos V, Somers J, Streiner DL, Tsemberis S. A Multiple-City RCT of Housing First With Assertive Community Treatment for Homeless Canadians With Serious Mental Illness. Psychiatr Serv. 2016 Mar;67(3):275-81. doi: 10.1176/appi.ps.201400587. Epub 2015 Dec 1. PMID: 26620289.  Following guidelines from CONSORT 2010 Explanation and Elaboration  Moher D,Hopewell S,Schulz K F, Montori V, GÃ¸tzsche P C, Devereaux P J et al. CONSORT 2010 Explanation and Elaboration: updated guidelines for reporting parallel group randomized trials BMJ 2010; 340 :c869 doi:10.1136/bmj.c869  We did not include p-values in Table 1, as any differences in our two groups from this randomized sample are expected to occur by chance. In addition, many of the variables in Table 1 are included for descriptive purposes only. For the purposes of this reply to reviewer suggestions, however, we did perform tests for selected variables and none had p <0.05, except study component (p=0.0077) and number of Comorbid medical conditions (P=0.0127). We have provided this information below in a table for the purposes of this response. |
| 17 | Reviewer #2: This manuscript reports a secondary analysis of the original Canadian five city Housing First trial focused solely on female participants. The authors largely repeat the analyses previously reported, but with a specific focus on women; they also add an examination of specific risk factors that might be associated with differential outcomes for women.  Because of the size and innovation of the original study, the secondary analysis is still able to provide some new insights and considerations in an important subsample. As the authors note, many investigators have argued for separate studies or at least distinguishing analyses to understand the numerous differences reported among men and women experiencing homelessness. Moreover, the homelessness epidemic has only worsened in the context of the COVID pandemic so the issues remain relevant if these new analyses can provide insights.  Several strengths are evident besides the original sample size and randomization. First, the authors do include novel risk/resilience factors that might alter the outcomes for women experiencing homelessness such as the caretaking status of dependent children, exposure to ACES, and others. These factors have not been sufficiently considered in earlier studies of Housing First. Secondly, the authors examined group by time effects and group differences at each interval to assess the relative changes from the intervention. Thirdly, they provide detailed reporting on the individual outcomes and the relative importance or size of the changes. | Thank you for this summary and suggestion. We have inserted a strong rationale for this study that focuses on the unique features of the data and the rationale for focusing on just women in this analysis. |
| 18 | Some changes would also strengthen the manuscript. The most important is better clarity and continuity among the introduction, the results and the discussion. Para 3 of the intro makes a strong argument for providing data for women experiencing homelessness. However, para 5 points to studies that tried and found no differences. Why will this study be different than those studies? | Thank you for pointing out this inconsistency. We have re-written this section of the Introduction. We now make it clear that those studies that examined women had very small sample sizes. Those large studies were observational and also were based upon comparing genders and not focusing on heterogeneity of the women in the sample. With the additional of text we distinguish our study from what is currently in the literature. |
| 19 | Similarly, in the results, the authors found intervention women did NOT differ on most assessments with the exception of stably housed days (the primary intervention). However, this is different than the original finding of the five city study where all participants (men and women together) had improved quality of life and integration. No comment or discussion on this ‘lessening’ of effects for women is provided.  This theme continues into the discussion where the authors conclude with the need for more studies of women even though they find no clear benefit from separate analysis in this example nor in the cited studies in their introduction. Reconciling their results, introduction and discussion seem like a critical piece because it is the main contribution of the study rather than some of the ancillary factors they look at. | We have clarified this point in the paper. In fact, the results for this subsample of women and those for the full sample align and we have noted this now in the discussion. |
| 20 | Tables 3 and 4 are difficult to read given the multitude of comparisons that are made. | We have applied better formatting for the tables making them easier to read. Specific changes are described above. |
| 21 | Reviewer #3: Thank you for the opportunity to review this important work. This study examined the social, health, and housing needs of women experiencing homelessness. It will make an important contribution to the evidence base as the experiences of this population have received little attention. The strengths of this study include a large sample of women experiencing homelessness in the context of a multi-site randomized controlled Housing First trial, a women-only analysis, and baseline factors and outcomes that have not been fully explored in past research. However, there are some comments that need to be addressed, including questions about the statistical analysis, and adding to the discussion section.  Is the manuscript technically sound? And do the data support the conclusions?  Yes, the manuscript is technically sound, and the methods and analysis are clearly described to allow for replication. Data are also consistent with discussion section and conclusions. There are a few items that needs to be addressed: - Please describe baseline predictors and how they were coded in the methods section - For women with children in the sample, were any living with their children? What type of support was provided to children? Financial? Social? - Study findings are very interesting and surprising, particularly the lack of significant findings associated with health and social outcomes. Can prior research provide an explanation for the decline in ED visits for the TAU group? Were there any demographic characteristics that could have influenced the findings (marital status, racial/ethnic group, sources of income, having children, mental health diagnosis, comorbid conditions)?  - The first half of the discussion section is a bit thin and reads more like a results section than a discussion section. Consider cutting down sentences that restate the results and incorporate more explanation of findings using past research. - This sentence is confusing: “Contrary to what we might have anticipated on the importance of ACEs on multiple study outcomes based upon prior demonstrating the association of ACEs with victimization and criminal justice involvement, in our study high baseline ACE scores only predicted significantly worse scores on mental illness severity (CSI).” (Line 298-300)  Has the statistical analysis been performed appropriately and rigorously?  Yes, the statistical analysis has been performed appropriately and rigorously. Please address the following comments: - Please add statistical results for linear mixed models to the abstract - Consider running a sensitivity analysis comparing imputed vs. non-imputed data - Why were no adjustments made to account for multiple tests? - Please include sample size calculations  Have the authors made all data underlying the findings in their manuscript fully available?  Yes, all data used in the manuscript has been made fully available.  Is the manuscript presented in an intelligible fashion and written in standard English?  Yes, the manuscript is clear and well-written. | We have added more information about our study variables. Specifically we added information to the methods about any scales we used (e.g., ACEs). We state in the results that support of children refers to partial or full financial support. We did not find any prior studies to explain the small difference in favor of TAU at 18 months for ED visits. We also explain in the methods the rationale for our selection of predictor variables for the statistical models for our third objective which did not include variables such as marital status. We have rewritten the Discussion so that it is more closely linked to the existing literature and is less like a repeat of the results section. We have also fixed the sentence that was unclear (e.g., Contrary to what….).  We have added the results of the linear regression to the abstract. Regarding sample sizes and sensitivity analyses we have provided clarification above in an earlier response.  We added the specific estimates in the sentence below of the abstract  Suicidality was a consistent predictor of increased mental health symptoms (beta=2.85, 95% CI 1.59-4.11, p<0.001), decreased quality of life (beta=-3.99, 95% CI -6.49 to -1.49, p<0.001), decreased community functioning (beta= -1.16, 95% CI -2.10 to -0.22, p=0.015) and more emergency department visits (rate ratio = 1.44, 95% CI 1.10-1.87, p<0.001) over the study period. Lower education was a predictor of lower community functioning (beta=-1.32, 95% CI -2.27 to -0.37, p=0.006) and higher substance use problems (rate ratio=1.27, 95% CI 1.06-1.52, p=0.009) during the study.  No adjustment for multiple testing was applied as we followed the guidelines in the reference below and which were observed in all of our previous publications  Streiner DL, Norman GR. Correction for multiple testing: is there a resolution? *Chest*. 2011; 140(1):16-18.  We do not have information about whether women with children were living with them or which type of support was provided to the children. The information we have is reported in Table 1: 121/275 (44%) women in the TAU and 153/374 (41%) in the HF group reported having at least 1 child under the age of 18. Among those women who had at least 1 child under 18, 33/121 (27.3%) in the TAU and 27/152 (17.8%) in the HF group reported providing full or partial support to at least 1 child. |

Supplemental Material for Response to Editor and Reviewers PONE-D-13748

Selected Diagnostic Plots for Intervention Group and Outcomes at 24 months

Multiple Imputation First 10 Imputations

Community Physical Integration

Colorado Symptom Index scores

Number of Emergency Room Visits in past 6 months

Global Assessment of Individual Needs Short Screener scores

Multnomah Community Ability Scale scores

Supplemental information regarding p-values for Table 1.

Table 1: Baseline characteristics of women participants in the Canadian At Home/Chez Soi study by randomization group (*N* = 653)

| **Characteristics** | **Treatment as Usual (N = 279)** | | **Housing First**  **(N = 374)** | | **p-value** |
| --- | --- | --- | --- | --- | --- |
|  | N | % | N | % |  |
| Age (M ± SD), y | 40.2 ± 11.7 |  | 39.7 ± 11.2 |  | 0.5922 |
| Ethnic or cultural identity |  |  |  |  | 0.5633 |
| Aboriginal | 76 | 27.2 | 95 | 25.4 |  |
| Ethno-racial | 67 | 24.0 | 81 | 21.7 |  |
| White | 136 | 48.8 | 198 | 52.9 |  |
| Education |  |  |  |  | 0.1543 |
| Less than high school | 130 | 46.9 | 202 | 54.3 |  |
| Completed high school | 57 | 20.6 | 61 | 16.4 |  |
| Some post-secondary school | 90 | 32.5 | 109 | 29.3 |  |
| Marital status |  |  |  |  | 0.1482 |
| Married/Partnered | 22 | 7.9 | 16 | 4.3 |  |
| Divorced/Separated/Widowed | 74 | 26.5 | 106 | 28.4 |  |
| Single/Never married | 183 | 65.6 | 251 | 67.3 |  |
| Country of birth, & length of time in Canada for immigrants |  |  |  |  | 0.9650 |
| Born in Canada | 235 | 84.8 | 315 | 84.2 |  |
| Immigrant, 10 years or less | 8 | 2.8 | 12 | 3.2 |  |
| Immigrant, more than 10 years | 34 | 12.3 | 47 | 12.6 |  |
| Employment status |  |  |  |  | 0.6867 |
| Unemployed (unemployed, volunteer work, retired,  student, housewife/husband, other) | 270 | 96.8 | 362 | 97.3 |  |
| Employed (including self-employed and special work  program) | 9 | 3.2 | 10 | 2.7 |  |
| Sources of income |  |  |  |  | not applicable, just descriptive |
| Earnings from regular/casual work or busking | 16 | 5.7 | 18 | 4.8 |  |
| Unemployment insurance | 22 | 7.9 | 26 | 7.0 |  |
| Social assistance (disability, welfare, pension, personal  needs allowance) | 245 | 87.8 | 338 | 90.4 |  |
| Selling papers, crafts, pan-handling, squeegee, or $ from  recycling (bottles, scrap metal) | 16 | 5.7 | 22 | 5.9 |  |
| Other (loans, theft, sex work, help from family or friends) | 12 | 4.3 | 10 | 2.7 |  |
| Housing status |  |  |  |  | 0.5232 |
| Absolutely Homeless | 215 | 77.1 | 296 | 79.1 |  |
| Precariously housed | 64 | 22.9 | 78 | 20.9 |  |
| Lifetime duration of homelessness (M ± SD)^a^, y | 3.8 ± 4.6 |  | 3.8 ± 4.8 |  | 0.5822 |
| Less than 3 years duration | 155 | 57.8 | 211 | 58.0 |  |
| 3 years or more duration | 113 | 42.2 | 153 | 42.0 |  |
| Has minor children (≤18 yrs of age) |  |  |  |  | 0.6995 |
| 0 | 154 | 56.0 | 221 | 59.1 |  |
| 1-2 | 81 | 29.4 | 110 | 29.4 |  |
| 3 or more | 40 | 14.6 | 43 | 11.5 |  |
| Has minor children (≤18 yrs of age) & provides full/partial support |  |  |  |  | 0.1683 |
| 0 | 88 | 72.7 | 125 | 82.2 |  |
| 1 | 20 | 16.5 | 16 | 10.5 |  |
| 2 or more | 13 | 10.7 | 11 | 7.2 |  |
| Relationship to minor children (≤ 18 yrs of age) |  |  |  |  | descriptive |
| Biological parent | 121 | 100.0 | 151 | 98.7 |  |
| Partner's, adoptive or other adult relative's child | 0 | 0 | 5 3.4 |  |  |
| MCAS^b^ score (M ± SD) | 60.7 ± 8.0 |  | 61.8 ± 7.8 |  | 0.0825 |
| MINI^c^ diagnostic categories |  |  |  |  |  |
| Depressive episode | 170 | 60.9 | 218 | 58.3 | 0.4962 |
| Manic or hypomanic episode | 50 | 17.9 | 55 | 14.7 | 0.2685 |
| Post-traumatic stress disorder | 126 | 45.3 | 145 | 38.8 | 0.0931 |
| Panic disorder | 94 | 33.7 | 102 | 27.3 | 0.0767 |
| Mood disorder with psychotic features | 54 | 19.4 | 54 | 14.5 | 0.0974 |
| Psychotic disorder | 81 | 29.0 | 95 | 25.4 | 0.3009 |
| Alcohol dependence | 97 | 34.8 | 108 | 28.9 | 0.1086 |
| Substance dependence | 134 | 48.0 | 167 | 44.7 | 0.3919 |
| Suicidality |  |  |  |  | 0.7096 |
| Not suicidal | 46 | 16.5 | 62 | 16.6 |  |
| Low | 119 | 42.7 | 170 | 45.5 |  |
| Moderate | 50 | 17.9 | 70 | 18.7 |  |
| High | 64 | 22.9 | 72 | 19.3 |  |
| Adverse Childhood Experiences Scale, mean (SD) | 5.1 | 3.0 | 5.2 | 3.2 | 0.8382 |
| Less than 5 | 95 | 39.9 | 137 | 40.5 |  |
| 5 or more | 143 | 60.1 | 201 | 59.5 |  |
| Comorbid medical conditions |  |  |  |  | 0.0127 |
| Less than 3 | 51 | 18.6 | 100 | 27.0 |  |
| 3 or more | 223 | 81.4 | 270 | 73.0 |  |
| Study Component |  |  |  |  |  |
| Moderate Needs | 144 | 51.6 | 232 | 62.0 | 0.0077 |
| High Needs | 135 | 48.4 | 142 | 38.0 |  |

^a^ Median (IQR), y 2.0 (0.8-5.0) for Treatment as Usual and 2.0 (0.7-5.0) for Housing First ; ^b^ MCAS: Multnomah Community Ability Scale. Possible scores range from 17 to 85, with lower scores indicating more disability.;

^c^ MINI: The Mini-International Neuropsychiatric Interview
