# Supplementary material for: Women experiencing homelessness and mental illness in a Housing First multi-site trial: Looking beyond housing to social outcomes and well-being
Source: PLoS One. 2023 Feb 10;18(2):e0277074. doi: 10.1371/journal.pone.0277074 (PMC9916643; doi:10.1371/journal.pone.0277074)
Supplement: Attachment — Submitted filename: Response to Reviewer PONE-D-21-13748.docx [file pone.0277074.s004.docx]

|  | Reviewer 4 comment | Author response |
| --- | --- | --- |
|  | A summary of the points Reviewer 4 raised in the May 11, 2022 email includes:  - Some missing details should be provided for some aspects of the statistical analysis.  - The manuscript should clearly state the exploratory nature of the study.  - Data should be made available, barring ethical considerations (in which case a clear statement should be added in the manuscript).  - More appropriate analyses should be used for the time series data, or statistical significance testing should be dropped altogether. | All of these points along with the detailed feedback from Reviewer 4 contained in comment boxes in the PDF are addressed in detail below. |
| 1 | The authors recognise the exploratory nature of the study in the comments to the editor, but the word "exploratory" or "hypothesis-generating" never appear in the manuscript, nor in the title or abstract.  Please, clearly state at least in the manuscript and abstract that this is an exploratory study. The best practice would be to also include it in the title. | We have added the word “exploratory” to qualify the type of study in the Abstract at the body of the manuscript in the Methods. |
| 2 | A Data Availability Statement describing where the data can be found is required at submission. Your answers to this question constitute the Data Availability Statement and will be published in the article, if accepted.  Important: Stating ‘data available on request from the author’ is not sufficient. If your data are only available upon request, select ‘No’ for the first question and explain your exceptional situation in the text box.  Do the authors confirm that all data underlying the findings described in their manuscript are fully available without restriction? | We have changed our answer to “no” the data are not available in the publication. As the data used in this paper from four of the five sites were made available for use by our team via a data sharing agreement for analysis purposes only and not for making the data publicaly available. As such, data for all five sites is available from a third party. Researchers may contact the Observational Epidemiology & Qualitative Research unit at the Health Research Solutions HUB at Unity Health Toronto (<https://www.hubresearch.ca/services/observational-epidemiology-qualitative-research/>) where requests for the data can be submitted. |
| 3 | First, as the largest randomized trial of a Housing First initiative and includes an unprecedented sample of several hundred women. Runaway verb | We have rewritten this sentence. |
| 4 | I am not familiar with this measure. Is the measure intended to be the sum of the score of each domain? Or is this something that the authors do? Please, just briefly specify this for clarity to those who are not familiar with the measure. | We have edited the description of the scale to clarify. This scale is widely used in homeless research and we provide the reference for the original paper and also our JAMA paper using this scale which provides additional details.  The Lehman Quality of Life Interview 20 (QoLI-20) measures participants’ condition-specific quality of life. Twenty items are scored on a 7-point Likert scale which encompass the domains of family, finance, leisure, living, safety, social, and overall quality of life. In this work, we use the total score defined as the sum of all 20 items, which ranges from 20 to 140, with higher scores indicating better quality of life [49, 58]. |
| 5 | Correct this sentence and remove one of the “includes”  “Baseline and predictor variables included in analyses included data collected on traumatic events that occurred before the age of 18 years using the Adverse Childhood Experiences module  assessing ten early childhood adversities (yes/no items)” | This problem has been corrected. |
| 6 | This is very hard to follow because there are many outcome variables. Please, clearly state which variables were treated as what (continuous, count, proportion etc) and specify for each which distribution family was used in the models. A bullet point list would be great and/or maybe a table. (I had to scroll back and forth from this paragraph to the previous sections to understand what was going on) (page 11, statistical analyses section) | We refer the reviewer to the **Outcomes and other study variables** section of the paper where outcomes are defined according to the type of variable.  The reviewer can also find edits further clarifying:  1-percentage of days stably housed varied from 0-100%.Since it is the ratio of days housed by days accounted, it is naturally a continuous variable  2-QOLI-20 is the sum of 20 items, and varies from 20 to 140. Such scores are commonly analyzed as continuous. See ref 49 and 58  3-MCAS is the sum of 17 items and varies between 17-85 . Such scores are commonly analyzed as continuous . See ref 49  4-Likewise for the CSI  5-ED is defined as count  6-The GAIN is explicitly defined as a count variable now  7-Physical community integration is clearly defined as a count, and psychological community integration ranges from 4 and 20, usually analyzed as a continuous variable [49] |
| 7 | (page 11 statistical analyses) The outcome "days spent stably housed" is continuous but bounded (between 0 and 1) so a Gaussian family is not appropriate. I suggest either to clearly state and stress that the results are to be taken as not robust or to fit a model using the beta distribution, which is appropriate for proportions/percentages. | As stated above, the percentage of days stably housed varies between 0 and 100%. While we appreciate the reviewer’s suggestion to use the beta distribution, this approach has its own limitations since the beta distribution is defined for values greater than 0 and lower than 1 (assuming we would work with proportions). One of the approaches to circumvent this problem is to add a small amount, say 0.005, to zero and subtract a small amount from 1. However, in our case, 0's and 1's are structural, and we did not feel comfortable with this approach. Even in the case where we might fit such a model, we could not guarantee that it will fit the data well.  Therefore, we used the linear model which has other limitations but is easier to interpret and communicate results. Given the exploratory objective with our study, we felt comfortable with this decision. |
| 8 | (page 13 reference 69) I was not familiar with this paper. I strongly believe that, given the exploratory nature of this study, it is of outmost importance that multiple testing is taken into consideration. In fact, I read the paper cited here and I was appalled by what I read.  The definition of p-values given in the paper is just utterly wrong, and the rest of the paper provides very general recommendations.  I disagree with the authors here in having chosen not to correct for multiple testing. Testing was done with 7 different outcome variables at 3 different time point pairs. Meaning that 21 comparisons where done. At least one comparison is bound to be significant, just because of the alpha level = 0.05. Correction MUST be applied, by at least dividing alpha by 21, alpha_corrected = 0.0023. | We do appreciate the reviewer's opinion, but also would like to point out that there is no consensus on the issue of adjusting for multiple testing in the literature. While some experts provide compelling reasons for not adjusting [Althouse, 2016; Parker & Weir 2020], others provide a rationale for both adjusting and not adjusting [Howard, 2018; Cook and Farewell, 1996]  Following the rationale provided in Althouse's points (1), (2) and (3), we decided to not adjust for multiple testing because ours is an exploratory substudy from a larger RCT. Following his recommendation we "(1) described what was done in a study; (2) reported effect sizes, confidence intervals, and p values; and (3) allow readers use their own judgment about the relative weight of the conclusions.”  We have edited our statistical methods section to refer to his paper as the reference for our decision to opt out of multiple testing adjustment.  A D Althouse, Adjust for Multiple Comparisons? It's Not That Simple. Ann Thorac Surg. 2016 May;101(5):1644-5  Parker RA, Weir CJ. Non-adjustment for multiple testing in multi-arm trials of distinct treatments: Rationale and justification. Clin Trials. 2020  Oct;17(5):562-566. doi: 10.1177/1740774520941419. Epub 2020 Jul 15. PMID:  32666813; PMCID: PMC7534018.  Cook RJ, Farewell VT Multiplicity Consideration in the Design of Clinical Trials. J R Statist Soc A, 1996 159(Part 1):93-110  Howard DR, Brown JM, Todd S, Gregory WM. Recommendations on multiple testing  adjustment in multi-arm trials with a shared control group. Stat Methods Med  Res. 2018 May;27(5):1513-1530. doi: 10.1177/0962280216664759. Epub 2016 Sep 19.  PMID: 27647808. |
| 9 | In any case, as far as I understand, t-tests were performed to test for differences between each follow-up time point. This method is not appropriate, among other things because each time-point is correlated with the next, this being a time series. This means that less inter-time-point variance is to be expected, which increases Type I error rates due to increased autocorrelation.  A better approach is to used techniques designed to work with time series data, such as generalised additive models.  If the authors don't have the skills or resources to apply such methods, I suggest them not to do significance testing at all and just report and discuss the patterns that can be observed in the plots (which is basically what is done in the paper already, so they would just have to drop the significance testing parts). | We completely agree with the reviewer and therefore we never conducted t-tests. Rather, for longitudinal outcomes, we did use methods to accommodate correlation between repeated measures. In the methods sections we state  "Longitudinal outcomes were analyzed using linear mixed analysis of repeated measures models for continuous outcomes and generalized estimating equations for count outcomes (Poisson distribution for CIS-Physical Integration and negative binomial distribution for GAIN-SS and ED Visits), assuming an unstructured correlation matrix for the repeated measures."  With respect to the reviewer's opinion about using GAM, we also note our models are valid given the design of our study and the nature of our outcomes. We chose to use these longitudinal models that are easier to interpret and communicate results.  Regarding the last comment in this set, we feel it is unnecessary and disrespectful to disparage the skills or resources of our authorship team. We are open to receiving respectful feedback and opportunities to respond to perceived limitations of our approach. Raising questions about our skills falls outside of respectful scientific dialogue. |
| 10 | Nothing is said about where one can find the data and the analysis scripts/instructions. Please specify where these can be found. | We have answered this issue above. |
| 11 | There are two “and” in this sentence on page 14. Table 2 displays results from models examining changes from baseline to 24 months for  those in HF and TAU arms for each of our social, well-being and, and comparing groups with  respect to housing stability over the 24-month period. | We have corrected this problem. |
| 12 | Please, don't use bold words like "demonstrated". Change to "suggested/indicated/ect/" | We have replaced ‘demonstrated’ with suggested as requested. |
| 13 | (page 14) significant based on what? the comparisons between each follow-up point? Please specify | As each study used a different approach we’ve omitted the word significant and replaced it with a description of the overall direction of the findings across the different studies. |
