# Supplementary material for: Women experiencing homelessness and mental illness in a Housing First multi-site trial: Looking beyond housing to social outcomes and well-being
Source: PLoS One. 2023 Feb 10;18(2):e0277074. doi: 10.1371/journal.pone.0277074 (PMC9916643; doi:10.1371/journal.pone.0277074)
Supplement: Attachment — Submitted filename: Response to Reviewer PONE-D-21-13748.docx [file pone.0277074.s006.docx]

| Reviewer comment | Author response. |
| --- | --- |
| (regarding “days spent stably housed)  If there are 0s and 1s that are structurally important, then a zero-one inflated beta regression (ZOIB) is the most appropriate choice. | We are surprised by how much effort this reviewer places on raising questions about our statistical modeling of the outcome related to housing stability as we have provided a sound rationale for our choice of our statistical model and the findings are so strong that the choice of model is not likely to change our conclusions. Nevertheless, to address the reviewer's concern, we fit an additional model, the fractional logistic model ^(1)^ for the proportion of days stably housed over 24 months instead of fitting the ZOIB model. The model recommended by the reviewer, the ZOIB, is not appropriate as it requires the assumption that 0's and 1's represent very distinct processes from the process which generated the continuous values, which we do not believe they do.  We are able to illustrate, that, for our data, the linear model and the fractional logistic model yield exactly the same conclusions.  The fractional logistic model ^(1)^ uses a quasi-likelihood function for estimation and is appropriate for proportion outcomes when the values of 0 and 1 are admissible and observable. The fractional logistic model predicts the mean of the proportion conditional on covariates. To ensure that the predicted mean is also between 0 and 1, the logit model for the mean is considered.  The parameters estimated from this model can be exponentiated to yield odds ratios. We now include both analyses in our paper. See the revisions in the Methods section and Tables of findings. |
| (regarding the issue of multiple testing)  I am not sure then what is the use of p-values if they are clearly affected by multiple testing and if they are not relevant. Perhaps it is worth dropping them altogether. Up to the authors to decide what to do. | We do appreciate the reviewer's comments, but we do stand by our position, that there is no consensus in the literature on the issue of multiple testing for exploratory studies, as indicated in the references we provided in our previous responses. For this reason, we believe that it is not a matter of who is right or wrong, but rather, which view one is more comfortable endorsing. Furthermore, we do have to focus on the intention of the paper that is to explore the wealth of data for homeless women with mental illness (rather than to test very specific hypotheses) and to generate hypothesis for future studies to test or use in their evaluation of interventions. Therefore, as permitted by this reviewer, we decided to keep the p-values, as well as confidence intervals, in our paper. We appreciate that the reviewer leaves the final decision on this point up to us. |
| However, if it is the case that linear models where used to analyse the longitudinal data, then I am afraid I cannot reconstruct what was done based on the highlighted sentence.* It is necessary to include a full account of how the models were constructed, in details. If the authors do have the resources to run GAMs on the longitudinal data (as I gather from their response) then they should proceed with it.  **highlighted sentence: "Longitudinal outcomes were analyzed using linear mixed analysis of repeated measures models for continuous outcomes and generalized estimating equations for count outcomes (Poisson distribution for CIS-Physical Integration and negative binomial distribution for GAIN- SS and ED Visits), assuming an unstructured correlation matrix for the repeated measures."* | We do not believe GAMs are necessary in our case since our models include a categorical time variable and we are not interested in deriving complex trajectories.  Regarding the specification of the models, we have provided as much detail that is typically provided in journal articles. Space limitations preclude us describing the specifics of each and every model we fit. Thus, to help the reviewer and readers completely understand what was done, we now include the SAS syntax in the Supplemental information, S3. We do provide the code for each objective and each model presented in the results Tables so we hope this will make clear the details about each model. |
| I still cannot find in the manuscript any mention to where the analysis scripts/software instructions to reproduce the analysis can be found. (I understand the data are not available, but that doesn't prevent one to share analysis scripts/instructions) | The SAS syntax for objectives and outcomes is now included in Supplemental Table 3. |

1.	Papke LE, Wooldridge JM. Econometric Methods for Fractional Response Variables With an Application to 401 (K) Plan Participation Rates. Journal of Applied Econometrics. 1996;11(6):619-32.
