# Supplementary material for: Women experiencing homelessness and mental illness in a Housing First multi-site trial: Looking beyond housing to social outcomes and well-being
Source: PLoS One. 2023 Feb 10;18(2):e0277074. doi: 10.1371/journal.pone.0277074 (PMC9916643; doi:10.1371/journal.pone.0277074)
Supplement: Peer review history

# Peer review: Decision Letter 0

**DOI:** 10.1371/journal.pone.0277074.r001

**Authors:** Janet E Rosenbaum

9 Dec 2021

PONE-D-21-13748Women experiencing homelessness and mental illness in a Housing First multi-site trial. Looking beyond housing to social outcomes and well-being.PLOS ONE

Dear Dr. O'Campo,

Thank you for submitting your manuscript to PLOS ONE. After careful consideration, we feel that it has merit but does not fully meet PLOS ONE’s publication criteria as it currently stands. Therefore, we invite you to submit a revised version of the manuscript that addresses the points raised during the review process.

Regarding missingness, it’s important to address the missing data mechanisms in more depth than currently, and also whether these mechanisms result in data that are missing at random, missing completely at random, or even not missing at random. Given the diverse reasons for missingness, it is possible that more than one type of missingness is present in the data.  

70% of data had at least one missing value. With such a large percent like 70%, a reader might think that is in the entire dataset. It seems from context and supplementary table 1 that this was all variables that are used in this paper’s analysis, so please say that more clearly.  Also given that 70% of data used in the study had at least one missing value, please cite research about the implications of MI in data with such a large percent missing.  

One reviewer asked for sensitivity analysis for imputed versus non-imputed. Diagnostics of MI method may be appropriate.  

With regard to sample size calculation, an alternative to a standard power calculation would be Gelman and Carlin type-M and type-S errors, which is less centered on p-values.  

The effects of the experiment have been displayed as p-values.  An effect size measure such as Hedge’s g or Cohen’s d in addition to the differences that are in the tables 2-4. These effect size measures may give a clearer picture of the true results of the experiment.

P-values have been displayed to 3 digits in all tables. That’s too many digits and gives false precision.  Just one significant digit is fine. For instance, in table 2 the p-values would be 0.001 (as is currently there), 0.5, 0.2, 0.2, 0.9, 0.4, 0.7, 0.9.

Figure 1 is really nice display of results, but the treated and control groups should be shifted slightly so the confidence intervals are clearer and don’t overlap— e.g., intervention at 0.5 months below each time point and control at 0.5 months above each time point. Many of these continuous variable outcomes in figure 1 probably have complex distributions, and it may be helpful to show the distributions of these variables for treatment versus control, such as using a violin plot or similar data display, rather than the point and CI shown in figure 1.

The tables are somewhat hard to read with many lines, and they may benefit from using data displays such as dumbbell plots for comparisons of the treatment vs control.

The mixed models should report evaluation of assumptions of the models, such as linear regression, Poisson regression.

Table 2 shows percent of the time housed over all visits. However, the other measures in table 2 are a comparison of baseline to 24 months adjusted for city and need level, which leaves out the middle 3 follow-up visits.  It seems like these observations in table 2 could be more completely displayed as an adjusted version of Figure 1 that includes all time points adjusted for city and need level.  

Table 4 shows mixed model effects that leaves out the treatment effect and treatment x time, among other variables. It seems that this table should at least include treatment effect coefficients.  Omitting the main effect of treatment doesn’t avoid table 2 fallacy. The authors should address whether table 2 fallacy may apply here. 

Please submit your revised manuscript by Jan 23 2022 11:59PM. If you will need more time than this to complete your revisions, please reply to this message or contact the journal office at plosone@plos.org. When you're ready to submit your revision, log on to https://www.editorialmanager.com/pone/ and select the 'Submissions Needing Revision' folder to locate your manuscript file.

Please include the following items when submitting your revised manuscript:

- A rebuttal letter that responds to each point raised by the academic editor and reviewer(s). You should upload this letter as a separate file labeled 'Response to Reviewers'.
- A marked-up copy of your manuscript that highlights changes made to the original version. You should upload this as a separate file labeled 'Revised Manuscript with Track Changes'.
- An unmarked version of your revised paper without tracked changes. You should upload this as a separate file labeled 'Manuscript'.

If you would like to make changes to your financial disclosure, please include your updated statement in your cover letter. Guidelines for resubmitting your figure files are available below the reviewer comments at the end of this letter.

If applicable, we recommend that you deposit your laboratory protocols in protocols.io to enhance the reproducibility of your results. Protocols.io assigns your protocol its own identifier (DOI) so that it can be cited independently in the future. For instructions see: https://journals.plos.org/plosone/s/submission-guidelines#loc-laboratory-protocols. Additionally, PLOS ONE offers an option for publishing peer-reviewed Lab Protocol articles, which describe protocols hosted on protocols.io. Read more information on sharing protocols at https://plos.org/protocols?utm_medium=editorial-email&utm_source=authorletters&utm_campaign=protocols.

We look forward to receiving your revised manuscript.

Kind regards,

Janet E Rosenbaum, Ph.D.

Academic Editor

PLOS ONE

Journal Requirements:

1. When submitting your revision, we need you to address these additional requirements.

Please ensure that your manuscript meets PLOS ONE's style requirements, including those for file naming. The PLOS ONE style templates can be found at 

https://journals.plos.org/plosone/s/file?id=wjVg/PLOSOne_formatting_sample_main_body.pdf and 

https://journals.plos.org/plosone/s/file?id=ba62/PLOSOne_formatting_sample_title_authors_affiliations.pdf

2. Please ensure that you include a title page within your main document. We do appreciate that you have a title page document uploaded as a separate file, however, as per our author guidelines (http://journals.plos.org/plosone/s/submission-guidelines#loc-title-page) we do require this to be part of the manuscript file itself and not uploaded separately.

Could you therefore please include the title page into the beginning of your manuscript file itself, listing all authors and affiliations.

3. Please provide additional details regarding participant consent. In the ethics statement in the Methods and online submission information, please ensure that you have specified what type you obtained (for instance, written or verbal, and if verbal, how it was documented and witnessed). If your study included minors, state whether you obtained consent from parents or guardians. If the need for consent was waived by the ethics committee, please include this information.

4. We note that the grant information you provided in the ‘Funding Information’ and ‘Financial Disclosure’ sections do not match. 

When you resubmit, please ensure that you provide the correct grant numbers for the awards you received for your study in the ‘Funding Information’ section.

5. Please include your tables as part of your main manuscript and remove the individual files. Please note that supplementary tables (should remain/ be uploaded) as separate "supporting information" files.

6. Please include captions for your Supporting Information files at the end of your manuscript, and update any in-text citations to match accordingly. Please see our Supporting Information guidelines for more information: http://journals.plos.org/plosone/s/supporting-information. 

7. Please review your reference list to ensure that it is complete and correct. If you have cited papers that have been retracted, please include the rationale for doing so in the manuscript text, or remove these references and replace them with relevant current references. Any changes to the reference list should be mentioned in the rebuttal letter that accompanies your revised manuscript. If you need to cite a retracted article, indicate the article’s retracted status in the References list and also include a citation and full reference for the retraction notice.

[Note: HTML markup is below. Please do not edit.]

Reviewers' comments:

Reviewer's Responses to Questions

**Comments to the Author**

1. Is the manuscript technically sound, and do the data support the conclusions?

The manuscript must describe a technically sound piece of scientific research with data that supports the conclusions. Experiments must have been conducted rigorously, with appropriate controls, replication, and sample sizes. The conclusions must be drawn appropriately based on the data presented. 

Reviewer #1: Yes

Reviewer #2: Yes

Reviewer #3: Yes

**********

2. Has the statistical analysis been performed appropriately and rigorously? 

Reviewer #1: I Don't Know

Reviewer #2: Yes

Reviewer #3: Yes

**********

3. Have the authors made all data underlying the findings in their manuscript fully available?

The PLOS Data policy requires authors to make all data underlying the findings described in their manuscript fully available without restriction, with rare exception (please refer to the Data Availability Statement in the manuscript PDF file). The data should be provided as part of the manuscript or its supporting information, or deposited to a public repository. For example, in addition to summary statistics, the data points behind means, medians and variance measures should be available. If there are restrictions on publicly sharing data—e.g. participant privacy or use of data from a third party—those must be specified.

Reviewer #1: No

Reviewer #2: Yes

Reviewer #3: Yes

**********

4. Is the manuscript presented in an intelligible fashion and written in standard English?

PLOS ONE does not copyedit accepted manuscripts, so the language in submitted articles must be clear, correct, and unambiguous. Any typographical or grammatical errors should be corrected at revision, so please note any specific errors here.

Reviewer #1: Yes

Reviewer #2: Yes

Reviewer #3: Yes

**********

5. Review Comments to the Author

Please use the space provided to explain your answers to the questions above. You may also include additional comments for the author, including concerns about dual publication, research ethics, or publication ethics. (Please upload your review as an attachment if it exceeds 20,000 characters)

Reviewer #1: Overall, the study sheds important light on the effectiveness of Housing First intervention among women with mental illness. However, there were a few areas that could be strengthened. In the introduction section, beyond the scant literature on women in permanent supportive housing programs, the authors should consider strengthening the significance of the study. A clearly stated rationale is warranted for the study.

The methods section includes instruments that have strong psychometric properties in the literature. However, the authors do not report any reliability coefficient of the instruments. Further, no testing of group differences on demographic characteristics were reported. The reasons for selecting predictor and control variables are unclear.

Reviewer #2: This manuscript reports a secondary analysis of the original Canadian five city Housing First trial focused solely on female participants. The authors largely repeat the analyses previously reported, but with a specific focus on women; they also add an examination of specific risk factors that might be associated with differential outcomes for women.

Because of the size and innovation of the original study, the secondary analysis is still able to provide some new insights and considerations in an important subsample. As the authors note, many investigators have argued for separate studies or at least distinguishing analyses to understand the numerous differences reported among men and women experiencing homelessness. Moreover, the homelessness epidemic has only worsened in the context of the COVID pandemic so the issues remain relevant if these new analyses can provide insights.

Several strengths are evident besides the original sample size and randomization. First, the authors do include novel risk/resilience factors that might alter the outcomes for women experiencing homelessness such as the caretaking status of dependent children, exposure to ACES, and others. These factors have not been sufficiently considered in earlier studies of Housing First. Secondly, the authors examined group by time effects and group differences at each interval to assess the relative changes from the intervention. Thirdly, they provide detailed reporting on the individual outcomes and the relative importance or size of the changes.

Some changes would also strengthen the manuscript. The most important is better clarity and continuity among the introduction, the results and the discussion. Para 3 of the intro makes a strong argument for providing data for women experiencing homelessness. However, para 5 points to studies that tried and found no differences. Why will this study be different than those studies?

Similarly, in the results, the authors found intervention women did NOT differ on most assessments with the exception of stably housed days (the primary intervention). However, this is different than the original finding of the five city study where all participants (men and women together) had improved quality of life and integration. No comment or discussion on this ‘lessening’ of effects for women is provided.

This theme continues into the discussion where the authors conclude with the need for more studies of women even though they find no clear benefit from separate analysis in this example nor in the cited studies in their introduction. Reconciling their results, introduction and discussion seem like a critical piece because it is the main contribution of the study rather than some of the ancillary factors they look at.

Tables 3 and 4 are difficult to read given the multitude of comparisons that are made.

Reviewer #3: Thank you for the opportunity to review this important work. This study examined the social, health, and housing needs of women experiencing homelessness. It will make an important contribution to the evidence base as the experiences of this population have received little attention. The strengths of this study include a large sample of women experiencing homelessness in the context of a multi-site randomized controlled Housing First trial, a women-only analysis, and baseline factors and outcomes that have not been fully explored in past research. However, there are some comments that need to be addressed, including questions about the statistical analysis, and adding to the discussion section.

Is the manuscript technically sound? And do the data support the conclusions?

Yes, the manuscript is technically sound, and the methods and analysis are clearly described to allow for replication. Data are also consistent with discussion section and conclusions. There are a few items that needs to be addressed:

- Please describe baseline predictors and how they were coded in the methods section

- For women with children in the sample, were any living with their children? What type of support was provided to children? Financial? Social?

- Study findings are very interesting and surprising, particularly the lack of significant findings associated with health and social outcomes. Can prior research provide an explanation for the decline in ED visits for the TAU group? Were there any demographic characteristics that could have influenced the findings (marital status, racial/ethnic group, sources of income, having children, mental health diagnosis, comorbid conditions)?

- The first half of the discussion section is a bit thin and reads more like a results section than a discussion section. Consider cutting down sentences that restate the results and incorporate more explanation of findings using past research.

- This sentence is confusing: “Contrary to what we might have anticipated on the importance of ACEs on multiple study outcomes based upon prior demonstrating the association of ACEs with victimization and criminal justice involvement, in our study high baseline ACE scores only predicted significantly worse scores on mental illness severity (CSI).” (Line 298-300)

Has the statistical analysis been performed appropriately and rigorously?

Yes, the statistical analysis has been performed appropriately and rigorously. Please address the following comments:

- Please add statistical results for linear mixed models to the abstract

- Consider running a sensitivity analysis comparing imputed vs. non-imputed data

- Why were no adjustments made to account for multiple tests?

- Please include sample size calculations

Have the authors made all data underlying the findings in their manuscript fully available?

Yes, all data used in the manuscript has been made fully available.

Is the manuscript presented in an intelligible fashion and written in standard English?

Yes, the manuscript is clear and well-written.

**********

6. PLOS authors have the option to publish the peer review history of their article (what does this mean?). If published, this will include your full peer review and any attached files.

If you choose “no”, your identity will remain anonymous but your review may still be made public.

**Do you want your identity to be public for this peer review?** For information about this choice, including consent withdrawal, please see our Privacy Policy.

Reviewer #1: No

Reviewer #2: **Yes: **Kelly J. Kelleher, MD, MPH

Reviewer #3: **Yes: **Dr. Alexia Polillo

[NOTE: If reviewer comments were submitted as an attachment file, they will be attached to this email and accessible via the submission site. Please log into your account, locate the manuscript record, and check for the action link "View Attachments". If this link does not appear, there are no attachment files.]

While revising your submission, please upload your figure files to the Preflight Analysis and Conversion Engine (PACE) digital diagnostic tool, https://pacev2.apexcovantage.com/. PACE helps ensure that figures meet PLOS requirements. To use PACE, you must first register as a user. Registration is free. Then, login and navigate to the UPLOAD tab, where you will find detailed instructions on how to use the tool. If you encounter any issues or have any questions when using PACE, please email PLOS at figures@plos.org. Please note that Supporting Information files do not need this step.

# Author comment: Author response to Decision Letter 0

**DOI:** 10.1371/journal.pone.0277074.r002

8 Mar 2022

Here we respond to the specific comments raised by the Editor. These are also included in our “Response to Reviewer” document along with supplemental figures/tables. 

Comment: Regarding missingness, it’s important to address the missing data mechanisms in more depth than currently, and also whether these mechanisms result in data that are missing at random, missing completely at random, or even not missing at random. Given the diverse reasons for missingness, it is possible that more than one type of missingness is present in the data. 

Answer: Missing data mechanisms have been widely presented and discussed in the literature. Although the Missing Completely at Random mechanism simplifies analyses, it is quite difficult to find real life applications that satisfy its assumption [VanBuuren2012], and therefore we did not consider it in our data. 

We did however consider the Missing at Random (MAR) Mechanism, where the probability of missingness depends only on observed data but not on the data that are missing. That is, after controlling for or stratifying by observed variables, missingness is random. We followed recommendation from Van Buuren 2012 and Barnard 1999 to include in our imputation models a high the number of predictors of the incomplete variables in order to make the MAR assumptions more plausible.

Van Buuren, S. (2012), Flexible Imputation of Missing Data. Chapman & Hall/CRC, Boca Raton, FL.

Barnard J, Meng XL Applications of multiple imputation in medical studies: from AIDS to NHANES. Statistical Methods in Medical Research. 8:17-36, 1999

Comment: 70% of data had at least one missing value. With such a large percent like 70%, a reader might think that is in the entire dataset. It seems from context and supplementary table 1 that this was all variables that are used in this paper’s analysis, so please say that more clearly. Also given that 70% of data used in the study had at least one missing value, please cite research about the implications of MI in data with such a large percent missing. 

Answer: We followed recommendations from White et al, 2010 and Horton et al. 2007 who suggest that depending on the number and type of variables, and amount of missing data, 50 to 100 imputations yield better estimates. However, a rule of thumb was provided by White et al. 2010 that the number of imputations should be at least 100 x (percent of incomplete cases) and we applied this recommendation to our data analysis approach.

Horton, N. & Kleinman, K.P. (2007). Much ado about nothing: A comparison of missing data methods and software to fit incomplete data regression models. The American Statistician 61(1): 79-90. Software appendix: http://www.math.smith.edu/muchado-appendix.pdf

White, I.R., Royston, P., and Wood, A.M. (2010). Multiple imputation using chained equations: Issues and guidance for practice. Statistics in Medicine 30:377-399. (Includes Stata codes) 

Comment: One reviewer asked for sensitivity analysis for imputed versus non-imputed. Diagnostics of MI method may be appropriate. 

Answer: As requested, we provide a sample of multiple imputation diagnostic plots as suggested by Van Buuren, 2012 at the end of this document. 

We also added the following sentence to the methods: Comparisons between observed and imputed data via diagnostic plots were used to assess the quality and plausibility of the imputations. 

Van Buuren, S. (2012), Flexible Imputation of Missing Data. Chapman & Hall/CRC, Boca Raton, FL.

Comment: With regard to sample size calculation, an alternative to a standard power calculation would be Gelman and Carlin type-M and type-S errors, which is less centered on p-values. 

Answer: Because ours was a post-hoc subgroup exploratory analysis of the main study, the sample size calculation which was predetermined for the main hypotheses for the main study involving the full sample, does not apply here. As such, for the analyses we present here, we would like to emphasize that our study was hypothesis generating and therefore power calculations do not apply. The original sample sizes were calculated for the original study, please refer to protocol in Goering et al.

Goering PN, Streiner DL, Adair C, Aubry T, Barker J, Distasio J, Hwang SW, Komaroff J, Latimer E, Somers J, Zabkiewicz DM. The At Home/Chez Soi trial protocol: a pragmatic, multi-site, randomised controlled trial of a Housing First intervention for homeless individuals with mental illness in five Canadian cities. BMJ Open. 2011 Nov 14;1(2):e000323

Comment: The effects of the experiment have been displayed as p-values. An effect size measure such as Hedge’s g or Cohen’s d in addition to the differences that are in the tables 2-4. These effect size measures may give a clearer picture of the true results of the experiment. 

Answer: We are familiar with Cohen’s d and the Hedge’s g correction as alternate ways of illustrating differences between treatment and control groups. We did not use Cohen’s d in any of our dozens of publications that emerged from this randomized trial as in our experience it is not as common in health or medical studies as it would be in psychology. In fact, a quick review of evaluations or randomized trials published in Plos One over a one-year period (back to Dec 2020, see citations below) demonstrated that these studies report findings in a similar way to which we presented our findings. Evaluations presented mean values of outcomes for treatment and controls groups along with 95% confidence intervals and p-values. Some studies add in the t-statistics. As such, we feel that adding Cohen’s d values were not necessary. 

Hajek P, Przulj D, Pesola F, McRobbie H, Peerbux S, Phillips-Waller A, et al. (2021) A randomised controlled trial of the 5:2 diet. PLoS ONE 16(11): e0258853. 

Ngugi P, Babic A, Were MC (2021) A multivariate statistical evaluation of actual use of electronic health record systems implementations in Kenya. PLoS ONE 16(9): e0256799. 

Müller SA, Diallo AOK, Rocha C, Wood R, Landsmann L, Camara BS, et al. (2021) Mixed methods study evaluating the implementation of the WHO hand hygiene strategy focusing on alcohol based handrub and training among health care workers in Faranah, Guinea. PLoS ONE 16(8): e0256760. 

Andersen TO, Dissing AS, Varga TV, Rod NH (2021) The SmartSleep Experiment: Evaluation of changes in night-time smartphone behavior following a mass media citizen science campaign. PLoS ONE 16(7): e0253783. 

Greene MC, Likindikoki S, Rees S, Bonz A, Kaysen D, Misinzo L, et al. (2021) Evaluation of an integrated intervention to reduce psychological distress and intimate partner violence in refugees: Results from the Nguvu cluster randomized feasibility trial. PLoS ONE 16(6): e0252982. 

Heimgartner N, Meier S, Grolimund S, Ponti S, Arpagaus S, Kappeler F, et al. (2021) Randomized controlled evaluation of the psychophysiological effects of social support stress management in healthy women. PLoS ONE 16(6): e0252568. 

Dardas A, Williams A, Wang L (2021) Evaluating changes in workplace culture: Effectiveness of a caregiver-friendly workplace program in a public post-secondary educational institution. PLoS ONE 16(5): e0250978. 

O’Donnell A, Wilson L, Bosch JA, Borrows R (2020) Life satisfaction and happiness in patients shielding from the COVID-19 global pandemic: A randomised controlled study of the ‘mood as information’ theory. PLoS ONE 15(12): e0243278. 

Comment: P-values have been displayed to 3 digits in all tables. That’s too many digits and gives false precision. Just one significant digit is fine. For instance, in table 2 the p-values would be 0.001 (as is currently there), 0.5, 0.2, 0.2, 0.9, 0.4, 0.7, 0.9. 

Answer:We respectfully disagree with this suggestion to report p-values using 1 significant digit. We not only feel this is imprecise but this practice is not aligned with what we discovered in our review of studies published in Plos One. Studies reviewed (see item above) reported p-values using 3 digits or sometimes more significant digits. Therefore, we have not reduced the number of significant digits we report for our p-values in our tables and text. 

Comment: Figure 1 is really nice display of results, but the treated and control groups should be shifted slightly so the confidence intervals are clearer and don’t overlap— e.g., intervention at 0.5 months below each time point and control at 0.5 months above each time point. Many of these continuous variable outcomes in figure 1 probably have complex distributions, and it may be helpful to show the distributions of these variables for treatment versus control, such as using a violin plot or similar data display, rather than the point and CI shown in figure 1. 

Answer:Thank you for your appreciation of our figures. The figures are intended to augment the study findings in the tables as Table 3 presents results of multivariate analyses. The Figures with the unadjusted results are intended to be a visual supplement to Table 3 as the differences or lack thereof are easier to grasp quickly in the figures. While CIs are included in the Figures to help identify whether the values at any one time point differ between treatment and TAU, the data in the Tables reporting multivariate regression findings should be the primary source of information about differences and significance of those differences. To change the scales on the figures to further separate the trend lines for the treatment and TAU would create larger figures that would take up too much space and is not necessary given that the multivariate findings are presented in Table 3. 

Comment: The tables are somewhat hard to read with many lines, and they may benefit from using data displays such as dumbbell plots for comparisons of the treatment vs control. 

Answer:We have added formats to the table to increase readability. In particular, we have shaded every other row in Tables 1, 3 and 4. We have added lines between each outcome to more clearly delineate the findings for each outcome and co-variate. We believe the format changes have considerably increased the readability of the Tables. Moreover the figures we provided as supplementary information about Table 3 is a visual aid to rapidly understanding the findings from the analysis of these trial data.

Comment: The mixed models should report evaluation of assumptions of the models, such as linear regression, Poisson regression. 

Answer:To respond to this suggestion we have added the following text in the Statistical analysis section: Normality of residuals was graphically checked for linear mixed models. The decision to fit a negative binomial rather than a Poisson model was based on whether over-dispersion (, ie, if the variance was much larger than the mean) was present in the count outcome data.

Comment: Table 2 shows percent of the time housed over all visits. However, the other measures in table 2 are a comparison of baseline to 24 months adjusted for city and need level, which leaves out the middle 3 follow-up visits. It seems like these observations in table 2 could be more completely displayed as an adjusted version of Figure 1 that includes all time points adjusted for city and need level. 

Answer:There were several reasons why our study measured housing success as cumulative stability at 24 months and we now state this in the methods. First, given the Housing First Theory of Change and Program Manual, stable housing is established within the first 1-3 months of enrolment for those in the treatment arm and housing status rarely changes after that point in time. Thus, we wanted to align with the program’s theory of change. Second, to measure housing status at different time points over the 24 months means that we would have seen very little change for those in the treatment arm and a single cumulative measure made more sense for this outcome. This measure captures overall stability and instability in that a small proportion of the treatment group was not stably housed throughout and this single measure captures that information. Third, this single measure captures the overall housing stability or instability across the two groups over a long period of time and for this outcome, knowing the housing status at different time points in the study is less relevant to the overall goals of the program. In sum, we chose the best measure for the outcome of housing even if it did not match how we approached other outcomes such as quality of life which is expected to demonstrate incremental changes over the 24-month follow-up period.

Comment: Table 4 shows mixed model effects that leaves out the treatment effect and treatment x time, among other variables. It seems that this table should at least include treatment effect coefficients. Omitting the main effect of treatment doesn’t avoid table 2 fallacy. The authors should address whether table 2 fallacy may apply here. 

Answer:Table 4 presents the findings for the third objective of the paper (see bottom of page 7 of the manuscript for the objective) intended to “explore baseline demographic, social, health measures, and ACEs as predictors of key study outcomes during the study period.” This objective is not concerned with differences between treatment and TAU. In fact, we include that objective because few differences between treatment and TAU were reported and we can, therefore, take advantage of this large sample of homeless women to learn more about predictors of our key outcomes. Consequently, our analysis for this objective does not account for treatment group membership by design. We do not address the issue of type 2 fallacy for this objective as it does not apply. However, we have made our intent clearer in the statistical analyses, see top of page 11 and in the results on page 13

Comment: 3. Please provide additional details regarding participant consent. In the ethics statement in the Methods and online submission information, please ensure that you have specified what type you obtained (for instance, written or verbal, and if verbal, how it was documented and witnessed). If your study included minors, state whether you obtained consent from parents or guardians. If the need for consent was waived by the ethics committee, please include this information. 

Answer:We have expanded the description of the consent process and Ethics Board approval process on page 8 of the revised manuscript. 

Comment: 4. We note that the grant information you provided in the ‘Funding Information’ and ‘Financial Disclosure’ sections do not match. 

Answer:We tried upon our initial submission to create a new entry online for our funding agency, the Mental Health Commission of Canada. However, when we try to add this new funding agency to your database the system freezes and cannot seem to complete the task. Therefore we did not do this task but are willing if we get assistance to do so. We did also try to email the generic help email but received an unhelpful response that we should enter the information into the field with no guidance on how to overcome our problem. 

Comment: When you resubmit, please ensure that you provide the correct grant numbers for the awards you received for your study in the ‘Funding Information’ section. 

Answer:There was no grant number for this award.

Attachment Submitted filename: Response to Reviewers PONE-D-21-13748.docx Click here for additional data file.

# Peer review: Decision Letter 1

**DOI:** 10.1371/journal.pone.0277074.r003

**Authors:** Janet E Rosenbaum

11 May 2022

PONE-D-21-13748R1

Women experiencing homelessness and mental illness in a Housing First multi-site trial: Looking beyond housing to social outcomes and well-being.

PLOS ONE

Dear Dr. O'Campo,

Thank you for submitting your manuscript to PLOS ONE. After careful consideration, we feel that it has merit but does not fully meet PLOS ONE’s publication criteria as it currently stands. Therefore, we invite you to submit a revised version of the manuscript that addresses the points raised during the review process.

We sent this manuscript for statistical review, and please see and address these comments.

Please submit your revised manuscript by Jun 10 2022 11:59PM. If you will need more time than this to complete your revisions, please reply to this message or contact the journal office at plosone@plos.org. When you're ready to submit your revision, log on to https://www.editorialmanager.com/pone/ and select the 'Submissions Needing Revision' folder to locate your manuscript file.

Please include the following items when submitting your revised manuscript:

- A rebuttal letter that responds to each point raised by the academic editor and reviewer(s). You should upload this letter as a separate file labeled 'Response to Reviewers'.
- A marked-up copy of your manuscript that highlights changes made to the original version. You should upload this as a separate file labeled 'Revised Manuscript with Track Changes'.
- An unmarked version of your revised paper without tracked changes. You should upload this as a separate file labeled 'Manuscript'.

If you would like to make changes to your financial disclosure, please include your updated statement in your cover letter. Guidelines for resubmitting your figure files are available below the reviewer comments at the end of this letter.

If applicable, we recommend that you deposit your laboratory protocols in protocols.io to enhance the reproducibility of your results. Protocols.io assigns your protocol its own identifier (DOI) so that it can be cited independently in the future. For instructions see: https://journals.plos.org/plosone/s/submission-guidelines#loc-laboratory-protocols. Additionally, PLOS ONE offers an option for publishing peer-reviewed Lab Protocol articles, which describe protocols hosted on protocols.io. Read more information on sharing protocols at https://plos.org/protocols?utm_medium=editorial-email&utm_source=authorletters&utm_campaign=protocols.

We look forward to receiving your revised manuscript.

Kind regards,

Janet E Rosenbaum, Ph.D.

Academic Editor

PLOS ONE

[Note: HTML markup is below. Please do not edit.]

Reviewers' comments:

Reviewer's Responses to Questions

**Comments to the Author**

1. If the authors have adequately addressed your comments raised in a previous round of review and you feel that this manuscript is now acceptable for publication, you may indicate that here to bypass the “Comments to the Author” section, enter your conflict of interest statement in the “Confidential to Editor” section, and submit your "Accept" recommendation.

Reviewer #2: All comments have been addressed

Reviewer #3: All comments have been addressed

Reviewer #4: (No Response)

********** 

2. Is the manuscript technically sound, and do the data support the conclusions?

The manuscript must describe a technically sound piece of scientific research with data that supports the conclusions. Experiments must have been conducted rigorously, with appropriate controls, replication, and sample sizes. The conclusions must be drawn appropriately based on the data presented. 

Reviewer #2: Yes

Reviewer #3: Yes

Reviewer #4: Partly

********** 

3. Has the statistical analysis been performed appropriately and rigorously? 

Reviewer #2: Yes

Reviewer #3: Yes

Reviewer #4: No

********** 

4. Have the authors made all data underlying the findings in their manuscript fully available?

The PLOS Data policy requires authors to make all data underlying the findings described in their manuscript fully available without restriction, with rare exception (please refer to the Data Availability Statement in the manuscript PDF file). The data should be provided as part of the manuscript or its supporting information, or deposited to a public repository. For example, in addition to summary statistics, the data points behind means, medians and variance measures should be available. If there are restrictions on publicly sharing data—e.g. participant privacy or use of data from a third party—those must be specified.

Reviewer #2: Yes

Reviewer #3: Yes

Reviewer #4: No

********** 

5. Is the manuscript presented in an intelligible fashion and written in standard English?

PLOS ONE does not copyedit accepted manuscripts, so the language in submitted articles must be clear, correct, and unambiguous. Any typographical or grammatical errors should be corrected at revision, so please note any specific errors here.

Reviewer #2: Yes

Reviewer #3: Yes

Reviewer #4: Yes

********** 

6. Review Comments to the Author

Please use the space provided to explain your answers to the questions above. You may also include additional comments for the author, including concerns about dual publication, research ethics, or publication ethics. (Please upload your review as an attachment if it exceeds 20,000 characters)

Reviewer #2: This revised manuscript is a sub-analysis of the Five City Housing First trial conducted in Canada focused on participants identifying as women. The original manuscript contained many strengths as the largest report of a trial focused on homeless women to date, an important set of outcome measures not previously considered in this population, and a thoughtful approach to the comparison.

The revision adds further strength. The authors have clarified their rationale and communication about their express purpose, the status of the extant literature, and how their findings are related to the original study analysis. In general, this reviewer finds the authors have done a very strong job of responding to the critiques of the reviewers and improved their manuscript in the process.

The only question raised by the changes is whether or not the additional tables combined with the figures is overwhelming to the reader or whether some of this material would be better placed as online supplements. The figures are particularly valuable, but the tables are still quite long.

Reviewer #3: (No Response)

Reviewer #4: Please see comments on attached PDF.

Please, make the original data available by clearly stating where that can be found. By data I mean here the raw data in tabular form with all demographics and scoring (not aggregated). If sensitive data cannot be shared, please state so and remove that data from the data table. If none of the data can be shared because consent to do so was not obtained from the participants, please clearly state so and select "no" in the upload form when asked if the data is available.

There are several points to be clarified/tackled in the manuscript, as reported in the comments on the attached PDF. In brief:

- Some missing details should be provided for some aspects of the statistical analysis.

- The manuscript should clearly state the exploratory nature of the study.

- Data should be made available, barring ethical considerations (in which case a clear statement should be added in the manuscript).

- More appropriate analyses should be used for the time series data, or statistical significance testing should be dropped altogether.

********** 

7. PLOS authors have the option to publish the peer review history of their article (what does this mean?). If published, this will include your full peer review and any attached files.

If you choose “no”, your identity will remain anonymous but your review may still be made public.

**Do you want your identity to be public for this peer review?** For information about this choice, including consent withdrawal, please see our Privacy Policy.

Reviewer #2: **Yes: **K Kelleher

Reviewer #3: **Yes: **Alexia Polillo

Reviewer #4: No

[NOTE: If reviewer comments were submitted as an attachment file, they will be attached to this email and accessible via the submission site. Please log into your account, locate the manuscript record, and check for the action link "View Attachments". If this link does not appear, there are no attachment files.]

While revising your submission, please upload your figure files to the Preflight Analysis and Conversion Engine (PACE) digital diagnostic tool, https://pacev2.apexcovantage.com/. PACE helps ensure that figures meet PLOS requirements. To use PACE, you must first register as a user. Registration is free. Then, login and navigate to the UPLOAD tab, where you will find detailed instructions on how to use the tool. If you encounter any issues or have any questions when using PACE, please email PLOS at figures@plos.org. Please note that Supporting Information files do not need this step.

Attachment Submitted filename: PONE-D-21-13748_R1_reviewer.pdf Click here for additional data file.

# Author comment: Author response to Decision Letter 1

**DOI:** 10.1371/journal.pone.0277074.r004

11 Jun 2022

A summary of the points Reviewer 4 raised in the May 11, 2022 email includes:

- Some missing details should be provided for some aspects of the statistical analysis.

- The manuscript should clearly state the exploratory nature of the study.

- Data should be made available, barring ethical considerations (in which case a clear statement should be added in the manuscript).

- More appropriate analyses should be used for the time series data, or statistical significance testing should be dropped altogether.

All of these points along with the additional detailed feedback from Reviewer 4 contained in comment boxes in the PDF are addressed in updated "Response to Reviewer" file provided in the attached files as instructed by the Editor. Please see that file for the specific responses and descriptions of revisions made to the manuscript.

Attachment Submitted filename: Response to Reviewer PONE-D-21-13748.docx Click here for additional data file.

# Peer review: Decision Letter 2

**DOI:** 10.1371/journal.pone.0277074.r005

**Authors:** Janet E Rosenbaum

6 Jul 2022

PONE-D-21-13748R2Women experiencing homelessness and mental illness in a Housing First multi-site trial: Looking beyond housing to social outcomes and well-being.PLOS ONE

Dear Dr. O'Campo,

Thank you for submitting your manuscript to PLOS ONE. After careful consideration, we feel that it has merit but does not fully meet PLOS ONE’s publication criteria as it currently stands. Therefore, we invite you to submit a revised version of the manuscript that addresses the points raised during the review process. Reviewer 2 had a small number of additional comments that they have addressed with respect to your responses.

Please submit your revised manuscript by Aug 20 2022 11:59PM. If you will need more time than this to complete your revisions, please reply to this message or contact the journal office at plosone@plos.org. When you're ready to submit your revision, log on to https://www.editorialmanager.com/pone/ and select the 'Submissions Needing Revision' folder to locate your manuscript file.

Please include the following items when submitting your revised manuscript:

- A rebuttal letter that responds to each point raised by the academic editor and reviewer(s). You should upload this letter as a separate file labeled 'Response to Reviewers'.
- A marked-up copy of your manuscript that highlights changes made to the original version. You should upload this as a separate file labeled 'Revised Manuscript with Track Changes'.
- An unmarked version of your revised paper without tracked changes. You should upload this as a separate file labeled 'Manuscript'.

If you would like to make changes to your financial disclosure, please include your updated statement in your cover letter. Guidelines for resubmitting your figure files are available below the reviewer comments at the end of this letter.

If applicable, we recommend that you deposit your laboratory protocols in protocols.io to enhance the reproducibility of your results. Protocols.io assigns your protocol its own identifier (DOI) so that it can be cited independently in the future. For instructions see: https://journals.plos.org/plosone/s/submission-guidelines#loc-laboratory-protocols. Additionally, PLOS ONE offers an option for publishing peer-reviewed Lab Protocol articles, which describe protocols hosted on protocols.io. Read more information on sharing protocols at https://plos.org/protocols?utm_medium=editorial-email&utm_source=authorletters&utm_campaign=protocols.

We look forward to receiving your revised manuscript.

Kind regards,

Janet E Rosenbaum, Ph.D.

Academic Editor

PLOS ONE

Journal Requirements:

Please review your reference list to ensure that it is complete and correct. If you have cited papers that have been retracted, please include the rationale for doing so in the manuscript text, or remove these references and replace them with relevant current references. Any changes to the reference list should be mentioned in the rebuttal letter that accompanies your revised manuscript. If you need to cite a retracted article, indicate the article’s retracted status in the References list and also include a citation and full reference for the retraction notice.

[Note: HTML markup is below. Please do not edit.]

Reviewers' comments:

Reviewer's Responses to Questions

**Comments to the Author**

1. If the authors have adequately addressed your comments raised in a previous round of review and you feel that this manuscript is now acceptable for publication, you may indicate that here to bypass the “Comments to the Author” section, enter your conflict of interest statement in the “Confidential to Editor” section, and submit your "Accept" recommendation.

Reviewer #3: All comments have been addressed

Reviewer #4: (No Response)

**********

2. Is the manuscript technically sound, and do the data support the conclusions?

The manuscript must describe a technically sound piece of scientific research with data that supports the conclusions. Experiments must have been conducted rigorously, with appropriate controls, replication, and sample sizes. The conclusions must be drawn appropriately based on the data presented. 

Reviewer #3: Yes

Reviewer #4: Partly

**********

3. Has the statistical analysis been performed appropriately and rigorously? 

Reviewer #3: Yes

Reviewer #4: No

**********

4. Have the authors made all data underlying the findings in their manuscript fully available?

The PLOS Data policy requires authors to make all data underlying the findings described in their manuscript fully available without restriction, with rare exception (please refer to the Data Availability Statement in the manuscript PDF file). The data should be provided as part of the manuscript or its supporting information, or deposited to a public repository. For example, in addition to summary statistics, the data points behind means, medians and variance measures should be available. If there are restrictions on publicly sharing data—e.g. participant privacy or use of data from a third party—those must be specified.

Reviewer #3: No

Reviewer #4: No

**********

5. Is the manuscript presented in an intelligible fashion and written in standard English?

PLOS ONE does not copyedit accepted manuscripts, so the language in submitted articles must be clear, correct, and unambiguous. Any typographical or grammatical errors should be corrected at revision, so please note any specific errors here.

Reviewer #3: Yes

Reviewer #4: Yes

**********

6. Review Comments to the Author

Please use the space provided to explain your answers to the questions above. You may also include additional comments for the author, including concerns about dual publication, research ethics, or publication ethics. (Please upload your review as an attachment if it exceeds 20,000 characters)

Reviewer #3: Thank you for addressing my comments and the opportunity to review this paper. No additional comments.

Reviewer #4: See comments in attached PDF.

**********

7. PLOS authors have the option to publish the peer review history of their article (what does this mean?). If published, this will include your full peer review and any attached files.

If you choose “no”, your identity will remain anonymous but your review may still be made public.

**Do you want your identity to be public for this peer review?** For information about this choice, including consent withdrawal, please see our Privacy Policy.

Reviewer #3: No

Reviewer #4: No

**********

[NOTE: If reviewer comments were submitted as an attachment file, they will be attached to this email and accessible via the submission site. Please log into your account, locate the manuscript record, and check for the action link "View Attachments". If this link does not appear, there are no attachment files.]

While revising your submission, please upload your figure files to the Preflight Analysis and Conversion Engine (PACE) digital diagnostic tool, https://pacev2.apexcovantage.com/. PACE helps ensure that figures meet PLOS requirements. To use PACE, you must first register as a user. Registration is free. Then, login and navigate to the UPLOAD tab, where you will find detailed instructions on how to use the tool. If you encounter any issues or have any questions when using PACE, please email PLOS at figures@plos.org. Please note that Supporting Information files do not need this step.

Attachment Submitted filename: PONE-D-21-13748_R2_reviewer-R2.pdf Click here for additional data file.

# Author comment: Author response to Decision Letter 2

**DOI:** 10.1371/journal.pone.0277074.r006

19 Aug 2022

Please see the Response to Reviewer documents for all our responses to the Reviewer comments.

Attachment Submitted filename: Response to Reviewer PONE-D-21-13748.docx Click here for additional data file.

# Peer review: Decision Letter 3

**DOI:** 10.1371/journal.pone.0277074.r007

**Authors:** Janet E Rosenbaum

20 Oct 2022

Women experiencing homelessness and mental illness in a Housing First multi-site trial: Looking beyond housing to social outcomes and well-being.

PONE-D-21-13748R3

Dear Dr. O'Campo,

We’re pleased to inform you that your manuscript has been judged scientifically suitable for publication and will be formally accepted for publication once it meets all outstanding technical requirements.

Within one week, you’ll receive an e-mail detailing the required amendments. When these have been addressed, you’ll receive a formal acceptance letter and your manuscript will be scheduled for publication.

An invoice for payment will follow shortly after the formal acceptance. To ensure an efficient process, please log into Editorial Manager at http://www.editorialmanager.com/pone/, click the 'Update My Information' link at the top of the page, and double check that your user information is up-to-date. If you have any billing related questions, please contact our Author Billing department directly at authorbilling@plos.org.

If your institution or institutions have a press office, please notify them about your upcoming paper to help maximize its impact. If they’ll be preparing press materials, please inform our press team as soon as possible -- no later than 48 hours after receiving the formal acceptance. Your manuscript will remain under strict press embargo until 2 pm Eastern Time on the date of publication. For more information, please contact onepress@plos.org.

Kind regards,

Janet E Rosenbaum, Ph.D.

Academic Editor

PLOS ONE

Additional Editor Comments (optional):

Reviewers' comments:

Reviewer's Responses to Questions

**Comments to the Author**

1. If the authors have adequately addressed your comments raised in a previous round of review and you feel that this manuscript is now acceptable for publication, you may indicate that here to bypass the “Comments to the Author” section, enter your conflict of interest statement in the “Confidential to Editor” section, and submit your "Accept" recommendation.

Reviewer #3: All comments have been addressed

Reviewer #4: (No Response)

**********

2. Is the manuscript technically sound, and do the data support the conclusions?

The manuscript must describe a technically sound piece of scientific research with data that supports the conclusions. Experiments must have been conducted rigorously, with appropriate controls, replication, and sample sizes. The conclusions must be drawn appropriately based on the data presented. 

Reviewer #3: Yes

Reviewer #4: Partly

**********

3. Has the statistical analysis been performed appropriately and rigorously? 

Reviewer #3: Yes

Reviewer #4: Yes

**********

4. Have the authors made all data underlying the findings in their manuscript fully available?

The PLOS Data policy requires authors to make all data underlying the findings described in their manuscript fully available without restriction, with rare exception (please refer to the Data Availability Statement in the manuscript PDF file). The data should be provided as part of the manuscript or its supporting information, or deposited to a public repository. For example, in addition to summary statistics, the data points behind means, medians and variance measures should be available. If there are restrictions on publicly sharing data—e.g. participant privacy or use of data from a third party—those must be specified.

Reviewer #3: Yes

Reviewer #4: No

**********

5. Is the manuscript presented in an intelligible fashion and written in standard English?

PLOS ONE does not copyedit accepted manuscripts, so the language in submitted articles must be clear, correct, and unambiguous. Any typographical or grammatical errors should be corrected at revision, so please note any specific errors here.

Reviewer #3: Yes

Reviewer #4: Yes

**********

6. Review Comments to the Author

Please use the space provided to explain your answers to the questions above. You may also include additional comments for the author, including concerns about dual publication, research ethics, or publication ethics. (Please upload your review as an attachment if it exceeds 20,000 characters)

Reviewer #3: Thank you for addressing our comments and providing us with the syntax. No further questions on my end.

Reviewer #4: Overall, clarity has much improved. There are still a few points that need to be clarified. See comments in attached PDF.

Another point not mention among the comments on the PDF is that sometimes p-values are reported in the text, some other times they are not and it's not entirely clear why. The authors may which to clarify this in the text with sentences like "for p-values see ...". Note that generally it is not enough to provide p-values only, but tables and reporting should also contain the value of the statistic and DFs used to get the specific p value.

**********

7. PLOS authors have the option to publish the peer review history of their article (what does this mean?). If published, this will include your full peer review and any attached files.

If you choose “no”, your identity will remain anonymous but your review may still be made public.

**Do you want your identity to be public for this peer review?** For information about this choice, including consent withdrawal, please see our Privacy Policy.

Reviewer #3: No

Reviewer #4: No

**********

Attachment Submitted filename: PONE-D-21-13748_R3_reviewer.pdf Click here for additional data file.

# Editor report: Acceptance letter

**DOI:** 10.1371/journal.pone.0277074.r008

**Authors:** Janet E Rosenbaum

22 Dec 2022

PONE-D-21-13748R3 

Women experiencing homelessness and mental illness in a Housing First multi-site trial: Looking beyond housing to social outcomes and well-being. 

Dear Dr. O'Campo:

I'm pleased to inform you that your manuscript has been deemed suitable for publication in PLOS ONE. Congratulations! Your manuscript is now with our production department. 

If your institution or institutions have a press office, please let them know about your upcoming paper now to help maximize its impact. If they'll be preparing press materials, please inform our press team within the next 48 hours. Your manuscript will remain under strict press embargo until 2 pm Eastern Time on the date of publication. For more information please contact onepress@plos.org.

If we can help with anything else, please email us at plosone@plos.org. 

Thank you for submitting your work to PLOS ONE and supporting open access. 

Kind regards, 

PLOS ONE Editorial Office Staff

on behalf of

Dr. Janet E Rosenbaum 

Academic Editor

PLOS ONE
